# Safety and efficacy of crenezumab in cognitively unimpaired carriers of the *PSEN1*^Glu280Ala^ mutation at risk for autosomal-dominant Alzheimer’s disease in Colombia (API ADAD Colombia Trial): a phase 2, randomised, double-blind, placebo-controlled trial

**DOI:** 10.1016/S1474-4422(25)00426-0

**Published:** 2026-02

**Authors:** Pierre N Tariot, Francisco S Lopera, Silvia Ríos-Romenets, Kaycee M Sink, Margarita Giraldo-Chica, Natalia Acosta-Baena, Gustavo Villegas, Alejandro Espinosa, Marisol Londoño Castaño, Claudia Muñoz, Paula Ospina, Yamile Bocanegra, Victoria Tirado, Eliana Henao, Eugenia Cardona, Ernesto Luna, Hugo Lopez, Gregorio Sánchez, Mario Muñoz Collazos, Sergio Alvarez, David Aguillón, Nan Hu, David Clayton, Tobias Bittner, Andres Schneider, Michael Dolton, Victor Poon, Jonas Nguyen, Ronald G Thomas, Lon S Schneider, Kewei Chen, Yi Su, Robert C Alexander, Yakeel T Quiroz, Yuqi Cai, Yi Ran Xu, Beth Ostaszewski, Dennis J Selkoe, Nicholas J Ashton, Marisa N Denkinger, Laura J Jakimovich, Rachelle S Doody, Jessica B Langbaum, Eric M Reiman

**Affiliations:** **Neuroscience Group of Antioquia (GNA), University of Antioquia, Medellín, Colombia** (Prof F S Lopera MD, S Ríos-Romenets MD, M Giraldo-Chica MD, N Acosta-Baena MD, A Espinosa MD, M Londoño Castaño MHSc, C Muñoz MA, P Ospina MA, Y Bocanegra PhD, V Tirado MSc, E Henao PhD, E Cardona, E Luna DSS, H Lopez MSc, D Aguillón MD PhD, Prof Y T Quiroz PhD); **Genentech, San Francisco, CA, USA** (K M Sink MD, N Hu PhD, D Clayton PhD, T Bittner PhD, M Dolton PhD, V Poon MS, J Nguyen MPH, Prof R S Doody MD PhD); **University of Antioquia, Medellín, Colombia** (G Villegas MSc); **Hospital San Juan de Dios de Yarumal, Antioquia, Colombia** (A Espinosa); **Cardiomet Cequin, Universidad del Quindío, Armenia, Colombia** (Prof G Sánchez MD); **San Juan de Dios Hospital, Armenia, Colombia** (Prof G Sánchez); **Marly’s Clinic, Bogotá, Colombia** (M Muñoz Collazos MD); **Hospital Pablo Tobón Uribe, Medellín, Colombia** (S Alvarez MD); **F Hoffmann-La Roche, Basel, Switzerland** (T Bittner, A Schneider MD, Prof R S Doody); **Herbert Wertheim School of Public Health and Human Longevity Science, University of California San Diego, San Diego, CA, USA** (Prof R G Thomas PhD); **Keck School of Medicine of the University of Southern California, Los Angeles, CA, USA** (Prof L S Schneider MD); **Banner Alzheimer’s Institute, Phoenix, AZ, USA** (Prof P N Tariot MD, Prof K Chen PhD, Y Su PhD, Prof R C Alexander MD, N J Ashton PhD, L J Jakimovich RN, J B Langbaum PhD, E M Reiman MD); **Departments of Psychiatry and Neurology, Harvard Medical School, Massachusetts General Hospital, Charlestown, MA, USA** (Prof Y T Quiroz); **Department of Neurology, Brigham and Women’s Hospital, Harvard Medical School, Boston, MA, USA** (Y Cai BS, Y Ran Xu BS, B Ostaszewski BS, Prof D J Selkoe MD); **Banner Sun Health Research Institute, Sun City, AZ, USA** (N J Ashton, M N Denkinger PhD); **Department of Psychiatry and Neurochemistry, Institute of Neuroscience and Physiology, the Sahlgrenska Academy at the University of Gothenburg, Mölndal, Sweden** (N J Ashton)

## Abstract

**Background:**

To have maximal benefit, Alzheimer’s disease-modifying treatments might need to be started before the onset of clinical symptoms. Mutations of the *PSEN1* gene are inherited as fully penetrant, autosomal-dominant traits, which almost always result in the clinical onset of Alzheimer’s disease before the age of 65 years. We aimed to evaluate the efficacy, including possible delayed emergence of cognitive impairment, and safety of crenezumab, an anti-amyloid monoclonal antibody, in cognitively unimpaired carriers of the *PSEN1*^Glu280Ala^ mutation at high imminent risk of developing symptoms due to Alzheimer’s disease.

**Methods:**

This 5–8-year common-close, double-blind, placebo-controlled, single-centre trial screened kindred members aged 30–60 years from the main health-care site in Medellín, Colombia. Participants who were cognitively unimpaired and carried the *PSEN1*^Glu280Ala^ autosomal-dominant mutation were randomly assigned 1:1 to receive placebo or subcutaneous crenezumab (investigators and participants were masked to treatment allocation), with an initial 300 mg dose every 2 weeks that increased to 720 mg every 2 weeks, and a later optional increase to 60 mg/kg intravenously every 4 weeks. Randomisation was stratified by age, education, *APOE* ε4 carrier status, and baseline Clinical Dementia Rating. Mutation non-carriers received placebo and were included in a 1:2 ratio of non-carriers to carriers to maintain genotype masking and include a genetic kindred control. Dual primary outcomes were the annualised rates of change in the Alzheimer’s Prevention Initiative (API) preclinical autosomal-dominant Alzheimer’s disease (ADAD) composite test total score and Free and Cued Selective Reminding Test–Cueing Index (FCSRT–CI) assessed in randomised participants who received at least one dose of the study drug, according to treatment assignment. Primary endpoints were assessed with a random coefficient regression model with a missing-at-random assumption adjusting for randomisation factors. Safety endpoints for mutation carriers were assessed in randomised participants who received at least one dose of the study drug. This trial is registered with ClinicalTrials.gov (NCT01998841) and is completed.

**Findings:**

619 Colombian API registrants were prescreened, 315 were assessed for eligibility, and 252 were enrolled (crenezumab–carrier, n=85; placebo–carrier, n=84; placebo–non-carrier, n=83; 160 [63%] women and 92 [37%] men) between Dec 20, 2013, and Feb 27, 2017. 237 (94%) completed the trial, with final data collection on March 22, 2022. The annualised rate of change in the API ADAD composite was −1·10 (SE 0·29) in the crenezumab group and −1·43 (0·29) in the placebo group (between-group difference 0·33 [95% CI −0·48 to 1·13]; p=0·43). The annualised rate of change in FCSRT–CI was −0·03 (0·00) in the crenezumab group and −0·04 (0·00) in the placebo group (between-group difference 0·01 [0·00 to 0·02]; p=0·16). All participants had at least one adverse event; serious adverse events occurred in 23 (27%) of 84 in the crenezumab group and 21 (25%) of 84 in the placebo group. No fatalities occurred.

**Interpretation:**

Crenezumab therapy administered for 5–8 years did not result in significant benefits on our primary clinical outcomes in cognitively unimpaired participants predisposed to developing ADAD dementia; secondary and exploratory outcomes also showed no significant effect on removal of amyloid plaques or other clinical or biomarker outcomes. Together with the results of other anti-amyloid β trials, robust fibrillar amyloid removal appears necessary for clinical efficacy in people with elevated brain amyloid. This study will further inform the biomarker, cognitive, and clinical trajectory of preclinical ADAD, the risk of clinical progression in amyloid-positive and amyloid-negative mutation carriers, and the size and design of future secondary and primary prevention trials.

## Introduction

Alzheimer’s disease is characterised by the presence of neurotoxic amyloid β plaques and tau tangles in the brain.^1,2^ Crenezumab is a humanised anti-amyloid β monoclonal IgG4 antibody, designed to bind monomeric and aggregated amyloid β, with higher affinity for oligomeric amyloid β.^3^ The low effector function of the IgG4 backbone and minimal binding to vascular amyloid might reduce brain vasculature inflammation and risk of amyloid-related imaging abnormalities (ARIA), permitting the administration of high antibody doses to safely achieve sufficient target engagement.

To have maximal benefit, Alzheimer’s disease-modifying treatments might need to be started before the onset of clinical symptoms and occurrence of extensive brain damage.^4^ Carriers of the autosomal-dominant Glu280Ala mutation in the presenilin 1 (*PSEN1*) gene are at nearly certain risk of developing symptoms of Alzheimer’s disease at a median age of 44 years.^5^ Members of this kindred (ie, a group of genetically related individuals) were enrolled in the Colombian Alzheimer’s Prevention Initiative (API) Registry, which was formally launched in March, 2010, and had the main goal of locating, enrolling, genotyping, and performing medical and cognitive evaluations of *PSEN1*^Glu280Ala^-carrier family members, and providing a resource for studies of autosomal-dominant Alzheimer’s disease (ADAD).^6^ The Banner Alzheimer’s Institute partnered with the University of Antioquia (Medellín, Colombia), Genentech, and other organisations in preparation for the trial to characterise Alzheimer’s disease-related biomarker changes in the kindred,^7^ expand the kindred,^6^ and develop an empirically derived cognitive measure to detect and track cognitive change before the onset of Alzheimer’s disease symptoms.^8^

We aimed to establish whether the prevention or delay of the onset of clinical symptoms of Alzheimer’s disease was possible, via the assessment of clinical and biomarker outcomes, in a homogeneous population of cognitively unimpaired individuals from an extended Colombian kindred carrying a single autosomal-dominant mutation (ie, Glu280Ala) in the *PSEN1* gene.^5,6^

## Methods

### Study design and participants

The API ADAD Colombia Trial was a phase 2, double-blind, placebo-controlled trial conducted at the main health-care site in Medellín, Colombia; three satellite health-care sites in remote areas provided investigational product administration and safety assessments. The API Colombia Registry recruited volunteers who were members of kindreds of interest. Eligibility criteria for this study included *PSEN1*^Glu280Ala^ kindred membership, being aged 30–60 years, being cognitively unimpaired and medically stable, having a study partner, having no history of treatment for Alzheimer’s disease, and having a bodyweight of 45–120 kg. Participants’ sex (male or female) was obtained from their national identification document (Cédula de Ciudadanía) in the registry and was confirmed via self-report. Participants were included irrespective of whether they had baseline florbetapir PET evidence of amyloid β plaques, defined by a mean cortical-to-cerebellar standardised uptake value ratio (SUVR) of 1·10 (24·3 centiloids).^9^ Enrolled participants were followed until the last randomised participant received 260 weeks of treatment, whereby the first and last participants enrolled concluded the trial at the same time; treatment periods ranged from 5 years to nearly 8 years. Assessments were performed as indicated in a protocol summary published previously.^10^

A Treatment Selection Advisory Committee vetted all potentially available candidate agents based on target engagement and safety and tolerability data, as described previously.^10^ Family members of the kindred were presented with masked profiles of the representative agents under consideration and were asked their preference (eg, anti-amyloid or other mechanism, route of administration, known clinical effects, and availability). The family members preferred an anti-amyloid agent with the optimal trade-off between potency and safety, preferably administered orally or subcutaneously. Crenezumab was selected based on its anti-oligomeric and monomeric amyloid antibody profile^3,11^ and Genentech’s willingness to share the API’s general scientific goals.

The trial was approved by the ethics committee at the Hospital Pablo Tobón Uribe, Medellín, Colombia, and the Colombian Health Authority, Instituto Nacional de Vigilancia de Medicamentos y Alimentos (ethics approval number PI-BA-796), monitored by an Independent Data Monitoring Committee, and conducted according to the principles of the Declaration of Helsinki and Good Clinical Practice guidelines. All participants and study partners provided written informed consent before undergoing any evaluations. The primary analysis occurred after all participants completed the assessments at week 260. This report summarises findings from the primary analysis performed on randomised trial data collected from Dec 20, 2013, to March 22, 2022. This trial was registered with ClinicalTrials.gov (NCT01998841) on Nov 22, 2013, and is completed.

### Randomisation and masking

To ensure that investigators and participants were masked to treatment allocation, a vendor of an interactive voice-based or web-based response system (not involved in the study) formulated a computer-generated sequence to randomly assign mutation carriers in a 1:1 ratio to receive crenezumab or placebo, stratified by age, education, *APOE* ε4 allele carrier status, and baseline Clinical Dementia Rating^12^ (CDR; zero *vs* non-zero). Mutation non-carriers, all of whom received placebo only, were included in a 1:2 ratio of non-carriers to carriers to maintain genotype masking and include a genetic kindred control. Study personnel preparing the study drug and the Independent Data Monitoring Committee were unmasked to treatment assignment; all other study personnel were masked. The success of masking techniques was not assessed.

### Procedures

The initial dose of crenezumab was 300 mg subcutaneously every 2 weeks. As more data became available from phase 1 and phase 2 crenezumab trials in sporadic Alzheimer’s disease, protocol amendments increased the crenezumab (and placebo) dose to 720 mg subcutaneously every 2 weeks. Later, after an optimal target dose was identified, an optional dose increase to 60 mg/kg intravenously every 4 weeks was offered. This final escalation was made optional to avoid late dropouts. These changes were based on internal and external data indicating that higher doses of anti-amyloid monoclonal antibodies were well tolerated and might be more effective.^11,13^ Once participants received intravenous dosing, they were not allowed to revert to subcutaneous dosing in an effort to maximise drug exposure in the most consistent way possible. Crenezumab was formulated as 180 mg/mL crenezumab in 200 mM arginine succinate and 0·05% (weight per volume) polysorbate 20, at pH 5·5, supplied in 6 mL glass vials as a 4 mL liquid. The placebo was formulated and supplied with the same method but without the active drug. All participants were allowed standard-of-care medications (eg, memantine and/or acetylcholinesterase inhibitors) if they developed symptomatic Alzheimer’s disease.

All primary, secondary, and exploratory outcome assessments were performed on the day of the specified visit, unless a time window was specified. Assessments scheduled on the day of study drug administration were performed before administration, unless otherwise noted. An end-of-treatment visit occurred 4 weeks after the last dose.

A subset of participants provided CSF at baseline and at years 2 and 5. Blood samples, including plasma, were collected from all participants at baseline and every year. Separate serial blood samples were collected for crenezumab serum pharmacodynamic and pharmacokinetic analyses at the same time points. Amyloid PET (ie, florbetapir PET), tau PET, [^18^F]fluorodeoxyglucose ([^18^F]FDG)-PET, MRI, and CSF and plasma data acquisition and analysis methods and measurements are described in more detail in the appendix (pp 3–5) and will be discussed further in a separate report that compares baseline and placebo-related biomarker findings in the mutation carrier and non-carrier groups.

Investigators assessed the occurrence of adverse events and serious adverse events at all participant evaluation timepoints. All events were recorded in the participants’ medical records and on the appropriate electronic case report form. Adverse events could also have been reported by participants in real time, in which case that timepoint would be used.

### Outcomes

Annualised rates of change in the API preclinical ADAD composite test total score (API ADAD composite) and the Free and Cued Selective Reminding Test–Cueing Index (FCSRT–CI) were dual primary outcomes. The API ADAD composite assesses overall cognitive function^8^ and the FCSRT–CI^14^ assesses episodic memory. The trial would be considered positive if either or both primary outcomes were significant at their specified α thresholds.

Secondary endpoints were predefined to support primary results and inform on the effect of treatment. The most important of these endpoints, designated as the key secondary outcome, was the annualised rate of change in mean cortical-to-white-matter florbetapir PET SUVR.^15^ The other secondary endpoints were time until an adjudicated clinical diagnosis of mild cognitive impairment (MCI) or dementia due to Alzheimer’s disease;^2,16^ the annualised rate of change in the CDR sum of boxes (CDR-SB), assessing global cognitive and functional status;^17^ the time to a CDR global score of more than 0; the annualised rate of change in the Repeatable Battery for the Assessment of Neuropsychological Status total score;^18^ the annualised rate of change in regional cerebral metabolic glucose rate (CMRgl) by use of FDG-PET; the annualised rate of change in volumetric measurements by use of MRI; and the annualised rate of change in CSF total tau and phosphorylated tau biomarkers. Efficacy endpoints were assessed by trained site raters who were unaware of the treatment assignments, participant genetic status, and ongoing trial information, including adverse events. The trial included prespecified, exploratory cognitive, clinical, and biomarker outcomes.

Safety endpoints included: the incidence, nature, and severity of treatment-emergent adverse events; withdrawal due to adverse events; the incidence of ARIA with oedema or effusion (ARIA-E) and ARIA with haemorrhage (ARIA-H); cerebral macrohaemorrhage; infusion-related and injection-related reactions; and anti-crenezumab antibodies. ARIA, cerebral macrohaemorrhage, and pneumonia were specified adverse events of special interest. Adverse events were coded with the Medical Dictionary for Regulatory Activities, version 25.0, and graded according to severity with the National Cancer Institute Common Terminology Criteria for Adverse Events, version 4.0, by the investigator. Serious adverse events were any adverse events that were fatal or life-threatening, required or extended hospitalisation, resulted in persistent or substantial disability or incapacity, resulted in a congenital anomaly or birth defect in a neonate born to a mother exposed to the study drug, or were considered to be serious medical events by the investigator.

The schedule of assessments and methods for all biomarkers used in the study are provided in the appendix (pp 3–6). Details regarding the methods used for all fluid and imaging biomarkers will be described in a separate publication (unpublished). Amyloid PET, FDG-PET, MRI scans, and plasma biomarkers were performed for all participants; Genentech Tau Probe 1 (GTP1; ie, tau) PET and CSF sampling were optional. Exploratory imaging and fluid biomarker assessments included baseline and annualised rates of change in: FDG-PET; CSF total Aβ42 and Aβ40, total tau and tau phosphorylated at position 181 (pTau181), total neurofilament light, S100 calcium-binding protein B, neurogranin, soluble triggering receptor expressed on myeloid cells 2 (sTREM2), glial fibrillary acidic protein (GFAP), chitinase-3-like protein 1 (YKL-40), α-synuclein, and interleukin-6; plasma total Aβ42, total Aβ40, pTau181, and tau phosphorylated at position 217 (pTau217; per initial protocol-specified assay and a revised, post-hoc assay), neurofilament light, GFAP, sTREM2, and YKL-40; and annualised rates of change in MRI whole brain, hippocampal, and ventricular volumes. The annualised rate of change was also measured for tau (GTP1 PET; all scans were performed after baseline as part of a protocol amendment). Annual changes in FDG-PET were assessed with an empirically predefined statistical region of interest preferentially affected by CMRgl decline in Alzheimer’s disease.^19^ An entorhinal-to-inferior-cerebellum SUVR was used as the tau burden index.^20,21^ Crenezumab serum pharmacokinetics were established with a validated analytical method, and CSF pharmacokinetics were assessed with a non-commercial enzyme-linked immunosorbent assay. In a post-hoc analysis, CSF oligomeric amyloid β was measured with an oligomeric amyloid β-preferring immunoassay platform that allows increased sensitivity compared with traditional immunodetection systems (appendix pp 3–4).

### Statistical analysis

The study was designed to enrol up to 200 participants in the mutation carrier cohort and 100 participants in the non-carrier cohort. Assuming a 25% dropout rate, two-sided testing at an overall α of 0·05, a placebo group coefficient of variation of 65% for the week 260 change scores, and 100 participants per arm, the study was designed to have at least 80% power to detect a true effect of a 30% difference between the crenezumab and placebo groups in mean decline on the API ADAD composite over 260 weeks.

Primary and secondary efficacy analyses were conducted on the modified intention-to-treat population (ie, participants who carried the *PSEN1*^Glu280Ala^ autosomal-dominant mutation causing early-onset Alzheimer’s disease and were treated with at least one dose of the study drug). Participants were grouped according to randomised treatment assignment.

Missing data were not imputed. Primary endpoints were assessed with a random coefficient regression model (RCRM) with a missing-at-random assumption to test the treatment effect on the annualised rate of change, adjusting for randomisation factors (age [≤38 years *vs* >38 years], education duration [<9 years *vs* ≥9 years], *APOE* genotype [*APOE* ε4 carrier *vs* non-carrier], and baseline CDR [zero *vs* non-zero]) as recorded in the interactive voice-based or web-based response system. All data, including those from after week 260, were used for the primary analysis. The type I error was split between testing the treatment effect on the API ADAD composite (α of 0·04) and on the FCSRT–CI (α of 0·01). A graph-based testing procedure was used to ensure the family-wise type I error was controlled at a two-sided α of 0·05 (appendix p 13).

Continuous secondary and exploratory endpoints were analysed with the same primary statistical model. Clinical exploratory endpoints include sensitivity analyses for the primary outcomes by use of mixed models for repeated measures (MMRM), the Kaplan–Meier plot for time to MCI or dementia due to Alzheimer’s disease, and the forest plot of primary outcomes according to baseline amyloid status. Time-to-event endpoints (time to MCI progression and time to non-zero in the CDR global score) were analysed with a stratified log-rank test with the primary analysis stratification factors. Within each time-to-event endpoint, the hazard ratio (HR) for recurrence was estimated with a stratified Cox proportional hazards model. All tests were based on a two-sided α of 0·05.

Safety endpoints were summarised with descriptive statistics in the safety-evaluable population (all mutation-carrier participants who were randomly assigned and received at least one dose of study drug), grouped according to treatment received. Mutation non-carriers were not included, partly to protect the genetic masking.

Pharmacokinetic and pharmacodynamic analyses were conducted with descriptive statistics. Mean serum and CSF crenezumab concentration and plasma Aβ40 and Aβ42 concentrations versus time were tabulated and plotted. Steady-state trough serum drug concentration was tabulated. Additional pharmacokinetic analyses were conducted as appropriate. An Independent Data Monitoring Committee periodically reviewed unblinded safety and efficacy data. Statistical analyses were performed using SAS version 9.4. Exposure-response analyses were conducted using R version 3.6.3.

### Role of the funding source

Genentech, F Hoffmann-La Roche, and Banner Alzheimer’s Institute had roles in the study design, study implementation, data collection, data management, data analysis, data interpretation, and writing of the report. The National Institute on Aging (NIA) served in an advisory capacity in the study design, vetted members of the Independent Data Monitoring Committee, and participated in blinded sessions of that committee, but had no role in data collection, data analysis, data interpretation, and writing of the report.

## Results

**Figure 1:**
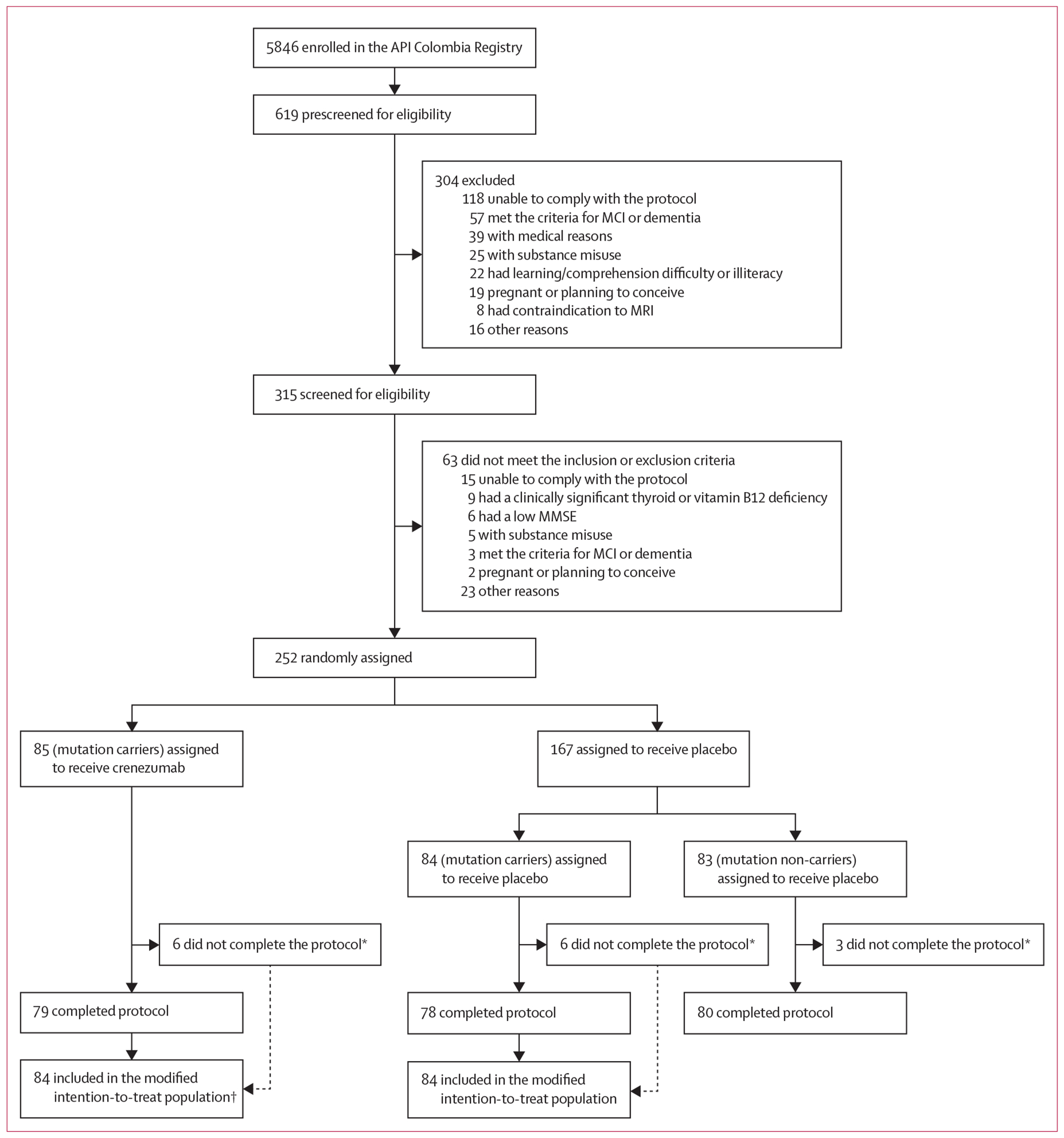
Trial profile API=Alzheimer’s Prevention Initiative. MCI=mild cognitive impairment. MMSE=Mini-Mental State Examination. *Of the 15 participants who did not complete the protocol, 12 withdrew consent, two discontinued due to non-compliance, and one withdrew due to an adverse event. †One participant assigned to receive crenezumab who did not complete the protocol did not receive at least one dose of the study drug.

A total of 5846 members of a *PSEN1*^Glu280Ala^ kindred were enrolled in the Colombian API Registry since its launch in March, 2010, including nearly 1200 mutation carriers.^6^ Trial enrolment began in Dec 20, 2013, and concluded in Feb 27, 2017. A genetically unmasked team referred 619 registrants for prescreening^10^ in tranches such that approximately 67% of trial participants would be mutation carriers; 304 registrants were excluded after prescreening and 315 consented and underwent eligibility assessment. 63 participants did not meet the eligibility criteria and 252 were randomly assigned (figure 1), representing the maximum feasible sample size given that registry expansion efforts were no longer fruitful. Among mutation carriers, 85 participants were assigned to receive crenezumab and 84 to receive placebo. Of these participants, 84 in each group received at least one dose of the study drug and were included in the modified intention-to-treat population for the assessment of the primary endpoints. 83 non-carrier participants were assigned to receive placebo. Details of the prescreening and screening processes have been published previously.^5,22^ Updated from a preliminary report,^22^ baseline demographics and characteristics are presented in table 1. There were no discrepancies between sex data retrieved from national identification documents and self-reported by participants. 18 (11%) of 169 carriers and five (6%) of 83 non-carriers had baseline CDR global scores of more than 0. Owing to the low educational background of the trial population, participants could receive a CDR score of 0·5 without having clinical evidence of a neurodegenerative disease. Per the protocol, these individuals were still classified as cognitively unimpaired because they did not meet the diagnostic criteria for MCI or dementia. 76 (45%) carriers versus 83 (100%) non-carriers were amyloid PET negative by protocol definition. Details regarding all other baseline biomarker characteristics are presented in the appendix (pp 7–8 or are unpublished). Baseline biomarker values were generally similar between the crenezumab-treated and placebo-treated carrier groups, including concentrations of oligomeric amyloid β. However, after adjusting for age, carriers had lower baseline concentrations of CSF total Aβ42 (p<0·0001), plasma total Aβ40 (p=0·0069), and FDG-PET (p<0·0001), and higher concentrations of amyloid PET (p<0·0001), CSF pTau181 (p<0·0001), CSF total tau (p<0·0001), CSF neurofilament light (p=0·0006), CSF neurogranin (p=0·0052), CSF GFAP (p<0·0001), CSF YKL-40 (p=0·0053), plasma pTau181 (p<0·0001), plasma pTau217 (p<0·0001), plasma neurofilament light (p<0·0001), and plasma GFAP (p<0·0001) compared with non-carriers.

**Figure 2:**
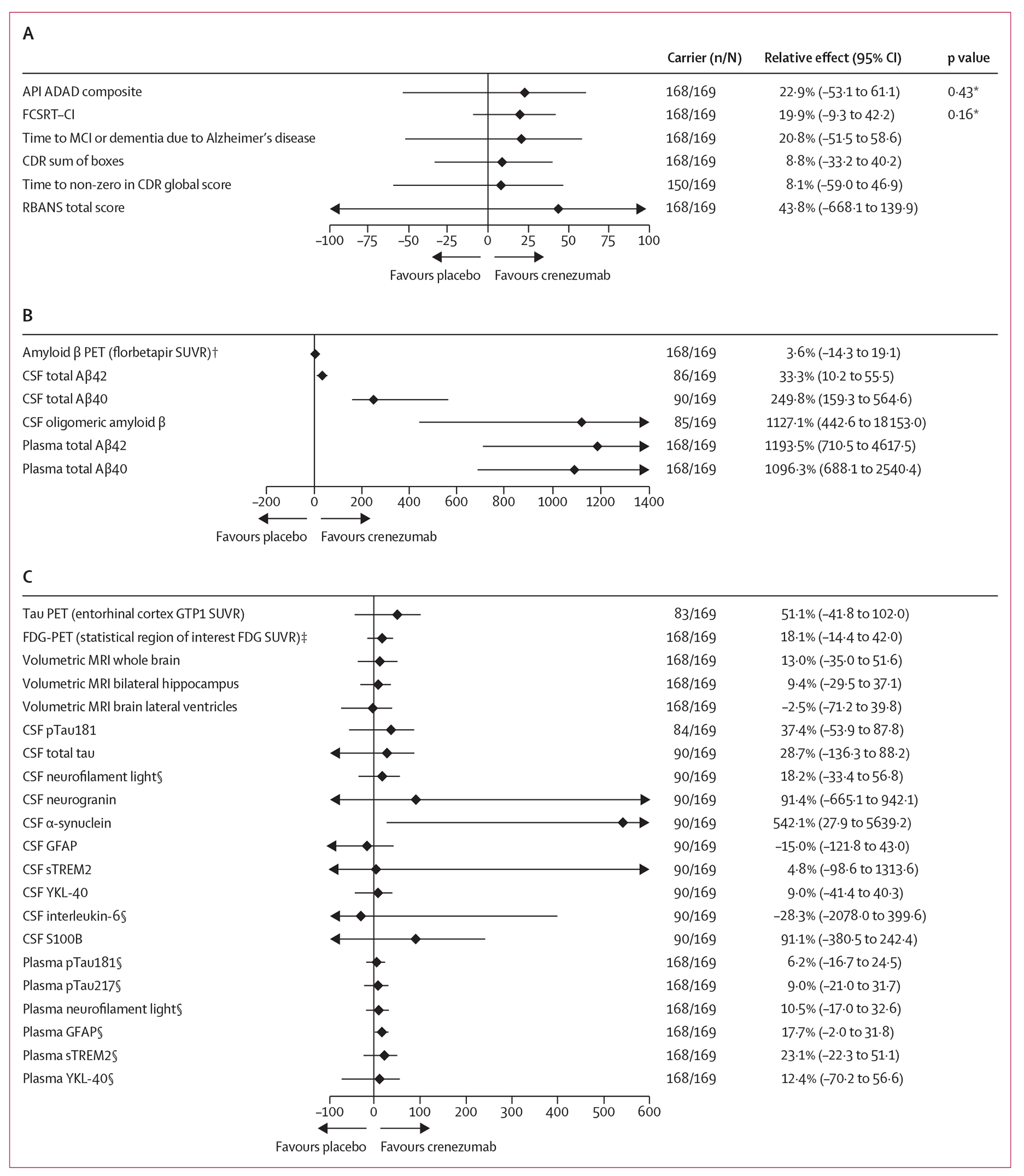
Treatment effects on clinical and biomarker outcomes Forest plots show mean relative reductions in annualised rates or hazards in the crenezumab carrier group compared with those in the placebo carrier group; 95% CIs are generated based on the bootstrap method for endpoints measured by annualised rate. (A) Summary of clinical treatment effects. Primary efficacy outcomes: the API ADAD composite (range 0–100; higher scores reflecting better cognitive performance),^8^ and FCSRT–CI (0–1; higher scores indicating better cognitive performance). Secondary outcomes: time until an adjudicated clinical diagnosis of MCI or dementia due to Alzheimer’s disease; annualised rate of change in the CDR sum of boxes, assessing global cognitive and functional status (0–18; higher scores indicating greater impairment); time to CDR global score of >0 (0–3; higher scores indicating greater impairment); and annualised rate of change in the RBANS total score (40–160; higher scores indicating less impairment). (B) Summary of amyloid biomarker treatment effects. Key secondary outcome: annualised rate of change in mean cortical-to-white-matter florbetapir PET SUVR. All other endpoints are exploratory. (C) Summary of non-amyloid biomarker treatment effects. Secondary outcomes: annualised rate of change in regional cerebral metabolic glucose rates via FDG-PET, annualised rate of change in volumetric measurements using MRI, and annualised rate of change in CSF total tau and phosphorylated biomarkers. All other endpoints are exploratory. ADAD=autosomal-dominant Alzheimer’s disease. API=Alzheimer’s Prevention Initiative. CDR=Clinical Dementia Rating. FCSRT–CI=Free and Cued Selective Reminding Test–Cueing Index. FDG=fluorodeoxyglucose. GFAP=glial fibrillary acidic protein. GTP1=Genentech Tau Probe 1. MCI=mild cognitive impairment. pTau181=tau phosphorylated at position 181. pTau217=tau phosphorylated at position 217. RBANS=Repeatable Battery for the Assessment of Neuropsychological Status. S100B=S100 calcium-binding protein B. sTREM2=soluble triggering receptor expressed on myeloid cells 2. SUVR=standardised uptake value ratio. YKL-40=chitinase-3-like protein 1. *Given that the p values of the dual primary endpoints were larger than the allocated α levels of 0·04 for the API ADAD composite and 0·01 for FCSRT–CI, the dual primary endpoints were not met. Thus, the key secondary endpoint specified in the α control could not be tested formally. Other than the dual primary endpoints, the reported 95% CIs for all comparisons have not been adjusted for multiplicity and cannot be used to formally infer treatment effects. †Key secondary outcome. ‡FDG-PET SUVR is computed as the ratio of a prespecified cortical region of interest and a prespecified reference region of interest that is preferentially associated with declines in Alzheimer’s disease-related cerebral metabolic glucose rates. §Reported as log_10_-transformed values.

The annualised rate of change in the API ADAD composite was −1·43 (SE 0·29) in the placebo group and −1·10 (0·29) in the crenezumab group. The between-group difference in the annualised rate of change of 0·33 (95% CI −0·48 to 1·13; p=0·43; figure 2A) was non-significant. The relative reduction in the annualised rate of change was 22·9% (95% CI −53·1 to 61·1). The annualised rate of change for the FCSRT–CI was −0·04 (SE 0·00) in the placebo group and −0·03 (0·00) in the crenezumab group. The between-group difference in the annualised rate of change of 0·01 (95% CI 0·00 to 0·02; p=0·16; figure 2A) was also non-significant. The relative reduction in the annualised rate of change was 19·9% (95% CI −9·3 to 42·2). The null results for the primary outcomes by amyloid status are shown in the appendix (p 16).

Figure 2A–C includes the forest plots of the dual primary outcomes and the secondary outcomes on the relative reduction scale. Figure 2B, C shows forest plots of the drug–placebo differences in annualised rates of change for amyloid and non-amyloid imaging and fluid biomarkers. The Kaplan–Meier curve for time to MCI or dementia due to Alzheimer’s disease, p=0·48 (log-rank) and HR 0·79 (95% CI 0·41–1·51), presented in the appendix (p 14) are exploratory in nature. CSF total Aβ42 concentrations decreased over time in both mutation carrier groups, but less so in crenezumab-treated carriers than in placebo-treated carriers (unadjusted p=0·005). CSF total Aβ40 concentrations did not change in mutation non-carriers and increased more in crenezumab-treated versus placebo-treated carriers (unadjusted p<0·0001; appendix p 15). Baseline CSF oligomeric amyloid β concentrations were not significantly different between the mutation carrier and non-carrier groups, did not change in mutation non-carriers, and increased more in crenezumab-treated carriers versus placebo-treated carriers (unadjusted p<0·0001; appendix p 21). Plasma total Aβ42 and plasma total Aβ40 concentrations increased more in crenezumab versus placebo carrier groups at year 5 (unadjusted p<0·0001). No treatment effects were observed for other serum or CSF biomarkers (figure 2B–C; appendix pp 30–33, 38–51; unpublished).

All participants had at least one adverse event (table 2). There were similar rates of serious and non-serious adverse events leading to treatment withdrawal between participants in the crenezumab group and the placebo group, and no fatalities. Adverse events of special interest are also presented in table 2. Incident ARIA-H (representing haemosiderin deposition, including cerebral microhaemorrhages and superficial siderosis) was uncommon (four [5%] participants on crenezumab, two [2%] on placebo); there were no cases of ARIA-E. Infusion-related and injection-site reactions were similar between groups; none were serious.

**Figure 3:**
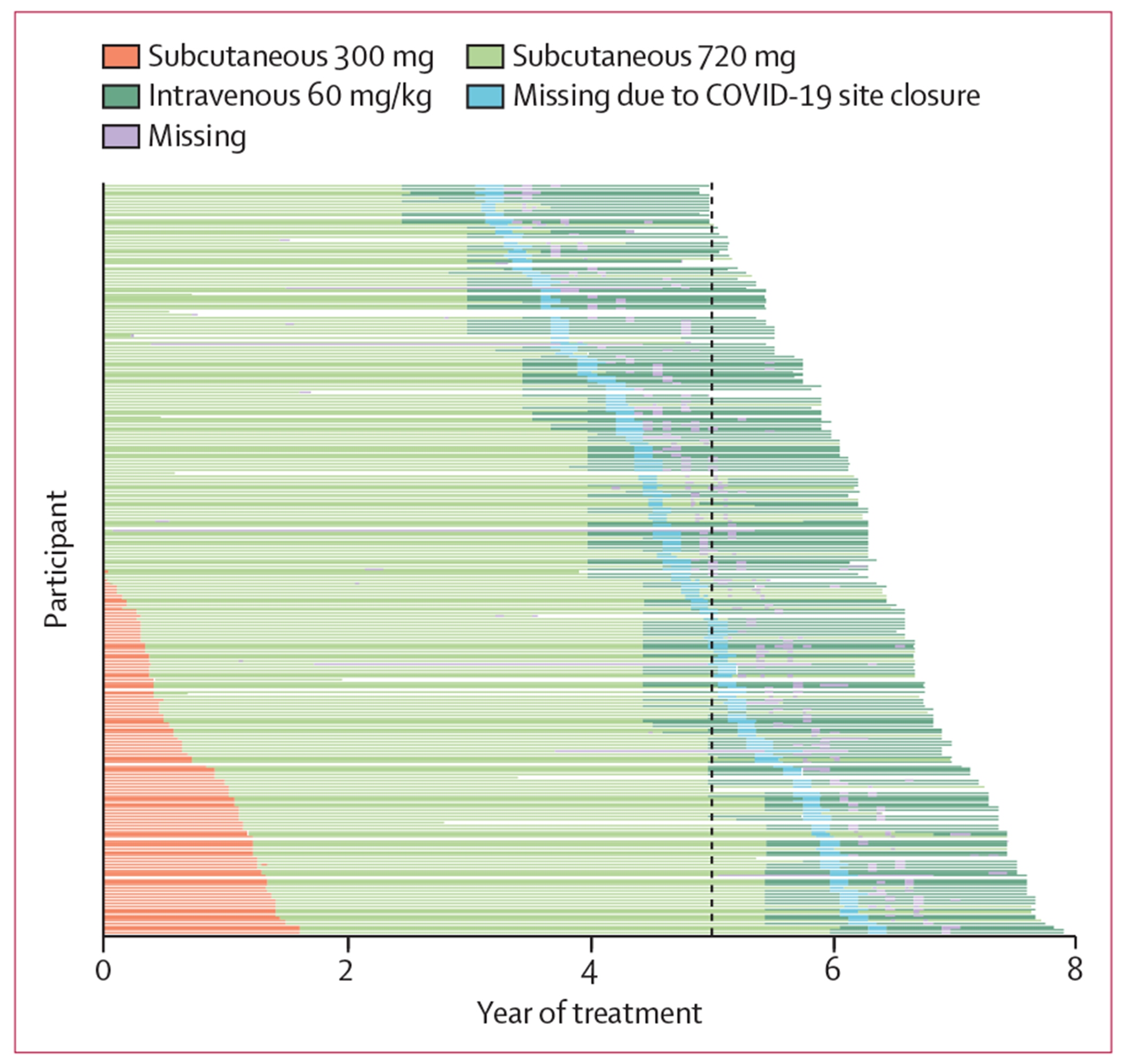
Treatment exposure Each horizontal line represents a participant, ordered based on their date of enrolment from top to bottom. Mean subcutaneous treatment duration: 4·3 years (SD 1·5). Mean intravenous treatment duration: 2·0 years (0·4). Mean subcutaneous dose intensity: 98·6% (2·9). Mean intravenous dose intensity: 88·2% (5·7). All participants, including both carriers and non-carriers, who received at least one dose of the study drug are included. Mean 6·1 years (1·4) of subcutaneous and/or intravenous treatments combined, up to 7·9 years.

Figure 3 shows the changes in the study drug dose per participant over time. Although 237 (94%) participants completed the protocol and 228 (90%) completed the trial on treatment, most of those who reached the target dose of 60 mg/kg intravenously every 4 weeks did not do so until after year 4 of the trial, and several had already progressed clinically by this time. Mean duration of subcutaneous exposure was 4·3 years (SD 1·5) and intravenous exposure was 2·0 years (0·4). During the COVID-19 pandemic, the site closed briefly with corresponding missed doses; however, given the long duration of the trial and the slope-based analyses (RCRM), the effect of COVID-19 on results was negligible. Overall, 3·3% of participants’ crenezumab exposure was affected by the COVID-19 site closure. Post-hoc exposure–response analyses showed no significant relationship between exposure and clinical or biomarker outcomes (appendix pp 33–34), except for the relationships between CSF crenezumab concentrations and CSF total Aβ40 (unadjusted p=0·0017) and CSF oligomeric amyloid β (unadjusted p<0·0001).

A prespecified sensitivity analysis of the API ADAD composite change from baseline was performed with MMRM, showing a difference in adjusted means of 2·1 (SE 1·4; 95% CI −0·78 to 4·9) in year 5. A similar analysis for change in FCSRT–CI at week 260 showed a difference in adjusted means of 0·07 (0·03; 0·01–0·12; appendix pp 52–53). These findings affirmed the null result of the primary analysis.

To evaluate target engagement, an exploratory pharmacokinetic–pharmacodynamic analysis was performed that showed no significant relationships between pharmacokinetic and pharmacodynamic effects for tau PET slope, CSF pTau181, total tau, total Aβ40, and total Aβ42 by either a categorical or a linear regression model (appendix pp 54–55).

## Discussion

Crenezumab therapy administered for 5–8 years showed no significant difference from placebo in annualised rate of change of the API ADAD composite or FCSRT–CI. Results from secondary and selected exploratory measures were consistent with the primary results. Injection and infusion reactions were the most common adverse events, occurring at similar rates in mutation carriers receiving crenezumab or placebo. There was no ARIA-E, and ARIA-H occurred at a low rate.

Roughly 45% of carriers did not meet the baseline florbetapir PET criteria for the presence of moderately frequent neuritic amyloid β plaques, providing an opportunity to explore treatment effects when started before or after amyloid β plaques are present. No differential effects of crenezumab and placebo were seen in this subgroup. We did not examine subgroups of participants with a CDR of 0·0 versus 0·5 at baseline because both groups were deemed to be cognitively unimpaired. The absence of differences in oligomeric amyloid β in the baseline carrier versus non-carrier groups could reflect limitations in the assay, the concentration in CSF relative to that in the brain, and/or the much lower concentrations of oligomers that are thought to occur independent of amyloid plaques.

Crenezumab treatment was not associated with significant effects on amyloid plaque burden or downstream Alzheimer’s disease biomarkers. However, CSF findings showed significantly greater total Aβ40 increases and less total Aβ42 decline in the crenezumab-treated carrier group compared with the placebo-treated carrier group. We postulate that the findings reflect crenezumab binding to these amyloid β species in the CNS, slower CSF clearance of crenezumab-bound amyloid species, and attenuation of the CSF total Aβ42 decline associated with amyloid β plaque deposition during the preclinical stages of Alzheimer’s disease.

In a post-hoc analysis, crenezumab treatment was associated with a significant increase in CSF oligomeric amyloid β concentrations that correlated with CSF crenezumab concentrations. This increase might have been due to increased clearance of oligomeric amyloid β from the brain into CSF and reduced clearance of the antibody-bound oligomeric amyloid β from CSF. The oligomeric amyloid β assay did not detect CSF amyloid β monomers (unpublished). These data suggest, without proving, that crenezumab achieved at least partial target engagement of soluble amyloid β oligomers, which might contribute to synaptotoxicity in Alzheimer’s disease.^1^ Further work on the analysis of amyloid β oligomers in human fluids is needed.

Crenezumab target engagement was evaluated via an exploratory exposure–response analysis relating crenezumab pharmacokinetics in serum or CSF with relevant pharmacodynamic metrics, including tau PET slope and change from baseline CSF tau and amyloid β indices, finding no significant relationships between crenezumab pharmacokinetic and pharmacodynamic effects. Although these results could be interpreted to indicate that a higher dose of crenezumab would not have led to larger changes in these pharmacodynamic metrics, the multiple dose changes in the study are likely to substantially confound this analysis, and the results should be interpreted with caution.

We postulate that the absence of observable effects of crenezumab on amyloid β plaque measurements might at least partly explain the absence of a clinical effect. Amyloid β plaque clearance is associated with clinical benefit in symptomatic patients receiving amyloid β plaque-clearing antibodies,^23–25^ whereas agents with an amyloid β-lowering ability produced little clinical benefit.^26,27^ Other factors, including several limitations of this study, could include: insufficient engagement of pathophysiologically relevant central oligomeric amyloid β (eg, due to competitive binding to Aβ42 and abundant Aβ40 monomers or absence of binding of crenezumab to relevant oligomeric amyloid β species), the minimal induction of microglia by crenezumab needed to remove amyloid β fibrils and the oligomeric amyloid β that might bind to fibrillar plaques,^28^ or inadequate statistical power. On this last point, for one of the primary endpoints—the API ADAD composite change from baseline at year 5—the signal-to-noise ratio was approximately eight times worse than expected. The API ADAD composite was developed empirically in an observational study of the same *PSEN1*^Glu280Ala^ kindred. This observational cohort differed in important ways from the API trial population, including in age, education, and cognitive performance. Despite the primary analysis being amended to use a more powerful RCRM approach than the traditional MMRM approach for detecting the treatment effect at year 5, with a missing-at-random assumption to test the treatment effect on annualised rates of change, the high study retention rate (the original assumption had been 5% dropouts per year), and the longer-than-planned trial duration that used a common-close design, the statistical power is still lower than expected.^29^ In addition, the low rate of amyloid positivity at baseline might have limited the ability of crenezumab to have clinical benefit, and, despite the length of the trial, the amyloid-negative participants might not have been able to show clinical decline. Insufficient dosing is a substantial concern given that only some participants reached the maximal target dose and that they reached this dose for a relatively short time. Multiple dose changes complicate the ability to discern a drug effect. Although the absence of correlation between fluid biomarkers and crenezumab pharmacokinetics suggests that inadequate exposure might not be implicated in the observed absence of efficacy, this possibility cannot be definitively ruled out.

The trial had several strengths and a major impact on Alzheimer’s disease prevention research. It was the first US National Institutes of Health (NIH)-supported trial of a putative Alzheimer’s disease-modifying treatment in cognitively unimpaired participants at biological risk for Alzheimer’s disease. It had the potential to investigate primary versus secondary prevention effects of crenezumab and to characterise the relationship between the biomarker and clinical effects of crenezumab. It was conducted in the world’s largest known ADAD kindred, carrying a single Alzheimer’s disease-causing mutation. The trial used communication strategies, social support programmes, and ethical considerations in a population that is vulnerable owing to economic barriers, which were valued by the participants and contributed to exceptional adherence and retention rates.^30^ Trial data and samples will be made available to inform the design of future secondary and primary prevention trials. Exploratory clinical outcomes and additional exploratory CSF and plasma biomarker assessments will be reported separately.

We believe that addressing the amyloid burden in this population merits further study with agents that have different mechanisms of action. Together with the findings of a separate analysis of longitudinal biomarker changes (unpublished) and statistical analysis innovations within this study,^29^ learnings from the API ADAD study can further inform the biomarker, cognitive, and clinical trajectory of preclinical ADAD, the risk of subsequent clinical progression in amyloid-positive and amyloid-negative mutation carriers, and the size and design of future secondary and primary prevention trials using biomarker and clinical endpoints. Based partly on these data, we have undertaken a new NIH-funded trial (NCT06996730) that will evaluate the individual and relative efficacy and safety, and potential synergy, of donanemab (with the ability to remove amyloid plaques, which will be confirmed during the trial using amyloid PET scans), nivegacetor (a γ-secretase modulator previously called RG6289, which might block the reaccumulation of brain amyloid after its removal), and their combination, to achieve and/or maintain low concentrations of brain amyloid in asymptomatic and symptomatic *PSEN1*^Glu280Ala^ mutation carriers and beneficially affect downstream biomarkers and clinical and other biomarker outcomes. Treatment with crenezumab had no meaningful effect on clinical or selected biomarkers of preclinical Alzheimer’s disease progression, with many caveats previously noted. With the absence of clinical efficacy of solanezumab—another antibody without effects on amyloid plaques—to delay clinical progression in a secondary prevention trial,^26^ the collective data provide further support to the hypothesis that robust fibrillar amyloid removal is necessary for clinical efficacy. The results do not preclude the possibility that different agents targeting oligomeric amyloid β could have effects on amyloid removal and clinical outcomes.

Although the API ADAD Colombia trial did not show significant effects of crenezumab treatment on its prespecified clinical, cognitive, and main biomarker endpoints, it introduced new frameworks to evaluate Alzheimer’s disease prevention therapies, support their potential regulatory approval, and inform the relationship between the biomarker and clinical effects of a treatment. It introduced strategies to engage research participants who are at risk and optimise their continued participation in this study and others. It provided a precedent-setting commitment to share trial data and samples (including in study drug-treated and placebo-treated mutation carriers and placebo-treated non-carriers) that could further inform the study of preclinical Alzheimer’s disease and the size and design of other prevention trials. It helped set the stage for a growing number of stakeholders to advance the evaluation and potential approval of Alzheimer’s disease prevention therapies that can substantially delay or prevent disease progression in people at risk for developing symptomatic Alzheimer’s disease.

## Supplementary Material

1

## Figures and Tables

**Table 1:** Baseline demographics and characteristics in the intention-to-treat population

	Crenezumab (carrier; n=85)	Placebo (carrier; n=84)	Placebo (non-carrier; n=83)
Age, years	36·8 (5·3)	36·9 (6·3)	43·3 (7·2)
>38 years*	31 (36%)	28 (33%)	58 (70%)

Sex			
Female	44 (52%)	59 (70%)	57 (69%)
Male	41 (48%)	25 (30%)	26 (31%)

Education duration ≥9 years*	48 (56%)	47 (56%)	38 (46%)

One or more *APOE* ε4 alleles*	19 (22%)	17 (20%)	19 (23%)

CDR global score of 0*	77 (91%)	74 (88%)	78 (94%)

CDR sum of boxes	0·16 (0·38)	0·14 (0·43)	0·05 (0·17)

API ADAD composite	81·93 (8·82)	80·44 (11·29)	83·70 (9·83)

FCSRT–CI	0·78 (0·16)	0·76 (0·20)	0·83 (0·14)

MMSE total score	28·85 (1·27)	28·79 (1·51)	29·19 (1·03)

Neuropsychiatric Inventory	0·26 (0·89)	0·64 (2·16)	0·37 (1·95)

RBANS total score	68·44 (11·79)	67·80 (13·50)	74·82 (11·52)

Amyloid PET positive	52 (61%)	41 (49%)	0
Amyloid PET SUVR†	1·15 (0·15)	1·11 (0·12)	0·96 (0·04)
Amyloid PET centiloids	33·41 (27·24)	25·36 (21·43)	−2·18 (8·23)

FDG-PET, SUVR‡	1·29 (0·04)	1·29 (0·04)	1·30 (0·04)

Whole brain volumetric MRI, mL	1187·65 (116·14)	1150·80 (89·91)	1144·84 (112·44)

Hippocampal volumetric MRI, mL	8·38 (0·85)	8·24 (0·73)	8·24 (0·76)

Lateral ventricles volumetric MRI, mL	13·34 (6·14)	13·39 (5·64)	14·14 (6·75)

CSF pTau181, pg/mL§	20·23 (18·36); n=48	19·13 (11·84); n=42	11·02 (5·18); n=37

CSF total tau, pg/mL§	197·91 (123·94); n=48	202·95 (95·08); n=42	137·61 (58·32); n=36

CSF neurofilament light (log_10_), pg/mL§	1·71 (0·33); n=48	1·72 (0·20); n=42	1·66 (0·15); n=37

Data are mean (SD) or n (%). The intention-to-treat population includes all participants who were randomly assigned, whether or not the participant received the assigned treatment. ADAD=autosomal-dominant Alzheimer’s disease. API=Alzheimer’s Prevention Initiative. CDR=Clinical Dementia Rating. FCSRT–CI=Free and Cued Selective Reminding Test–Cueing Index. FDG=fluorodeoxyglucose. GTP1=Genentech Tau Probe 1. MMSE=Mini-Mental State Examination. pTau181=tau phosphorylated at threonine 181. RBANS=Repeatable Battery for the Assessment of Neuropsychological Status. SUVR=standardised uptake value ratio.

* Stratification variables.

† Whole cerebellum was used as the reference region; >1·1 was defined as positive. Baseline tau PET measurements are not available because GTP1 PET was introduced later in the trial.

‡ FDG SUVR is computed as the ratio of a prespecified cortical region of interest and a prespecified reference region of interest that is preferentially associated with declines in Alzheimer’s disease-related cerebral metabolic glucose rate.

§ A subset of participants took part in the optional CSF substudy.

**Table 2:** Summary of adverse events in mutation carriers

	Crenezumab (n=84)	Placebo (n=84)
Participants with any adverse event	84 (100%)	84 (100%)
Total number of adverse events	2164	2295

Participants with any serious adverse event	23 (27%)	21 (25%)
Total number of serious adverse events	30	22

NCI CTCAE grade ≥3	18 (21%)	23 (27%)

Participants with at least one adverse event resulting in treatment discontinuation	2 (2%)	2 (2%)

Death	0	0

Adverse events by MedDRA preferred term with incidence ≥15% in either group		
Nasopharyngitis	81 (96%)	81 (96%)
Headache	53 (63%)	54 (64%)
Back pain	37 (44%)	40 (48%)
Dizziness	27 (32%)	35 (42%)
Infusion-related reaction	25 (30%)	24 (29%)
Arthralgia	19 (23%)	20 (24%)
Gastroenteritis	18 (21%)	24 (29%)
Urinary tract infection	17 (20%)	21 (25%)
Abdominal pain	21 (25%)	16 (19%)
Gastritis	19 (23%)	22 (26%)
Tonsilitis	16 (19%)	17 (20%)
Neck pain	11 (13%)	19 (23%)
Insomnia	13 (15%)	18 (21%)
Pain in extremity	14 (17%)	17 (20%)
Vaginal infection	15 (18%)	15 (18%)
Diarrhoea	19 (23%)	14 (17%)
Depression	16 (19%)	17 (20%)
Urticaria	19 (23%)	10 (12%)
Hypertriglyceridaemia	21 (25%)	10 (12%)
Injection-site haemorrhage	10 (12%)	13 (15%)

Serious adverse events by MedDRA system organ class with incidence ≥2% in either group		
Infections and infestations	5 (6%)	5 (6%)
Pregnancy, puerperium, and prenatal conditions	5 (6%)	3 (4%)
Injury, poisoning, and procedural complications	3 (4%)	2 (2%)
Renal and urinary disorders	3 (4%)	2 (2%)
Reproductive system and breast disorders	2 (2%)	2 (2%)
Psychiatric disorders	2 (2%)	1 (1%)
Gastrointestinal disorders	2 (2%)	1 (1%)
Cardiac disorders	0	3 (4%)

Adverse events of interest		
ARIA-E	0	0
ARIA-H	4 (5%)	2 (2%)
Cerebral intraparenchymal macrohaemorrhage	0	0
Pneumonia	0	0
Injection-related reactions	34 (40%)	31 (37%)
Infusion-related reactions	25 (30%)	24 (29%)

Data are n or n (%). ARIA-E=amyloid-related imaging abnormalities–oedema. ARIA-H=amyloid-related imaging abnormalities–haemorrhage. MedDRA=Medical Dictionary for Regulatory Activities. NCI CTCAE=National Cancer Institute Common Terminology Criteria for Adverse Events.

## Data Availability

The study protocol and statistical analysis plan are available in the appendix (pp 57–255). In accordance with the API policies and Collaboration for Alzheimer’s Prevention (CAP) data-sharing guidelines, Banner Alzheimer’s Institute and Roche have made the deidentified participant data and biological samples available to qualified researchers. Access is granted to researchers who meet NIH controlled-access criteria for human subject research including submission of a written research plan, curriculum vitae or NIH-style biosketch, and formal agreement to data-use terms. Deidentified clinical data and trial imaging are hosted at, and can be requested from the University of Southern California Laboratory of Neuroimaging (USC LONI) at https://ida.loni.usc.edu/ login.jsp. Residual study biomaterial is accessible by request through the National Cellular Repository for Alzheimer’s Disease (NCRAD) at https://ncrad.iu.edu/access-samples/available-samples/api-adad; requests are reviewed by an independent panel for scientific merit and feasibility. Data and samples at USC LONI and NCRAD are harmonised to enable cross-resource analysis. All data have been made available before publication. Inquiries regarding study data and biomaterial including access requests can also be made directly APIData@bannerhealth.com.

## Research in context

### Evidence before this study

We searched PubMed and Embase for articles published in English and other languages from Jan 1, 2010, to Sept 30, 2025, using combinations of Medical Subject Headings and the free-text terms “Alzheimer’s”, “autosomal dominant Alzheimer’s disease”, “prevention”, biomarker”, “surrogate biomarker”, “composite cognitive test scores”, “Alzheimer’s disease biomarkers”, “amyloid”, “regulatory policy”, and “data sharing”. Our review of the existing literature was further informed by private meetings with pharmaceutical companies; symposia and scientific conferences; discussions with researchers affiliated with the Alzheimer’s Prevention Initiative (API), Dominantly Inherited Alzheimer’s Network, the Alzheimer’s Clinical Trials Consortium (ACTC), and the Collaboration for Alzheimer’s Prevention; and other meetings. At the time of conception of the API autosomal-dominant Alzheimer’s disease (ADAD) Colombia Trial, the approved treatments for established Alzheimer’s disease dementia showed only modest efficacy and did not change the disease course. Contemporary clinical trials in preclinical Alzheimer’s disease were not feasible due to the need for large sample sizes, long follow-up periods to show an effect, and sensitive assessment tools capable of discerning cognitive decline in people who are at risk who do not have cognitive impairment and biomarkers that can be used to predict and monitor disease progression.

### Added value of this study

We conducted this trial to establish whether the prevention or delay of the clinical onset of Alzheimer’s disease was possible, as assessed with clinical and biomarker outcomes, in a homogeneous population of cognitively unimpaired individuals from an extended Colombian kindred carrying a single autosomal-dominant mutation in the *PSEN1* gene (Glu280Ala). This trial was the first National Institutes of Health-supported trial of a putative Alzheimer’s disease-modifying treatment in cognitively unimpaired participants predisposed to the clinical onset of Alzheimer’s disease. It was conducted in the world’s largest known ADAD kindred affected by a single Alzheimer’s disease-causing mutation and helped set the stage for other prevention trials to follow.

### Implications of all the available evidence

Robust fibrillar amyloid removal appears necessary to achieve clinical efficacy in people who already have elevated brain amyloid. Our results do not rule out the possibility that different anti-oligomeric agents could confer benefit. Our trial also introduced new strategies to evaluate and support the potential approval of drug therapies in cognitively unimpaired people at biological risk for Alzheimer’s disease and a precedent-setting agreement to share trial data and samples to further inform the study of preclinical Alzheimer’s disease and the design of future trials.
