# Supplementary material for: Safety and efficacy of crenezumab in cognitively unimpaired carriers of the PSEN1Glu280Ala mutation at risk for autosomal-dominant Alzheimer’s disease in Colombia (API ADAD Colombia Trial): a phase 2, randomised, double-blind, placebo-controlled trial
Source: Lancet Neurol. Author manuscript; Available in PMC 2026 Mar 17. (PMC12994566; doi:10.1016/S1474-4422(25)00426-0)
Supplement: 1 [file NIHMS2142445-supplement-1.pdf]

THE LANCET
Neurology

Supplementary appendix

This appendix formed part of the original submission and has been peer reviewed. We post it as supplied by the authors.

Supplement to: Tariot PN, Lopera FS, Ríos-Romenet S, et al. Crenezumab in cognitively unimpaired carriers of the *PSEN1*^{Glu280Ala} mutation at risk for autosomal-dominant Alzheimer's disease in Colombia (API ADAD Colombia Trial): a phase 2, randomised, double-blind, placebo-controlled trial. *Lancet Neurol* 2026; **25:** 147–59.

SUPPLEMENTARY APPENDIX

Supplementary appendix for: Crenezumab in cognitively unimpaired *PSEN1 E280A* mutation carriers at risk for autosomal-dominant Alzheimer's disease in Colombia (API ADAD Colombia Trial): a phase 2, randomised, double-blind, placebo-controlled trial

CONTENTS

List of investigators **2**

Supplementary methods **3**

Statistical analysis 3

Cerebrospinal fluid (CSF) biomarker collection and analysis 3

CSF oAβ analysis 3

Blood biomarker collection and analysis 4

PET image acquisition and analysis 4

MRI acquisition and analysis 5

Supplementary tables **6**

Supplementary Table 1: Schedule of biomarker assessments in the API ADAD study 6

Supplementary Table 2: Baseline biomarker levels in crenezumab- vs placebo-treated mutation carriers and placebo-treated non-carriers 7

Supplementary Table 3: Summary of all reported AEs in mutation carriers by system organ class 9

Supplementary Table 4: Summary of clinical and biomarker treatment effects in mutation carriers at Week 260 (RCRM analyses) 30

Supplementary Table 5: Summary of clinical and biomarker treatment effects in mutation carriers who received crenezumab compared with placebo at Week 260 (MMRM analyses) 32

Supplementary figures **34**

Figure S1: Graph-based testing procedure for type I error control 34

Figure S2: Kaplan–Meier curve for time to MCI or dementia due to AD 35

Figure S3: Total CSF Aβ40 on crenezumab vs placebo in mutation carriers 36

Figure S4: Primary outcomes by baseline amyloid status 37

Figure S5: Biomarker treatment effects in crenezumab- vs placebo-treated mutation carriers (A–Z) 38

Figure S6: Dual primary endpoint sensitivity analysis: MMRM 52

Figure S7: PK/PD analysis 54

References **56**

Study protocol **57**

Statistical analysis plan **204**

List of investigators

Francisco Lopera *(Neuroscience Group of Antioquia, University of Antioquia, Medellín, Colombia)*, Silvia Ríos-Romenets *(Neuroscience Group of Antioquia, University of Antioquia, Medellín, Colombia)*, Margarita Giraldo-Chica *(Neuroscience Group of Antioquia, University of Antioquia, Medellín, Colombia)*, Natalia Acosta-Baena *(Neuroscience Group of Antioquia, University of Antioquia, Medellín, Colombia)*, Claudia Ramos *(Neuroscience Group of Antioquia, University of Antioquia, Medellín, Colombia)*, Claudia Aponte *(Neuroscience Group of Antioquia, University of Antioquia, Medellín, Colombia)*, Laureano Mesta *(Neuroscience Group of Antioquia, University of Antioquia, Medellín, Colombia)*, Ramiro Martinez *(Neuroscience Group of Antioquia, University of Antioquia, Medellín, Colombia),* Alejandro Espinosa *(Neuroscience Group of Antioquia, University of Antioquia, Medellín, Colombia and Hospital San Juan de Dios de Yarumal, Antioquia, Colombia),* Carlos Tobón *(Universidad de Antioquia, School of Medicine, Medellín, Colombia)*, Gregorio Sánchez *(Cardiomet Cequin, Universidad del Quindío, and San Juan de Dios Hospital, Armenia, Colombia)* Mario Muñoz Collazos *(Marly's Clinic, Bogotá, Colombia)*

Supplementary methods

Statistical analysis

A graph-based testing procedure was used to ensure the family-wise type I error would be controlled at a two-sided α=0·05 (figure S1).[1-3] If both endpoints were significant, key secondary endpoints would be tested hierarchically at α=0·05. The hierarchy was as follows: annualised rate of change in mean fibrillar amyloid accumulation in a composite region with a subcortical white matter reference region using florbetapir positron emission tomography, time to progression from preclinical Alzheimer's disease (AD) to mild cognitive impairment due to AD or from preclinical AD to dementia due to AD, annualised rate of change in the Clinical Dementia Rating Scale – Sum of Boxes, time to progression to non-zero in Clinical Dementia Rating Scale – Global Score, and annualised rate of change in a measure of overall neurocognitive functioning: Repeatable Battery for the Assessment of Neuropsychological Status total score.

Cerebrospinal fluid (CSF) biomarker collection and analysis

Participants were encouraged but not required to have lumbar punctures to collect CSF at baseline and Years 2 and 5 follow-up visits. At each time point, approximately 22 mL of CSF was collected into 10 mL syringes (standard hospital supply) using a 24 G Sprotte needle (order number 121151-30A, Pajunk GmbH, Germany) by lumbar puncture at L4/L5 (the first 1–2 mL of CSF was discarded if the CSF was pink). 0·5 mL of the CSF from the first syringe was distributed into 1·5 mL LoBind® PP tubes (order number 022431081, Eppendorf, Germany) using a 3·5 mL transfer pipette (order number 86.1171.002, Sarstedt AG, Nümbrecht, Germany) and immediately frozen and shipped on dry ice the day of sample collection. CSF samples were stored at the central lab at −70°C and thawed at room temperature for 1 hour before measurement. Baseline and follow-up samples of the same participant were measured in one batch using single reagent lots at the end of the study. Effects of differences of sample time in the freezer were not evaluated.

CSF biomarkers included those associated with amyloid-beta (Aβ) pathophysiology (oligomeric Aβ [oAβ], total Aβ40 and total Aβ42), tau pathology (phosphorylated tau at threonine 181 [pTau181] and total tau [tTau]), neuronal degeneration (neurofilament light [NfL] and calcium-binding protein B [S100B]), and synaptic dysfunction (neurogranin); microglial activation (soluble triggering receptor expressed on myeloid cells 2 [sTREM2]), astroglial activation (glial fibrillary acidic protein [GFAP] and chitinase-3-like protein 1 [YKL-40]), and other inflammatory processes (interleukin-6 [IL-6]); and α-synuclein (α-syn) pathology. Plasma biomarkers included those associated with Aβ plaque deposition, Aβ-mediated tau pathophysiology and tau tangle deposition (pTau181, pTau217), neuronal injury and degeneration (NfL), and neuroinflammation (GFAP, sTREM2, and YKL-40).

CSF total Aβ42, tTau, and pTau181 were measured with Roche Diagnostics Elecsys® *in vitro* diagnostic medical device immunoassays and performed as described previously.[4,5] CSF biomarkers, including total Aβ40, NfL, S100B, neurogranin, sTREM2, GFAP, YKL-40, α-syn, and IL-6, were measured.

CSF oAβ analysis

CSF oAβ was measured using an oAβ-preferring immunoassay on the SMCxPRO® (EMD Millipore) platform. The SMCxPRO (EMD Millipore) was based on single-molecule-counting technology that typically allows a 20-fold to 100-fold increase in sensitivity compared with traditional immunodetection systems.[6] Biotinylated capture monoclonal antibody (mAb) 71A1 was conjugated to streptavidin magnetic particles (MPs) (Dynabeads MyOne, Thermo Fisher Scientific) at a ratio of 12·5 μg biotinylated 71A1 antibody per milligram of MPs using a kit from EMD Millipore. MPs with bound capture antibody were diluted to 50 μg/mL in Aβ-Oligomer Assay Buffer (Tris buffer; 50 mM Tris, 150 mM NaCl, pH 7·6 with 1% Triton X-100, 0·0005% [w/v] d-des-thio-biotin and 0·1% bovine serum albumin); 50 μL of this suspension was then added to 150 μL of sample, standard, or blank, and incubated at 600 rpm on a shaking incubator at 25°C for 2 hours. MPs were isolated using a magnet, and unbound material was removed by washing with 1x SMC wash buffer (EMD Millipore) using a BioTek plate washer. Fluorescently labelled (Alexa-647 dye) anti-Aβ detection antibody 3D6 (20 μL, 200 ng/mL) was added to each well. MPs bearing the antibody–oligomer Aβ sandwich were then incubated with agitation using a Jitterbug shaker (Boekel, Feasterville, PA, USA) for 1 hour at 25°C. Unbound detection reagent was removed by washing (four times) with the SMC wash buffer. The wash buffer was removed by aspiration, and the fluorescently labelled 3D6 detection antibody was released from the beads by shaking in Elution Buffer B (EMD Millipore, Cat # 02-0297-00,

11·5 µL/well) for 10 minutes at 25°C. Eleven microlitres of each eluate was then transferred to the wells of a clean 96-well plate containing the Neutralization Buffer D (EMD Millipore, Cat # 02-0368-00, 11 µL/well). The neutralised sample (20 µL/well) was transferred to a black 386-well read plate (Aurora) and read by the SMCxPRO instrument. When fluorescently labelled antibodies are excited by a 642 nm laser and passed through the interrogation space, they emit light that is measured using a confocal microscope lens and a photon detector. The output from the detector is a train of pulses, with each pulse representing one photon that was detected. The lower limit of reliable quantification was defined as the lowest back interpolated standard that provides a signal two-fold the background with a percentage of recovery calculated between 80% and 100% and coefficient of variance ≤20%. Mixed models for repeated measures adjusting for age, education, apolipoprotein E (*APOE*) genotype (*APOE* ε4 carrier *vs* non-carrier), baseline Clinical Dementia Rating (zero *vs* non-zero), baseline value, treatment group, visit, treatment-by-visit interaction, and baseline-by-visit interaction were used to estimate mean changes from baseline in each treatment arm at Weeks 104 and 260.

Blood biomarker collection and analysis

Blood samples, including plasma, were collected from all participants at baseline and annual follow-up visits. Approximately 6 mL whole blood was collected into a 6 mL K2 EDTA primary collection tube (order number 367863, BD, Allschwil, Switzerland). The primary collection tube was inverted gently 8–10 times by hand to ensure the anticoagulant was mixed well with the blood. Within 60 minutes of blood collection, the blood was centrifuged at 1500 g for 15 minutes at room temperature, and 0·5 mL of supernatant was dispensed into 4 mL PP cryovials (order number 430490, Corning, Root, Switzerland) and immediately frozen and stored at –20°C for screening and baseline and at −70°C for all subsequent samples. Samples were shipped on dry ice to the central lab for testing and long-term storage was kept at −70°C. All baseline and follow-up samples of the same participant were analysed in one batch using a single reagent lot at the end of the study.

Plasma biomarkers, including total Aβ40, total Aβ42, pTau181, first-generation pTau217, NfL, GFAP, sTREM2, and YKL-40, were also measured with the Roche Diagnostics Elecsys® *in vitro* diagnostic medical device using Elecsys® NeuroToolKit robust prototype assays, as described previously.[7]

A second-generation plasma pTau217 assay, run on Roche's fully automated Elecsys® immunoassay platform, was performed *post hoc* and used for the main pTau217 analyses in this report. The assay, which uses a different pTau217 capture antibody and an N-terminal domain tau detector antibody, was recently shown to have a 0·94 (95% confidence interval 0·92, 0·96) receiver operating characteristic area under the curve for the presence or absence of at least moderately frequent neuritic Aβ plaques using positron emission tomography (PET) in cognitively unimpaired older adults without known ADAD mutations.[8]

PET image acquisition and analysis

PET images were acquired on a Siemens/CTI Biograph™ mCT and reconstructed using a standard iterative algorithm corrected for radiation attenuation and scatter. Florbetapir PET images were performed with a 10 mCi intravenous (IV) bolus florbetapir injection, 50-minute radiotracer uptake period, and 20-minute dynamic emission scan (4 × 5-minute frames). For baseline assessments, mean cortical florbetapir standardised uptake value ratios (SUVRs) were calculated using inferior medial frontal gyrus, superior parietal cortex, lateral temporal cortex, posterior/anterior cingulate cortex, and precuneus regions of interest (ROIs) and a whole cerebellar reference ROI. Baseline mean cortical florbetapir SUVRs were transformed into Centiloids (CL), a standardised measurement used to characterise and compare PET measurements of neuritic plaque burden using different amyloid PET ligands, as previously described.[9,10]

Amyloid PET positivity was defined as SUVR ≥1·10 (24·3 CL), corresponding to evidence of at least moderately frequent neuritic Aβ plaques in an antemortem–postmortem neuropathological validation study.[11] The presence of any measurable neuritic plaques was defined *post hoc* as SUVR >1·07, based on the maximum value of the entire young-adult and middle-aged mutation non-carrier group (**Fig. 2A**). For longitudinal assessments, mean cortical SUVR changes were calculated using the same cerebral ROI, but a subcortical white matter (corpus callosum and centrum semiovale) reference ROI was used based on its established ability to detect longitudinal changes with improved precision and statistical power.[12,13] Participants were scanned at baseline, Year 2, Year 5, and study completion (if more than 6 months had elapsed since the previous florbetapir PET scan).

Fluorodeoxyglucose (FDG) PET scans were performed in the resting state after at least a 4-hour fast using a 5 mCi IV bolus FDG injection, 30-minute radiotracer uptake period, and 30-minute dynamic emission scan (6 × 5-minute frames). SUVRs in an empirically predefined statistical ROI were calculated using bilateral posterior cingulate, medial and lateral parietal, frontal and temporal regions that decline preferentially, and white matter, cerebellum and somatosensory cortex regions that are preferentially spared in the early clinical stages of AD.[14] Participants were scanned at baseline, Week 12, Year 2, Year 5, and study completion (only if more than 6 months had elapsed since the previous FDG PET scan).

Genentech Tau Probe 1 (GTP1) PET images of tau tangle burden were performed using 7 mCi IV bolus GTP1 injection, 60-minute radiotracer uptake period, and 30-minute dynamic emission scan (6 × 5-minute frames). Mean SUVR values were calculated for the entorhinal cortex using an inferior cerebellum reference region, defined based on the joint analysis of a cerebellum atlas in the Montreal Neurological Institute (MNI) space and the FreeSurfer-defined cerebellar cortex.[15] Unlike the florbetapir PET and FDG PET scans required as part of the main study, tau PET scans were part of an optional sub-study initiated when GTP1 was approved for use by local health authorities and after the start of the main study, preventing the acquisition of pre-treatment baseline scans. First scans were acquired as soon as eligible individuals consented to participate in the sub-study, therefore the timing of the scans relative to each participant's start of treatment was variable, ranging from Week 130 to Week 224, depending on the date of entrance into the main study. Second scans were acquired as close as possible to Year 5 (as late as possible in the Week 248–260 window), regardless of the timing of the first scan so that, although the relative separations between the first and second scans varied, all participants would have approximately the same treatment duration. A third optional scan was acquired at study completion if more than 6 months had elapsed since the previous GTP1 PET scan.

All PET analyses were performed using a hybrid pipeline combining the PET Unified Pipeline[16,17] and Statistical Parametric Mapping (SPM; https://www.fil.ion.ucl.ac.uk/spm). Image frames were corrected for motion, summed, and co-registered with baseline magnetic resonance imaging (MRI) exams, and transformed into the MNI space using SPM. Mean SUVRs were computed in either native or template space, depending on the radiotracer method.

MRI acquisition and analysis

MRI exams were performed on a 3T Siemens Tim Trio at Hospital Pablo Tobón Uribe and consisted of several sequences for safety and structural and functional assessments. For volumetric measurements, a 3D T1 magnetization-prepared rapid acquisition gradient echo (MP RAGE) was used (repetition time 1·8 seconds, echo time 2·5 milliseconds, inversion time 900 milliseconds, field of view 24 cm, 1·25 × 1·25 mm^2 in-plane resolution, 1·2-mm slices). MRIs were acquired at baseline, Weeks 12, 24, 36, and 52, and then every 6 months until study completion. In participants who were switched from subcutaneous to IV treatment, an additional MRI was performed 3 months after the first IV dose and every 6 months thereafter.

Bilateral hippocampal and brain lateral ventricle volumes at each visit were calculated using FreeSurfer[18] to compute longitudinal percentage changes from baseline at each follow-up visit. Baseline whole brain volumes were measured using the same FreeSurfer methods, while longitudinal percentage changes were measured using an iterative principal component analysis (IPCA) applied to pairs of baseline and follow-up images.[19] For each participant, the inherent bias in the IPCA follow-up-to-baseline whole brain volume changes was corrected by incorporating the average of 12-week-to-baseline and baseline-to-12-week whole brain volume changes.

Supplementary tables

Supplementary Table 1: Schedule of biomarker assessments in the API ADAD study

	**Methodology**	**Schedule of assessments**
Amyloid PET	Florbetapir in SUVR and CL<br>mean cortical-to-cerebellar SUVRs for baseline comparisons;<br>mean cortical-white matter SUVRs for longitudinal comparisons	BL, 24 months, 60 months, and end of study
Tau PET	GTP1<br>entorhinal cortex SUVRs	Optional, 32–52 months, 45–67 months, and end of study
FDG PET	SUVRs (CMRgl decline) in an AD-related statistical ROI	BL, 3, 24, and 60 months, as well as end of study
vMRI	Whole brain<br>Bilateral hippocampus<br>Brain lateral ventricles	BL, 3, 6, 9, and 12 months, as well as every 6 months, and end of study
Plasma	Roche Elecsys®<br>Total Aβ40, total Aβ42, pTau181, pTau217, & NfL (A/T/N);<br>GFAP, sTREM2, YKL-40 (neuroinflammation)	BL, annual, and end of study
CSF	Roche Elecsys®<br>Total Aβ40, total Aβ42, pTau181, tTau, NfL, & neurogranin (A/T/N);<br>GFAP, sTREM2, YKL-40, IL-6, S100B (neuroinflammation);<br>α-synuclein<br>Selkoe lab assay[6] for oAβ	Optional, BL, 24 months, 60 months, and end of study

Aβ=amyloid-beta. AD=Alzheimer's disease. ADAD=autosomal-dominant Alzheimer's disease. API=Alzheimer's Prevention Initiative. A/T/N=amyloid/tau/biomarkers of neurodegeneration. BL=baseline. CL=Centiloids. CMRgl=cerebral metabolic rate for glucose. CSF=cerebrospinal fluid. FDG=fluorodeoxyglucose. GFAP=glial fibrillary acidic protein. GTP1=Genentech Tau Probe 1. IL-6=interleukin-6. NfL=neurofilament light. oAβ=oligomeric amyloid-beta. PET=positron emission tomography. pTau181/217=phosphorylated tau at threonine 181/217. ROI=region of interest. S100B=S100 calcium-binding protein B. sTREM2=soluble triggering receptor expressed on myeloid cells 2. SUVR=standardised uptake value ratio. tTau=total tau. vMRI=volumetric magnetic resonance imaging. YKL-40=chitinase-3-like protein 1.

Supplementary Table 2: **Baseline biomarker levels in crenezumab- vs placebo-treated mutation carriers and placebo-treated non-carriers**

	**Crenezumab–carrier (n = 85)**	**Placebo–carrier (n = 84)**	**Placebo–non-carrier (n = 83)**	**Nominal p value (carriers *vs* non-carriers, adjusted for age)**
**PET**				
Amyloid PET positive participants: SUVR > 1·1 (24·3 CL), %	61·2	48·8	0	<0·0001
Mean amyloid PET, SUVR (SD)*	1·15 (0·15)	1·11 (0·12)	0·96 (0·04)	<0·0001
Mean amyloid PET, CL (SD)	33·11 (27·22)	25·36 (21·43)	–2·18 (8·23)	<0·0001
Mean FDG PET, SUVR (SD)	1·29 (0·05)	1·29 (0·04)	1·30 (0·04)	<0·0001
**vMRI, mean (SD) Participants, n**				
vMRI whole brain, mL	1187651·833 (116137·386); n=84	1150080·083 (89912·350); n=84	1144841·422 (112438·683); n=83	0·59415
vMRI bilateral hippocampus, mL	8374·927 (849·131); n=85	8243·615 (726·938); n=84	8241·700 (763·589); n=83	0·58835
vMRI brain lateral ventricles, mL	13386·786 (6119·661); n=85	13387·295 (5641·237); n=84	14138·036 (6746·178); n=83	0·32927
**CSF, mean (SD) Participants, n**				
Aβ42, pg/mL[†]	890·94 (328·74); n=48	850·91 (317·84); n=41	1402·61 (326·06); n=37	<0·0001
Aβ40, pg/mL[†]	13628·13 (3714·62); n=48	13616·19 (3608·81); n=42	14799·19 (4281·55); n=37	0·4597
oAβ, pg/mL	3540·44 (1943·49); n=42	3718·64 (2085·57); n=38	3886·28 (1898·00); n=34	0·93738
pTau181, pg/mL[†]	20·43 (18·20); n=48	19·32 (11·62); n=42	11·44 (4·80); n=37	<0·0001
tTau, pg/mL[†]	197·91 (123·94); n=48	202·99 (93·44); n=42	138·49 (57·30); n=36	<0·0001
NfL (log_{10}), pg/mL[†]	1·71 (0·33); n=48	1·72 (0·20); n=42	1·66 (0·15); n=37	0·0006
Neurogranin, pg/mL[†]	1051·19 (622·25); n=48	1110·30 (658·52); n=42	804·66 (350·03); n=37	0·00515
αSyn, pg/mL[†]	324·49 (247·35); n=48	464·03 (690·28); n=42	251·59 (152·13); n=37	0·0149
GFAP, pg/mL[†]	4674·17 (2842·91); n=48	4762·38 (1946·85); n=42	4073·24 (1979·91); n=37	<0·0001
sTREM2, ng/mL[†]	7·52 (2·65); n=48	7·67 (2·19); n=42	7·44 (2·11); n=37	0·0508
YKL-40, ng/L[†]	87882·71 (51123·54); n=48	97458·81 (39429·08); n=42	90774·32 (31519·41); n=37	0·0053
IL-6 (log_{10}), ng/L[†]	0·57 (0·10); n=48	0·56 (0·22); n=42	0·55 (0·12); n=37	0·8808
S100B, µg/L[†]	0·70 (0·18); n=48	0·77 (0·28); n=42	0·68 (0·28); n=37	0·1647
**Plasma, mean (SD) Participants, n**				
Aβ42, pg/mL[†]	27·40 (5·58); n=84	25·93 (5·86); n=84	24·78 (14·32); n=82	0·2342
Aβ40, pg/mL[†]	285·19 (52·46); n=84	276·04 (68·42); n=84	326·84 (189·40); n=83	0·0069
pTau181 (log_{10}), pg/mL[†]	–0·01 (0·19); n=84	–0·01 (0·22); n=84	–0·25 (0·11); n=83	<0·0001
First-generation pTau217 (log_{10}), pg/mL[†]	–0·60 (0·26); n=79	–0·57 (0·30); n=77	–0·85 (0·25); n=79	<0·0001
Second-generation pTau217 (log_{10}), pg/mL[†]	–0·62 (0·288); n=84	–0·466 (0·331); n=77	–0·918 (0·164); n=79	<0·0001

NfL ($\log_{10}$), pg/mL†	0·09 (0·21); n=84	0·13 (0·17); n=84	0·05 (0·19); n=83	<0·0001
GFAP ($\log_{10}$), pg/mL†	1·83 (0·30); n=84	1·86 (0·33); n=84	1·54 (0·23); n=83	<0·0001
sTREM2 ($\log_{10}$), ng/mL†	0·66 (0·18); n=84	0·63 (0·18); n=84	0·69 (0·22); n=83	0·9903
YKL-40 ($\log_{10}$), µg/L†	1·47 (0·25); n=84	1·43 (0·19); n=84	1·54 (0·28); n=83	0·5906

*Whole cerebellum is used as the reference region; threshold >1.1 is defined as positive. Baseline tau PET measurements are not available since GTP1 PET was introduced later in the trial. †A subset of participants took part in the optional CSF substudy.

αSyn=α-synuclein. Aβ=amyloid-beta. CL=Centiloids. CSF=cerebrospinal fluid. FDG=fluorodeoxyglucose. GFAP=glial fibrillary acidic protein. GTP1=Genentech Tau Probe 1. IL-6=interleukin-6. NfL=neurofilament light. oAβ=oligomeric amyloid-beta. PET=positron emission tomography. pTau181/217=phosphorylated tau at threonine 181/217. S100B=S100 calcium-binding protein B. SD=standard deviation. sTREM2=soluble triggering receptor expressed on myeloid cells 2. SUVR=standardised uptake value ratio. tTau=total tau. vMRI=volumetric magnetic resonance imaging. YKL-40=chitinase-3-like protein 1.

Supplementary Table 3: **Summary of all reported AEs in mutation carriers by system organ class**

	**Crenezumab (n=84)**	**Placebo (n=84)**
**Participants with any AE**	**84 (100%)**	**84 (100%)**
**NCI CTCAE grade ≥3**	**18 (21·4%)**	**23 (27·4%)**
**Any grade AEs by system organ class**		
Infections and infestations		
Overall	84 (100.0%)	81 (96.4%)
Nasopharyngitis	81 (96.4%)	81 (96.4%)
Gastroenteritis	18 (21.4%)	24 (28.6%)
Urinary tract infection	17 (20.2%)	21 (25.0%)
Tonsillitis	16 (19.0%)	17 (20.2%)
Vaginal infection	15 (17.9%)	15 (17.9%)
Upper respiratory tract infection	10 (11.9%)	11 (13.1%)
Pharyngitis	9 (10.7%)	10 (11.9%)
Laryngitis	8 (9.5%)	11 (13.1%)
Oral herpes	8 (9.5%)	10 (11.9%)
Respiratory tract infection	9 (10.7%)	8 (9.5%)
Sinusitis	6 (7.1%)	9 (10.7%)
Viral infection	5 (6.0%)	10 (11.9%)
Cystitis	7 (8.3%)	6 (7.1%)
Gastrointestinal infection	5 (6.0%)	7 (8.3%)
Furuncle	4 (4.8%)	12 (14.3%)
Conjunctivitis	6 (7.1%)	6 (7.1%)
Bronchitis	5 (6.0%)	5 (6.0%)
Gastroenteritis viral	2 (2.4%)	7 (8.3%)
COVID-19	12 (14.3%)	2 (2.4%)
Bacterial vaginosis	3 (3.6%)	7 (8.3%)
Cellulitis	4 (4.8%)	2 (2.4%)
Rhinitis	5 (6.0%)	3 (3.6%)

Ear infection	3 (3.6%)	3 (3.6%)
Parasitic gastroenteritis	2 (2.4%)	2 (2.4%)
Vulvovaginal candidiasis	2 (2.4%)	2 (2.4%)
Hordeolum	3 (3.6%)	3 (3.6%)
Folliculitis	4 (4.8%)	1 (1.2%)
Onychomycosis	1 (1.2%)	5 (6.0%)
Suspected COVID-19	1 (1.2%)	0
Fungal skin infection	2 (2.4%)	2 (2.4%)
Herpes zoster	2 (2.4%)	2 (2.4%)
Otitis externa	3 (3.6%)	3 (3.6%)
Subcutaneous abscess	3 (3.6%)	1 (1.2%)
Otitis media	1 (1.2%)	0
Tooth abscess	1 (1.2%)	2 (2.4%)
Localised infection	2 (2.4%)	1 (1.2%)
Abscess limb	1 (1.2%)	2 (2.4%)
Oral candidiasis	1 (1.2%)	3 (3.6%)
Otitis media acute	1 (1.2%)	3 (3.6%)
Acute sinusitis	3 (3.6%)	0
Amoebic dysentery	0	3 (3.6%)
Body tinea	3 (3.6%)	0
Gingivitis	0	3 (3.6%)
Soft tissue infection	0	1 (1.2%)
Appendicitis	1 (1.2%)	1 (1.2%)
Cervicitis	1 (1.2%)	0
Infection parasitic	1 (1.2%)	1 (1.2%)
Perineal abscess	1 (1.2%)	1 (1.2%)
Paronychia	2 (2.4%)	1 (1.2%)
Abscess	0	2 (2.4%)
Enteritis infectious	0	1 (1.2%)
Pelvic inflammatory disease	0	2 (2.4%)

Pharyngotonsillitis	0	2 (2.4%)
Tinea versicolour	3 (3.6%)	0
Dengue fever	1 (1.2%)	1 (1.2%)
Infection	1 (1.2%)	0
Latent syphilis	1 (1.2%)	1 (1.2%)
Tinea infection	1 (1.2%)	1 (1.2%)
Tooth infection	1 (1.2%)	0
Erysipelas	0	1 (1.2%)
Fungal infection	0	1 (1.2%)
Gingival abscess	0	1 (1.2%)
Groin abscess	0	1 (1.2%)
HIV infection	0	1 (1.2%)
Helicobacter gastritis	0	1 (1.2%)
Papilloma viral infection	0	1 (1.2%)
Secondary syphilis	0	1 (1.2%)
Tonsillitis bacterial	0	1 (1.2%)
Abscess neck	1 (1.2%)	0
Ascariasis	1 (1.2%)	0
Bartholin's abscess	1 (1.2%)	0
Conjunctivitis bacterial	1 (1.2%)	0
Epididymitis	1 (1.2%)	0
Genital abscess	1 (1.2%)	0
Herpes simplex	1 (1.2%)	0
Lice infestation	1 (1.2%)	0
Orchitis	1 (1.2%)	0
Pilonidal disease	1 (1.2%)	0
Postoperative wound infection	1 (1.2%)	0
Pyelonephritis	1 (1.2%)	0
Syphilis genital	1 (1.2%)	0
Tinea pedis	1 (1.2%)	0

Urethritis	1 (1.2%)	0
Viral tonsillitis	1 (1.2%)	0
Viral upper respiratory tract infection	1 (1.2%)	0
Vulval abscess	1 (1.2%)	0
Vulvovaginitis	1 (1.2%)	0
Nervous system disorders		
Overall	66 (78.6%)	70 (83.3%)
Headache	53 (63.1%)	54 (64.3%)
Dizziness	27 (32.1%)	35 (41.7%)
Migraine	6 (7.1%)	12 (14.3%)
Paraesthesia	7 (8.3%)	4 (4.8%)
Head discomfort	5 (6.0%)	5 (6.0%)
Somnolence	1 (1.2%)	3 (3.6%)
Carpal tunnel syndrome	3 (3.6%)	2 (2.4%)
Migraine without aura	2 (2.4%)	0
Myoclonus	3 (3.6%)	3 (3.6%)
Tension headache	2 (2.4%)	2 (2.4%)
Cerebral microhaemorrhage	1 (1.2%)	2 (2.4%)
Hypersomnia	1 (1.2%)	2 (2.4%)
Trigeminal neuralgia	1 (1.2%)	2 (2.4%)
Amyloid related imaging abnormality–microhaemorrhages and haemosiderin deposits	3 (3.6%)	0
Burning sensation	1 (1.2%)	1 (1.2%)
Migraine with aura	1 (1.2%)	0
Tremor	1 (1.2%)	0
Epilepsy	2 (2.4%)	0
Autonomic nervous system imbalance	0	1 (1.2%)
Dizziness postural	0	1 (1.2%)
Hypoxic-ischaemic encephalopathy	0	1 (1.2%)
Intention tremor	0	1 (1.2%)
Petit mal epilepsy	0	1 (1.2%)

Speech disorder	0	1 (1.2%)
Cerebellar ischaemia	1 (1.2%)	0
Cervicobrachial syndrome	1 (1.2%)	0
Haemorrhage intracranial	1 (1.2%)	0
Hyperesthesia	1 (1.2%)	0
Hypoaesthesia	1 (1.2%)	0
Hyposmia	1 (1.2%)	0
Neuropathy peripheral	1 (1.2%)	0
Post-traumatic headache	1 (1.2%)	0
Radiculopathy	1 (1.2%)	0
Transient global amnesia	1 (1.2%)	0
Musculoskeletal and connective tissue disorders		
Overall	64 (76.2%)	64 (76.2%)
Back pain	37 (44.0%)	40 (47.6%)
Arthralgia	19 (22.6%)	20 (23.8%)
Neck pain	11 (13.1%)	19 (22.6%)
Pain in extremity	14 (16.7%)	17 (20.2%)
Myalgia	8 (9.5%)	12 (14.3%)
Costochondritis	7 (8.3%)	6 (7.1%)
Muscle spasms	4 (4.8%)	6 (7.1%)
Musculoskeletal chest pain	3 (3.6%)	3 (3.6%)
Tendonitis	4 (4.8%)	4 (4.8%)
Tendon disorder	4 (4.8%)	4 (4.8%)
Musculoskeletal pain	2 (2.4%)	2 (2.4%)
Torticollis	1 (1.2%)	2 (2.4%)
Exostosis	0	2 (2.4%)
Osteoarthritis	1 (1.2%)	1 (1.2%)
Rotator cuff syndrome	1 (1.2%)	0
Bone pain	0	1 (1.2%)
Sacroiliitis	0	1 (1.2%)

Synovial cyst	1 (1.2%)	1 (1.2%)
Muscle contracture	2 (2.4%)	1 (1.2%)
Osteochondritis	0	1 (1.2%)
Temporomandibular joint syndrome	0	2 (2.4%)
Musculoskeletal stiffness	1 (1.2%)	1 (1.2%)
Sacral pain	1 (1.2%)	0
Fibromyalgia	0	1 (1.2%)
Groin pain	0	1 (1.2%)
Muscle fatigue	0	1 (1.2%)
Tenosynovitis	0	1 (1.2%)
Tenosynovitis stenosing	0	1 (1.2%)
Bursitis	1 (1.2%)	0
Flank pain	1 (1.2%)	0
Lordosis	1 (1.2%)	0
Myofascial pain syndrome	1 (1.2%)	0
Myopathy endocrine	1 (1.2%)	0
Neck mass	1 (1.2%)	0
Pain in jaw	1 (1.2%)	0
Pubic pain	1 (1.2%)	0
Spinal pain	1 (1.2%)	0
Injury, poisoning and procedural complications		
Overall	58 (69.0%)	58 (69.0%)
Infusion related reaction	25 (29.8%)	24 (28.6%)
Limb injury	12 (14.3%)	11 (13.1%)
Procedural pain	8 (9.5%)	10 (11.9%)
Post lumbar puncture syndrome	9 (10.7%)	8 (9.5%)
Soft tissue injury	6 (7.1%)	10 (11.9%)
Joint injury	4 (4.8%)	7 (8.3%)
Face injury	4 (4.8%)	4 (4.8%)
Ligament sprain	4 (4.8%)	3 (3.6%)

Chest injury	3 (3.6%)	2 (2.4%)
Injury	4 (4.8%)	3 (3.6%)
Procedural headache	2 (2.4%)	3 (3.6%)
Thermal burn	1 (1.2%)	4 (4.8%)
Tooth fracture	4 (4.8%)	2 (2.4%)
Foreign body in eye	2 (2.4%)	1 (1.2%)
Head injury	2 (2.4%)	0
Burns second degree	3 (3.6%)	0
Foot fracture	3 (3.6%)	1 (1.2%)
Joint dislocation	0	3 (3.6%)
Back injury	1 (1.2%)	0
Contusion	1 (1.2%)	3 (3.6%)
Epicondylitis	4 (4.8%)	0
Skin laceration	0	3 (3.6%)
Craniocerebral injury	1 (1.2%)	2 (2.4%)
Skin injury	1 (1.2%)	0
Animal bite	1 (1.2%)	1 (1.2%)
Arthropod sting	1 (1.2%)	1 (1.2%)
Burns first degree	1 (1.2%)	1 (1.2%)
Eye injury	1 (1.2%)	0
Injury of conjunctiva	1 (1.2%)	0
Nail injury	1 (1.2%)	0
Neck injury	1 (1.2%)	1 (1.2%)
Toxicity to various agents	1 (1.2%)	0
Animal scratch	0	1 (1.2%)
Chemical poisoning	0	1 (1.2%)
Hand fracture	0	1 (1.2%)
Ligament injury	0	1 (1.2%)
Muscle injury	0	1 (1.2%)
Nasal injury	0	1 (1.2%)

Poisoning	0	1 (1.2%)
Skin abrasion	0	1 (1.2%)
Skin wound	0	1 (1.2%)
Abdominal injury	1 (1.2%)	0
Accidental overdose	1 (1.2%)	0
Ankle fracture	1 (1.2%)	0
Arthropod bite	1 (1.2%)	0
Chemical burns of eye	1 (1.2%)	0
Eye contusion	1 (1.2%)	0
Fibula fracture	1 (1.2%)	0
Multiple injuries	1 (1.2%)	0
Muscle rupture	1 (1.2%)	0
Muscle strain	1 (1.2%)	0
Post procedural swelling	1 (1.2%)	0
Procedural dizziness	1 (1.2%)	0
Spinal column injury	1 (1.2%)	0
Sternal fracture	1 (1.2%)	0
Tendon rupture	1 (1.2%)	0
Wound	1 (1.2%)	0
Wound dehiscence	1 (1.2%)	0
Gastrointestinal disorders		
Overall	54 (64.3%)	59 (70.2%)
Abdominal pain	21 (25.0%)	16 (19.0%)
Gastritis	19 (22.6%)	22 (26.2%)
Diarrhoea	19 (22.6%)	14 (16.7%)
Toothache	7 (8.3%)	12 (14.3%)
Gastrooesophageal reflux disease	3 (3.6%)	7 (8.3%)
Irritable bowel syndrome	3 (3.6%)	3 (3.6%)
Vomiting	4 (4.8%)	3 (3.6%)
Haemorrhoids	2 (2.4%)	1 (1.2%)

Constipation	1 (1.2%)	3 (3.6%)
Dyspepsia	2 (2.4%)	2 (2.4%)
Abdominal pain lower	2 (2.4%)	2 (2.4%)
Abdominal pain upper	2 (2.4%)	2 (2.4%)
Dysphagia	2 (2.4%)	2 (2.4%)
Nausea	1 (1.2%)	0
Chronic gastritis	2 (2.4%)	2 (2.4%)
Inguinal hernia	2 (2.4%)	1 (1.2%)
Umbilical hernia	1 (1.2%)	0
Abdominal hernia	0	1 (1.2%)
Stomatitis	0	2 (2.4%)
Food poisoning	1 (1.2%)	0
Odynophagia	1 (1.2%)	1 (1.2%)
Oesophagitis	1 (1.2%)	1 (1.2%)
Periodontal disease	1 (1.2%)	1 (1.2%)
Rectal haemorrhage	1 (1.2%)	1 (1.2%)
Abdominal incarcerated hernia	1 (1.2%)	0
Anal pruritus	0	1 (1.2%)
Aphthous ulcer	0	1 (1.2%)
Cheilitis	0	1 (1.2%)
Duodenal ulcer	0	1 (1.2%)
Duodenitis	0	1 (1.2%)
Erosive duodenitis	0	1 (1.2%)
Haematochezia	0	1 (1.2%)
Lip dry	0	1 (1.2%)
Mouth cyst	0	1 (1.2%)
Flatulence	1 (1.2%)	0
Hiatus hernia	1 (1.2%)	0
Stomach mass	1 (1.2%)	0
Psychiatric disorders		

Overall	41 (48.8%)	43 (51.2%)
Insomnia	13 (15.5%)	18 (21.4%)
Depression	16 (19.0%)	17 (20.2%)
Anxiety disorder	10 (11.9%)	9 (10.7%)
Middle insomnia	4 (4.8%)	7 (8.3%)
Initial insomnia	4 (4.8%)	4 (4.8%)
Mixed anxiety and depressive disorder	5 (6.0%)	3 (3.6%)
Anxiety	4 (4.8%)	5 (6.0%)
Adjustment disorder	5 (6.0%)	5 (6.0%)
Irritability	5 (6.0%)	5 (6.0%)
Libido decreased	3 (3.6%)	3 (3.6%)
Panic attack	3 (3.6%)	2 (2.4%)
Grief reaction	1 (1.2%)	2 (2.4%)
Claustrophobia	2 (2.4%)	1 (1.2%)
Suicide attempt	2 (2.4%)	1 (1.2%)
Panic disorder	1 (1.2%)	3 (3.6%)
Major depression	1 (1.2%)	1 (1.2%)
Stress	1 (1.2%)	1 (1.2%)
Agoraphobia	0	1 (1.2%)
Alcohol use disorder	0	1 (1.2%)
Delusion	0	1 (1.2%)
Delusional disorder, unspecified type	0	1 (1.2%)
Factitious disorder	0	1 (1.2%)
Persistent depressive disorder	0	1 (1.2%)
Personality change	0	1 (1.2%)
Tobacco abuse	0	1 (1.2%)
Adjustment disorder with depressed mood	1 (1.2%)	0
Alcohol abuse	1 (1.2%)	0
Bipolar disorder	1 (1.2%)	0
Deja vu	1 (1.2%)	0

Delirium	1 (1.2%)	0
Dependent personality disorder	1 (1.2%)	0
Drug abuse	1 (1.2%)	0
Hallucination, visual	1 (1.2%)	0
Skin and subcutaneous tissue disorders		
Overall	38 (45.2%)	41 (48.8%)
Urticaria	19 (22.6%)	10 (11.9%)
Dermatitis	5 (6.0%)	5 (6.0%)
Dermatitis allergic	4 (4.8%)	4 (4.8%)
Alopecia	5 (6.0%)	5 (6.0%)
Dermatitis contact	5 (6.0%)	2 (2.4%)
Pruritus	4 (4.8%)	3 (3.6%)
Rash	4 (4.8%)	3 (3.6%)
Acne	1 (1.2%)	2 (2.4%)
Ecchymosis	1 (1.2%)	3 (3.6%)
Hand dermatitis	1 (1.2%)	2 (2.4%)
Cellulite	1 (1.2%)	2 (2.4%)
Umbilical erythema	1 (1.2%)	2 (2.4%)
Chloasma	2 (2.4%)	1 (1.2%)
Erythema	2 (2.4%)	2 (2.4%)
Seborrhoeic dermatitis	2 (2.4%)	0
Dyshidrotic eczema	0	1 (1.2%)
Dermatitis atopic	1 (1.2%)	0
Macule	0	1 (1.2%)
Psoriasis	0	1 (1.2%)
Skin exfoliation	0	1 (1.2%)
Skin mass	0	1 (1.2%)
Urticaria chronic	1 (1.2%)	1 (1.2%)
Actinic keratosis	1 (1.2%)	0
Dry skin	0	1 (1.2%)

Eczema	0	1 (1.2%)
Eczema nummular	0	1 (1.2%)
Erythematotelangiectatic rosacea	0	1 (1.2%)
Guttate psoriasis	0	1 (1.2%)
Hypertrophic scar	0	1 (1.2%)
Ingrowing nail	0	1 (1.2%)
Miliaria	0	1 (1.2%)
Rash maculo-papular	0	1 (1.2%)
Rash papular	0	1 (1.2%)
Skin hypopigmentation	0	1 (1.2%)
Skin ulcer	0	1 (1.2%)
Umbilical haematoma	0	1 (1.2%)
Vitiligo	0	1 (1.2%)
Alopecia areata	1 (1.2%)	0
Intertrigo	1 (1.2%)	0
Pruritus allergic	1 (1.2%)	0
Skin hyperpigmentation	1 (1.2%)	0
General disorders and administration site conditions		
Overall	39 (46.4%)	40 (47.6%)
Injection site haemorrhage	10 (11.9%)	13 (15.5%)
Chest pain	9 (10.7%)	4 (4.8%)
Fatigue	3 (3.6%)	9 (10.7%)
Influenza like illness	5 (6.0%)	10 (11.9%)
Pyrexia	4 (4.8%)	5 (6.0%)
Injection site hypersensitivity	5 (6.0%)	5 (6.0%)
Malaise	3 (3.6%)	2 (2.4%)
Oedema peripheral	1 (1.2%)	4 (4.8%)
Discomfort	2 (2.4%)	3 (3.6%)
Application site erythema	0	2 (2.4%)
Application site haemorrhage	2 (2.4%)	1 (1.2%)

Injection site pruritus	5 (6.0%)	0
Facial pain	3 (3.6%)	0
Application site pain	0	2 (2.4%)
Asthenia	0	2 (2.4%)
Application site oedema	0	1 (1.2%)
Puncture site pain	0	2 (2.4%)
Vessel puncture site haemorrhage	0	2 (2.4%)
Injection site dermatitis	1 (1.2%)	0
Injection site pain	2 (2.4%)	0
Injection site rash	2 (2.4%)	0
Chest discomfort	0	1 (1.2%)
Cyst rupture	0	1 (1.2%)
Feeling cold	0	1 (1.2%)
Hangover	0	1 (1.2%)
Injection site erythema	0	1 (1.2%)
Injection site papule	0	1 (1.2%)
Pain	0	1 (1.2%)
Peripheral swelling	0	1 (1.2%)
Ill-defined disorder	1 (1.2%)	0
Injection site oedema	1 (1.2%)	0
Injection site urticaria	1 (1.2%)	0
Papillitis	1 (1.2%)	0
Vessel puncture site pain	1 (1.2%)	0
Reproductive system and breast disorders		
Overall	24 (28.6%)	34 (40.5%)
Polymenorrhoea	4 (4.8%)	7 (8.3%)
Amenorrhoea	4 (4.8%)	7 (8.3%)
Abnormal uterine bleeding	4 (4.8%)	9 (10.7%)
Heavy menstrual bleeding	2 (2.4%)	6 (7.1%)
Dysmenorrhoea	3 (3.6%)	3 (3.6%)

Pelvic pain	1 (1.2%)	5 (6.0%)
Breast pain	4 (4.8%)	5 (6.0%)
Oligomenorrhoea	2 (2.4%)	2 (2.4%)
Adenomyosis	1 (1.2%)	4 (4.8%)
Breast cyst	1 (1.2%)	2 (2.4%)
Ovarian cyst	1 (1.2%)	2 (2.4%)
Cervical dysplasia	1 (1.2%)	1 (1.2%)
Premenstrual syndrome	1 (1.2%)	2 (2.4%)
Uterine haemorrhage	1 (1.2%)	2 (2.4%)
Erectile dysfunction	2 (2.4%)	0
Endometriosis	1 (1.2%)	1 (1.2%)
Intermenstrual bleeding	1 (1.2%)	1 (1.2%)
Vaginal haemorrhage	0	2 (2.4%)
Menstrual disorder	0	1 (1.2%)
Coital bleeding	0	1 (1.2%)
Dyspareunia	0	1 (1.2%)
Endometrial hyperplasia	0	1 (1.2%)
Hydrosalpinx	0	1 (1.2%)
Vulvovaginal pruritus	0	1 (1.2%)
Balanoposthitis	1 (1.2%)	0
Hypomenorrhoea	1 (1.2%)	0
Pelvic congestion	1 (1.2%)	0
Uterine polyp	1 (1.2%)	0
Metabolism and nutrition disorders		
Overall	33 (39.3%)	19 (22.6%)
Hypertriglyceridaemia	21 (25.0%)	10 (11.9%)
Dyslipidaemia	6 (7.1%)	4 (4.8%)
Hyperglycaemia	2 (2.4%)	3 (3.6%)
Hypercholesterolaemia	3 (3.6%)	2 (2.4%)
Type 2 diabetes mellitus	2 (2.4%)	0

Diabetes mellitus	1 (1.2%)	1 (1.2%)
Hyperuricaemia	1 (1.2%)	0
Decreased appetite	3 (3.6%)	0
Glucose tolerance impaired	1 (1.2%)	0
Abnormal loss of weight	0	1 (1.2%)
Obesity	0	1 (1.2%)
Vitamin B12 deficiency	0	1 (1.2%)
Vitamin D deficiency	0	1 (1.2%)
Calcium metabolism disorder	1 (1.2%)	0
Hypercalcaemia	1 (1.2%)	0
Hyperkalaemia	1 (1.2%)	0
Respiratory, thoracic and mediastinal disorders		
Overall	15 (17.9%)	17 (20.2%)
Cough	8 (9.5%)	8 (9.5%)
Rhinitis allergic	1 (1.2%)	0
Asthma	2 (2.4%)	2 (2.4%)
Dysphonia	3 (3.6%)	2 (2.4%)
Epistaxis	2 (2.4%)	1 (1.2%)
Oropharyngeal pain	2 (2.4%)	0
Dyspnoea	2 (2.4%)	0
Bronchospasm	0	1 (1.2%)
Laryngospasm	0	1 (1.2%)
Obstructive sleep apnoea syndrome	0	1 (1.2%)
Status asthmaticus	0	1 (1.2%)
Allergic cough	1 (1.2%)	0
Pulmonary embolism	1 (1.2%)	0
Pulmonary infarction	1 (1.2%)	0
Vascular disorders		
Overall	15 (17.9%)	20 (23.8%)
Essential hypertension	4 (4.8%)	10 (11.9%)

Hypertension	5 (6.0%)	5 (6.0%)
Peripheral venous disease	0	1 (1.2%)
Hot flush	2 (2.4%)	2 (2.4%)
Varicose vein	1 (1.2%)	1 (1.2%)
Haematoma	0	1 (1.2%)
Hypertensive crisis	0	1 (1.2%)
Phlebitis	0	1 (1.2%)
Lymphoedema	1 (1.2%)	0
Orthostatic hypotension	1 (1.2%)	0
Prehypertension	1 (1.2%)	0
Renal and urinary disorders		
Overall	19 (22.6%)	15 (17.9%)
Calculus urinary	9 (10.7%)	7 (8.3%)
Dysuria	2 (2.4%)	2 (2.4%)
Renal colic	2 (2.4%)	3 (3.6%)
Renal pain	2 (2.4%)	0
Stress urinary incontinence	2 (2.4%)	2 (2.4%)
Urinary incontinence	0	1 (1.2%)
Chronic kidney disease	1 (1.2%)	1 (1.2%)
Nocturia	1 (1.2%)	1 (1.2%)
Renal cyst	1 (1.2%)	0
Nephrolithiasis	2 (2.4%)	0
Haematuria	0	1 (1.2%)
Micturition disorder	0	1 (1.2%)
Micturition urgency	0	1 (1.2%)
Ureterolithiasis	0	1 (1.2%)
Choluria	1 (1.2%)	0
Lower urinary tract symptoms	1 (1.2%)	0
Renal failure	1 (1.2%)	0
Ear and labyrinth disorders		

Overall	15 (17.9%)	10 (11.9%)
Vertigo	7 (8.3%)	5 (6.0%)
Ear pain	3 (3.6%)	1 (1.2%)
Tinnitus	4 (4.8%)	0
Vertigo positional	1 (1.2%)	1 (1.2%)
Cerumen impaction	1 (1.2%)	0
Excessive cerumen production	1 (1.2%)	1 (1.2%)
Ear pruritus	0	1 (1.2%)
Hypoacusis	0	1 (1.2%)
Investigations		
Overall	16 (19.0%)	14 (16.7%)
Blood creatine phosphokinase increased	7 (8.3%)	4 (4.8%)
Weight increased	6 (7.1%)	3 (3.6%)
Alanine aminotransferase increased	2 (2.4%)	2 (2.4%)
Aspartate aminotransferase increased	1 (1.2%)	1 (1.2%)
Gamma-glutamyltransferase increased	1 (1.2%)	2 (2.4%)
Blood triglycerides increased	0	1 (1.2%)
Activated partial thromboplastin time prolonged	1 (1.2%)	0
Electrocardiogram QT prolonged	1 (1.2%)	2 (2.4%)
Glomerular filtration rate decreased	1 (1.2%)	2 (2.4%)
Prothrombin time prolonged	0	1 (1.2%)
Transaminases increased	0	1 (1.2%)
Coagulation time prolonged	1 (1.2%)	0
ECG signs of myocardial ischaemia	0	1 (1.2%)
Platelet count decreased	0	1 (1.2%)
Smear cervix abnormal	0	1 (1.2%)
Blood lactate dehydrogenase increased	1 (1.2%)	0
Blood pressure increased	1 (1.2%)	0
Cardiac murmur	1 (1.2%)	0
Weight decreased	1 (1.2%)	0

Eye disorders		
Overall	20 (23.8%)	10 (11.9%)
Conjunctival haemorrhage	2 (2.4%)	3 (3.6%)
Blepharitis	3 (3.6%)	1 (1.2%)
Pterygium	3 (3.6%)	1 (1.2%)
Photopsia	1 (1.2%)	0
Dry eye	2 (2.4%)	2 (2.4%)
Eye pain	2 (2.4%)	2 (2.4%)
Cataract	2 (2.4%)	1 (1.2%)
Conjunctivitis allergic	2 (2.4%)	0
Ulcerative keratitis	2 (2.4%)	1 (1.2%)
Visual acuity reduced	1 (1.2%)	0
Dacryostenosis acquired	0	1 (1.2%)
Eye allergy	0	1 (1.2%)
Keratitis	0	1 (1.2%)
Punctate keratitis	0	1 (1.2%)
Chalazion	1 (1.2%)	1 (1.2%)
Eye pruritus	1 (1.2%)	0
Myopia	1 (1.2%)	0
Photophobia	1 (1.2%)	0
Vitreous detachment	1 (1.2%)	0
Xerophthalmia	1 (1.2%)	0
Blood and lymphatic system disorders		
Overall	13 (15.5%)	9 (10.7%)
Anaemia	7 (8.3%)	5 (6.0%)
Lymphadenopathy	4 (4.8%)	5 (6.0%)
Neutropenia	2 (2.4%)	1 (1.2%)
Leukopenia	2 (2.4%)	0
Lymphadenitis	2 (2.4%)	1 (1.2%)
Iron deficiency anaemia	1 (1.2%)	0

Leukocytosis	1 (1.2%)	1 (1.2%)
Abnormal clotting factor	1 (1.2%)	0
Neutrophilia	1 (1.2%)	0
Neoplasms benign, malignant and unspecified (including cysts and polyps)		
Overall	4 (4.8%)	11 (13.1%)
Uterine leiomyoma	1 (1.2%)	4 (4.8%)
Leiomyoma	0	1 (1.2%)
Skin papilloma	0	1 (1.2%)
Seborrhoeic keratosis	2 (2.4%)	1 (1.2%)
Blepharal papilloma	0	1 (1.2%)
Bone neoplasm	0	1 (1.2%)
Hair follicle tumour benign	0	1 (1.2%)
Lipoma	0	1 (1.2%)
Ovarian neoplasm	0	1 (1.2%)
Haemangioma	1 (1.2%)	0
Parathyroid tumour benign	1 (1.2%)	0
Cardiac disorders		
Overall	8 (9.5%)	11 (13.1%)
Tachycardia	2 (2.4%)	4 (4.8%)
Palpitations	1 (1.2%)	2 (2.4%)
Cardiac failure	0	2 (2.4%)
Atrioventricular block	0	1 (1.2%)
Sinus bradycardia	2 (2.4%)	0
Aortic valve incompetence	0	1 (1.2%)
Atrioventricular block first degree	0	1 (1.2%)
Bradycardia	0	1 (1.2%)
Cardiac arrest	0	1 (1.2%)
Coronary artery disease	0	1 (1.2%)
Myocardial ischaemia	0	1 (1.2%)
Bundle branch block left	1 (1.2%)	0

Bundle branch block right	1 (1.2%)	0
Chronotropic incompetence	1 (1.2%)	0
Extrasystoles	1 (1.2%)	0
Sinus arrhythmia	1 (1.2%)	0
Endocrine disorders		
Overall	4 (4.8%)	8 (9.5%)
Hypothyroidism	1 (1.2%)	5 (6.0%)
Thyroid mass	1 (1.2%)	0
Autoimmune thyroiditis	0	1 (1.2%)
Pituitary haemorrhage	0	1 (1.2%)
Thyroid cyst	0	1 (1.2%)
Thyroiditis	0	1 (1.2%)
Goitre	1 (1.2%)	0
Hyperprolactinaemia	1 (1.2%)	0
Immune system disorders		
Overall	7 (8.3%)	5 (6.0%)
Hypersensitivity	3 (3.6%)	1 (1.2%)
Food allergy	0	2 (2.4%)
Allergy to arthropod bite	1 (1.2%)	1 (1.2%)
Immunisation reaction	2 (2.4%)	0
Dust allergy	0	1 (1.2%)
Rubber sensitivity	0	1 (1.2%)
Allergy to venom	1 (1.2%)	0
Drug hypersensitivity	1 (1.2%)	0
Hepatobiliary disorders		
Overall	6 (7.1%)	2 (2.4%)
Cholelithiasis	3 (3.6%)	1 (1.2%)
Hepatic steatosis	2 (2.4%)	1 (1.2%)
Gallbladder polyp	1 (1.2%)	1 (1.2%)
Autoimmune hepatitis	1 (1.2%)	0

Hepatitis toxic	1 (1.2%)	0
Pregnancy, puerperium and perinatal conditions		
Overall	6 (7.1%)	4 (4.8%)
Abortion spontaneous	2 (2.4%)	1 (1.2%)
Anembryonic gestation	0	1 (1.2%)
Hyperemesis gravidarum	0	1 (1.2%)
Ruptured ectopic pregnancy	0	1 (1.2%)
Foetal death	1 (1.2%)	0
Gestational diabetes	1 (1.2%)	0
Gestational hypertension	1 (1.2%)	0
Jaundice neonatal	1 (1.2%)	0
Pre-eclampsia	1 (1.2%)	0
Premature delivery	1 (1.2%)	0
Social circumstances		
Overall	1 (1.2%)	1 (1.2%)
Tobacco user	0	1 (1.2%)
Sexual abuse	1 (1.2%)	0
Congenital, familial and genetic disorders		
Congenital pyelocaliectasis	0	1 (1.2%)
Product issues		
Device breakage	0	1 (1.2%)
No coding available	1 (1.2%)	0

Supplementary Table 4: **Summary of clinical and biomarker treatment effects in mutation carriers at Week 260 (RCRM analyses)**

**Endpoint**	**Carrier (n)**	**Relative effect in crenezumab vs placebo treatment groups (%)**	**P value**	**CI lower**	**CI upper**
**Dual primary and key secondary clinical outcomes**					
API ADAD composite	168	22·9	0·43	–53·05	61·05
FCSRT-CI	168	19·9	0·16	–9·27	42·19
Time to MCI/dementia due to AD	168	20·8	-	–51·5	58·6
CDR–SB	168	8·8	-	–33·2	40·2
Time to non-zero in CDR-GS	150	8·1	-	–59	46·9
RBANS total score	168	43·8	-	–668·1	139·9
**Amyloid biomarker treatment effects**					
Aβ PET (florbetapir SUVR)*	168	3·6	-	–14·3	19·1
CSF total Aβ42	86	33·33	-	10·15	55·46
CSF total Aβ40	90	249·79	-	159·31	564·59
CSF oAβ	85	1127·10	-	442·55	18153·01
Plasma total Aβ42	168	1193·48	-	710·50	4617·51
Plasma total Aβ40	168	1096·30	-	688·10	2540·43
**Non-amyloid biomarker treatment effects**					
Tau PET (ERC GTP1 SUVR)	83	51·1	-	–41·8	102
FDG PET (sROI FDG SUVR)†	168	18·1	-	–14·4	42
vMRI whole brain	168	13·0	-	–35	51·6
vMRI bilateral hippocampus	168	9·4	-	–29·5	37·1
vMRI brain lateral ventricles	168	–2·5	-	–71·2	39·8
CSF pTau181	84	37·4	-	–53·9	87·8
CSF tTau	90	28·7	-	–136·3	88·2
CSF NfL (log_{10})	90	18·2	-	–33·4	56·8
CSF neurogranin	90	91·44	-	–665·06	942·13
CSF αSyn	90	542·08	-	27·87	5639·16
CSF GFAP	90	–15·03	-	–121·82	43·02
CSF sTREM2	90	4·83	-	–98·57	1313·56
CSF YKL-40	90	8·97	-	–41·42	40·33

CSF IL-6 ($\log_{10}$)	90	–28·30	-	–2078·02	399·60
CSF S100B	90	91·12	-	–380·47	242·41
Plasma pTau181 ($\log_{10}$)	168	6·16	-	–16·69	24·53
Plasma pTau217 ($\log_{10}$)	168	8·97	-	–21·02	31·68
Plasma NfL ($\log_{10}$)	168	10·53	-	–17·03	32·58
Plasma GFAP ($\log_{10}$)	168	17·68	-	1·97	31·75
Plasma sTREM2 ($\log_{10}$)	168	23·06	-	–22·33	51·07
Plasma YKL-40 ($\log_{10}$)	168	12·44	-	–70·19	56·59

Since the p values of the dual primary endpoints were larger than the allocated α levels (α=0·04 for API ADAD composite and α=0·01 for FCSRT-CI), the dual primary endpoints were not met. Thus, the key secondary endpoints specified in the α control could not be tested formally. Other than the dual primary endpoints, the reported CIs for all comparisons have not been adjusted for multiplicity and cannot be used to formally infer treatment effects. [*]Key secondary outcome. [†]FDG SUVR is computed as the ratio of a prespecified cortical ROI and a prespecified reference ROI that is preferentially associated with AD-related CMRgl declines. αSyn=α-synuclein. Aβ=amyloid-beta. AD=Alzheimer's disease. ADAD=autosomal-dominant Alzheimer's disease. API=Alzheimer's Prevention Initiative. API ADAD composite=Alzheimer's Prevention Initiative preclinical autosomal-dominant Alzheimer's disease composite test total score. BL=baseline. CDR-GS=Clinical Dementia Rating – Global Score. CDR SB=Clinical Dementia Rating – Sum of Boxes. CI=confidence interval. CMRgl= cerebral metabolic rate for glucose. CSF=cerebrospinal fluid. ERC=entorhinal cortex. FCSRT-CI=Free and Cued Selective Reminding Test – Cueing Index. FDG=fluorodeoxyglucose. GFAP=glial fibrillary acidic protein. GTP1=Genentech Tau Probe 1. IL-6=interleukin-6. MCI=mild cognitive impairment. NfL=neurofilament light. oAβ=oligomeric amyloid-beta. PET=positron emission tomography. pTau181/217=phosphorylated tau at threonine 181/217. RBANS=Repeatable Battery for the Assessment of Neuropsychological Status. RCRM=random coefficient regression model. ROI=region of interest. S100B=S100 calcium-binding protein B. sROI=statistical region of interest. sTREM2=soluble triggering receptor expressed on myeloid cells 2. SUVR=standardised uptake value ratio. tTau=total tau. vMRI=volumetric magnetic resonance imaging. YKL-40=chitinase-3-like protein 1.

Supplementary Table 5: **Summary of clinical and biomarker treatment effects in mutation carriers who received crenezumab compared with placebo at Week 260 (MMRM analyses)**

**Endpoint**	**Carriers (N)**	**Between-group difference in adjusted means (95% CI)**	**Relative reduction in adjusted means (%)**	**P value**
**Dual primary and key secondary clinical outcomes**				
API ADAD composite	157	2·0529 (–0·7805, 4·8864)	29·12	0·1553
FCSRT-CI	157	0·0662 (0·0096, 0·1228)	22·42	0·0219
**Amyloid biomarker treatment effects**				
CSF total Aβ42	60	106·5 (33·53, 179·53)	40·96	0·005
CSF total Aβ40	63	4069 (2532, 5604)	209·09	<0·001
Plasma total Aβ42	156	2781 (2530, 3032)	10,740·04	<0·0001
Plasma total Aβ40	156	35,779 (32,499, 39,058)	17,375·38	<0·0001
**Non-amyloid biomarker treatment effects**				
vMRI whole brain	157	0·0316 (–0·0377, 0·1009)	7·70	0·3711
vMRI bilateral hippocampus	157	0·3879 (–1·1676, 1·9433)	5·13	0·6244
vMRI brain lateral ventricles	157	–1·2296 (–9·3208, 6·8616)	3·50	0·7655
CSF pTau181	57	–3·222 (–8·3502, 1·9062)	50·93	0·2112
CSF tTau	62	–22·61 (–59·55, 14·32)	57·77	0·2244
CSF NfL (log_{10})	63	–0·0613 (–0·1689, 0·0462)	38·83	0·2584
CSF neurogranin	62	–85·31 (–252·9, 82·28)	70·85	0·3119

CSF αSyn	61	12·51 (–64·67, 89·69)	–20·30	0·7449
CSF GFAP	63	111·6 (–485·9, 709·1)	–8·07	0·7096
CSF sTREM2	63	0·0271 (–0·7514, 0·8057)	–3·25	0·9446
CSF IL-6 (log_{10})	63	0·0135 (–0·0567, 0·0836)	–242·56	0·7021
CSF S100B	62	–0·0359 (–0·1454, 0·0736)	131·07	0·5142
Plasma pTau181 (log_{10})	157	–0·0026 (–0·0441, 0·0389)	1·73	0·9023
Plasma pTau217 (log_{10})	149	–0·0107 (–0·0714, 0·0499)	6·21	0·7269
Plasma NfL (log_{10})	157	–0·0399 (–0·0926, 0·0127)	22·58	0·1358
Plasma GFAP (log_{10})	155	–0·0453 (–0·0937, 0·0031)	18·98	0·0665
Plasma sTREM2 (log_{10})	157	–0·0277 (–0·0588, 0·0134)	32·22	0·2165
Plasma YKL-40 (log_{10})	157	–0·0260 (–0·0762, 0·0243)	62·42	0·3089

Assessed in the modified intention-to-treat population. Since the p values of the dual primary endpoints were larger than the allocated α levels (α=0·04 for API ADAD composite and α=0·01 for FCSRT-CI), the dual primary endpoints were not met. Thus, the key secondary endpoints specified in the α control could not be tested formally. Other than the dual primary endpoints, the reported CIs for all comparisons have not been adjusted for multiplicity and cannot be used to formally infer treatment effects. αSyn=α-synuclein. Aβ=amyloid-beta. AD=Alzheimer's disease. ADAD=autosomal-dominant Alzheimer's disease. API=Alzheimer's Prevention Initiative. API ADAD composite=Alzheimer's Prevention Initiative preclinical autosomal-dominant Alzheimer's disease composite test total score. BL=baseline. CDR-GS=Clinical Dementia Rating – Global Score. CDR-SB=Clinical Dementia Rating – Sum of Boxes. CI=confidence interval. CSF=cerebrospinal fluid. FCSRT-CI=Free and Cued Selective Reminding Test – Cueing Index. FDG=fluorodeoxyglucose. GFAP=glial fibrillary acidic protein. GTP1=Genentech Tau Probe 1. IL-6=interleukin-6. MCI=mild cognitive impairment. MMRM=mixed models for repeated measures. NfL=neurofilament light. PET=positron emission tomography. pTau181/217=phosphorylated tau at threonine 181/217. RBANS=Repeatable Battery for the Assessment of Neuropsychological Status. S100B=S100 calcium-binding protein B. sROI=statistical region of interest. sTREM2=soluble triggering receptor expressed on myeloid cells 2. SUVR=standardised uptake value ratio. tTau=total tau. vMRI=volumetric magnetic resonance imaging. YKL-40=chitinase-3-like protein 1.

Supplementary figures

Figure S1: **Graph-based testing procedure for type I error control**

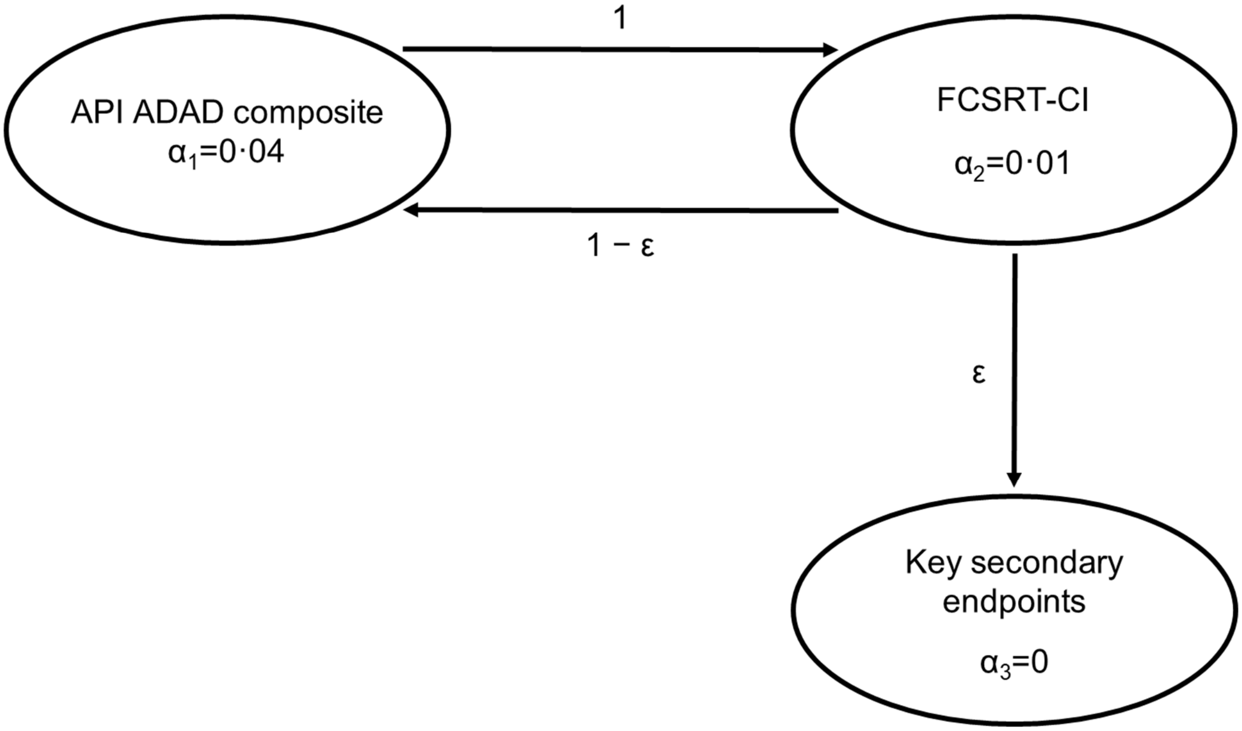

The arrows represent the direction of α propagation; the notation ε represents the potential to pass α from the FCSRT-CI to key secondary endpoints, but only if it is not necessary to pass α from the FCSRT-CI to the API ADAD composite. ADAD=autosomal-dominant Alzheimer's disease. API=Alzheimer's Prevention Initiative. API ADAD composite=Alzheimer's Prevention Initiative preclinical autosomal-dominant Alzheimer's disease composite test total score. FCSRT-CI=Free and Cued Selective Reminding Test – Cueing Index.

Figure S2: **Kaplan−Meier curve for time to MCI or dementia due to AD**

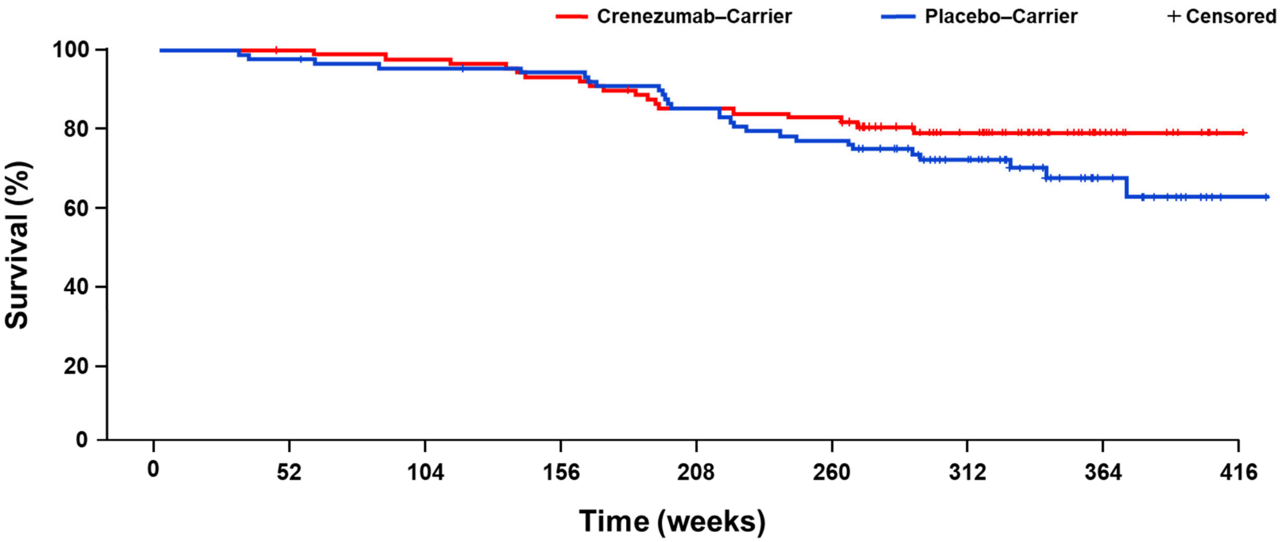

Number of participants at risk

**Crenezumab–Carrier**	84	83	81	77	69	67	44	16	1
**Placebo–Carrier**	84	82	79	77	69	62	44	14	1

AD=Alzheimer's disease. MCI=mild cognitive impairment.

Figure S3: **Total CSF Aβ40 on crenezumab vs placebo in mutation carriers**

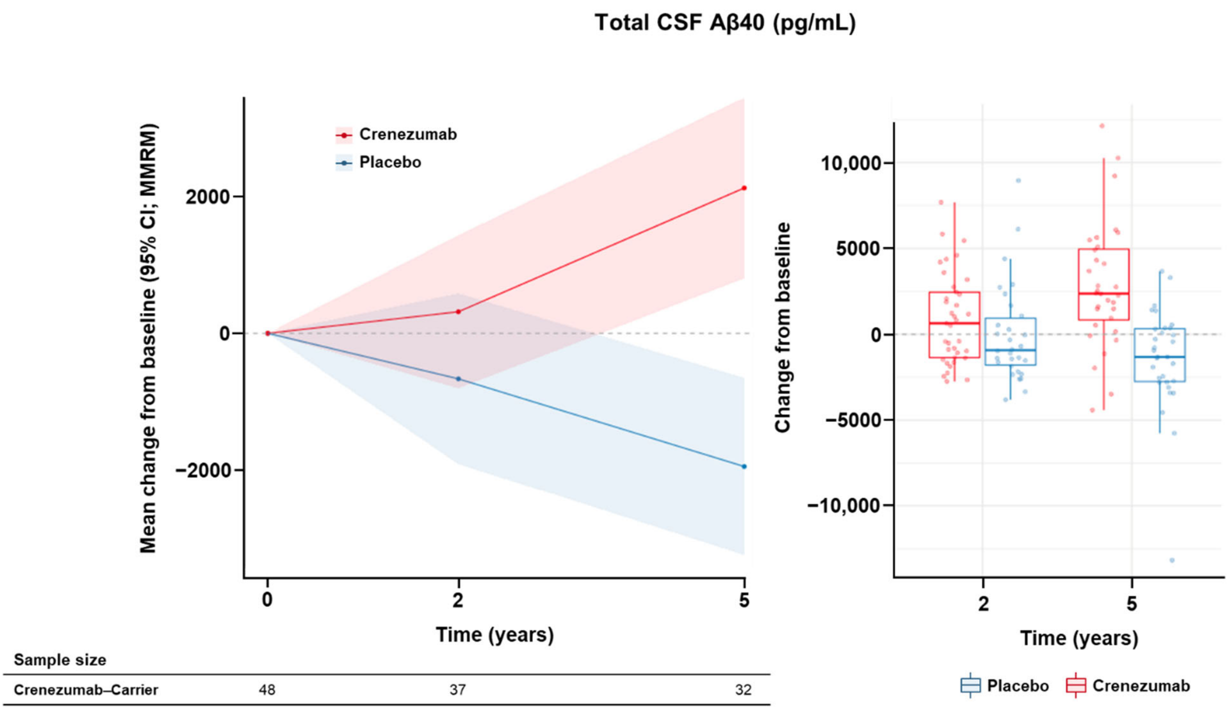

Sample size			
Crenezumab–Carrier	48	37	32
Placebo–Carrier	42	30	31

Aβ=amyloid-beta. CI=confidence interval. CSF=cerebrospinal fluid. MMRM=mixed models for repeated measures.

Figure S4: **Primary outcomes by baseline amyloid status**

Endpoint		Carrier	Relative effect	p value	95% CI
API ADAD composite	All	168	22·9%	0·43	
	Amyloid-positive	93	17·3%	0·50	
	Amyloid-negative	75	113·2%	0·21	
FCSRT-CI	All	168	19·9%	0·16	
	Amyloid-positive	93	24·2%	0·06	
	Amyloid-negative	75	31·6%	0·33	

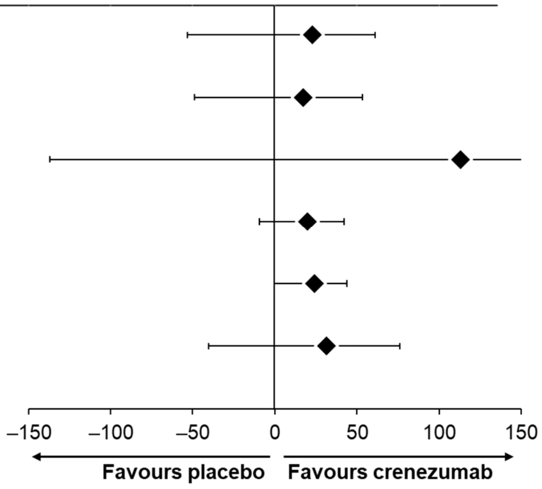

API ADAD composite=Alzheimer's Prevention Initiative preclinical autosomal-dominant Alzheimer's disease composite test total score. CI=confidence interval. FCSRT-CI=Free and Cued Selective Reminding Test – Cueing Index.

Figure S5: **Biomarker treatment effects in crenezumab- vs placebo-treated mutation carriers (A–Z)**

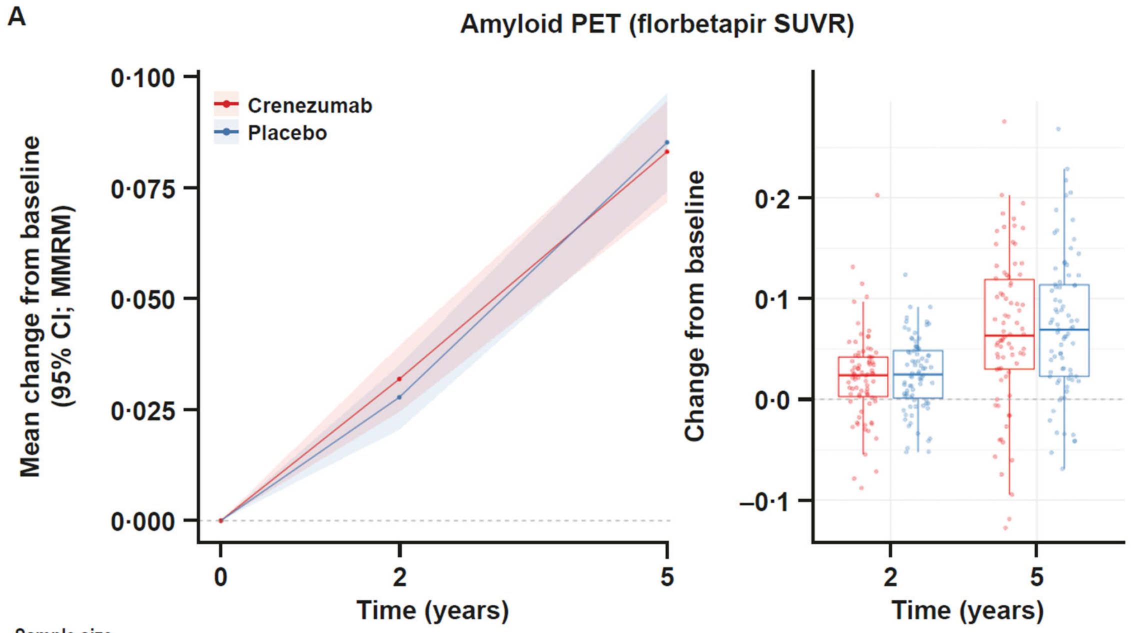

Sample size			
Crenezumab – Carrier	84	80	78
Placebo – Carrier	84	80	79

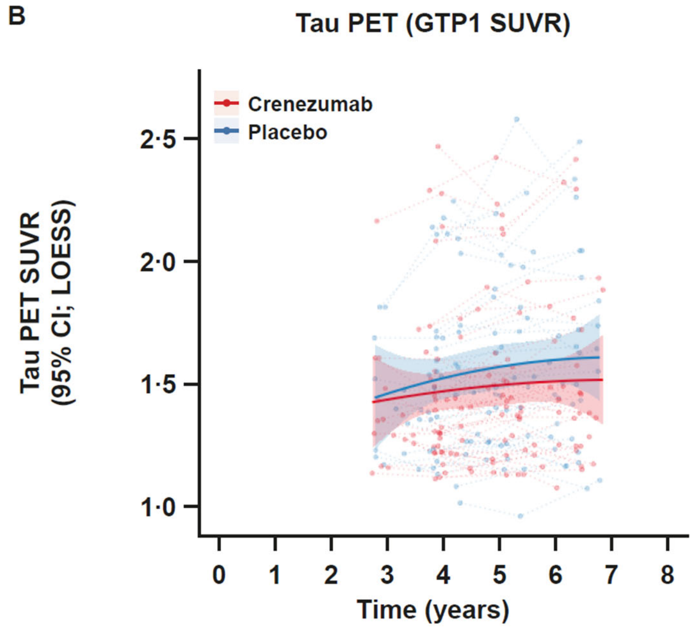

C

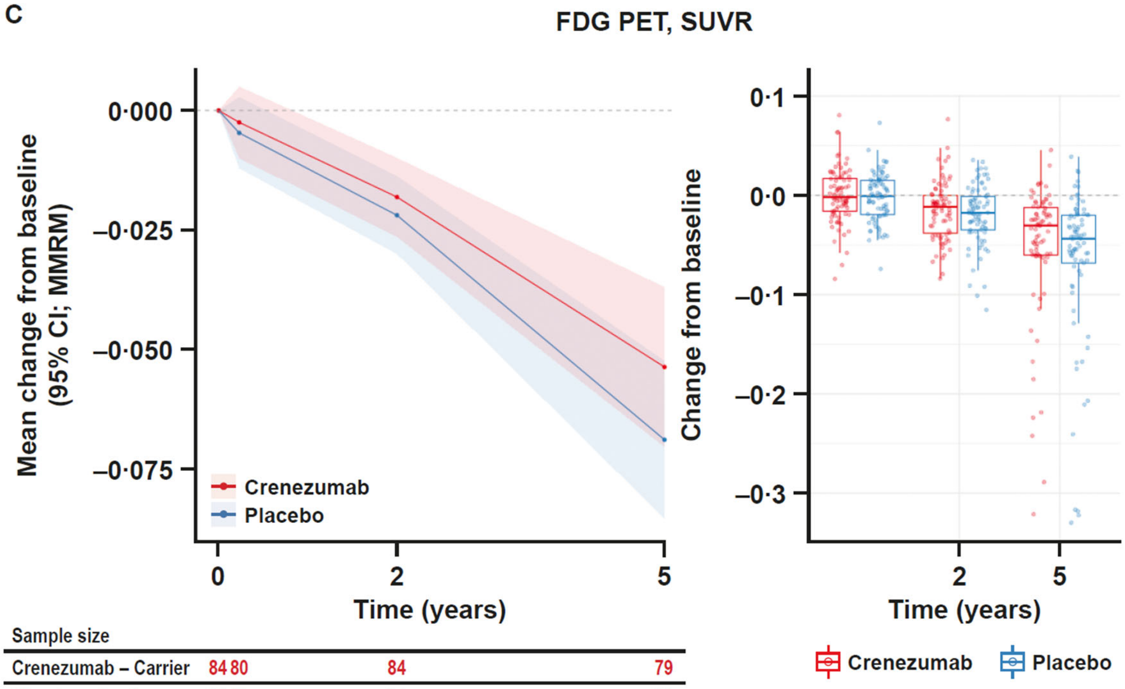

Sample size

Crenezumab – Carrier	84	80	84	79
Placebo – Carrier	84	80	84	81

D

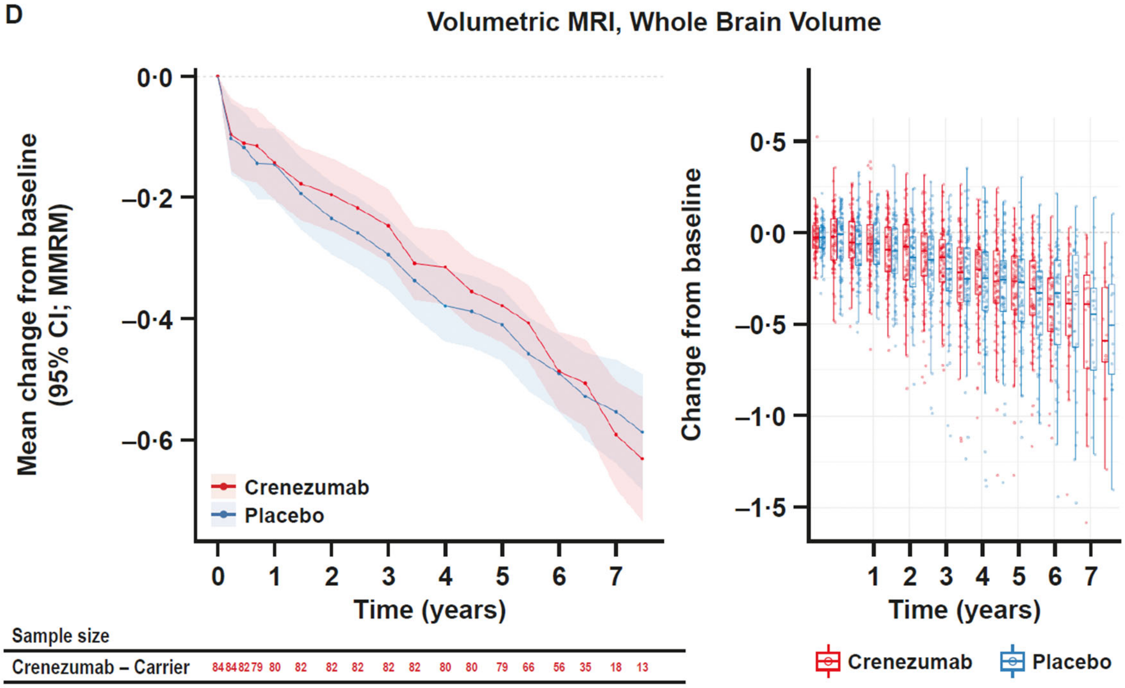

Sample size

Crenezumab – Carrier	84	84	82	79	80	82	82	82	82	82	80	80	79	66	56	35	18	13
Placebo – Carrier	84	83	83	83	82	83	81	82	82	81	81	80	78	70	56	34	20	15

E

Volumetric MRI, Bilateral Hippocampus

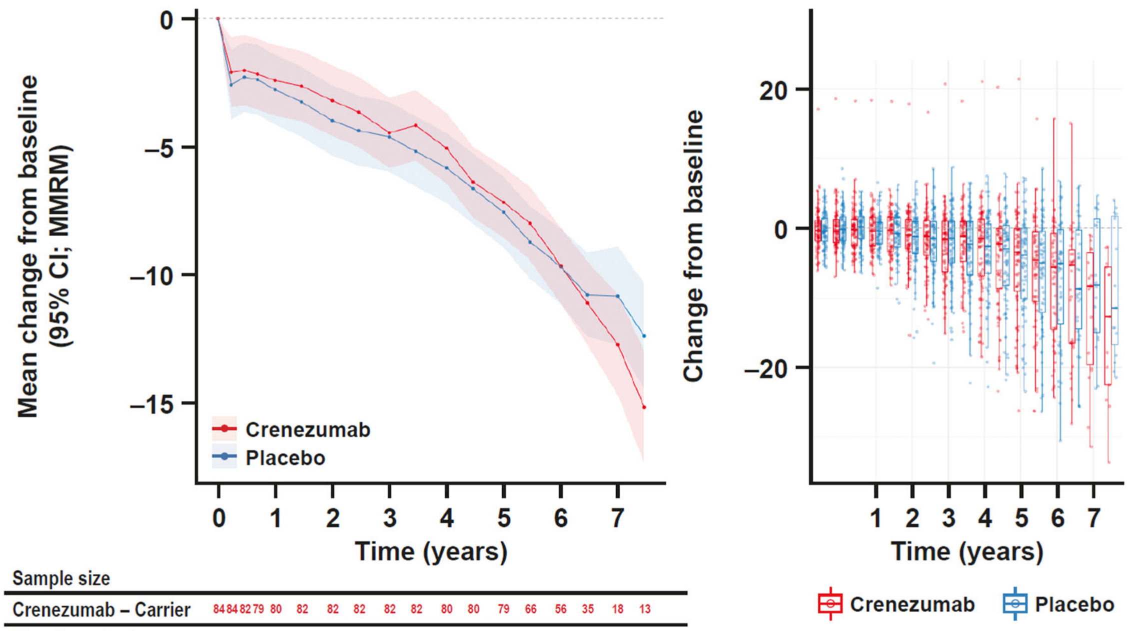

Sample size

Crenezumab – Carrier	84	84	82	79	80	82	82	82	82	82	80	80	79	66	56	35	18	13
Placebo – Carrier	84	83	83	83	82	83	81	82	82	81	81	80	78	70	56	34	20	15

F

Volumetric MRI, Brain Lateral Ventricle Volume

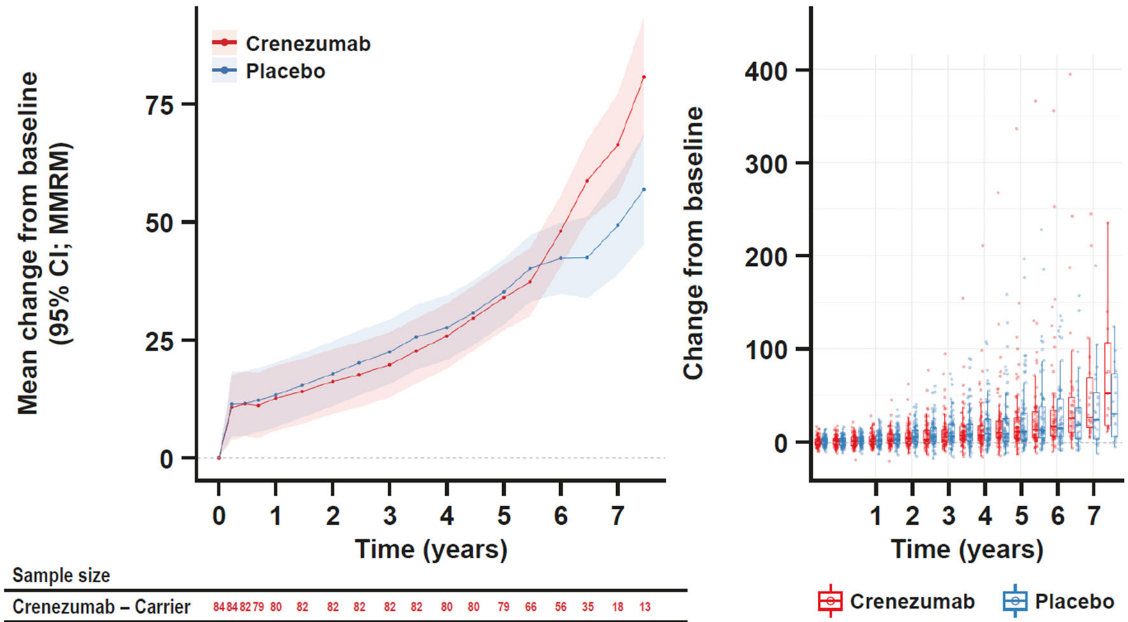

Sample size

Crenezumab – Carrier	84	84	82	79	80	82	82	82	82	82	80	80	79	66	56	35	18	13
Placebo – Carrier	84	83	83	83	82	83	81	82	82	81	81	80	78	70	56	34	20	15

G

CSF total Aβ40 (pg/mL)

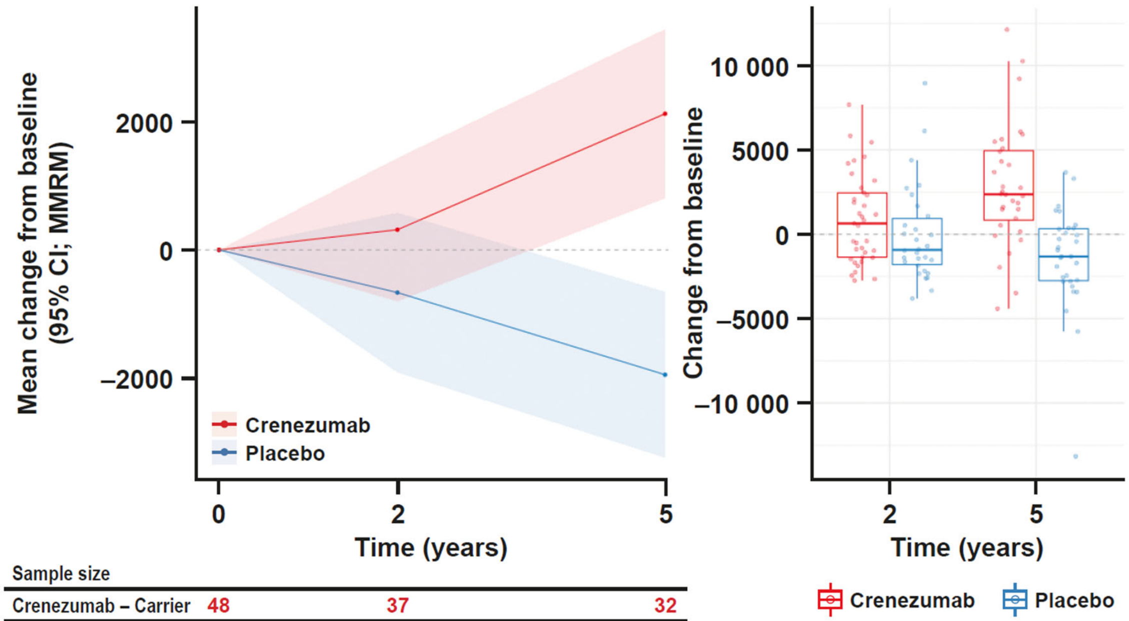

Sample size			
Crenezumab – Carrier	48	37	32
Placebo – Carrier	42	30	31

H

CSF total Aβ42 (pg/mL)

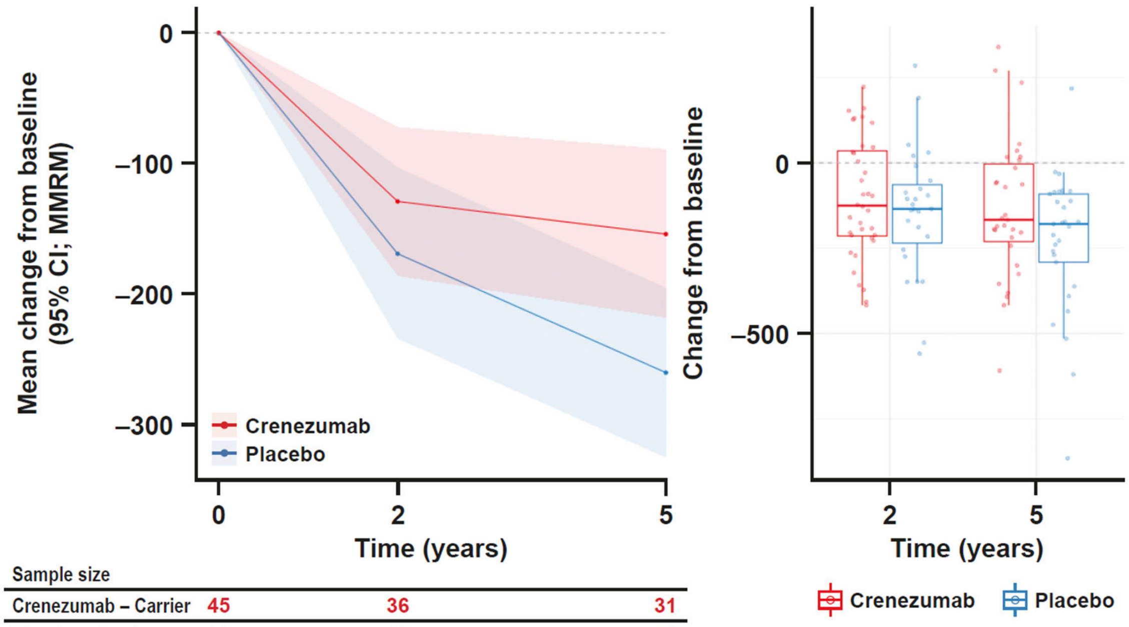

Sample size			
Crenezumab – Carrier	45	36	31
Placebo – Carrier	39	27	29

I

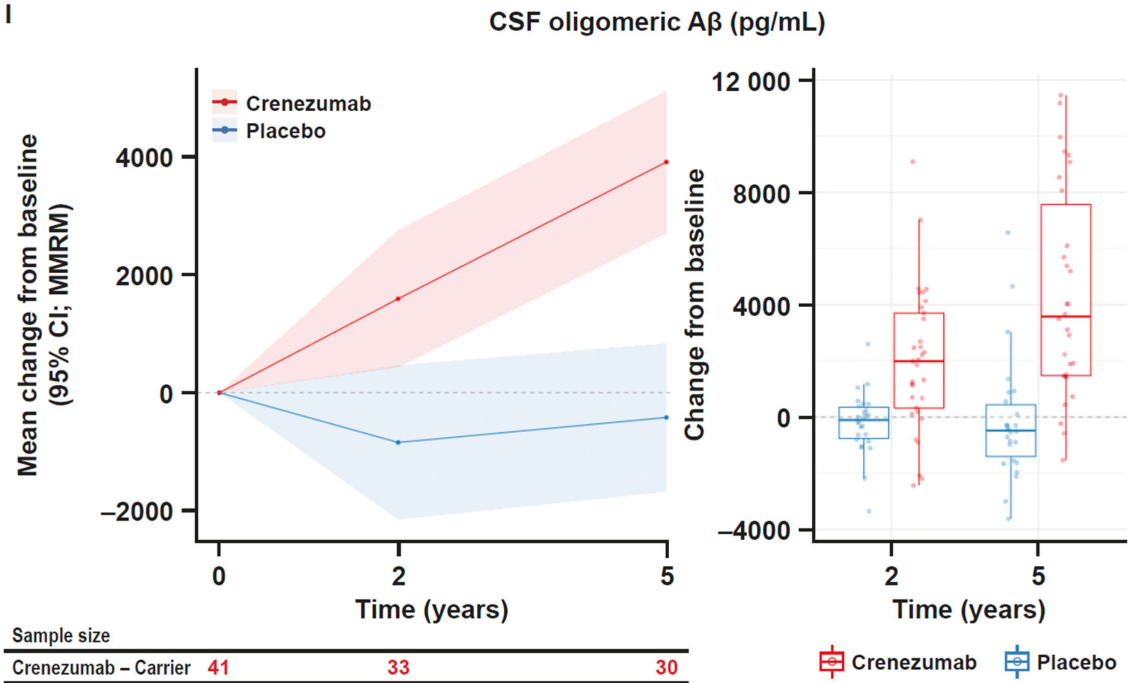

Sample size			
Crenezumab – Carrier	41	33	30
Placebo – Carrier	38	26	26

J

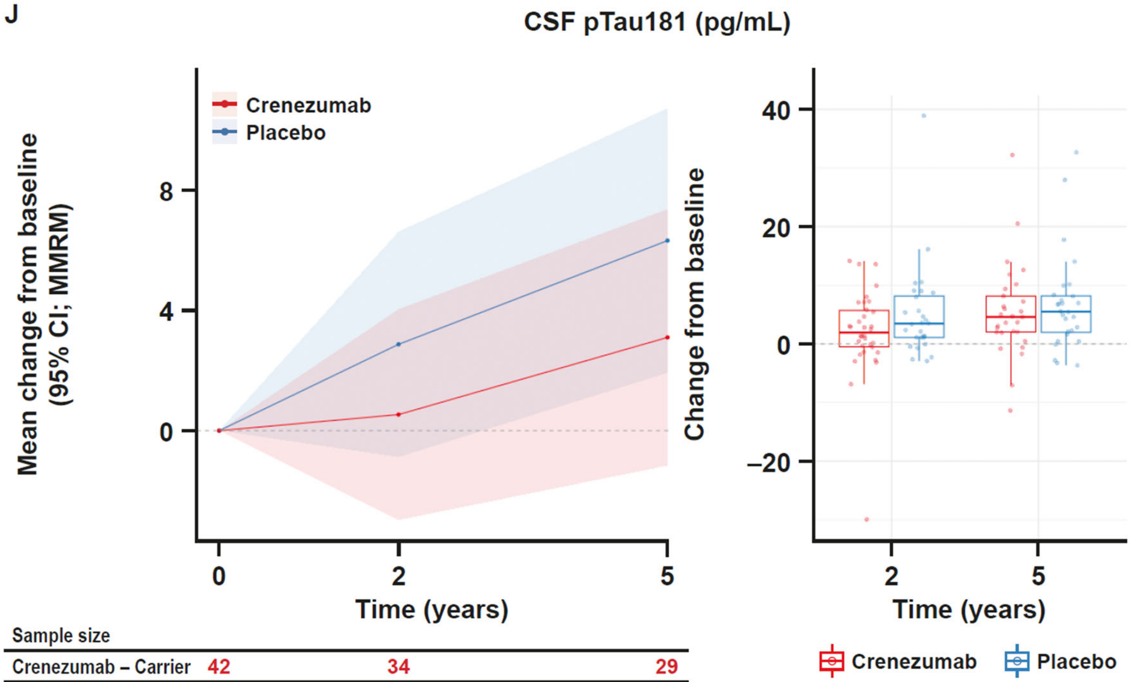

Sample size			
Crenezumab – Carrier	42	34	29
Placebo – Carrier	39	28	28

K CSF tTau (pg/mL)

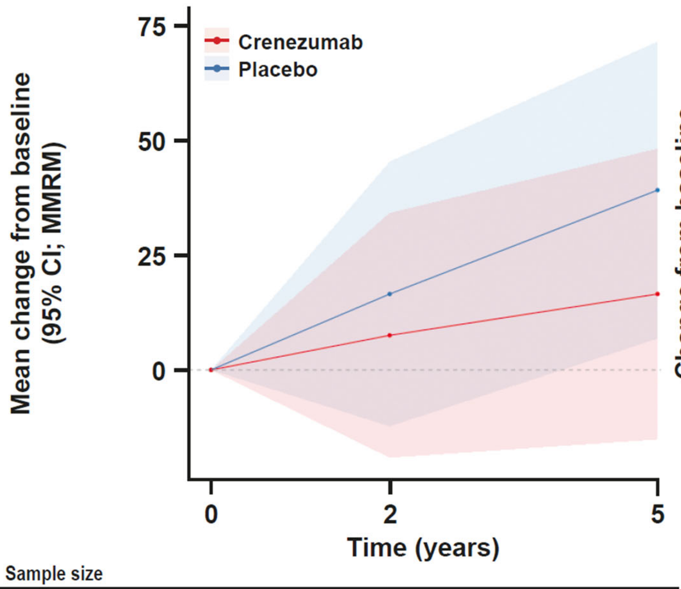

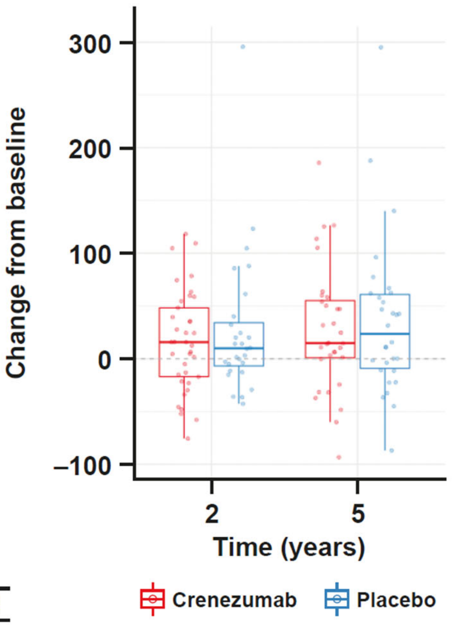

Sample size			
Crenezumab – Carrier	48	37	32
Placebo – Carrier	41	28	30

L Log_{10} CSF NfL (pg/mL)

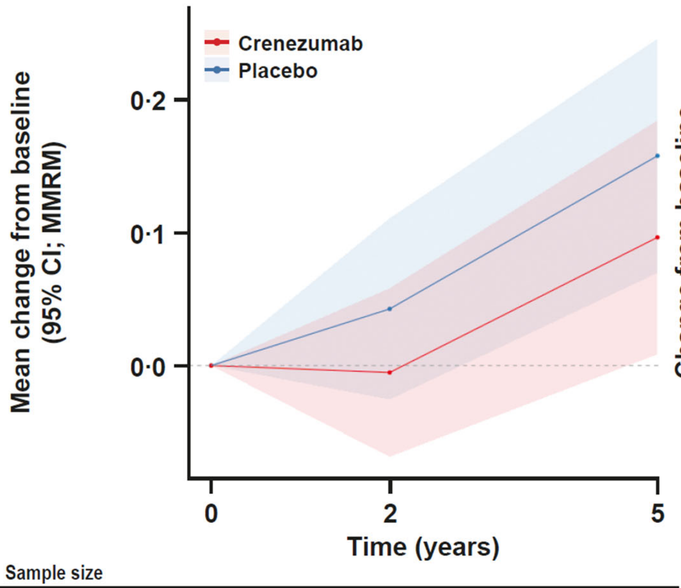

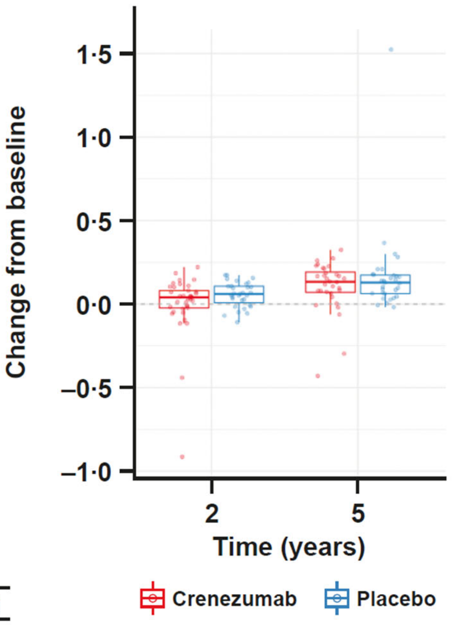

Sample size			
Crenezumab – Carrier	48	37	32
Placebo – Carrier	42	31	31

M

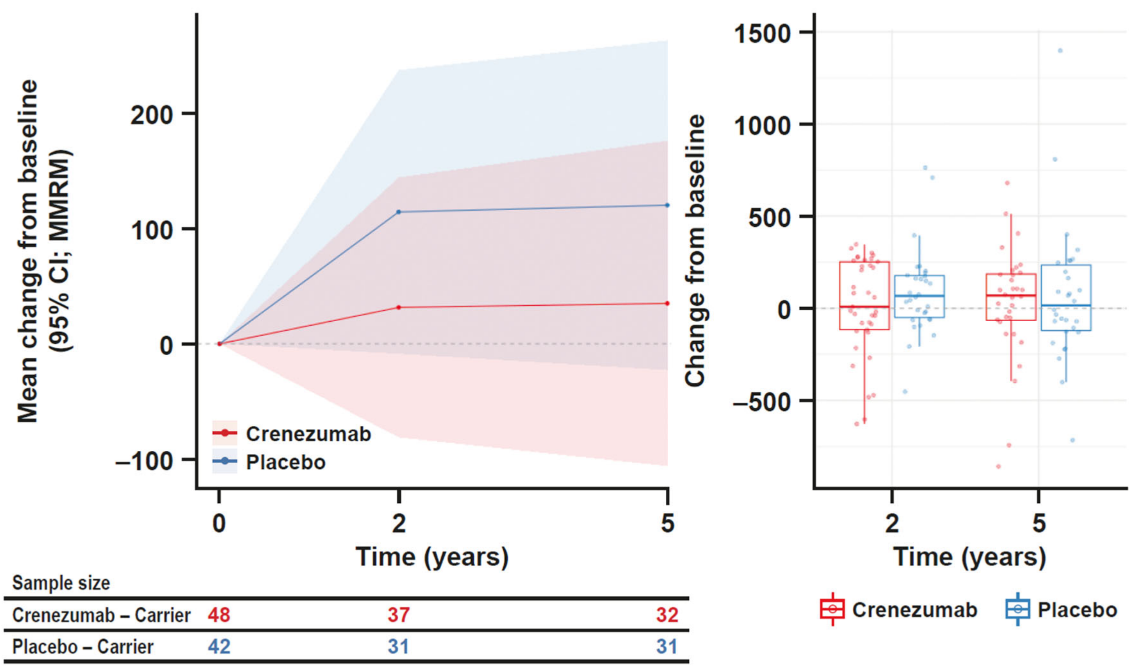

Sample size			
Crenezumab – Carrier	48	37	32
Placebo – Carrier	42	31	31

N

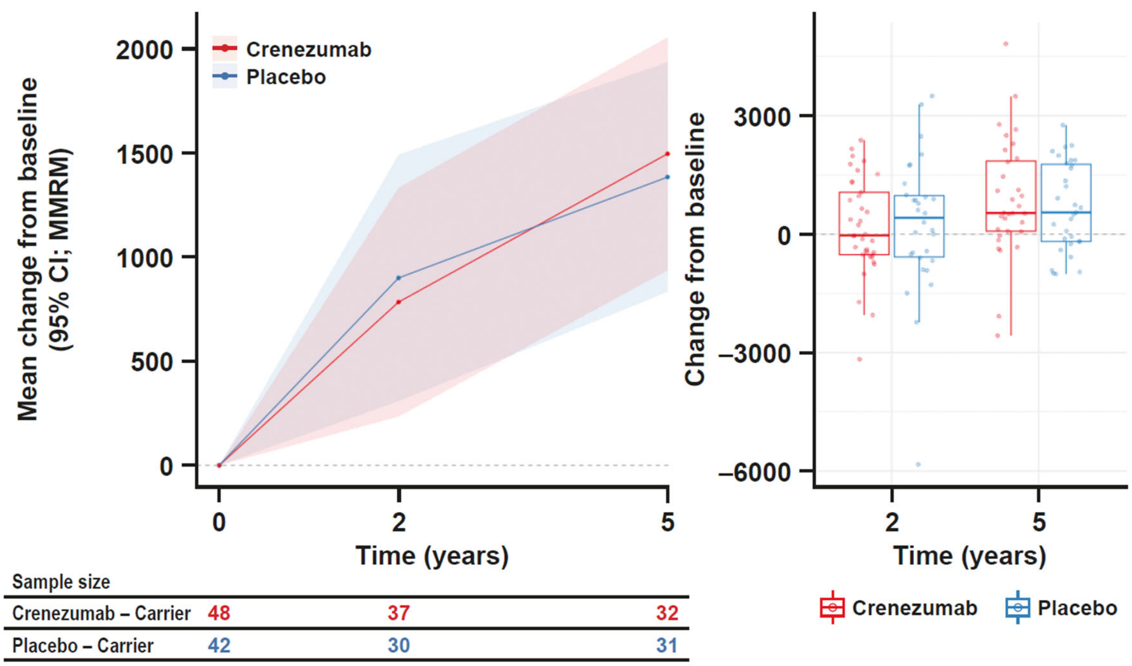

Sample size			
Crenezumab – Carrier	48	37	32
Placebo – Carrier	42	30	31

O

CSF sTREM2 (ng/mL)

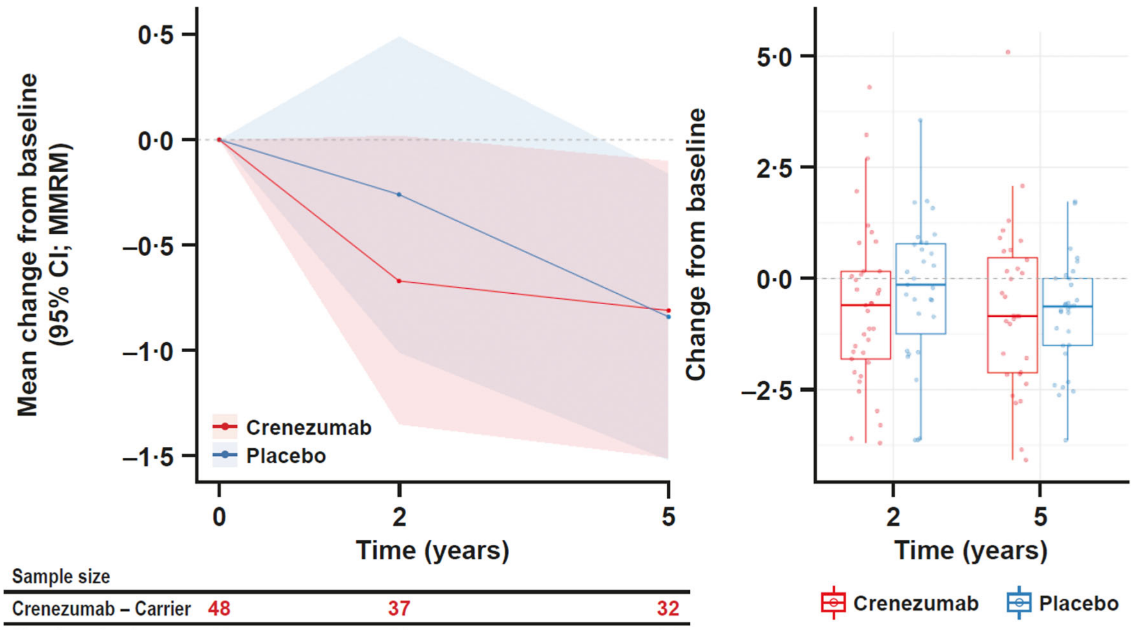

Sample size			
Crenezumab – Carrier	48	37	32
Placebo – Carrier	42	31	31

P

CSF YKL-40 (ng/L)

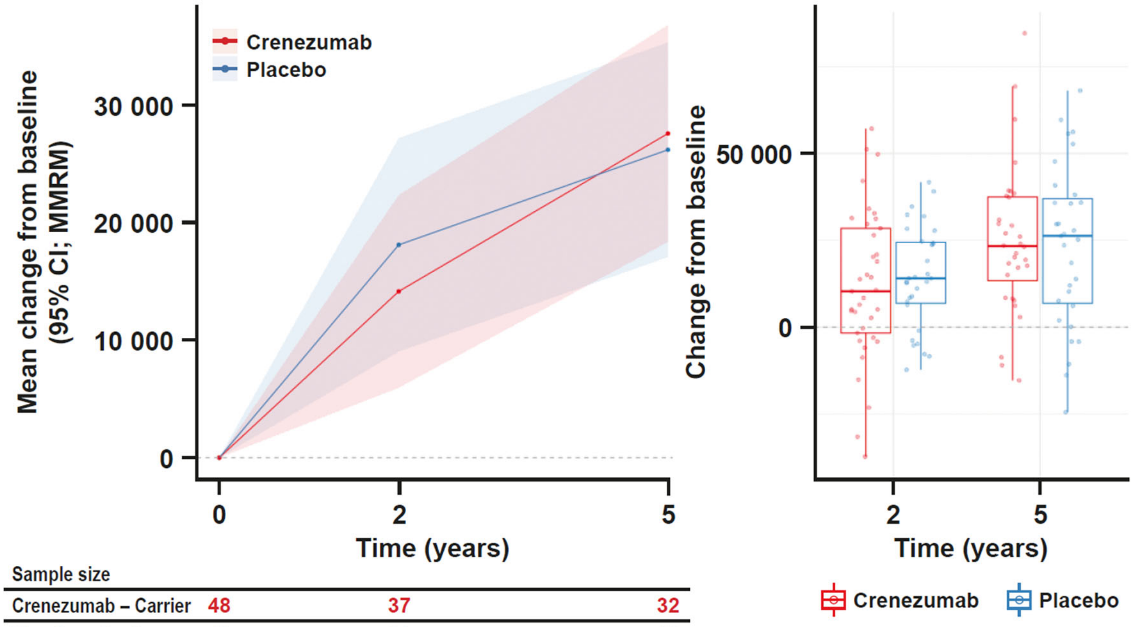

Sample size			
Crenezumab – Carrier	48	37	32
Placebo – Carrier	42	31	31

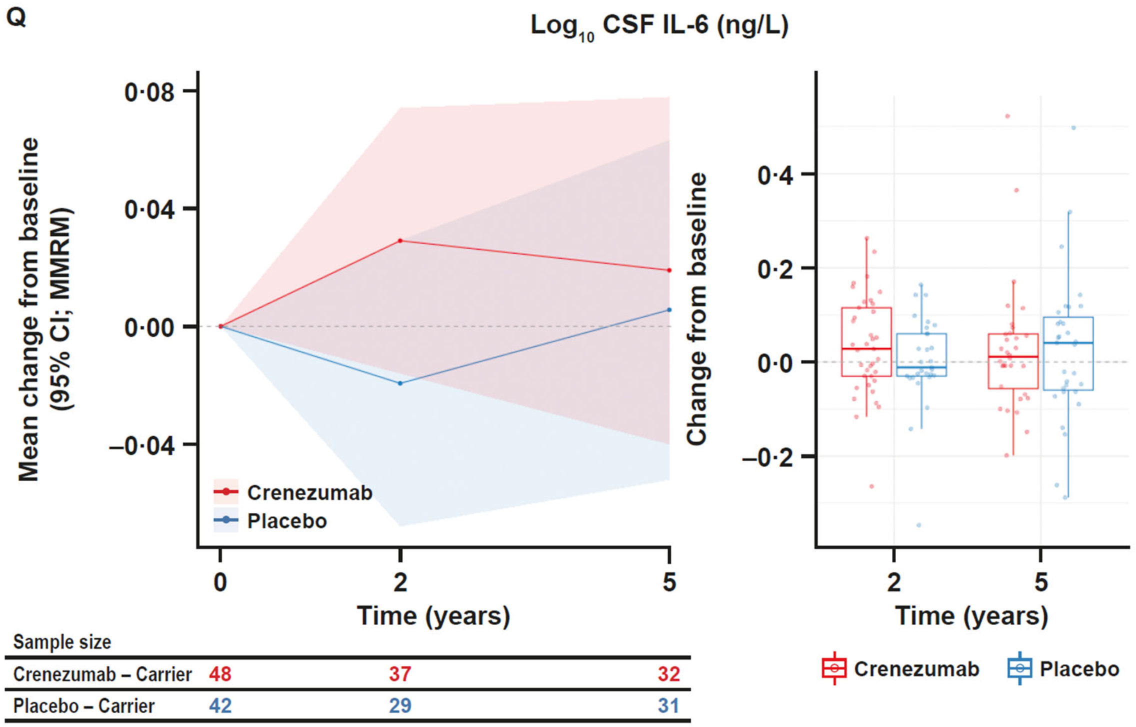
Q
Log10 CSF IL-6 (ng/L)
Mean change from baseline (95% CI; MMRM)
0·08
0·04
0·00
−0·04
Crenezumab
Placebo
0
2
5
Time (years)
Change from baseline
0·4
0·2
0·0
−0·2
2
5
Time (years)
Crenezumab
Placebo
Sample size
Crenezumab – Carrier 48 37 32
Placebo – Carrier 42 29 31

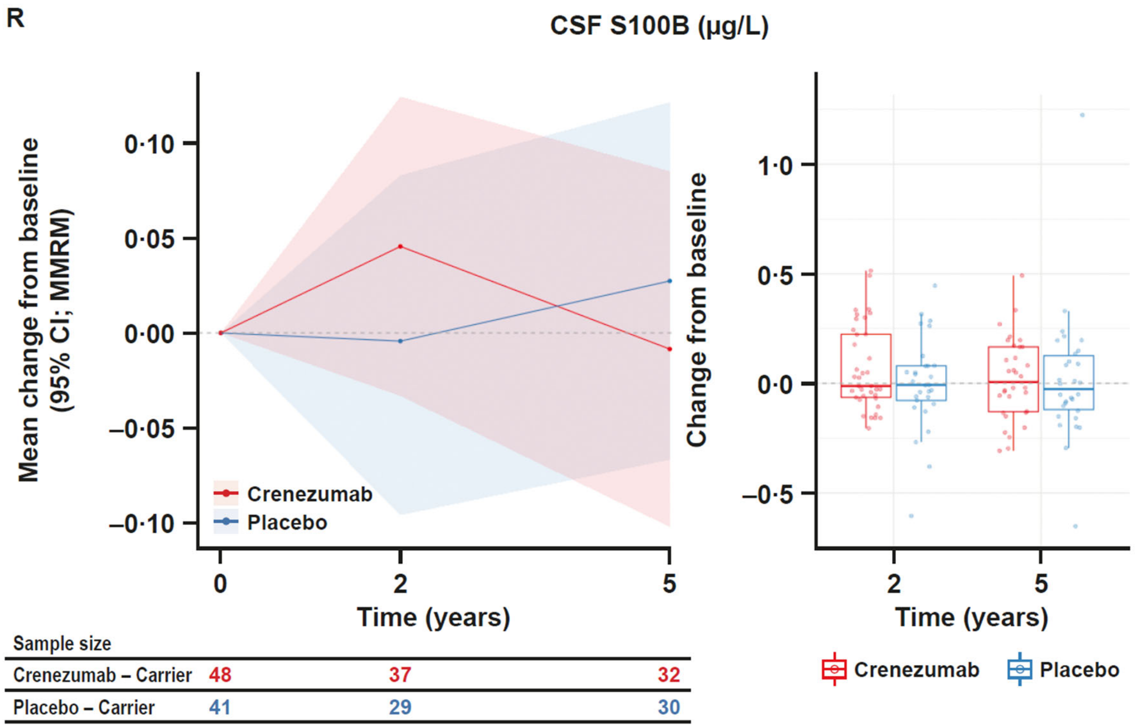
R
CSF S100B (µg/L)
Mean change from baseline (95% CI; MMRM)
0·10
0·05
0·00
−0·05
−0·10
Crenezumab
Placebo
0
2
5
Time (years)
Change from baseline
1·0
0·5
0·0
−0·5
2
5
Time (years)
Crenezumab
Placebo
Sample size
Crenezumab – Carrier 48 37 32
Placebo – Carrier 41 29 30

S Plasma total Aβ40 (pg/mL)

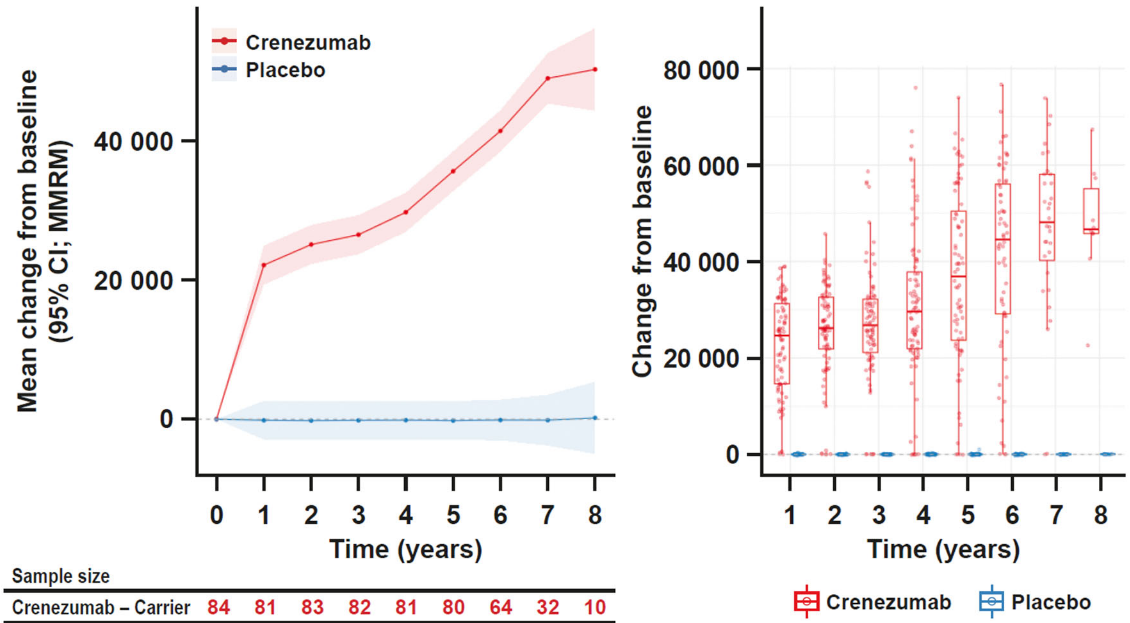

Sample size

Crenezumab – Carrier	84	81	83	82	81	80	64	32	10
Placebo – Carrier	84	83	82	82	80	76	66	31	13

T Plasma total Aβ42 (pg/mL)

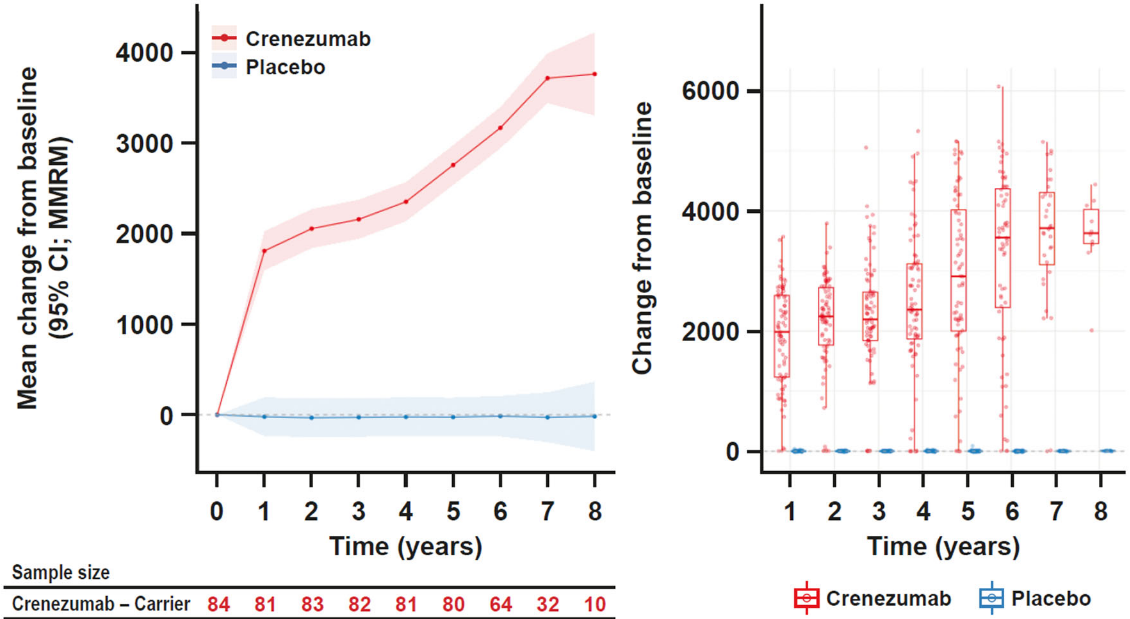

Sample size

Crenezumab – Carrier	84	81	83	82	81	80	64	32	10
Placebo – Carrier	84	83	82	82	80	76	66	31	13

U

Log_{10} Plasma pTau181 (pg/mL)

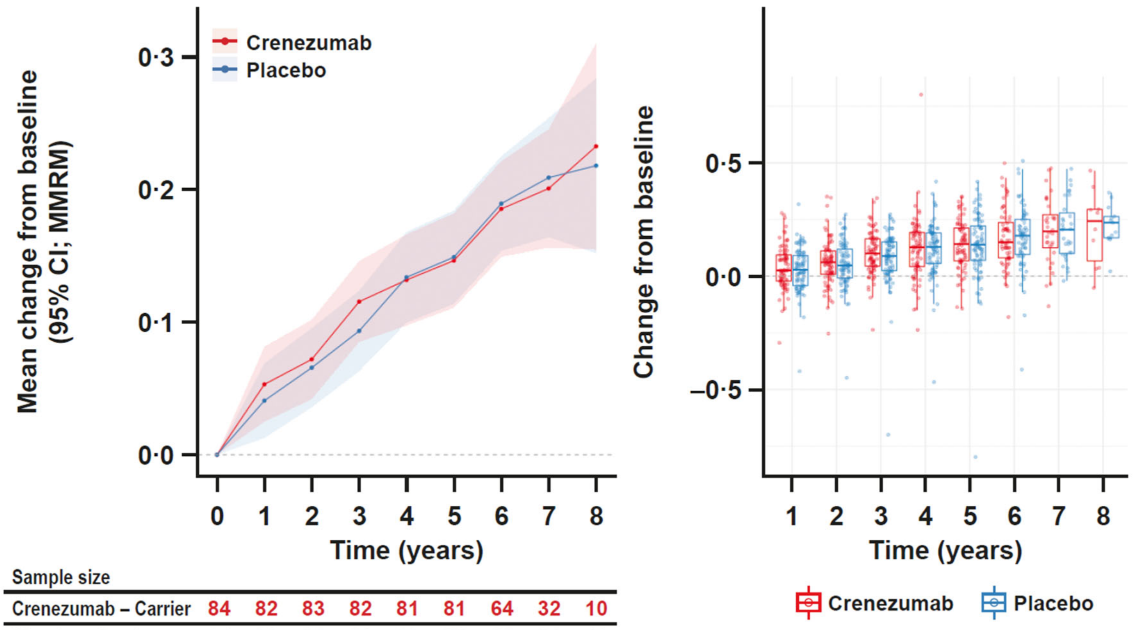

Sample size									
Crenezumab – Carrier	84	82	83	82	81	81	64	32	10
Placebo – Carrier	84	83	82	82	80	76	66	31	13

V

Log_{10} Plasma pTau217 (pg/mL)

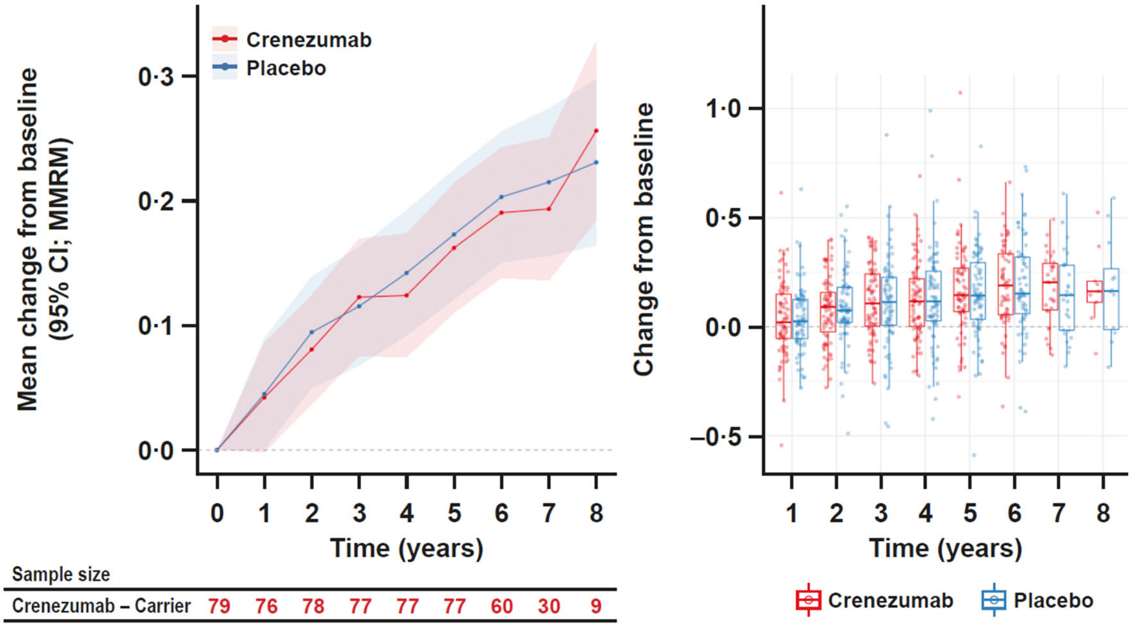

Sample size									
Crenezumab – Carrier	79	76	78	77	77	77	60	30	9
Placebo – Carrier	80	78	76	77	76	72	62	27	12

W

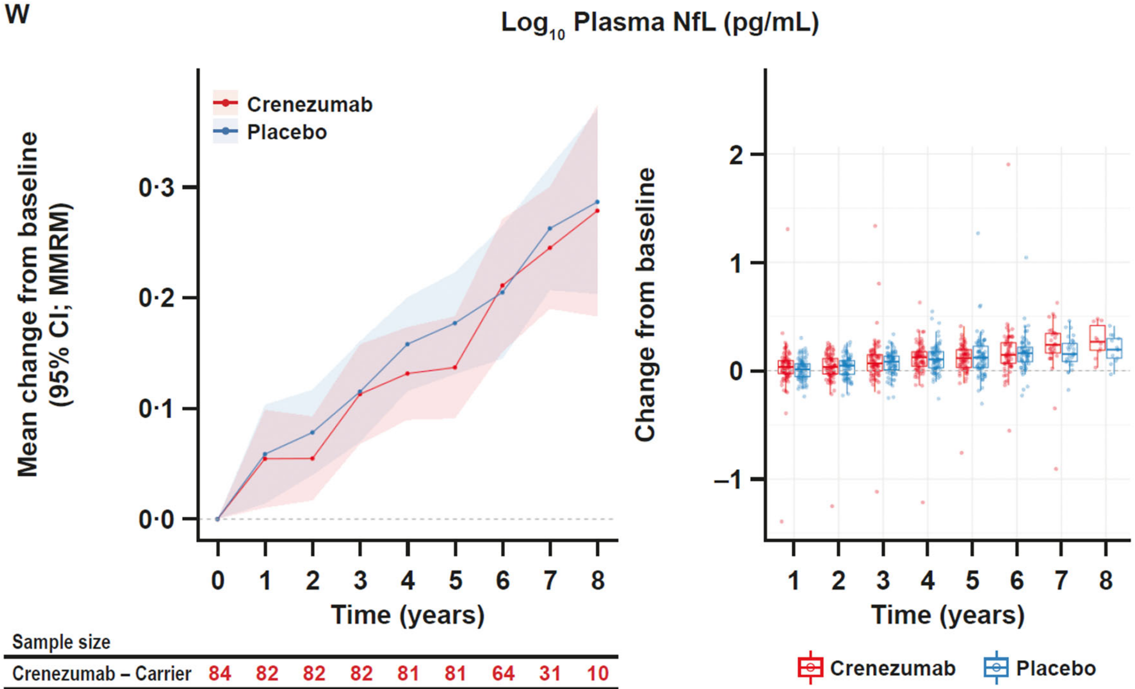

Sample size									
Crenezumab – Carrier	84	82	82	82	81	81	64	31	10
Placebo – Carrier	84	83	82	82	80	76	66	31	13

X

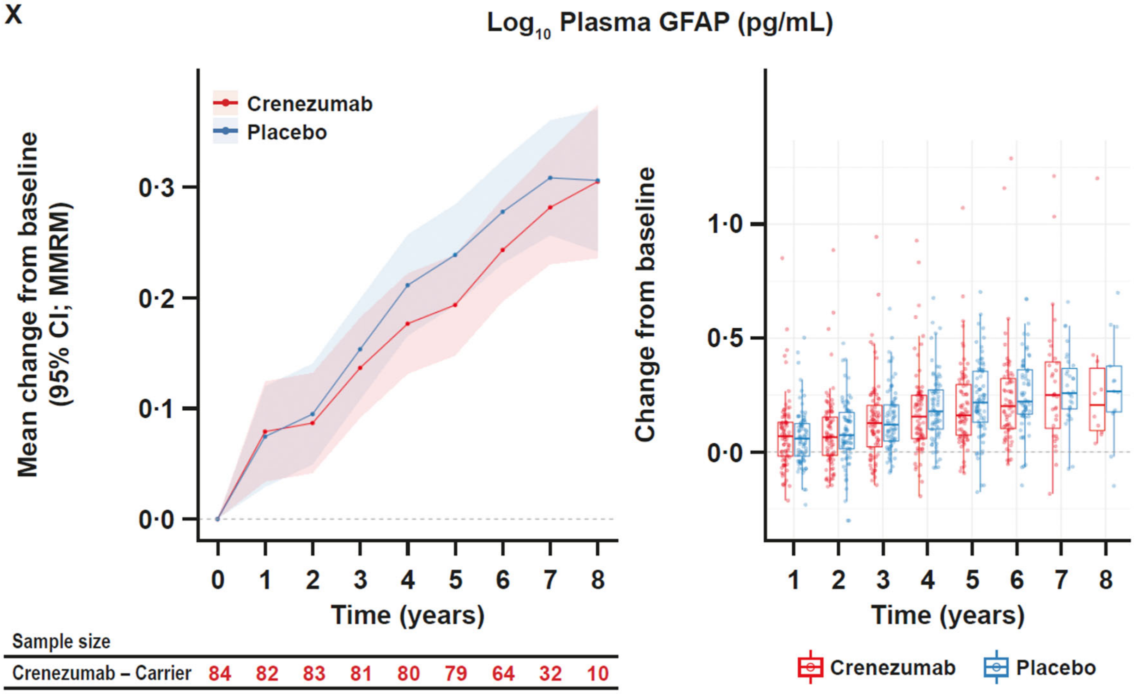

Sample size									
Crenezumab – Carrier	84	82	83	81	80	79	64	32	10
Placebo – Carrier	83	82	81	82	80	76	65	31	13

Y

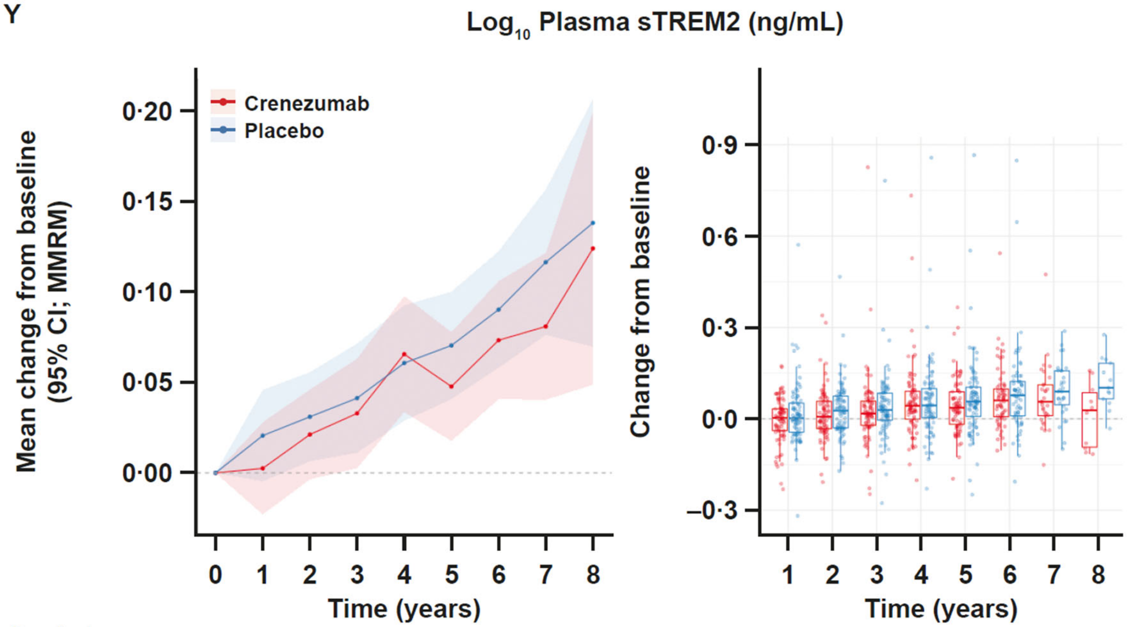

Sample size

Crenezumab – Carrier	84	82	82	82	81	81	64	31	10
Placebo – Carrier	84	83	82	82	80	76	66	31	13

Z

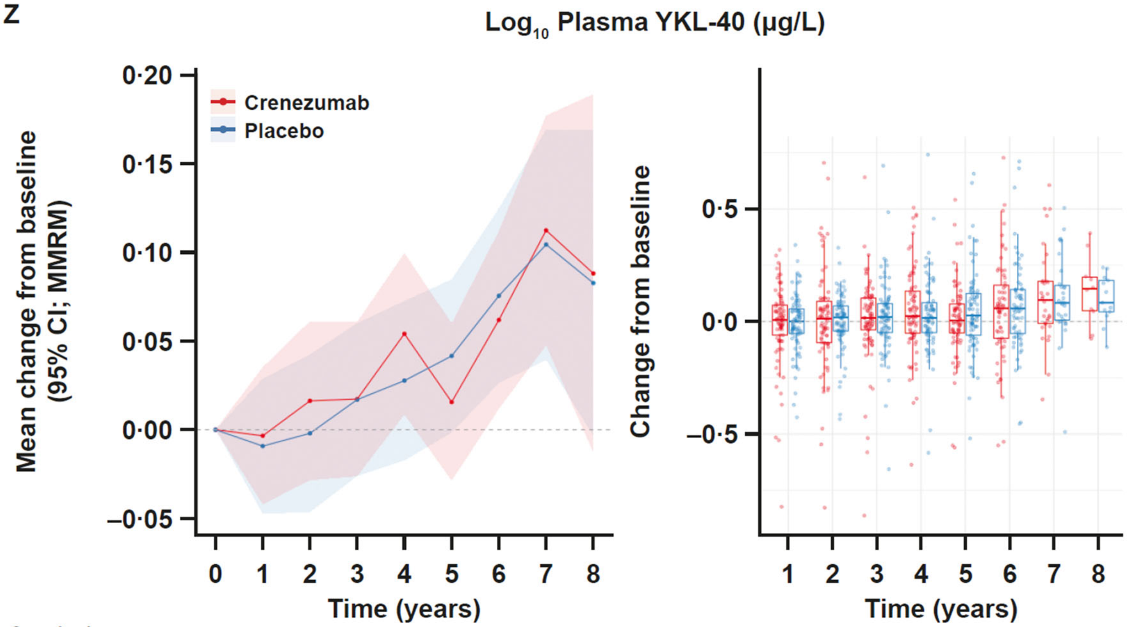

Sample size

Crenezumab – Carrier	84	82	82	82	81	81	64	32	10
Placebo – Carrier	84	83	82	82	80	76	66	31	13

Aβ, amyloid-beta; CI, confidence interval; CSF, cerebrospinal fluid; FDG, fluorodeoxyglucose; GFAP, glial fibrillary acidic protein; GTP1, Genentech Tau Probe 1; IL-6, interleukin-6; LOESS, locally estimated/weighted scatterplot smoothing; MMRM, mixed models for repeated measures; MRI, magnetic resonance imaging; NfL, neurofilament light; PET, positron emission tomography; pTau181/217, phosphorylated tau at threonine

181/217; S100B, S100 calcium-binding protein B; sTREM2, soluble triggering receptor expressed on myeloid cells 2; SUVR, standardised uptake value ratio; tTau, total tau; YKL-40, chitinase-3-like protein 1.

Figure S6: **Dual primary endpoint sensitivity analysis: MMRM**

A. API ADAD composite

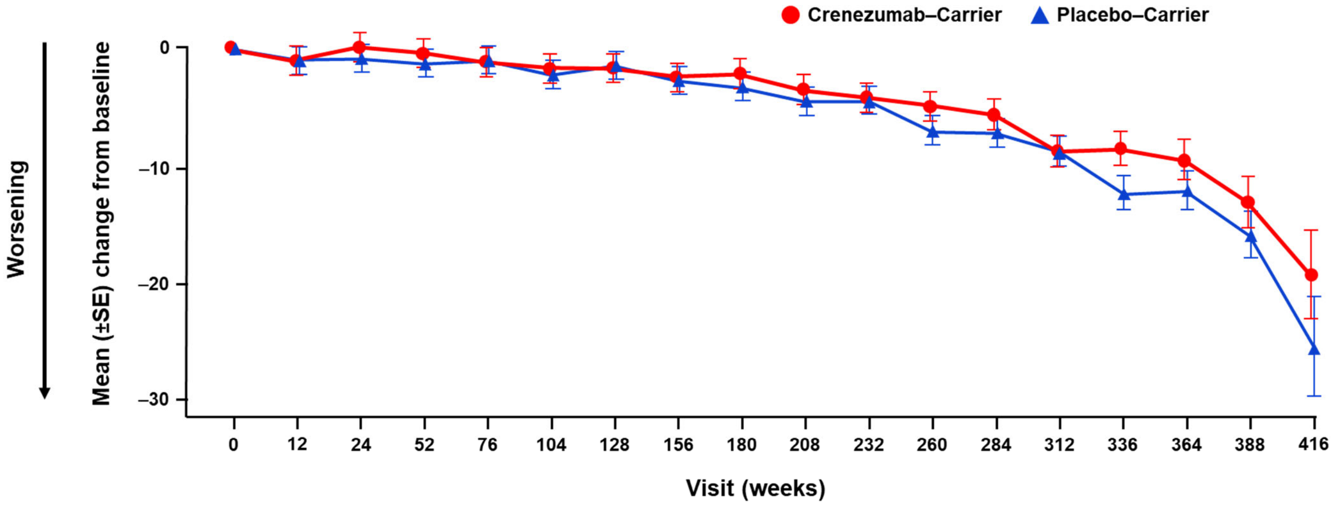

Number of participants

Crenezumab–Carrier	83	83	81	81	82	82	82	81	80	76	77	78	67	57	42	25	13	4
Placebo–Carrier	84	84	84	83	83	82	82	82	81	78	82	79	72	58	43	26	18	5

B. FCSRT-CI

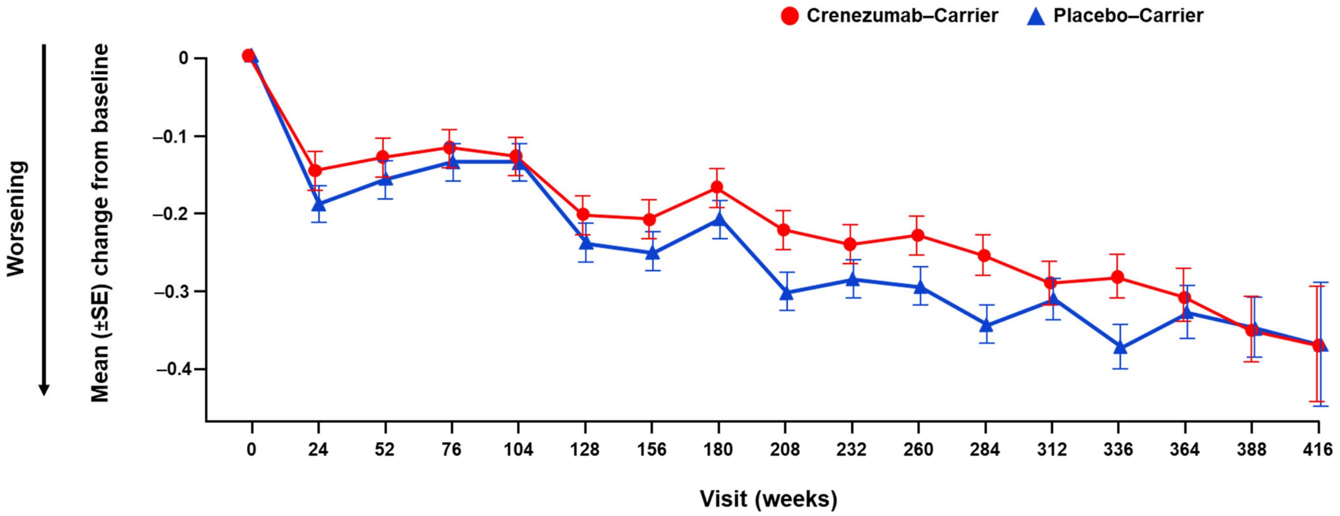

Number of participants

Crenezumab–Carrier	84	82	82	83	83	83	82	80	78	78	79	68	57	43	25	14	4
Placebo–Carrier	83	83	81	82	81	82	81	80	77	80	78	73	57	41	25	18	5

MMRM analysis adjusted for baseline score, baseline age, education, *APOE* ε4, CDR-GS, treatment, visit, baseline-by-visit interaction, and treatment-by-visit interaction to estimate the mean change from baseline. API ADAD composite=Alzheimer's Prevention Initiative preclinical autosomal-dominant Alzheimer's disease composite test

total score. *APOE* ε4=apolipoprotein E ε4 allele. CDR-GS=Clinical Dementia Rating Scale – Global Score. FCSRT-CI=Free and Cued Selective Reminding Test – Cueing Index. MMRM=mixed models for repeated measures. SE=standard error.

Figure S7: **PK/PD analysis**

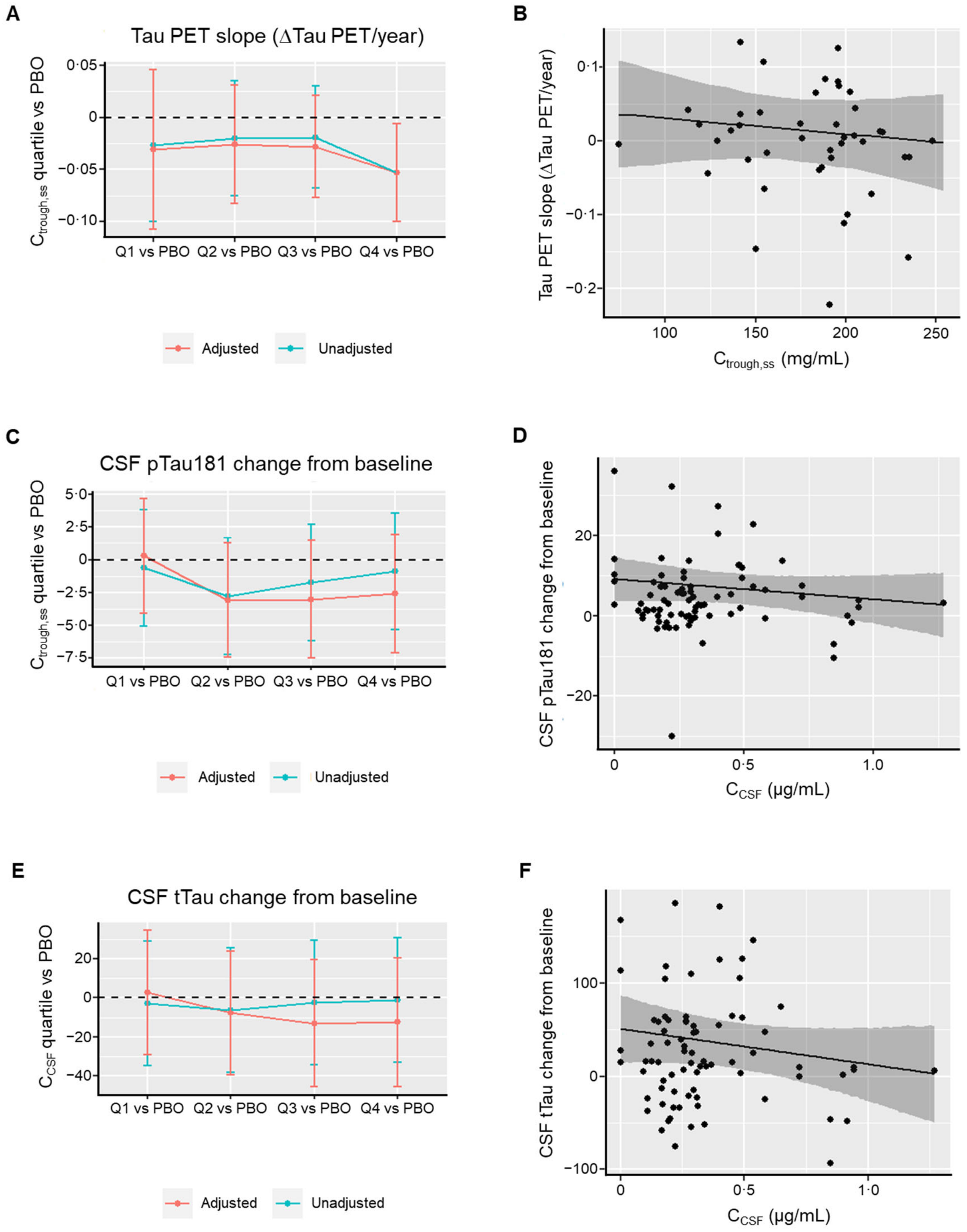

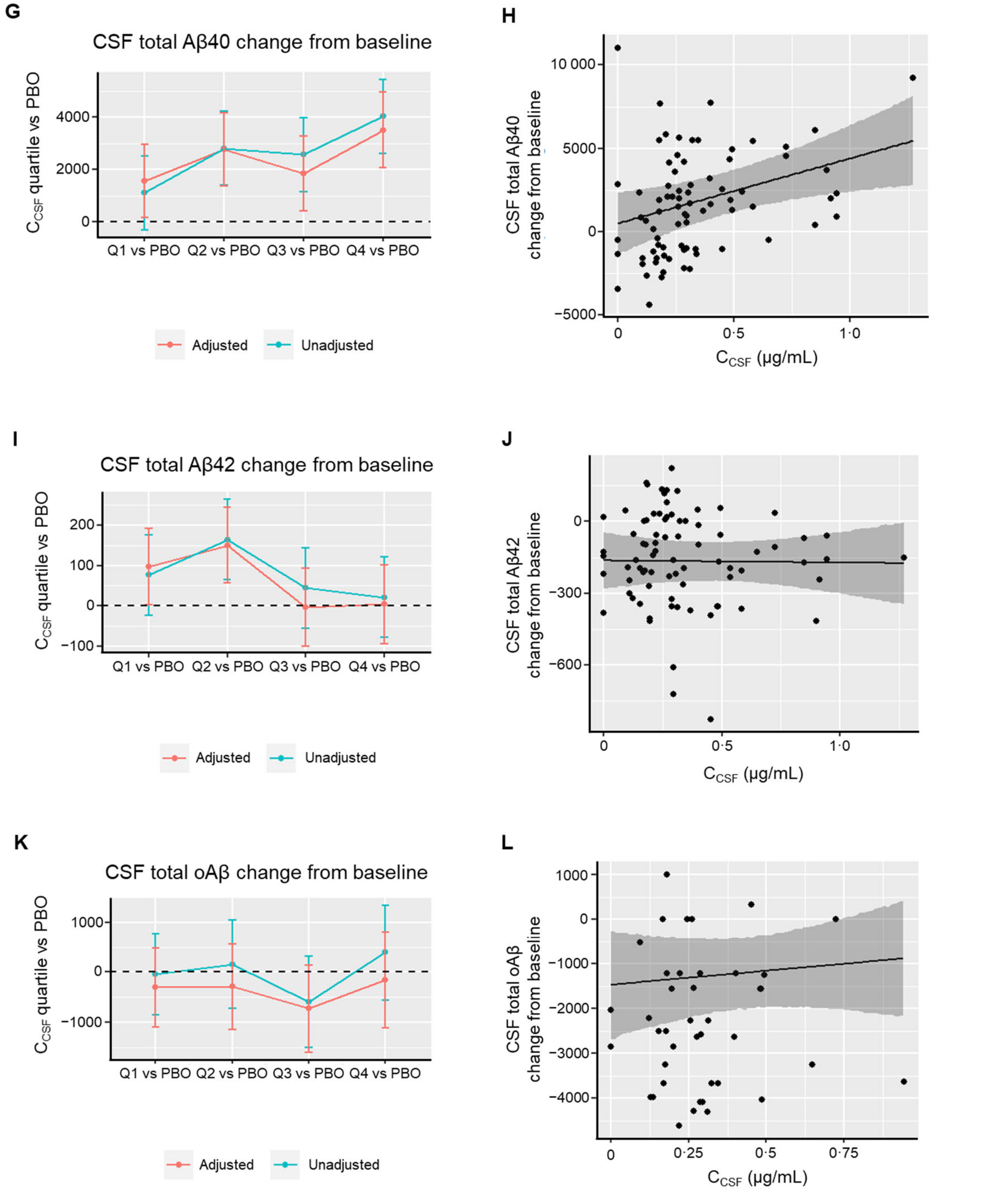

PK/PD analysis of crenezumab $C_{trough,ss}$ and Tau PET slope using a categorical model (A) and linear regression model (B), CSF pTau181 using a categorical model (C) and linear regression model (D), CSF tTau using a categorical model (E) and linear regression model (F), CSF total Aβ40 using a categorical model (G) and linear regression model (H), and CSF total Aβ42 using a categorical model (I) and linear regression model (J). Δ=delta. Aβ=amyloid-beta. C=concentration. CSF=cerebrospinal fluid. $C_{trough.ss}$=steady-state average serum C_{trough} concentration. oAβ=oligomeric amyloid-beta. PBO=placebo. PD=pharmacodynamics. PET=positron emission tomography. PK=pharmacokinetics. pTau181=phosphorylated tau at threonine 181. Q=quartile. tTau=total tau.

References

1. Bretz F, Maurer W, Brannath W, Posch M. A graphical approach to sequentially rejective multiple test procedures. *Stat Med* 2009;**28**:586-604. DOI: 10.1002/sim.3495.
2. Bretz F, Posch M, Glimm E, Klinglmueller F, Maurer W, Rohmeyer K. Graphical approaches for multiple comparison procedures using weighted Bonferroni, Simes, or parametric tests. *Biom J* 2011;**53**:894-913. DOI: 10.1002/bimj.201000239.
3. US Department of Health and Human Services, US Food and Drug Administration (FDA), Center for Drug Evaluation and Research (CDER), Center for Biologics Evaluation and Research (CBER). Multiple Endpoints in Clinical Trials: Guidance for Industry. https://www.fda.gov/regulatory-information/search-fda-guidance-documents/multiple-endpoints-clinical-trials-guidance-industry. Accessed September 18, 2024.
4. Bittner T, Zetterberg H, Teunissen CE, et al. Technical performance of a novel, fully automated electrochemiluminescence immunoassay for the quantitation of β-amyloid (1-42) in human cerebrospinal fluid. *Alzheimers Dement* 2016;**12**:517-526. DOI: 10.1016/j.jalz.2015.09.009.
5. Lifke V, Kollmorgen G, Manuilova E, et al. Elecsys® Total-Tau and Phospho-Tau (181P) CSF assays: analytical performance of the novel, fully automated immunoassays for quantification of tau proteins in human cerebrospinal fluid. *Clin Biochem* 2019;**72:**:30-38. DOI: 10.1016/j.clinbiochem.2019.05.005.
6. Liu L, Kwak H, Lawton TL, et al. An ultra-sensitive immunoassay detects and quantifies soluble Aβ oligomers in human plasma. *Alzheimers Dement* 2022;**18**:1186-1202. DOI: 10.1002/alz.12457.
7. Alzheimer's Disease Data Initiative. The NeuroToolKit. https://www.alzheimersdata.org/ntk. Accessed October 3, 2024.
8. Denkinger M, Singh A, Liu J, et al. Longitudinal head-to-head of high-precision plasma p-Tau217 assays to better determine disease progression. In: Alzheimer's Association International Conference (AAIC); July 12-16, 2025; Toronto, Canada.
9. Klunk WE, Koeppe RA, Price JC, et al. The Centiloid Project: standardizing quantitative amyloid plaque estimation by PET. *Alzheimers Dement* 2015;**11**:1-15.e11-14. DOI: 10.1016/j.jalz.2014.07.003.
10. Navitsky M, Joshi AD, Kennedy I, et al. Standardization of amyloid quantitation with florbetapir standardized uptake value ratios to the centiloid scale. *Alzheimers Dement* 2018;**14**:1565-1571. DOI: 10.1016/j.jalz.2018.06.1353.
11. Fleisher AS, Chen K, Liu X, et al. Using positron emission tomography and florbetapir F18 to image cortical amyloid in patients with mild cognitive impairment or dementia due to Alzheimer disease. *Arch Neurol* 2011;**68**:1404-1411. DOI: 10.1001/archneurol.2011.150.
12. Chen K, Roontiva A, Thiyyagura P, et al. Improved power for characterizing longitudinal amyloid-β PET changes and evaluating amyloid-modifying treatments with a cerebral white matter reference region. *J Nucl Med* 2015;**56**:560-566. DOI: 10.2967/jnumed.114.149732.
13. Salloway S, Honigberg LA, Cho W, et al. Amyloid positron emission tomography and cerebrospinal fluid results from a crenezumab anti-amyloid-beta antibody double-blind, placebo-controlled, randomized phase II study in mild-to-moderate Alzheimer's disease (BLAZE). *Alzheimers Res Ther* 2018;**10**:96. DOI: 10.1186/s13195-018-0424-5.
14. Chen K, Langbaum JB, Fleisher AS, et al. Twelve-month metabolic declines in probable Alzheimer's disease and amnestic mild cognitive impairment assessed using an empirically pre-defined statistical region-of-interest: findings from the Alzheimer's Disease Neuroimaging Initiative. *Neuroimage* 2010;**51**:654-664. DOI: 10.1016/j.neuroimage.2010.02.064.
15. Baker SL, Maass A, Jagust WJ. Considerations and code for partial volume correcting [^{18}F]-AV-1451 tau PET data. *Data Brief* 2017;**15**:648-657. DOI: 10.1016/j.dib.2017.10.024.
16. Su Y, D'Angelo GM, Vlassenko AG, et al. Quantitative analysis of PiB-PET with FreeSurfer ROIs. *PLoS One* 2013;**8**:e73377. DOI: 10.1371/journal.pone.0073377.
17. Su Y, Blazey TM, Snyder AZ, et al. Partial volume correction in quantitative amyloid imaging. *Neuroimage* 2015;**107:**:55-64. DOI: 10.1016/j.neuroimage.2014.11.058.
18. Fischl B. FreeSurfer. *Neuroimage* 2012;**62**:774-781. DOI: 10.1016/j.neuroimage.2012.01.021.
19. Chen K, Reiman EM, Alexander GE, et al. An automated algorithm for the computation of brain volume change from sequential MRIs using an iterative principal component analysis and its evaluation for the assessment of whole-brain atrophy rates in patients with probable Alzheimer's disease. *Neuroimage* 2004;**22**:134-143. DOI: 10.1016/j.neuroimage.2004.01.002.

PROTOCOL

**TITLE:**	**A DOUBLE-BLIND, PLACEBO-CONTROLLED PARALLEL-GROUP STUDY IN PRECLINICAL *PSEN1 E280A* MUTATION CARRIERS RANDOMIZED TO CRENEZUMAB OR PLACEBO, AND IN NON-RANDOMIZED, PLACEBO-TREATED NON-CARRIERS FROM THE SAME KINDRED, TO EVALUATE THE EFFICACY AND SAFETY OF CRENEZUMAB IN THE TREATMENT OF AUTOSOMAL-DOMINANT ALZHEIMER'S DISEASE**
**PROTOCOL NUMBER:**	GN28352
**VERSION NUMBER:**	10
**STUDY DRUG:**	Crenezumab (MABT5102A)
**NCT NUMBER**	NCT01998841
**MEDICAL MONITOR:**	[redacted], M.D., M.A.S.
**SPONSOR:**	F. Hoffmann-La Roche Ltd<br>CH-4070 Basel, Switzerland<br><br>In collaboration with:<br>Banner Alzheimer's Institute<br>901 East Willetta St.<br>Phoenix, AZ 85006
**DATE FINAL:**	See electronic date stamp below

PROTOCOL AMENDMENT APPROVAL

Date and Time (UTC)	Title	Approver's Name
23-Aug-2021 03:49:09	Company Signatory	[redacted]

CONFIDENTIAL

This clinical study is being sponsored globally by F. Hoffmann-La Roche Ltd of Basel, Switzerland. However, it may be implemented in individual countries by Roche's local affiliates, including Genentech, Inc. in the United States. The information contained in this document, especially any unpublished data, is the property of F. Hoffmann-La Roche Ltd (or under its control) and therefore is provided to you in confidence as an investigator, potential investigator, or consultant, for review by you, your staff, and an applicable Ethics Committee or Institutional Review Board. It is understood that this information will not be disclosed to others without written authorization from Roche except to the extent necessary to obtain informed consent from persons to whom the drug may be administered.

PROTOCOL HISTORY

Protocol	
**Version**	**Date Final**
9	19 March 2021
8	10 September 2020
7	18 December 2019
6	26 May 2017
5	9 March 2016
4	19 November 2014
3	19 July 2014
2	12 April 2013
1	20 August 2012

PROTOCOL AMENDMENT, VERSION 10
RATIONALE

Protocol GN28352 has been amended. Changes to the protocol, along with a rationale for each change, are summarized below:

- The primary efficacy outcome measures have been modified from annualized rate of change on the API ADAD Composite Cognitive Test Battery total score to a primary efficacy outcome family consisting of: 1) annualized rate of change on API ADAD Composite Cognitive Test Battery total score and 2) annualized rate of change on FCSRT Cueing Index. Promotion of the FCSRT from a secondary outcome to the primary efficacy outcome family was done due to concerns that preclinical ADAD participants may have learning effects and/or the ADAD composite may not be sensitive enough to change in this population. The FCSRT has been shown to be a sensitive measure of early episodic memory dysfunction-- the hallmark of emerging symptomatic AD and the most commonly observed, early, and consistent neuropsychological marker of AD.
- The Preclinical Alzheimer's Cognitive Composite (PACC), modified for the API Colombia trial is added to the list of exploratory cognitive endpoints to allow comparison of trial results with other contemporary AD prevention and treatment trials.
- Correction of Study Period A to B in the safety analysis population Section 4.10.4.
- Changed visit windowing for dosing days to allow for a 7 day window.

Additional minor changes have been made to improve readability, clarity, and consistency. Substantive new information appears in italics. This amendment represents cumulative changes to the original protocol.

TABLE OF CONTENTS

PROTOCOL AMENDMENT ACCEPTANCE FORM 10

PROTOCOL SYNOPSIS 11

1. BACKGROUND 28

1.1 Background on Disease 28

1.2 Background on Crenezumab 29

1.2.1 Summary of Nonclinical Studies 31

1.2.2 Clinical Experience 32

1.2.2.1 Safety Experience in Patients with Sporadic AD 33

1.2.2.2 Clinical Pharmacokinetics 34

1.2.2.3 Immunogenicity 35

1.3 Rationale for Doing this Study 36

1.3.1 Rationale for the Alzheimer’s Prevention Initiative 36

1.3.2 Rationale for Study 36

2. OBJECTIVES 38

2.1 Efficacy Objectives 38

2.1.1 Primary Efficacy Objective 38

2.1.2 Secondary Efficacy Objectives 39

2.2 Safety Objectives 39

2.3 Pharmacokinetic/Pharmacodynamic Objectives 39

2.4 Exploratory Objectives 39

3. STUDY DESIGN 40

3.1 Description of the Study 40

3.2 Rationale for Study Design 43

3.2.1 Rationale for Study Period B 43

3.2.2 Rationale for Crenezumab Dosage 44

3.2.3 Rationale for Participant Population 46

3.3 Outcome Measures 47

3.3.1 Primary Efficacy Outcome Measures 47

3.3.2 Secondary Efficacy Outcome Measures 47

3.3.2.1 Clinical 48

3.3.2.2 Biomarkers ... 48
3.3.3 Safety Outcome Measures ... 48
3.3.4 Pharmacokinetic/Pharmacodynamic Outcome Measures ... 49
3.3.5 Exploratory Outcome Measures ... 49
3.3.5.1 Clinical ... 49
3.3.5.2 Fluid Biomarkers ... 49
3.3.5.3 Imaging Biomarkers ... 49
3.3.5.4 Other Outcome Measures ... 50
3.4 Safety Plan ... 50
3.4.1 Immunogenicity ... 51
3.4.2 Amyloid-Related Imaging Abnormalities ... 51
3.4.2.1 Monitoring for Amyloid-Related Imaging Abnormalities ... 52
3.4.2.2 Management of Amyloid-Related Imaging Abnormalities ... 53
3.4.3 Pneumonia ... 54
3.4.3.1 Monitoring for Pneumonia ... 54
3.4.4 Injection and Infusion Reactions ... 55
3.4.4.1 Systemic Injection and Infusion-Related Reactions ... 55
3.4.5 Additional Safety Monitoring ... 55
3.5 Study Participants and Analysis Groups ... 56
3.6 Control Groups ... 56
3.7 Minimization of Bias ... 56
3.8 Ethical Considerations ... 57
3.9 Administrative Structure ... 58
3.10 Compliance with Laws and Regulations ... 58
4. MATERIALS AND METHODS ... 58
4.1 Participants ... 58
4.1.1 Participant Selection ... 58
4.1.2 Inclusion Criteria ... 59
4.1.3 Exclusion Criteria ... 61
4.2 Method of Treatment Assignment and Blinding ... 64

4.3 Study Treatment 65
4.3.1 Crenezumab and Placebo 65
4.3.1.1 Formulation 65
4.3.1.2 Dosage, Administration, and Storage 66
4.3.1.3 Dosage Modification 67
4.3.2 PET Tracers 68
4.4 Concomitant and Excluded Therapies 68
4.4.1 Concomitant Therapy 68
4.4.1.1 Permitted Therapies 68
4.4.1.2 Permitted Therapies under Special Circumstances (Requires Medical Monitor Approval) 69
4.4.1.3 Prohibited Therapies 69
4.5 Study Assessments 70
4.5.1 Definitions of Study Assessments 70
4.5.2 Screening and Pretreatment Assessments 77
4.5.3 Assessments During Treatment 79
4.5.4 Remote Assessments 79
4.5.5 Study Completion 79
4.5.6 Early Termination Visit 80
4.5.7 Follow-Up Assessments 81
4.5.8 Study Period B visits and Assessments 81
4.5.9 Samples for Roche and Other Clinical Repositories 81
4.5.9.1 Overview of the Biological Sample Repositories 81
4.5.9.2 Future Research 82
4.5.9.3 Sample Collection 82
4.5.9.4 Confidentiality 83
4.5.9.5 Participation in the RCR and NCRAD 83
4.5.9.6 Withdrawal from the RCR and NCRAD 84
4.5.9.7 Monitoring and Oversight 84
4.6 Participant Discontinuation 84
4.7 Study Discontinuation 85
4.8 Post-Trial Access to Study Drug 85

4.9 Assay Methods 86
4.10 Statistical Methods 86
4.10.1 Analysis of the Conduct of the Study 86
4.10.2 Analysis of Treatment Group Comparability 86
4.10.3 Efficacy Analyses 86
4.10.4 Safety Analysis 88
4.10.4.1 Adverse Events 88
4.10.4.2 Clinical Laboratory Evaluations 89
4.10.4.3 MRI Evaluations 89
4.10.4.4 Vital Signs 89
4.10.4.5 ECGs 89
4.10.4.6 Anti-Drug Antibodies 89
4.10.5 Pharmacokinetic and Pharmacodynamic Analyses 89
4.10.6 Exploratory Analyses 89
4.10.7 Handling of Missing Data 90
4.10.8 Determination of Sample Size 90
4.10.9 Interim Analysis 90
4.11 Data Quality Assurance 91

5. ASSESSMENT OF SAFETY 91
5.1 Safety Parameters and definitions 91
5.1.1 Adverse Events 92
5.1.2 Serious Adverse Event 92
5.1.3 Protocol-Defined Events of Special Interest/Non-Serious Expedited Adverse Events 93
5.2 Methods and Timing for Capturing and Assessing Safety Parameters 94
5.2.1 Adverse Event Reporting Period 94
5.2.2 Eliciting Adverse Events 94
5.2.3 Assessment of Severity and Causality of Adverse Events 94
5.3 Procedures for Recording Adverse Events 96
5.3.1 Recording Adverse Events on the eCRF 96

5.4 Expedited Reporting Requirements for Serious Adverse Events (and Protocol-Defined Events of Special Interest) 100
5.4.1 Reporting Requirements for Fatal/Life-Threatening Serious Adverse Events Related to Investigational Product 100
5.4.2 Emergency Medical Contacts 101
5.4.3 Reporting Requirements for All SAEs and Protocol-Defined Events of Special Interest 101
5.5 Type and Duration of Follow-Up of Participants after Adverse Events 101
5.6 Post-Study Adverse Events 102
5.7 Expedited Reporting to Health Authorities, Investigators, Institutional Review Boards, and Ethics Committees 102
6. INVESTIGATOR REQUIREMENTS 103
6.1 Study Initiation 103
6.2 Study Completion 103
6.3 Informed Consent Form 104
6.4 Communication with the Institutional Review Board or Ethics Committee 105
6.5 Study Monitoring Requirements 105
6.6 Electronic Case Report Forms 105
6.7 Source Data Documentation 106
6.8 Use of Computerized Systems 106
6.9 Study Medication Accountability 107
6.10 Disclosure of Data 107
6.11 Retention of Records 108
7. REFERENCES 109

LIST OF TABLES

Table 1 Adverse Event Grading (Severity) Scale 95
Table 2 Causal Attribution Guidance 96

LIST OF FIGURES

Figure 1a Study Design ... 42
Figure 1b Study Schematic: ... 42
Figure 2 Pharmacokinetic Profile Based on Modeling and Simulation Data for 60 mg/kg Crenezumab Administered Intravenously Q4W Compared with 15 mg/kg Crenezumab Administered Intravenously Q4W ... 46

LIST OF APPENDICES

Appendix A-1 (SC Dosing) Study Flowchart (Screening–Week 104 [jj]) ... 114
Appendix A-2 (SC Dosing) Study Flowchart (Week 106–End of Study(Period A) ... 119
Appendix A-3 (IV Dosing) Study Flowchart (First IV Dosing–Week 104 [bb]) ... 124
Appendix A-4 (IV Dosing) Study Flowchart (Week 108–End of Study Period A) ... 128
Appendix A-5 Study Flow Chart for Study Period B: SC dosing ... 132
Appendix A-6 Study Flow Chart for Study Period B: IV dosing ... 136
Appendix B Neurocognitive and Functional Assessments ... 140

PROTOCOL AMENDMENT ACCEPTANCE FORM

**TITLE:**	**A DOUBLE-BLIND, PLACEBO-CONTROLLED PARALLEL-GROUP STUDY IN PRECLINICAL *PSEN E280A* MUTATION CARRIERS RANDOMIZED TO CRENEZUMAB OR PLACEBO, AND IN NON-RANDOMIZED, PLACEBO-TREATED NON-CARRIERS FROM THE SAME KINDRED, TO EVALUATE THE EFFICACY AND SAFETY OF CRENEZUMAB IN THE TREATMENT OF AUTOSOMAL-DOMINANT ALZHEIMER'S DISEASE**
**PROTOCOL NUMBER:**	GN28352
**VERSION NUMBER**:	10
**STUDY DRUG:**	Crenezumab (MABT5102A)
**NCT NUMBER**	NCT01998841
**MEDICAL MONITOR:**	[redacted], M.D., M.A.S.
**SPONSOR:**	F. Hoffmann-La Roche Ltd<br>CH-4070 Basel, Switzerland<br><br>In collaboration with:<br>Banner Alzheimer's Institute<br>901 East Willetta St.<br>Phoenix, AZ 85006

I agree to conduct the study in accordance with the current protocol.

____
Principal Investigator's Name (print)

____ __________________
Principal Investigator's Signature Date

Please return a copy of the form to the contract research organization's representative.
Please retain the original for your study files.

PROTOCOL SYNOPSIS

**TITLE:**	**A DOUBLE-BLIND, PLACEBO-CONTROLLED PARALLEL-GROUP STUDY IN PRECLINICAL PSEN E280A MUTATION CARRIERS RANDOMIZED TO CRENEZUMAB OR PLACEBO, AND IN NON-RANDOMIZED, PLACEBO-TREATED NON-CARRIERS FROM THE SAME KINDRED, TO EVALUATE THE EFFICACY AND SAFETY OF CRENEZUMAB IN THE TREATMENT OF AUTOSOMAL-DOMINANT ALZHEIMER'S DISEASE**
**PROTOCOL NUMBER:**	GN28352
**VERSION NUMBER:**	10
**STUDY DRUG:**	Crenezumab (MABT5102A)
**NCT NUMBER**	NCT01998841
**PHASE:**	Phase II
**INDICATION:**	Alzheimer's Disease
**SPONSOR:**	F. Hoffmann-La Roche Ltd<br>CH-4070 Basel, Switzerland<br><br>In collaboration with:<br>Banner Alzheimer's Institute<br>901 East Willetta St.<br>Phoenix, AZ 85006

Objectives

Primary Efficacy Objective

The primary objective*s* of this Phase II trial *are*

1. to evaluate the efficacy of crenezumab treatment compared with placebo for at least 260 weeks on change in cognitive function *as measured by the API ADAD Cognitive Composite Test Battery* in preclinical *presenilin 1 (PSEN1) E280A* autosomal dominant mutation carriers.
2. *to evaluate the efficacy of crenezumab treatment compared with placebo for at least 260 weeks on change in episodic memory function as measured by the FCSRT Cueing Index in preclinical PSEN1 E280A autosomal-dominant mutation carriers.*

Secondary Efficacy Objectives

The secondary efficacy objectives of this study (comparing crenezumab with placebo) will be to evaluate the following in *PSEN1 E280A* mutation carriers:

- Ability of crenezumab to affect clinical endpoints other than *those in* the primary *endpoin* family*:* Alzheimer's Prevention Initiative (API) Autosomal-Dominant Alzheimer's disease (ADAD) Cognitive Composite Test Battery *and the FCSRT Cueing Index.*
- Ability of crenezumab to reduce cerebral fibrillar amyloid burden in a predefined region of interest (ROI) using florbetapir positron emission tomography (PET)
- Ability of crenezumab to reduce decline in regional cerebral metabolic rate of glucose (CMRgl) using fluorodeoxyglucose (FDG)-PET measurements in a ROI

- Ability of crenezumab to reduce brain atrophy as measured by volumetric measurements using magnetic resonance imaging (MRI)
- Ability of crenezumab to affect a tau based cerebrospinal fluid (CSF) biomarker

Safety Objectives

The safety objectives for this study are as follows:

- To assess the safety and tolerability of crenezumab in preclinical *PSEN1 E280A* mutation carriers (comparing crenezumab with placebo)

Pharmacokinetic/Pharmacodynamic Objectives

The pharmacokinetic (PK) and pharmacodynamic (PD) objectives for this study are as follows:

- Collect sparse PK samples to support confirmation of exposure to crenezumab and to explore the pharmacodynamic (PD) response (as measured by plasma total Aβ levels comparing crenezumab with placebo) in preclinical *PSEN1 E280A* mutation carriers

Exploratory Objectives

The exploratory objectives for this study are as follows:

- Assess further the effect of crenezumab in preclinical *PSEN1 E280A* mutation carriers on additional clinical measures of efficacy and biological markers of disease that have not been prespecified as primary *or* secondary endpoints in the Statistical Analysis Plan (SAP)
- Explore pharmacogenetic effects including, but not limited to, a person's apolipoprotein E (*APOE*) ε4 carrier status on the active treatment's cognitive, clinical, and adverse effects
- Explore effects of genetic variation, including, but not limited to, how genes affect the biology of Alzheimer's disease (AD) and other diseases and how genes influence biomarker responses
- Examine clinical and biomarker changes in non-carriers and to compare these changes with those seen in carriers treated with placebo
- Relate the treatment's biomarker effects to clinical outcomes and to examine predictive and prognostic utility of baseline characteristics
- Assess the impact of treatment on brain tau load over time, as measured by tau PET imaging in an optional substudy (GN28352-1/BN40199)

Study Design

Description of the Study

Study Period A: This is a prospective, randomized, double-blind, placebo-controlled, parallel-group study of crenezumab versus placebo in individuals who carry the *PSEN1 E280A* autosomal-dominant, mutation-causing early-onset AD and do not meet criteria for mild cognitive impairment (MCI) due to AD or dementia due to AD and are, thus, in a preclinical phase of AD. The study also incorporates administration of placebo to individuals who are not *PSEN1 E280A* autosomal-dominant mutation carriers. This efficacy and safety study will be conducted at a single primary site and approximately three satellite sites in Colombia.

In the Colombian study, *PSEN1 E280A* autosomal-dominant mutation carriers who meet study eligibility criteria will be randomized in a 1:1 ratio to one of two treatment groups: crenezumab or placebo. Crenezumab will be administered either subcutaneously (720 mg every 2 weeks) or intravenously (60 mg/kg every 4 weeks). Matching placebo will be administered by the same routes at the same frequencies. The switch to the higher IV dose (approximately 4-fold higher exposure to crenezumab) will be optional. Participants may decide whether to change from the current SC dosing to the IV dose; however, once IV dosing in a given participant has occurred, it is not intended for a participant to switch back to the lower SC dose. In order to maintain genotype blind and to have a genetic kindred control, a cohort of *PSEN1 E280A* mutation non-carrier kindred family members will also be enrolled in the study and dosed only with placebo.

The study includes three arms with approximately 100 participants per arm; two arms of participants with a *PSEN1 E280A* mutation randomized in a 1:1 ratio to active or placebo and a

third arm of non-carriers randomized to placebo. Participants will receive the randomized treatment until the final participant has reached their final treatment visit at 260 weeks.

The study duration for individual participants will be at least approximately 284 weeks, including an 8-week screening period, a double-blind treatment period of at least 260 weeks in length, 4-week final dose visit, and a final safety follow-up visit 16 weeks after the last dose of study drug (crenezumab or placebo) that will allow for clinical follow-up after treatment discontinuation for participants who do not to continue with study drug beyond the end of Study Period A or who terminate study drug early. One or more interim analyses may be performed at the Sponsor's discretion.

Study Period B: Following the completion of Study Period A, participants will be offered the opportunity to continue to receive study drug until the results of the study are known and post trial access to crenezumab is started (via an open label extension[OLE] or other program) or development of crenezumab is discontinued. This is termed Study Period B. All PSEN1 mutation carriers will be provided crenezumab in Study Period B regardless of treatment allocation during Study Period A and all non-carriers will continue on placebo. Treatment allocation for both study periods A and B will remain blinded. Study Period B may last up to approximately 9-12 months depending on when participants enter Study Period B.

Outcome Measures

Primary Efficacy Outcome Measures

The primary efficacy outcome measure*s* for this study *are* as follows:

- Annualized rate of change on the API ADAD Composite Cognitive Test total score, which is computed from the following five cognitive test scores:
  - Word List: Recall
  - Multilingual Naming Test
  - Mini-Mental State Examination (MMSE) (for orientation to time)
  - Consortium to Establish a Registry for Alzheimer's Disease (CERAD) Constructional Praxis (a measure of visuospatial ability)
  - Raven's Progressive Matrices, (a measure of nonverbal fluid reasoning and visuospatial abilities), *Set A*
- *Annualized rate of change on the FCSRT Cueing Index*

Secondary Efficacy Outcome Measure

The final selection of secondary outcome measures will be made on the basis of baseline data from the study population, relevant completed trials, and other external data that will emerge prior to database unblinding. The selected outcomes will be provided in the SAP to be submitted prior to database unblinding. Secondary outcome measures will be selected from the following.

Clinical

- Time to progression from preclinical AD to MCI due to AD or from preclinical AD to dementia due to AD
- Time to progression to non-zero in the Clinical Dementia Rating (CDR) Scale global score
- Annualized rate of change in the CDR Scale–Sum of Boxes
- Annualized rate of change in a measure of overall neurocognitive functioning: Repeatable Battery for the Assessment of Neuropsychological Status (RBANS)

Biomarkers

- Imaging biomarkers
  - Annualized rate of change in mean cerebral fibrillar amyloid accumulation using florbetapir PET from a predefined ROI
  - Annualized rate of change in regional cerebral metabolic rate of glucose (CMRgl) using FDG-PET in a predefined ROI
  - Annualized rate of change in volumetric measurements using MRI
- CSF biomarkers

– Annualized rate of change in a tau-based CSF biomarker

Safety Outcome Measures

Safety will be assessed through regular neurological and physical examinations, prospective suicidality assessment (copyright Pfizer Inc. and Janssen Alzheimer Immunotherapy; used with permission), laboratory tests, electrocardiograms (ECGs), and MRI assessments. In addition, the following data will be collected and analyzed:

- Frequency and severity of treatment-emergent adverse events (AEs) and serious adverse events (SAEs)
- Withdrawals due to AEs
- *Incidence of treatment-emergent a*myloid-related imaging abnormalities–edema/effusion (ARIA-E) (cerebral vasogenic edema [VE], sulcal effusion), amyloid-related imaging abnormalities–hemosiderin deposition (ARIA-H) (superficial central nervous system [CNS] siderosis, cerebral microhemorrhages), cerebral macrohemorrhages, and pneumonia.
- Incidence of injection reactions and infusion-related reactions (IRRs)
- Incidence of anti-crenezumab antibodies

Pharmacokinetic/Pharmacodynamic Outcome Measures

The PK outcome measures are CSF and serum crenezumab concentrations at protocol-specified timepoints. In serum, trough serum crenezumab concentrations at steady state ($C_{trough, ss}$), will be assessed. Additional PK analyses may be conducted, as appropriate.

The PD outcome measures are as follows: Plasma Aβ1-40 and Aβ1-42 concentrations.

Exploratory Outcome Measures

Exploratory outcome measures are listed in Sections 3.3.5.1 –3.3.5.3 of the protocol.
In addition, any endpoints that are not prespecified in the SAP as secondary outcome measures may be analyzed as exploratory outcome measures.

<u>Safety Plan</u>

See Assessment of Safety in the protocol for complete details of the safety evaluation for this study.

Crenezumab has not been approved for use by global health authorities and is currently in clinical development. Thus, the entire safety profile is not known at this time. The crenezumab safety information is based on results from nonclinical and clinical studies, as well as published data on similar molecules. The safety monitoring for this study is designed to ensure participant safety and will include specific eligibility criteria and monitoring assessments. Any AEs/SAEs or any other abnormalities (e.g., laboratory assessments, radiographs, physical examination findings) should be managed by the site investigators, as medically appropriate (unless otherwise specified in the protocol).

The safety of participants in this trial will be ensured through the use of stringent inclusion and exclusion criteria and through close monitoring of participants. Investigators will assess the occurrence of AEs and SAEs at all participant evaluation timepoints during the study. All AEs and SAEs, whether volunteered by the participant, discovered by study personnel during questioning, or detected through physical examination, laboratory tests, or other means, will be recorded in the participant's medical record and on the appropriate AE or SAE electronic Case Report Form (eCRF).

Each recorded AE or SAE will include a description of its duration (i.e., start and end dates), severity, seriousness according to regulatory criteria, if applicable, and suspected relationship to the investigational product, as well as any actions taken.

Participants will be followed carefully for AEs during the study, including a 4-week postdose visit and a final safety follow-up visit 16 weeks after the last dose of study drug. AEs will be graded according to severity using the National Cancer Institute Common Terminology for Adverse Events, Version 4.0.

An Independent Data Monitoring Committee (iDMC) formerly known as the Data and Safety Monitoring Board (DSMB) will be used for this study.

Please refer to the latest Crenezumab Investigator's Brochure for additional safety information.

Immunogenicity

Immunogenicity, as determined by the presence of positive response in the ADA enzyme-linked immunosorbent assay (ELISA), will be assessed at specified timepoints during the course of the study. Additional characterizations will be performed if necessary.

Amyloid-Related Imaging Abnormalities

Amyloid-related imaging abnormalities are a spectrum of image abnormalities detected on MRI. Two types of ARIA have been described: ARIA-E refers to the MRI alterations thought to represent vasogenic edema and related extravasated fluid phenomena, and ARIA-H refers to the MRI alterations attributable to microhemorrhages and leptomeningeal hemosiderosis.

The occurrence of imaging abnormalities believed to represent cerebral VE has been reported in association with the investigational use of compounds that are intended to treat AD by reducing Aβ in the brain. These imaging abnormalities (Salloway et al. 2009; Sperling et al. 2011a) have, in the majority of instances, been asymptomatic, and their presence has been detected by brain MRI. Symptoms, when present in association with such imaging abnormalities, have been reported to include headache, worsening cognitive function, alteration of consciousness, seizures, unsteadiness, and vomiting.

The clinical significance of ARIA-H is not yet fully understood.

When anti-Aβ antibodies bind to amyloid deposited around blood vessels, an FcγR-mediated immune response may be elicited, compromising vascular integrity and resulting in ARIA-E or ARIA-H. Crenezumab, a human IgG4, has reduced FcγR binding compared with IgG1/2 and, thus, has reduced effector function that theoretically might lower the risk of ARIA-E. This has been supported by emerging safety data observed in both clinical and nonclinical studies conducted to date. A lack of binding to vascular amyloid, noted following in vivo dosing in PS2APP transgenic mice (Study 15-2817B), may additionally reduce the risk of ARIA.

To minimize the potential safety risks associated with ARIA-E and ARIA-H in this study, participants will be screened for ARIA-E and ARIA-H before enrollment and will be monitored for ARIA-E and ARIA-H by MRI during treatment. Given the uncertainty of risk and concerns about cerebral amyloid angiopathy (CAA) severity, participants with superficial siderosis of the CNS, more than four cerebral microhemorrhages, or evidence of a prior cerebral macrohemorrhage at screening will be excluded from this study.

An overview of the monitoring and management guidelines for ARIA-E and ARIA-H is provided in the following sections.

The cerebral microhemorrhage thresholds for study entry and treatment discontinuation allow for imaging measurement variability to be taken into account and reflect the uncertainty regarding the clinical relevance of small numbers of cerebral microhemorrhages. This approach is intended to minimize potential safety risks associated with cerebral microhemorrhages but would not preclude participants from receiving potentially beneficial treatment.

Monitoring for Amyloid-Related Imaging Abnormalities

In this Phase II study, a safety monitoring plan has been designed to mitigate and monitor the potential risk of ARIA-E and ARIA-H associated with anti-Aβ immunotherapy. This plan consists of the following key elements:

- Participants with evidence of one or more of the following abnormalities assessed by MRI during screening will be excluded from the trial:
  - Significant cerebral vascular pathology
  - Superficial siderosis of the CNS
  - More than four cerebral microhemorrhages
  - Evidence of a prior cerebral macrohemorrhage
- Participants enrolled in the study will regularly undergo neurological examinations to evaluate for any neurologic signs or symptoms that are suggestive of the presence of the following:

  Cerebral VE (e.g., confusion, dizziness, vomiting, and lethargy), superficial siderosis of the CNS (e.g., sensorineural deafness, cerebellar ataxia, and pyramidal signs), or

cerebral microhemorrhages (e.g., transient or permanent focal motor or sensory impairment, ataxia, aphasia, and dysarthria)

- Brain MRI exams (including fluid-attenuated inversion recovery and T2*-weighted gradient-recalled echo sequences will be performed for safety monitoring approximately every 3 months for the first year and approximately every 6 months thereafter to detect potential safety risks noted below. If switching to 60 mg/kg IV Q4W, MRI scans will be performed at 3 months and 6 months after initiating the higher dose and then every 24–28 weeks thereafter to detect the following: ARIA-E, ARIA-H, and cerebral macrohemorrhages
- All safety MRI scans will be read for the evaluation of ARIA-E, ARIA-H, and cerebral macrohemorrhages by independent radiologists at a central reading facility.
- Regardless of severity, all events of cerebral VE, sulcal effusion, superficial siderosis of the CNS, and cerebral micro- or macro-hemorrhage that occur at any time after receiving study drug are considered to be AESI and will be reported in an expedited manner.

Management of Amyloid-Related Imaging Abnormalities

Amyloid-Related Imaging Abnormalities–Edema/Effusion (ARIA-E)

The following rules for the management of MRI-related findings apply for ARIA-E.

- Asymptomatic ARIA-E with size $<$ 4 Barkhof Grand Total Score (BGTS)
  - Continue study drug at the same dose (i.e., 60 mg/kg crenezumab).
  - Repeat MRI 4 weeks later.
  - If ARIA-E is stable or decreased, continue study drug and monthly MRI monitoring until event resolves. Once ARIA-E completely resolves, conduct MRI monitoring as per study flowchart.
  - If ARIA-E increases (BGTS $\geq$ 4) or symptoms develop, refer to the rules below.
- Symptomatic ARIA-E (of any size) or asymptomatic ARIA-E with size $\geq$ 4 BGTS:
  - Temporarily interrupt study drug and implement monthly MRI monitoring.
  - Once symptoms and ARIA-E resolve, reintroduce study drug and perform an MRI after 4 weeks of dosing. If no new MRI abnormality is detected, resume MRI monitoring as per the study flowchart.

For any new onset of ARIA-E:

- Treat the same as the first event, on the basis of symptoms and BGTS.
- If a participant is diagnosed with three recurrent, symptomatic ARIA-E events or exacerbations of previous events, permanently discontinue the study drug.
- Implement monthly MRI monitoring until resolution of both symptoms and ARIA-E.
- As per the protocol, maintain the participant in the study until study end and perform assessments as per the study flowcharts.

Amyloid-Related Imaging Abnormalities–Hemosiderin Deposition (ARIA-H)

The following dose adjustments and discontinuation rules for MRI-related findings will apply to ARIA-H:

- Dose reduction: Participants who develop $>$ 8 ARIAH-H deposition cumulatively will receive a lower dose of the study drug. Cumulative is the sum of ARIA-H at baseline and newly detected ARIA-H during the study.
- Study drug discontinuation: Participants who develop $>$ 10 ARIA-H cumulatively will be permanently discontinued from the study drug. Cumulative is the sum of ARIA-H at baseline and newly detected ARIA-H during the study.

The Sponsor believes that the above rules, coupled with frequent MRI monitoring and collection of CNS symptoms, will ensure safety while trying to maximize benefit for the participants.

Pneumonia

In the RPCP of Phase II clinical trials in mild to moderate AD (ABE4869g and ABE4955g) there was an imbalance in the rates of TLRTI (including preferred terms of pneumonia, LRTI, and bronchitis) driven by pneumonia. However, this imbalance was not replicated in the pooled data of the two large Phase III Studies (BN29552 and BN29553) where the pneumonia cases were balanced between the active treatment and placebo arms (13 and 15 cases).

There is no clinical evidence of immunosuppression (e.g., effects on white blood cells or neutrophils; or pattern of infection type, e.g., opportunistic infections). In addition, no evidence of immunosuppression or of a lung toxicity effect has been observed in preclinical studies.

Based on the currently available data, a causal relationship between the drug and events of pneumonia cannot be established or ruled out. Pneumonia is considered a potential risk to be monitored closely, and is added to this protocol as an AESI.

Monitoring for Pneumonia

It is of paramount importance to carefully document any pneumonia cases and other serious respiratory infections that occur in this study, by means of providing all relevant information required by the eCRF.

Participants with signs and/or symptoms suggestive of pneumonia (e.g., cough, fever, pleuritic chest pain) should undergo chest radiography for confirmation of diagnosis. Participants should be treated as per standard treatment care and any change to study drug should be considered within a benefit–risk assessment frame, and in agreement with the Medical Monitor.

- A chest X-ray is required under the following circumstances:
  - Serious and non-serious pneumonia
  - Serious and non-serious lower respiratory tract infections
  - Serious upper respiratory tract infections

In addition, whenever possible, additional relevant investigations should be conducted (e.g., white blood cell (WBC) counts, pathogen identification by means of hemocultures, bronchoalveolar lavage).

Injection and Infusion Reactions

Administration of monoclonal antibodies may be associated with local reactions at the site of injection or infusion. Systemic injection/infusion reactions as well as hypersensitivity or anaphylactic reactions may also occur.

Infusion and injection-related reactions are defined as any AE that occurs during or within 24 hours after the study drug injection of infusion.

No relationship between treatment dose and the occurrence of infusion/injection related reactions has been observed. The majority of infusion-related reactions in all ongoing and completed studies with crenezumab were of mild to moderate severity.

Monitoring and Management of Systemic Injection and Infusion-Related Reactions

Crenezumab will be administered to participants under close medical supervision in a setting with access to appropriate emergency equipment and staff who are trained to monitor and respond to medical emergencies.

In the event that a participant experiences a mild IRR, the infusion will be halted. Once the reaction has resolved, the infusion rate will be resumed at half of the most recently used rate (e.g., 3–1.5 mL/min).

Participants who experience a moderate IRR (e.g., fever or chills) should have the infusion stopped immediately and should receive aggressive symptomatic treatment. The infusion should not be restarted before all symptoms have disappeared, and then it should be restarted at half the initial rate.

Participants who experience serious or severe hypersensitivity reactions (e.g., hypotension, mucosal involvement) should not receive additional study drug. In addition, in any case of anaphylaxis, anaphylactoid reaction, serious or severe hypersensitivity reaction, ADA and concurrent PK samples should be collected as close as possible to the event, and then at 4 and 16 weeks post-dose (see study flowcharts for further details). Participants who receive the SC formulation of study drug will be observed for a minimum of 15 minutes following all injections.

Participants who receive IV formulation of study drug, will be observed for a minimum of 60 minutes following all infusions.

Additional Safety Monitoring

Laboratory tests, including hematology, chemistry, coagulation, urinalysis, and ADAs will be performed throughout the study. ECGs will be recorded regularly, including at baseline. Physical and neurological examinations will be performed throughout the study to assess AEs, particularly any neurological events that are not consistent with normal disease progression. Suicidality assessments will be used to measure prospective suicidality. If the suicidality assessment reveals a concern about a significant level of suicidality, the participant will undergo a more detailed assessment by a qualified clinician who has experience in the evaluation of suicidal ideation and behavior, either at the site or by referral to an outside clinician.

Serum pregnancy testing will be performed at screening for all women of childbearing potential. Urine pregnancy testing will be performed prior to study drug administration or PET scan for all women of childbearing potential. Pregnancy testing will apply to all women of childbearing potential unless documented (by medical records or physician's note) to be surgically sterile or postmenopausal, as defined in International Council for Harmonisation (ICH) Guidance M3 (R2), Section 11.2 (12 months with no menses without an alternative medical cause). If urine pregnancy testing returns a positive result, study drug will be held until pregnancy is verified. If pregnancy is confirmed, the participant will not receive further doses of study drug.

The incidence and nature of AEs, SAEs, and laboratory abnormalities will be assessed on a regular basis by the iDMC. The committee will recommend changes to study conduct or study termination, as required. The responsibilities and operation of the committee will be documented in the iDMC Charter.

Number of Subjects

Up to 300 people will take part in this study in Colombia.

Target Population

Inclusion Criteria

Participants must meet the following criteria for study entry:

1. Signed and dated written informed consents obtained from the participant and study partner in accordance with local regulations prior to the initiation of any protocol required procedures
2. Membership in *PSEN1 E280*A mutation carrier kindred
3. Agrees to conditions of, and is willing to undergo, genetic testing (e.g., *APOE, PSEN1 E280A*, and other genetic testing)
4. *PSEN1 E280A* mutation carrier or non-carrier status has been confirmed prior to or during the screening period
5. Males and females, age ≥ 30 years and ≤ 60 years
6. MMSE of ≥ 24 for participants with less than 9 years of education or MMSE of ≥ 26 for participants with 9 or more years of education
7. Does not meet criteria for dementia due to AD
8. Does not meet criteria for MCI due to AD as defined by the following:
    - Cognitive concern in the judgment of the investigator, based in part on the average Subjective Memory Checklist score > 22 (average of participant and study partner component scores)
    - Word List: Recall < 3 for participants with less than 9 years of education
    - Word List: Recall < 5 for participants with 9 or more years of education
    - Preservation of independence in functional activities in the judgment of the investigator, based in part on review of the Functional assessment staging (FAST)
9. Adequate vision and hearing in the investigator's judgment to be able to complete testing
10. If female, and not documented (by medical records or physician's note) to be surgically sterile (absence of ovaries and/or uterus or tubal ligation) or postmenopausal, willing to undergo pregnancy tests at protocol-specific timepoints

11. For women who are not documented (by medical records or physician's note) to be surgically sterile (absence of ovaries and/or uterus or tubal ligation) or postmenopausal, agreement to remain abstinent or use two adequate methods of contraception, including at least one method with a failure rate of < 1% per year (e.g., hormonal implants, combined oral contraceptives, vasectomized partner) during the treatment period and for at least 16 weeks (approximately 5 half-lives) after the last dose of study drug
12. For men of reproductive potential with partners of childbearing potential (i.e., women who are not surgically sterile and are not postmenopausal agreement to remain abstinent or use a condom as a method of contraception during the treatment period and for at least 8 weeks (approximately 2 half-lives) after the last dose of study drug

    For male participants, a documented successful vasectomy (by medical records or physician documentation) is an acceptable alternative form of birth control.
13. Study partner who agrees to participate in the study and is capable of and willing to:
    - Accompany the participant to all required visits as specified in the footnotes to the study flowcharts provided in Appendix A-1 and Appendix A-2 for SC administration and Appendix A-3 and Appendix A-4 for IV administration.
    - Provide information for required telephone assessments
    - Spend sufficient time with the participant to be familiar with his/her overall function and behavior, and be able to provide adequate information about the participant including:
        - Knowledge about domestic activities, hobbies, routines, social skills and basic activities of daily life
        - Work and educational history
        - Cognitive performance, including memory abilities, language abilities, temporal and spatial orientation, judgment, and problem solving
        - Emotional and psychological state
        - General health status
14. Participant and study partner have evidence of the following:
    - Adequate premorbid functioning (e.g., intellectual, visual, and auditory)
    - Fluency in, and able to read, the language in which study assessments are administered
15. Willing and able to undergo neuroimaging (PET and MRI)
16. In the judgment of the investigator, no clinically significant thyroid dysfunction or B12 deficiency, as determined by the following criteria:
    - Serum thyroid stimulating hormone (TSH) and B12 levels within normal or expected ranges for the testing laboratory or, if TSH or B12 values are out of range, they are judged by the investigator not to be clinically significant.
    - If the participant is undergoing thyroid replacement therapy, TSH levels must either be within normal or expected ranges for the testing laboratory or, if TSH values are out of range, they do not require any therapeutic actions (treatment or surveillance).
    - If the participant is receiving vitamin B12 injections or oral vitamin B12 therapy, B12 levels must be at or above the lower limit of normal for the testing laboratory or, if B12 values are out of range, they do not require any therapeutic actions (treatment or surveillance).
17. In good general health with no known co-morbidities expected to interfere with participation in the study
18. In the opinion of the investigator, the participant and study partner will be in accordance with the following:
    - Able to comply with the protocol
    - Able to participate in all scheduled evaluations
    - Have a high probability of completing all required tests and the study

Exclusion Criteria

Participants who meet any of the following criteria will be excluded from study entry:

1. Significant medical, psychiatric, or neurological condition or disorder documented by history, physical, neurological, laboratory, or ECG examination that would place the participant at undue risk in the investigator's judgment or impact the interpretation of efficacy
2. History of stroke
    - Participants with a history of transient ischemic attack may be enrolled if the event occurred ≥ 2 years prior to screening.
3. History of severe, clinically significant (persistent neurological deficit or structural brain damage) CNS trauma (e.g., cerebral contusion)
4. Body weight < 45 or > 120 kg
5. History or presence of atrial fibrillation that poses a risk for future stroke in the investigator's judgment
6. Clinically significant laboratory or ECG abnormalities (e.g., abnormally prolonged or shortened QTc interval) in the investigator's judgment
7. Current presence of bipolar disorder or other clinically significant major psychiatric disorder according to DSM-IV-TR or symptom (e.g., hallucinations, agitation, paranoia) that could affect the participant's ability to complete evaluations
    - Recorded as an AE if diagnosed after study enrollment
8. Clinically significant depression, based in part by a Geriatric Depression Scale (short form GDS) (15-point scale) score > 9 at screening
9. History of seizures (excluding febrile seizures of childhood, or other isolated seizure episodes that were not due to epilepsy in the judgment of the investigator, and required at most time-limited anticonvulsant treatment, and which occurred more than 7 years prior to the screening visit)
10. Myocardial infarction within 2 years, congestive heart failure, atrial fibrillation, or uncontrolled hypertension
11. Women who are pregnant or intend to become pregnant during the conduct of this trial
12. Women who are nursing infants or intend to nurse infants during the conduct of this trial
13. History of cancer within 5 years with the exception of basal cell carcinoma, squamous cell cancer of the skin that has been previously excised with clean margins, or any cancer that is not actively being treated with anti-cancer drugs or radiotherapy, as well as cancers that are considered to have a low probability of recurrence (with supporting documentation of this from the treating oncologist if possible)
14. Clinically significant infection within the last 30 days prior to screening (e.g., chronic, persistent, or acute infection [e.g., upper respiratory infection, urinary tract infection])
15. Brain MRI imaging results at baseline showing any of the following:
    - Evidence of any ARIA-E (cerebral VE, sulcal effusion), infection, significant cerebral vascular pathology, clinically significant lacunar infarct in a region important for cognition or multiple lacunes or a cortical infarct or focal lesions of clinical significance
    - More than four cerebral microhemorrhages (lesions with diameter ≤ 10 mm), regardless of their anatomical location or diagnostic characterization as "possible" or "definite"
    - Single area of superficial siderosis of the CNS or evidence of a prior cerebral macrohemorrhage (lesion with diameter > 0 mm)
16. Clinically significant screening blood or urine laboratory abnormalities requiring further evaluation or treatment, including the following:
    - Impaired hepatic function, as indicated by transaminases $> 2 \times$ the upper limit of normal (ULN) or abnormalities in synthetic function tests judged by the investigator to be clinically significant
    - Impaired coagulation (aPTT $> 1.2 \times$ ULN)

- Platelet count < 100,000/μL
- Glycosylated hemoglobin > 8.0%

17. Positive urine test for drugs of abuse at screening
    - One additional screening may be performed; a positive test (except for cannabinoids) will result in exclusion. Results of cannabinoid assays will not be used for the determination of eligibility.
18. History of alcohol or substance dependence within the previous two years (DSM-IV TR criteria)
19. Use of any other medications with the potential to significantly affect cognition (including, but not limited to, sedatives, narcotics [e.g., opiates/opioids], hypnotics, over-the-counter [OTC] sleeping aids, sedating anti-allergy medications)

    Intermittent or short-term use of these medications may be allowed if deemed medically necessary for the treatment of a non-excluded medical condition with approval from the Medical Monitor. In addition, use of tricyclic antidepressants or benzodiazepines will be permitted if used in stable, low doses for the treatment of a non-excluded medical condition with approval from the Medical Monitor.
20. Use of typical anti-psychotics or barbiturates
21. Use of non–anti-cholinergic antidepressant medications or atypical anti-psychotics unless maintained on a stable dose regimen for at least 6 weeks prior to screening
22. Use of any FDA/INVIMA-approved medications for treatment of late-onset AD at screening/baseline

    Cholinesterase inhibitors and/or memantine are prohibited during the study except in participants enrolled in the study who develop AD dementia. These participants will be permitted to take approved AD treatments (cholinesterase inhibitors and/or memantine).
23. Use of anti-coagulant medication (e.g., heparanoids, heparin, warfarin, thrombin inhibitors, Factor Xa inhibitors), or known coagulopathy or platelet count < 100,000 cells/μL within 4 weeks of the screening visit

    Documentation required for normal PT, PTT, and INR during screening for discontinued anti-coagulants

    Anti-platelet medications (e.g., aspirin, clopidigrel, dipyridamole) are permitted if on a stable dose for 4 or more weeks prior to screening.

    Short-term, peri-operative use of anti-coagulants may not result in discontinuation from the study; however, any such use must be discussed with the Medical Monitor.
24. Treatment with any biologic therapy within five half-lives or 3 months prior to screening, whichever is longer, with the exception of routinely recommended vaccinations, which are allowed
25. Use of anti-seizure medication (except in childhood for febrile seizures or if used for non-seizure indications), anti-Parkinsonian, or stimulant (e.g., methylphenidate) medications
26. Use of investigational drug, device, or experimental medication within 60 days (or five half-lives, whichever is longer) of the screening visit
27. Previous treatment with crenezumab (MABT5102A) or any other therapeutic that targets Aβ
28. History of severe allergic, anaphylactic, or other hypersensitivity reactions to chimeric, human, or humanized antibodies or fusion proteins
29. Contraindication to MRI scan procedures, possibly including, but not limited to, the following:
    - Pacemakers, implantable cardioverter defibrillators, cochlear implants, cerebral aneurysm clips, implanted infusion pumps, implanted nerve stimulators, metallic splinters in the eye, other magnetic, electronic, or mechanical implants; metal fragments or foreign objects in the skin or body
    - Clinically significant claustrophobia that would contraindicate a brain MRI scan

- Clinical history or examination finding that, in the judgment of the investigator, would pose a potential hazard in combination with MRI

30. Contraindication to PET scan procedures, possibly including, but not limited to, the following:
    - Current or recent (within 12 months prior to screening) participation in studies involving radioactive agents, such that the total research-related radiation dose to the participant in any given year would exceed the limits set forth in the U.S. Code of Federal Regulations Title 21 Section 361.1 (http://www.accessdata.fda.gov/scripts/cdrh/cfdocs/cfcfr/CFRSearch.cfm?fr=361.1)
31. Abnormal findings that, in the opinion of the investigator, may affect the participant's response to the radiopharmaceutical and related testing procedures required for PET scans
32. Unable to read and write in Spanish
33. Any other clinical contraindications for safety or participation in lumbar punctures (optional) at the clinical judgment of the investigator

Statistical Methods

Primary Analysis

Primary efficacy analysis *for the primary endpoint* family in Study Period A will be based on a modified intent-to-treat (mITT) population. The mITT population is defined as participants who are mutation carriers treated with any amount of study drug. Participants in the efficacy analysis population will be grouped based on treatment assigned at randomization.

If the Sponsor decides to initiate an OLE study, efficacy data from Study Period B could be used in the OLE study to contribute to delayed start analyses *and time-to-event analyses.*

Primary Efficacy Endpoint Family

The primary efficacy analysis will be performed on the mITT population. The primary endpoint family*, 1)* annualized rate of change on the API ADAD Composite Cognitive Test *Battery* total score *and 2) annualized rate of change on the FCSRT Cueing Index*, will be assessed using a mixed effects model. The model will be used to test the treatment effect on annualized rate of change adjusting for selected baseline characteristics and randomization factors. Statistical tests for the primary endpoint*s* will be two sided and performed at *an overall* 0.05 level*: α=0.04 for testing the treatment effect on the annualized rate of change on the API ADAD Composite Cognitive Test Battery total score; alpha=0.01 for testing the treatment effect on the annualized rate of change on the FCSRT Cueing Index*. Estimated treatment effects and confidence intervals will also be provided as an aid to interpretation of study results.

In order to maximize the power, data beyond each participant's Week 260 assessment through the unblinding of the study will be included in the primary analysis or its sensitivity analysis.

All details of the prespecified statistical analyses for the primary efficacy endpoint*s* will be provided in the SAP.

Determination of Sample Size

This study will enroll up to 300 participants: up to 200 participants will be enrolled into the carrier cohort and up to 100 participants will be enrolled into the non-carrier cohort.

Participants in the carrier cohort will be randomized in a 1:1 randomization ratio of active to placebo participants. The study *was originally* powered to compare the mean change from baseline over 260 weeks in the API ADAD Composite Cognitive Test total score between the active group and the placebo. Assuming a 25% dropout rate, two-sided testing at an overall 0.05 level, a placebo group CV of 65% for the week 260 change scores (= 100% × standard deviation of placebo participant change scores/mean of placebo participant change scores) and 100 participants per arm, the study will have at least 80% power to detect a true effect of 30% reduction of the mean decline in the placebo group.

Participants in the non-carrier cohort will all receive placebo and will be included in the study in order to maintain the genotype blind.

Interim Analysis

To adapt to information that may emerge during the course of this study, the Sponsor may choose to conduct one or more interim analyses. Sites are to be informed prior to an interim analysis. Below are the specifications to ensure the study continues to meet the highest standards of integrity when an optional interim analysis is executed.

If an interim analysis is conducted, the Sponsor will remain blinded. The interim analysis will be conducted by an external statistical group and reviewed by the iDMC. Interactions between the iDMC and Sponsor will be carried out as specified in the iDMC Charter.

The decision to conduct the optional interim analysis, along with the rationale, timing, and statistical details for the analysis, will be documented in the SAP. The iDMC Charter will be updated to document potential recommendations the iDMC can make to the Sponsor as a result of the analysis (e.g., stop the study for positive efficacy, stop the study for futility), and the iDMC Charter will also be made available to relevant health authorities.

If there is a potential for the study to be stopped for positive efficacy as a result of the interim analysis, the type I error rate of the primary analysis will be controlled to ensure statistical validity is maintained. If the study continues beyond the interim analysis, the critical value at the final analysis would be adjusted accordingly to maintain the protocol-specified overall type I error rate, per standard Lan-DeMets methodology.

LIST OF ABBREVIATIONS AND DEFINITION OF TERMS

Abbreviation	Definition
Aβ	β-amyloid
AChE	acetylcholinesterase
AD	Alzheimer's disease
ADA	anti-drug antibody
ADAD	autosomal-dominant Alzheimer's disease
ADLs	activities of daily living
AE	adverse event
AESI	adverse event of special interest
API	Alzheimer's Prevention Initiative
APOE	apolipoprotein E
APP	amyloid precursor protein
ALT	alanine transaminase
ARIA	amyloid-related imaging abnormalities
ARIA-E	amyloid-related imaging abnormalities–edema/effusion
ARIA-H	amyloid-related imaging abnormalities–hemosiderin deposition
AST	aspartate aminotransferase
AUC	area under the concentration–time curve
β-hCG	β–human chorionic gonadotropin
BGTS	Barkhof Grand Total Score
BP	blood pressure
CAA	cerebral amyloid angiopathy
CDR	Clinical Dementia Rating
CDR-SOB	Clinical Dementia Rating–Sum of Boxes
CERAD	Consortium to Establish a Registry for Alzheimer's Disease
C_{max}	maximum concentration
CMRgl	cerebral metabolic rate for glucose
CNS	central nervous system
$C_{peak, ss}$	peak concentration at steady state
CREAD	Crenezumab for Early Alzheimer's Disease
CRF	Case Report Form
CRO	contract research organization
CSF	cerebrospinal fluid
CTCAE	Common Terminology Criteria for Adverse Events
$C_{trough, ss}$	trough concentration at steady state
CV	coefficient of variation
DANA	D265A and N297A (mutations)

Abbreviation	Definition
DLT	dose-limiting toxicity
DSMB	Data and Safety Monitoring Board
DSM-IV	Diagnostic and Statistical Manual of Mental Disorders IV
EC	Ethics Committee
ECG	electrocardiogram
eCRF	electronic Case Report Form
EDC	electronic data capture
ELISA	enzyme-linked immune sorbent assay
EOAD	early-onset Alzheimer's disease
ET	early termination
FAST	Functional Assessment Staging of Alzheimer’s Disease
FcγR	Fcγ receptor
FCSRT	Free and Cued Selective Reminding Test
FDA	Food and Drug Administration
FDG	fluorodeoxyglucose
FLAIR	fluid-attenuated inversion recovery
fMRI	functional magnetic resonance imaging
GCP	Good Clinical Practice
GDS	Geriatric Depression Scale
GRE	gradient-recalled echo
GTP1	[^{18}F] Genentech Tau Probe 1
hAPP	human amyloid precursor protein
HbA_{1c}	glycosylated hemoglobin
HIPAA	Health Insurance Portability and Accountability Act
HED	human equivalent dose
HR	heart rate
ICH	International Council for Harmonisation
iDMC	Independent Data Monitoring Committee
IND	Investigational New Drug
INR	international normalized ratio
INVIMA	Instituto Nacional de Vigilancia de Medicamentos y Alimentos
IP	intraperitoneal
IRB	Institutional Review Board
IRR	infusion-related reaction
IV	intravenous
IxRS	interactive voice or web-based response system
LOAD	late-onset Alzheimer’s disease
LP	lumbar puncture

Abbreviation	Definition
LRTI	lower respiratory tract infection
MCI	mild cognitive impairment
MedDRA	Medical Dictionary for Regulatory Activities
mITT	modified intent to treat (population)
MMSE	Mini-Mental State Examination
MRI	magnetic resonance imaging
NCI	National Cancer Institute
NCRAD	National Cell Repository for Alzheimer's Disease
NINCDS–ADRDA	National Institute of Neurological and Communicative Disorders and Stroke and the Alzheimer's Disease and Related Disorder Association
NOAEL	no observable adverse effect level
NPI	Neuropsychiatric Inventory
OLE	open-label extension
OTC	over the counter
PD	pharmacodynamic
PET	positron emission tomography
PK	Pharmacokinetic
PRA	Pharmaceutical Research Associates
PSEN (1, 2)	presenilin (1, 2) gene
PT	prothrombin time
PTT	partial thromboplastin time
Q2W	every 2 weeks
Q4W	every 4 weeks
RBANS	Repeatable Battery for the Assessment of Neuropsychological Status
RBC	red blood cell
RCR	Roche Clinical Repository
RCT	randomized clinical trial
ROI	region of interest
RPCP	randomized, placebo-controlled portions of the Phase II program
RR	respiratory rate
SAE	serious adverse event
SAP	Statistical Analysis Plan
SC	subcutaneous
SD	single dose
SDV	source document verification
SMC	Subjective Memory Checklist

Abbreviation	Definition
SPCQ	Study Partner Characterization Questionnaire
Study drug	Study drug is defined as crenezumab or placebo
Study medication	Study medication is synonymous with study drug.
T1	longitudinal magnetization relaxation time
T2	transverse magnetization relaxation time
T2*	T2 with additional inhomogeneous dephasing
Tg	transgenic
t_{max}	time to maximum concentration
TLRTI	total lower respiratory tract infections
TMT	Trail-Making Test
TSH	thyroid-stimulating hormone
ULN	upper limit of normal
VE	vasogenic edema
WBC	white blood cell

1. BACKGROUND

1.1 BACKGROUND ON DISEASE

Alzheimer's disease (AD) is the most common form of disabling cognitive impairment in older people, and it takes a devastating toll on patients and families. Due to the growing number of people living to older ages, AD will lead to a significant increase in the number of clinically affected individuals and will have an overwhelming economic impact around the world by the time today's young adults become senior citizens (Evans et al. 1989; Corrada et al. 2010).

The cardinal neuropathological features of AD include amyloid plaques and neurofibrillary tangles. The major component of amyloid plaques is a form of the amyloid-β peptide with 42 amino acids (Aβ1-42), and the major component of neurofibrillary tangles is the microtubule-associated protein, tau. Postulated elements of the pathogenic cascade include the accumulation of amyloid-β (Aβ) peptides in monomeric, oligomeric, and fibrillar Aβ species, the aggregation and phosphorylation of tau, neuroinflammation, synaptic dysfunction, and neuronal loss. The amyloid hypothesis proposes that the accumulation of certain Aβ species (e.g., soluble Aβ1-42 oligomers or Aβ1-42 fibrils) play a critical, early role in the development of AD. Aβ-modifying medication and immunization therapies have been developed to target the production, accumulation, or clearance of these Aβ species.

More than 200 mutations of the presenilin 1 (*PSEN1*), presenilin 2 (*PSEN2*), and amyloid precursor protein (*APP*) genes exist and are inherited as fully penetrant, autosomal-dominant traits which almost always result in the clinical onset of AD before the age of 65 (www.molgen.ua.ac.be/admutations/). Given this early age of onset, autosomal-dominant Alzheimer's disease (ADAD) is frequently referred to as autosomal-dominant early onset AD (autosomal-dominant EOAD). In contrast to this early onset of disease, the vast majority of AD, known as late-onset Alzheimer's disease (LOAD), is characterized by symptom onset after the age of 65. In LOAD, neither the certainty nor the timing of the onset of clinical symptoms can be predicted reliably and, therefore, LOAD is frequently referred to as "sporadic AD." While there are several genetic, mechanistic, and other biological differences between ADAD and sporadic AD, these forms of AD have very similar neuropathological and clinical features. Sporadic AD may be associated with reduced Aβ1-42 clearance, while ADAD has been associated with increased Aβ1-42 production. However, the biochemical consequences of these alterations in clearance and production in ADAD and sporadic AD, respectively, are similar, namely Aβ accumulation in the brain. It has been proposed that accumulation of the same Aβ species in the brain plays an early role in the pathobiology of ADAD as well as sporadic AD, and that both forms of the disease might respond to Aβ-modifying treatments (Bateman et al. 2011).

There is a growing belief that AD-modifying treatments targeting Aβ may need to be started before the onset of overt symptoms, when fibrillar Aβ has already plateaued, tau

(neurofibrillary) pathology is apparent, and there has been irreversible synaptic or neuronal loss, in order to have their most profound benefit (Hardy and Selkoe 2002). Because progression to mild cognitive impairment (MCI) and dementia is certain, people inheriting ADAD mutations offer a scientifically compelling group for assessing the efficacy of putative prevention strategies in smaller and shorter trials. A large *PSEN1 E280A* autosomal-dominant EOAD kindred in Colombia provides what may be a unique opportunity to conduct a study large enough to address both clinical and biomarker outcomes in a well-characterized population at virtually certain risk for AD dementia.

This population offers the opportunity to study mutation carriers in a stage of “preclinical AD,” that is, they are at certain risk of developing AD dementia but do not yet have overt symptoms and do not meet criteria for MCI or dementia (Reiman et al. 2010; Sperling et al. 2011).

There is a high unmet medical need in the ADAD population for a treatment that will slow disease progression.

1.2 BACKGROUND ON CRENEZUMAB

Crenezumab (RO5490245; MABT5102A), is a fully humanized IgG4 monoclonal antibody to Aβ slected for its ability to bind monomeric, oligomeric, and fibrillar forms of Aβ in vitro (Adolfsson et al. 2012). Crenezumab binds both Aβ1-40 and Aβ1-42, inhibits Aβ aggregation, and promotes Aβ disaggregation. Because crenezumab is a human IgG4 backbone antibody, it has reduced Fcγ receptor (FcγR)-binding affinity compared with human IgG1 or IgG2, which is predictive of reduced immune-effector response. These properties, combined with the ability of systemically delivered crenezumab to decrease Aβ CNS levels in a murine model of AD, suggest that this anti-Aβ therapeutic approach may offer clinical efficacy while reducing risk of toxicity, with the ultimate goal of modifying disease progression in patients with AD with lower risk of amyloid-related imaging abnormalities–edema/effusion (ARIA-E), including cerebral vasogenic edema (VE) and sulcal effusion, or amyloid-related imaging abnormalities–hemosiderin deposition (ARIA-H), including cerebral microhemorrhages and superficial siderosis (Sperling et al. 2011a). The encouraging safety profile to date offers the opportunity to administer high doses of the agent and achieve high brain concentrations relative to alternative monoclonal antibodies.

Crenezumab is a fully humanized IgG4 monoclonal antibody that binds to the Aβ peptides, Aβ1-40 and Aβ1-42, in multiple forms (monomers, oligomers, fibers, and plaques), notably with high affinity to oligomers, and is hypothesized to reduce oligomer neurotoxicity and accumulation (Adolfsson et al. 2012; Ultsch et al. 2016). In vitro studies demonstrated the ability of crenezumab to block Aβ aggregation, promote Aβ disaggregation, and protect neurons from oligomer-induced cytotoxicity (Adolfsson et al. 2012). Its IgG4 backbone confers reduced activation of FcγRs in comparison to IgG1 and was shown to minimize FcγR-mediated activation of microglia and release of inflammatory cytokines upon oligomer engagement—which has also

been proposed to contribute to neurotoxicity (Xing et al. 2011; Heneka et al. 2015)—while preserving FcγR-mediated microglial phagocytosis of oligomers (Adolfsson et al. 2012). Following in vivo dosing in PS2APP transgenic mice, crenezumab localizes to brain areas with putative high concentrations of Aβ oligomers, i.e., hippocampal mossy fibers and the periphery of amyloid plaques (Koffie et al. 2009; Liu et al. 2015) but not to the dense core of plaques or vascular amyloid (Study 15-2817B).

Amyloid-related imaging abnormalities (ARIA), indicative of vasogenic edema or effusions (ARIA-E) and microhemorrhages or leptomeningeal hemosiderosis (ARIA-H), have been reported in recent AD trials involving monoclonal antibodies that bind aggregated forms of Aβ and have IgG1 backbones with fully preserved FcγR-mediated effector function (Salloway et al. 2009; Ostrowitzki et al. 2012; Fuller et al. 2014; Sevigny et al. 2015). As these molecules demonstrated increases in ARIA-E incidence with increasing dose and apolipoprotein E4 (APOE4) allele frequency, the dose levels administered in past trials have been constrained to limit these events (Salloway et al. 2009). Crenezumab was designed as an IgG4 based on the hypothesis that reducing effector function would lower the risk of inducing ARIA-E/H, possibly by minimizing inflammation at brain vasculature (Wilcock et al. 2006). A lack of binding to vascular amyloid, noted following in vivo dosing in PS2APP transgenic mice (Study 15-2817B), may additionally reduce the risk of ARIA. This safety finding on crenezumab allows for the evaluation of relatively higher dose levels compared with other monoclonal antibodies that bind aggregated forms of Aβ.

The safety profile of crenezumab to date (see the Crenezumab Investigator’s Brochure for details on nonclinical and clinical studies) allows doses higher than 720 mg SC every 2 weeks (Q2W) to be studied in participants. Specifically, the dose of 60 mg/kg IV every 4 weeks (Q4W) was included as an option for participants in this study (GN28352, amendment 6). This dose was also evaluated in a Phase Ib study (GN29632), in the global Phase III studies (BN29552 and BN29553) in sporadic prodromal to mild AD and the open-label extension (OLE) study BN40031.

Crenezumab has been investigated in patients with early (prodromal to mild [sporadic]) AD with two Phase III studies (Crenenzumab for Early Alzheimer’s Disease [CREAD] [BN29552] and CREAD 2 [BN29553]) that were discontinued because a preplanned interim analysis indicated that the CREAD study was unlikely to meet the primary endpoint of change from baseline in Clinical Dementia Rating–Sum of Boxes (CDR-SOB) score. No new safety signals for crenezumab were observed in this analysis and the overall safety profile was similar to that observed in previous trials.

More details on all clinical studies conducted with crenezumab, including the Phase III studies, can be found in the Crenezumab Investigator’s Brochure.

1.2.1 Summary of Nonclinical Studies

In vivo pharmacology and CNS distribution studies were conducted in human amyloid precursor protein (hAPP) transgenic (Tg) mouse models of AD. Crenezumab was detected in both brain homogenates and cerebrospinal fluid (CSF) following a single intravenous (IV) dose. Furthermore, administration of two weekly doses of 5 mg/kg crenezumab to hAPP Tg mice decreased total Aβ levels in soluble fractions of brain homogenates while increasing plasma total Aβ levels.

An in vivo pharmacology study in PS2APP transgenic mice showed on immunofluorescence microscopy that crenezumab localized to brain areas with putative high concentrations of Aβ oligomers (i.e., hippocampal mossy fibers and the periphery of amyloid plaques) (Koffie et al. 2009; Liu et al. 2015) but not to the dense core of plaques or vascular amyloid, consistent with higher binding affinity of crenezumab for oligomers over aggregated fiber and plaque forms.

In a study to further evaluate the pharmacology of crenezumab, hAPP Tg mice received crenezumab weekly or monthly intraperitoneal (IP) administration for 16 weeks (total of 17 weekly or 5 monthly doses) at doses of up to 50 mg/kg. When given at ≥10 mg/kg for more than two weekly or monthly doses, unexpected deaths were observed. No microscopic findings were identified in the CNS (specifically, no evidence of cerebral microhemorrhage) or peripheral tissues to explain the mortalities. A high incidence of anti-crenezumab antibodies was observed (93% of samples collected from animals given crenezumab), which resulted in attenuation of both serum crenezumab concentrations and pharmacodynamic (PD) responses (plasma total Aβ1-40 and Aβ1-42). Taken together, these data suggest that these deaths are likely the result of an adverse immunogenic response to the xenogenic crenezumab antibody; that is, native mouse antibodies are produced in response to the humanized form of the antibody, and are, therefore, not predictive of human safety.

Weekly IP administration of two human/mouse chimeric anti-Aβ monoclonal antibodies to hAPP Tg mice for 24 weeks (total of 25 doses) was well tolerated at doses of up to 50 mg/kg. Specifically, no changes in the cerebral microvasculature (e.g., microhemorrhage or amyloid deposition) were observed, despite evidence of sustained antibody exposure and PD activity (plasma total Aβ1-40). These chimeric antibodies share a humanized variable domain with crenezumab but contain a murine IgG2a Fc region with either wild-type or D265A and N297A (DANA) mutations. The murine IgG2a Fc region was selected to decrease immunogenic potential in mice. The Fc DANA mutations were designed to decrease binding to murine FcγRs and to more closely approximate the reduced effector function of an IgG4 antibody (i.e., crenezumab).

Weekly subcutaneous (SC) administration of crenezumab to cynomolgus monkeys for 39 weeks (total of 40 doses) was well tolerated at doses of up to 100 mg/kg (highest dose tested). No findings related to crenezumab administration were seen on

expanded neurohistopathology of the brain. SC injection volumes of up to 5.2 mL were well tolerated. A slight increase in microscopic findings (multifocal perivascular mononuclear cell infiltrates) was observed at injection sites in crenezumab groups compared with the control group. These changes reversed following the 14-week dose-free period.

A single, 1.5-mL, SC injection of 225 mg of crenezumab (150 mg/mL) administered to New Zealand White rabbits to evaluate local tolerance was considered to be well tolerated at a concentration of 150 mg/mL. The study results supported the SC administration of 1.0 mL of crenezumab per injection site to humans in Study ABE4662g (SC bioavailability study) and in the three ongoing, Phase II studies.

Additional information on the nonclinical studies with crenezumab is provided in the Investigator Brochure.

Overall, results from nonclinical safety and pharmacology studies supported the clinical development program in sporadic AD, as well as this Phase II study in ADAD.

1.2.2 Clinical Experience

As of May 2020, crenezumab had been investigated in a total of 13 completed studies and one ongoing study in Phase I, Phase II, and Phase III clinical trials. Approximately 1789 patients and healthy volunteers have been exposed to crenezumab in these studies.

Thirteen studies have been completed including Phase I studies (ABE4427g, AB4662g, GP29172, GP29523, GN29632, GP40201, and JP29973), Phase II studies (ABE4869g, ABE4955g and GN28525) and Phase III studies (BN29552, BN29553 and BN40031). Study GN28352 is ongoing.

Study ABE4427g studied the safety, tolerability, and pharmacokinetics of single- and multiple-ascending doses of IV crenezumab in patients with mild to moderate AD who were 50–86 years of age. Study ABE4662g studied the pharmacokinetics of IV and SC dosing and the relative bioavailability of crenezumab (SC dosing) in healthy volunteers who were 18–50 years of age. Study GP29172 evaluated the pharmacokinetics of SC dosing and the bioequivalence of two formulations of crenezumab (SC dosing) in healthy volunteers who were 18–65 years of age. Study GP29523 assessed the tolerability and pharmacokinetics of a single, dose-escalated, SC administration of crenezumab in healthy volunteers. Study GN29632 evaluated the safety and tolerability of multiple doses of crenezumab in patients with mild to moderate AD with IV doses of 30, 45, 60, and 120 mg/kg Q4W. The 13-week double-blinded, dose-escalation part of the study was followed by an OLE part.

Study GP40201 was a Phase I, multicenter, partially blinded, placebo-controlled study designed to assess the safety, tolerability, and pharmacokinetics of subcutaneously-

administered crenezumab while varying dose, injection volume, flow rate, and the use of recombinant human hyaluronidase in healthy subjects.

Study JP29973 was a Phase I, double-blind, placebo-controlled, randomized, dose-escalation study investigating the safety of repeated doses of crenezumab for 13 weeks in 8 Japanese patients with mild to moderate AD.

The Phase II program (ABE4869g, ABE4955g, and GN28352), including an OLE study (GN28525), was designed to study safety and tolerability, as well as clinical and biomarker effects of crenezumab in two populations within the AD spectrum: an AD dementia population (50–80 years of age) with mild to moderate disease severity (MMSE; 18–26); and a younger population of participants (30–60 years of age) at risk for autosomal dominant AD due to a mutation (E280A) in the presenilin (PSEN) 1 gene. Studies ABE4869g and ABE4955g and the OLE study (GN28525) in patients who previously participated in either Study ABE4869g or ABE4955g have been completed.

The Phase III studies BN29552 and BN29553, which were designed to evaluate the efficacy, safety, and tolerability of 60 mg/kg IV of crenezumab in the sporadic AD population (pAD-mAD; MMSE 22–30; 50–85 years of age), were stopped early on 30 January 2019 after a preplanned interim analysis of Study BN29552 found that the study was unlikely to meet its primary endpoint of change in CDR-SOB. No new safety signals were found. The Phase III OLE study BN40031 that included patients who completed Studies BN29552 and BN29553 was also stopped.

Please refer to the *Crenezumab* Investigator's Brochure for more details.

1.2.2.1 Safety Experience in Patients with Sporadic AD

Overall, in all ongoing and completed studies in patients with sporadic AD, crenezumab was well tolerated. Adverse events occurred across different MedDRA system organ classes (SOCs) and were predominantly of mild to moderate severity. No dose relationship was observed with respect to the nature, severity, and frequency of AEs across crenezumab studies. In patients with sporadic AD, the most commonly reported AEs were as expected in elderly patients with prodromal, mild, or moderate AD.

A high-level summary of safety data is provided below. Up-to-date safety information from crenezumab studies, including the Phase II and Phase III studies, is provided in the most recent Crenezumab Investigator's Brochure.

Amyloid-Related Imaging Abnormalities

Overall, in the completed studies with a control arm, the frequency of ARIA-H was balanced between treatment arms and was as expected in an AD population. The majority of ARIA-H findings were asymptomatic.

Overall, the incidence of ARIA-E in crenezumab studies was low. Across all crenezumab studies, as of 12 December 2019, 5 patients were diagnosed with new

ARIA-E. All events were mild with a low BGTS. In the two Phase III Studies BN29552 and BN29553, 2 ARIA-E were reported in the active arms and 1 ARIA-E in the placebo arm. One event of ARIA-E was symptomatic (worsening of headache, in a patient in the active arm of BN29552). One patient who was diagnosed with ARIA-E during the double-blind part of the Phase II program (ABE4869g) developed recurrent ARIA-E during the OLE phase (GN28525). In the 4 other patients, ARIA-E resolved within 5 weeks without recurrence.

Please refer to the current Crenezumab Investigator's Brochure for further details on the incidence of ARIA in crenezumab studies.

Pneumonia

An imbalance in serious adverse events (SAEs) of total lower respiratory tract infections (TLRTIs) (including the MedDRA preferred terms of pneumonia, lower respiratory tract infection, and bronchitis) driven by pneumonia was observed in the RPCP of the Phase II program. However, this imbalance was not replicated in the pooled data of the two large Phase III Studies (BN29552 and BN29553) where the pneumonia cases were balanced between the active treatment and placebo arms (13 and 15 cases).

In addition, the rate of pneumonia in crenezumab-treated patients was within the expected ranges for an elderly AD population (Chong and Street 2008). Details on the incidence of pneumonia in the different crenezumab studies are provided in the most recent Crenezumab Investigator's Brochure.

Infusion and Injection-Related Reactions

Across all crenezumab studies in patients with sporadic AD, the vast majority of local and systemic infusion and injection-related reactions (IRRs) were non-serious, of mild or moderate severity and did not lead to changes in study treatment. An overview of the nature of IRRs and a description of the serious and severe IRRs are provided in the most recent Crenezumab Investigator's Brochure.

In the Phase Ib study GN29632, investigating treatment doses of up to 120 mg/kg, there was no relationship between the treatment dose and the incidence or type of injection and infusion-related adverse events.

1.2.2.2 Clinical Pharmacokinetics

The pharmacokinetics of crenezumab after IV administration in patients with mild-to-moderate AD were dose proportional across the dose range tested in both the single-dose (0.3–10 mg/kg) and multiple-dose (0.5–5 mg/kg weekly for four doses) phases of Study ABE4427g and were characterized by slow clearance (2.5–3.3 mL/day/kg) and a long half-life ($t_{1/2}$) (17–26 days) following a single IV dose. Overall, the observed clearance and volume of distribution in the Phase I studies are consistent with those for other humanized IgG monoclonal antibodies that exhibit

kinetics in the linear range (Mould and Sweeney 2007; Dirks and Meibohm 2010; Deng et al. 2011).

The steady-state pharmacokinetics of crenezumab were assessed in patients with mild to moderate AD in the Phase II studies (ABE4869g and ABE4955g). Steady-state trough crenezumab levels for the SC and IV cohorts were reached between Week 13 and Week 25 (after 6–12 SC doses or 3–6 IV doses). A dose-dependent increase in crenezumab steady-state trough concentrations was observed with a mean (SD) steady-state trough crenezumab concentration of 69 (30) μg/mL following administration of 300 mg SC every 2 weeks (Q2W) and 118 (72) μg/mL following 15 mg/kg IV every 4 weeks (Q4W).

In Study GN29632 in patients with mild-to-moderate AD, crenezumab pharmacokinetics at higher doses of 30–120 mg/kg IV Q4W increased proportionally to dosse of up to 120 mg/kg, as demonstrated by the proportional increase in the PK parameters C_{peak} and area under the concentration-time curve from Day 0 extrapolated to infinity (AUC_{inf}), following the first administered dose. The pharmacokinetics in this study appear to be consistent with those in prior studies.

In two Phase III studies BN29552 and BN29553, at a crenezumab dose of 60 mg/kg IV Q4W, the serum crenezumab steady-state mean [SD] trough concentrations (345 [146] μg/mL and 360 [162] μg/mL respectively, at Week 13 predose) in patients with prodromal to mild AD were consistent with those achieved in Study GN29632, and approximately 4-fold higher than the steady-state mean trough concentrations in Phase II studies ABE4869g and ABE4955g at 15 mg/kg IV Q4W.

Penetration into CSF was similar between doses and routes of administration, with a mean crenezumab CSF/serum ratio of approximately 0.3%. Overall, the steady-state crenezumab concentrations in CSF were low, with mean (SD) concentrations of 0.25 (0.12) μg/mL after 15 mg/kg IV Q4W and 0.19 (0.14) μg/mL after 300 mg Q2W SC in Studies ABE4955g and ABE4869g and approximately 1.0 ug/mL after 60 mg/kg IV Q2W in Studies BN29552 and BN29553.

Additional PK information is provided in the Crenezumab Investigator's Brochure.

1.2.2.3 Immunogenicity

The immunogenicity of crenezumab was assessed in the completed Phase I, Phase II, and Phase III Studies ABE4427g, ABE4662g, ABE4869g, ABE4955g, GN28525, GP29172, GP29523, GN29632, GP40201, and BN29552.

In the two studies GN29632 and BN29552 in patients with sporadic AD, the same dose of 60 mg/kg BW was investigated as in this study, with the following findings:

GN29632: ADA incidence was 0% (0 of 75 patients)

BN29552: The incidence of ADAs was 0.5% with two patients out of 397 exposed to crenezumab having transient ADAs throughout the study. In these two patients the presence of ADAs did not appear to have an effect on the exposure to crenezumab, based on PK data.

See the Crenezumab Investigator's Brochure for additional details on nonclinical and clinical studies.

1.3 RATIONALE FOR DOING THIS STUDY

1.3.1 Rationale for the Alzheimer's Prevention Initiative

The Alzheimer's Prevention Initiative (API) is helping to launch a new era in AD prevention research by a) evaluating suitable investigational AD-modifying treatments in cognitively normal people who, on the basis of their age and genetic background, are at the highest imminent risk of AD symptoms; b) helping to establish the biomarker endpoints and accelerated regulatory approval pathway needed for the field to rapidly evaluate promising preclinical AD treatments; c) providing a better test of the amyloid hypothesis than clinical trials in the symptomatic stages of AD; and d) providing unusually large prevention registries as a shared resource for enrollment into these and other preclinical AD trials.

These programs have been designed in an effort to provide the greatest possible benefit to the field. For instance, the trial described in this protocol will provide an opportunity to explore the effects of an anti-amyloid treatment on well-studied AD biomarkers, and the extent to which the treatment's biomarker effects predicts a clinical benefit. Furthermore, data and biological samples from the trial will be made available to the research community within specified times after the last participant's last study visit to help the field develop better ways to test preclinical AD treatments.

1.3.2 Rationale for Study

Currently approved treatments for established AD dementia are of modest efficacy. Investigational AD-modifying treatments targeting postulated elements of the pathogenic cascade include those intended to interfere with the production, accumulation, or toxic sequelae of Aβ species (Kramp and Herrling 2011). There is a growing concern that AD-modifying treatments may need to be started before the onset of overt symptoms, when fibrillar Aβ has already plateaued, tau (neurofibrillary) pathology is apparent, and there has been irreversible synaptic or neuronal loss, in order to have their most profound benefit (Hardy and Selkoe 2002). There is a poor correlation between fibrillar Aβ and the severity of cognitive impairment in clinically affected patients; trials so far suggest that Aβ-modifying treatments may have limited benefits; and an Aβ immunization therapy has been shown to retard progression of both Aβ and tau pathology in a triple transgenic mouse model only if introduced before the onset of tau pathology (Oddo et al. 2004). If one or more forms of Aβ play an early role in the development of AD (Selkoe 2008), and if an appropriately specific treatment is safe and

tolerated and is started early enough, this treatment could have a profound clinical and public health impact, helping to stave off the clinical stages of AD. There is, therefore, an urgent need to study investigational agents in people who do not yet have dementia symptoms.

It is imperative to find effective "preclinical" AD treatments, that is, therapeutic interventions initiated in people who have not yet developed MCI or dementia and aimed at postponing the onset of, reducing the risk of, or completely preventing the clinical stages of AD (Reiman and Langbaum 2009). Even modestly effective treatments could have public health benefit. For example, delaying the onset of symptoms by 5 years, without increasing lifespan, may reduce the number of AD dementia cases by 50% (Brookmeyer et al. 1998). However, preclinical AD treatment trials using progression to dementia as the endpoint require too many cognitively normal people and too many years. New and improved methods and outcomes are needed.

Because progression to MCI and dementia is certain, people inheriting ADAD mutations offer a scientifically compelling group for assessing the efficacy of putative prevention strategies in smaller and shorter trials. An extraordinarily large *PSEN1 E280A* autosomal-dominant EOAD kindred in Colombia provides what may be a unique opportunity to conduct a study large enough to address both clinical and biomarker outcomes in a well-characterized population at virtually certain risk for AD dementia. This population offers the opportunity to study mutation carriers in a stage of "preclinical AD," that is, they are at certain risk of developing AD dementia but do not yet have overt symptoms and do not meet criteria for MCI or dementia (Reiman et al. 2010; Sperling et al. 2011b).

Sensitive cognitive endpoints have been proposed that may allow subtle cognitive decline to be tracked years before manifest dementia symptoms—measurements that aim to distinguish ADAD mutation carriers without MCI or dementia from non-carriers and also to distinguish between cognitively normal persons who did and did not progress to the clinical stages of "sporadic" LOAD (Ayutyanont et al. 2014; Langbaum et al. 2014). Capitalizing on this development, the primary aim of this study is to evaluate whether treatment with crenezumab will result in better cognitive performance over time, in comparison to placebo treatment, in individuals without MCI or dementia but who are at high risk for developing symptomatic AD. With effective preventive AD treatments being needed urgently, ADAD families hold a key by permitting shorter and smaller trials to be conducted so that critical answers can be found sooner.

Another important goal of the study is to further develop biomarkers that can be used to predict and monitor the progress of disease, as well as predict and monitor treatment effects (Reiman and Langbaum 2009; Reiman et al. 2010). Brain imaging and fluid biomarkers of AD pathology and progression (e.g., PET measurements of cerebral metabolic rate for glucose [CMRgl] decline in AD-affected regions, MRI measurements of regional or whole brain volume loss, PET measurements of fibrillar Aβ or of tau

deposition, CSF Aβ1-42 reductions, and total-tau [t-tau] and phospho-tau [p-tau] increases) offer promising endpoints for the accelerated evaluation of AD treatments in people in the preclinical stages of AD who are at increased risk for MCI and dementia due to AD. However, these biomarkers need to be studied in therapeutic trials to clarify the extent to which they are moved by AD-modifying treatments.

There is no precedent that indicates which outcome measures are likely to change with treatment or which changes during a preclinical state of AD are meaningful for preventing functional deficits in symptomatic stages of AD. Because of this lack of precedent, and the scarcity of ADAD participants, health authorities have suggested there may be flexibility when considering outcome measures in a rare clinical trial opportunity in nonclinical ADAD (http://www.alzforum.org/sites/default/files/legacy/new/pdf/ColombianSeries.pdf). Additionally, AD biomarker development is rapidly evolving. Therefore, this trial in preclinical ADAD will incorporate multiple objectives and outcome measures that reflect some flexibility on the part of health authorities in the approaches used to assess treatment effects.

Despite the futility of the recently discontinued Phase III program of crenezumab in sporadic AD (see Section 1.2), Study GN28352 continues given that this at risk ADAD population differs from the sporadic prodromal to mild AD population in the following ways: The population is much younger, clinically asymptomatic at study entry, and carries a mutation that is specifically linked to amyloid metabolism.

2. OBJECTIVES

2.1 EFFICACY OBJECTIVES

2.1.1 Primary Efficacy Objective

The primary objective*s* of this Phase II trial are

- to evaluate the efficacy of crenezumab treatment compared with placebo for at least 260 weeks on change in cognitive function *as measured by the API ADAD Cogntive Composite Test Battery* in preclinical *PSEN1 E280A* autosomal-dominant mutation carriers

- *to evaluate the efficacy of crenezumab treatment compared with placebo for at least 260 weeks on change in episodic memory function as measured by the Free and Cued Selective Reminding Test (FCSRT) Cueing Index in preclinical PSEN1 E280A autosomal-dominant mutation carriers.*

2.1.2 Secondary Efficacy Objectives

The secondary efficacy objectives of this study (comparing crenezumab with placebo) will be to evaluate the following in *PSEN1 E280A* mutation carriers:

- Ability of crenezumab to affect clinical endpoints other than*those in* the primary *endpoint family*: API ADAD Cognitive Composite Test Battery (see Section 3.3.2.1 for details on clinical endpoints) *and FCSRT Cueing Index*
- Ability of crenezumab to reduce cerebral fibrillar amyloid burden in a predefined region of interest (ROI) using florbetapir PET
- Ability of crenezumab to reduce decline in regional cerebral metabolic rate of glucose (CMRgl) using FDG-PET measurements in a predefined ROI
- Ability of crenezumab to reduce brain atrophy as measured by volumetric measurements using MRI
- Ability of crenezumab to affect a tau-based CSF biomarker

2.2 SAFETY OBJECTIVES

The safety objectives for this study are as follows:

- To assess the safety and tolerability of crenezumab in preclinical PSEN1 E280A mutation carriers (comparing crenezumab with placebo)

2.3 PHARMACOKINETIC/PHARMACODYNAMIC OBJECTIVES

The PK and PD objectives for this study are as follows:

- Collect sparse PK samples to support confirmation of exposure to crenezumab and to explore the PD response (as measured by plasma total Aβ levels comparing crenezumab with placebo) in preclinical *PSEN1 E280A* mutation carriers

2.4 EXPLORATORY OBJECTIVES

The exploratory objectives for this study are as follows:

- Assess further the effect of crenezumab in preclinical *PSEN1 E280A* mutation carriers on additional clinical measures of efficacy and biological markers of disease that have not been prespecified as primary *or* secondary endpoints in the Statistical Analysis Plan (SAP)
- Explore pharmacogenetic effects including, but not limited to, a person's apolipoprotein E (APOE) ε4 carrier status on the active treatment's cognitive, clinical, and adverse effects
- Explore effects of genetic variation, including, but not limited to, how genes affect the biology of AD and related disorders and how genes influence biomarker responses
- Examine clinical and biomarker changes in non-carriers and to compare these changes with those seen in carriers treated with placebo
- Relate the treatment's biomarker effects to clinical outcomes and to examine predictive and prognostic utility of baseline characteristics

- Assess the impact of treatment on brain tau load over time, as measured by tau PET imaging in an optional substudy (GN28352-1/BN40199)

3. STUDY DESIGN

3.1 DESCRIPTION OF THE STUDY

Study Period A: This is a prospective, randomized, double-blind, placebo-controlled, parallel-group study of crenezumab versus placebo in individuals who carry the *PSEN1 E280A* autosomal-dominant, mutation-causing EOAD and do not meet criteria for MCI due to AD (Albert et al. 2011) or dementia due to AD (McKhann et al. 2011) and are, thus, in a preclinical phase of AD (Sperling et al. 2011b). The study also incorporates administration of placebo to individuals who are not PSEN1 E280A autosomal-dominant mutation carriers. This efficacy and safety study will be conducted at a single primary site and approximately three satellite sites in Colombia.

In the Colombian study, *PSEN1 E280A* autosomal-dominant mutation carriers who meet study eligibility criteria will be randomized in a 1:1 ratio to one of two treatment groups: crenezumab or placebo. Crenezumab will be administered either subcutaneously (720 mg Q2W) or intravenously (60 mg/kg Q4W). Matching placebo will be administered by the same routes at the same frequencies. The switch to the higher IV dose (approximately 4-fold higher exposure to crenezumab) will be optional. Participants may decide whether to change from the current SC dosing to the IV dose; however, once IV dosing in a given participant has occurred, it is not intended for a participant to switch back to the lower SC dose. In order to maintain genotype blind and to have a genetic kindred control, a cohort of *PSEN1 E280A* mutation non-carrier kindred family members will also be enrolled in the study and dosed only with placebo.

The study includes three arms with approximately 100 participants per arm; two arms of participants with a *PSEN1 E280A* mutation randomized in a 1:1 ratio to active or placebo and a third arm of non-carriers randomized to placebo. Participants will receive the randomized treatment until the final participant has reached their final treatment visit at 260 weeks (see Figure 1a).

The study duration for individual participants will be at least approximately 284 weeks, including an 8-week screening period, a double-blind treatment period of at least 260 weeks in length, 4-week final dose visit, and a final safety follow-up visit 16 weeks after the last dose of study drug (crenezumab or placebo) that will allow for clinical follow-up after treatment discontinuation for participants who do not to continue with study drug beyond the end of Study Period A or who terminate study drug early. One or more interim analyses may be performed at the Sponsor's discretion (see Sections 3.2 and 4.10.9).

Study Period B: Following the completion of Study Period A, participants will be offered the opportunity to continue to receive study drug until the results of the study are known

and post trial access to crenezumab is started (via an OLE or other program) or development of crenezumab is discontinued. This is termed Study Period B(see Sections 3.2.1, 4.5.5, 4.5.8). All PSEN1 mutation carriers will be provided crenezumab in Study Period B regardless of treatment allocation during Study Period A and all non-carriers will continue on placebo. Treatment allocation for both study periods A and B will remain blinded. Study Period B may last up to approximately 9-12 months depending on when participants enter Study Period B. See figure 1b.

See Appendix A-1 and Appendix A-2 for the study flowchart for SC dosing.

See Appendix A-3 and Appendix A-4 for the study flowchart for IV dosing.

Figure 1a Study Design

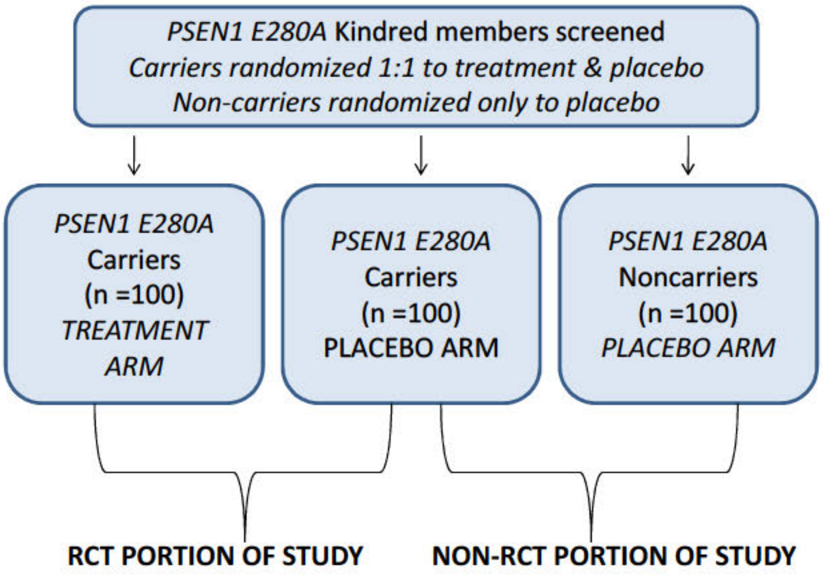

Figure 1b: Study Schematic:

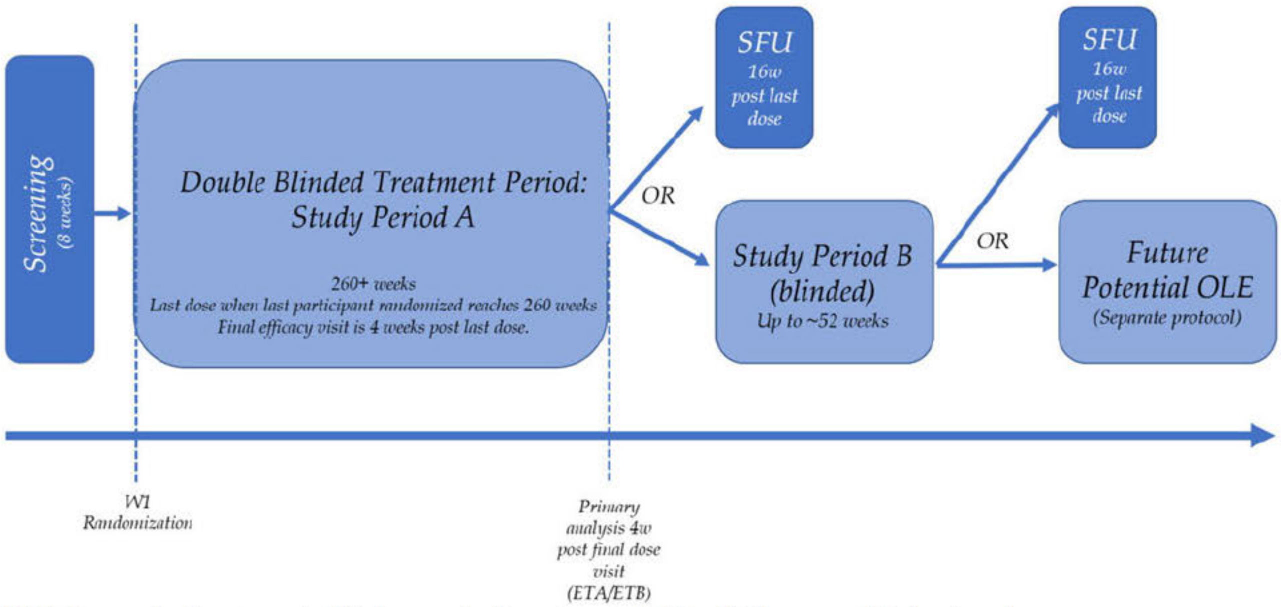

ETA/ETB = end of treatment visit A or end of treatment visit B; OLE = open label extension; RCT = randomized controlled trial; SFU = safety follow up; W = week

3.2 RATIONALE FOR STUDY DESIGN

Study Period A: The study will utilize a common-close design. All participants will be enrolled in the double-blind portion of the study until the last ongoing participant who is enrolled in the study and remains on treatment (i.e., ongoing enrolled participant) has received approximately 260 weeks of treatment, as far as is practical (see Section 4.5.4). All participants will not have the same amount of follow-up after enrollment but all will have at least 5 years of double-blind treatment. The common-close design will increase the power of detecting a difference between crenezumab and placebo by utilizing the data obtained from each participant, including those data from participants with more than 5 years' treatment duration, in the overall analysis. Furthermore, the common-close design allows similar duration of treatment with the higher IV dose (60 mg/kg IV) or matching placebo, for analysis of treatment at a higher dose.

The rationale for changing the study design is to maintain statistical power of 80% to detect a treatment effect (see Section 4.10 for further details)—despite the fact that only 252 participants of the 300 originally planned could be recruited from this kindred—and to allow reasonable duration of treatment for assessment of benefit at the higher dose.

Study Period A will be continued until the last ongoing enrolled participant has reached 260 weeks, as far as is practical (see Section 4.5.4).

One or more interim analyses may be conducted at the Sponsor's discretion at any point during the study. Such an analysis may be triggered by, for example, the availability of results from another clinical study with crenezumab.

3.2.1 Rationale for Study Period B

For the first participants reaching the final efficacy visit in Study Period A (End of Treatment-A or End of Treatment-B visit), several months will pass before the study is completed and results are available. In order to honor the long commitment of participants and not to force a treatment gap in case crenezumab is effective in slowing disease progression, participants will be offered the opportunity to continue to receive blinded study drug until post trial drug access is started (via an OLE or other program) or there is a decision to terminate the development of crenezumab on the basis of the results in GN28352. Continuation into Study Period B is optional.

Treatment and Dosing:

After completing the final efficacy visit in Study Period A, all mutation carriers who enter Study Period B will receive crenezumab and all non-carriers entering Study Period B will continue to receive placebo. Participants, site staff, vendor staff, and Sponsor will remain blinded to study drug treatment assignment during the blinded treatment period in Study Period A as well as during the Study Period B. (See Section 3.7 for details on blinding). Following database lock for Study Period A, selected members of the sponsor

team may become unblinded for the purpose of analysis and interpretation of Study Period A data.

Participants will have the option to receive the same dose (720 mg Q2W SC or 60 mg/kg Q4W IV) they were receiving at the end of Study GN28352 blinded treatment period or switch to the higher dose of 60 mg/kg Q4W IV if they have not already switched. See Section 3.2.2 for rationale for Crenezumab Dosage.

3.2.2 Rationale for Crenezumab Dosage

In this Phase II study, crenezumab 720 mg SC Q2W was selected as the dose and dosing regimen, following the results of the Phase II studies in sporadic AD; this dose provides similar exposures as the 15-mg/kg IV Q4W dose in Phase II that suggested a trend for efficacy in sporadic AD dementia. This SC dose has been administered in this study since August 2015 and has so far been well tolerated. Since July 2019, a higher dose of 60 mg/kg IV Q4W is being offered to study participants as this is assumed to have a higher potential for efficacy and was also being investigated in Phase III studies in sporadic AD.

The concurrently running Phase III program (CREAD [BN29552] and CREAD 2 [BN29553] studies) in patients with prodromal or mild (sporadic) AD tested a 4-fold higher dose (i.e., 60 mg/kg IV Q4W) of crenezumab than in Phase II compared with placebo. Based on results from a preplanned interim analysis of CREAD assessing the safety and efficacy of crenezumab that indicated that the CREAD study was unlikely to meet the primary endpoint of change from baseline in CDR-SOB score, both Phase III studies were recently discontinued.

Study GN28352 continues given that this at risk ADAD population differs from the sporadic prodromal to mild AD population in the following ways: The population is much younger, clinically asymptomatic at study entry, and carries a mutation that is specifically linked to amyloid metabolism. Approximately half of the carrier participants have been reported to have negative amyloid PET scans at baseline (Reiman et al. 2018). In addition, this PSEN1 E280A mutation is reported to be associated with an increased rate of amyloid accumulation in brain, as measured by amyloid PET compared with sporadic AD (Fleisher et al. 2015), suggesting the potential for crenezumab in delaying the progression of amyloid pathology may be may provide greater benefit in this patient population compared with sporadic AD. Furthermore, a quantitative systems pharmacology model of AD has been developed by Genentech and calibrated to both the ADAD (PSEN1 E280A mutation) and sporadic AD populations. Simulations from this model predict that both the 720-mg SC Q2W and 60-mg/kg IV crenezumab Q4W dosing regimens will delay the progression of amyloid accumulation in the brain in the ADAD population, as measured by amyloid PET standard uptake ratio, with a greater effect associated with the 60 mg/kg IV Q4W regimen (internal data).

Both doses of crenezumab offered in this study are predicted to result in drug concentrations in the brain above the in vitro half maximal effective concentration (EC_{50}) in the brain (see Figure 2). Higher doses of crenezumab are predicted to increase concentrations in the brain compared to the concentrations resulting from the 15-mg/kg IV Q4W dose, and therefore to maintain drug concentrations above the in vitro EC_{50} in the brain throughout the treatment period. In vitro, the EC_{50} for crenezumab binding to Aβ1-42 monomers and aggregates was approximately 0.2 μg/mL, and higher target engagement of soluble Aβ was observed with higher crenezumab concentrations (Adolfsson et al. 2012). Crenezumab binds to both Aβ1-40 and Aβ1-42 with high affinity (7.3 ± 0.6 nM and 11.1 ± 1.2 nM, respectively), inhibits Aβ aggregation and promotes Aβ disaggregation.

Doses of crenezumab up to 120 mg/kg IV Q4W have been safe and well tolerated (Phase Ib study GN29632) and Phase III studies investigated the dose of 60 mg/kg IV Q4W. No safety signals for crenezumab were observed in the CREAD interim analysis and the overall safety profile was similar to that observed in previous trials. Nonclinical safety data provide 2-, 3-, and 6-fold safety factors for the 60-mg/kg dose at steady state on the basis of human equivalent dose, C_{max}, and average exposure (AUC), respectively, relative to the maximum tested dose of 100 mg/kg/wk administered by SC injection in cynomolgus monkeys (for more details, refer to the Crenezumab Investigator's Brochure).

In summary, based on available in vitro affinity, clinical PK and PD data, the dose of 720 mg SC Q2W remains a dose with the potential to result in meaningful efficacy in this at risk population (ADAD) that has not previously been studied. The higher dose of 60 mg/kg IV Q4W is predicted to yield higher target engagement and has been found to be safe and well tolerated in patients with sporadic AD.

In the current study, beginning with protocol Version 6, use of a higher dose (60 mg/kg IV Q4W) than previously used in this study will be offered to participants in addition to the dose already available (720 mg SC Q2W) as it provides participants with an opportunity to receive a dose that may provide greater efficacy. Participation in the study with treatment at a higher dose is at the discretion of the participant after discussion of the different dosing options.

Figure 2 Pharmacokinetic Profile Based on Modeling and Simulation Data for 60 mg/kg Crenezumab Administered Intravenously Q4W Compared with 15 mg/kg Crenezumab Administered Intravenously Q4W

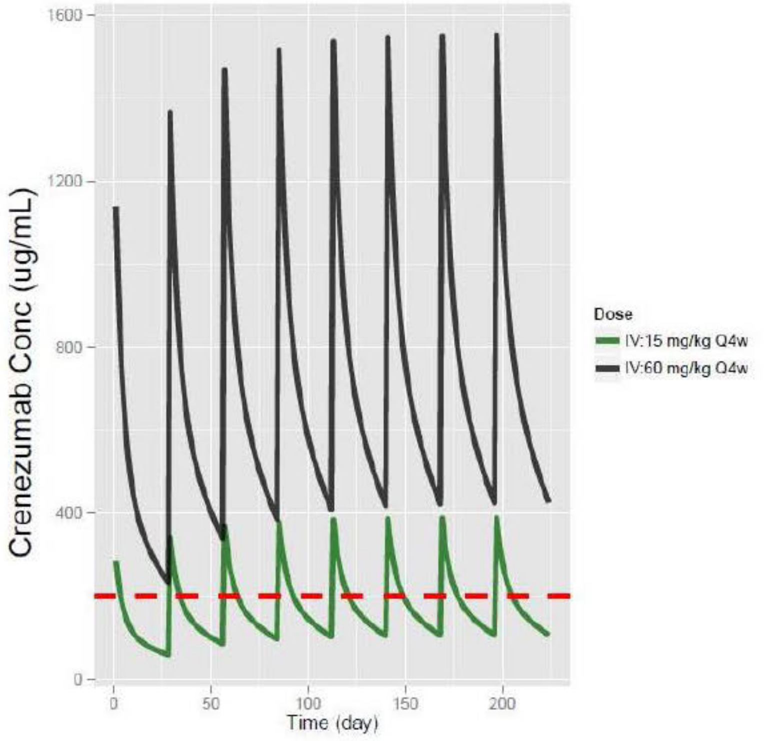

Conc = concentration; Q4W = every 4 weeks.
Note: Dotted red line is half maximal effective concentration (EC_{50}).

3.2.3 Rationale for Participant Population

The study will enroll up to 200 *PSEN1 E280A* autosomal-dominant mutation carriers who meet research criteria for preclinical AD (Sperling et al. 2011b) and up to 100 kindred mutation non-carriers in Colombia. Participants will be enrolled at the University of Antioquia in Medellin, Colombia. As mentioned previously, people with ADAD mutations offer a scientifically compelling group for assessing the efficacy of putative prevention strategies in smaller and shorter trials because progression to MCI and dementia is certain. The well-characterized, world's largest *PSEN1 E280A* autosomal-dominant EOAD kindred in Colombia (Acosta-Baena et al. 2011) provides a rare opportunity to conduct a well-powered study.

The participant population will be selected to include mutation carriers in a stage of "preclinical AD," that is, they do not yet meet criteria for MCI or dementia (Reiman et al. 2010; Sperling et al. 2011b) but are destined to develop AD dementia by virtue of their ADAD mutation status.

In order to maximize the chances of observing a change in clinical and biomarker outcomes during the ≥ 5-year treatment period of this trial, the enrolled participant population will be 30 years of age or older. The preclinical population ≥ 30 years of age was the population in which the empirically derived API ADAD Composite Cognitive Test Battery was developed and determined to be sensitive in tracking decline. Furthermore, imaging data in Colombian *PSEN1 E280* mutation carriers suggests that amyloid accumulation begins around the age of 30 (Fleisher et al. 2012). Therefore, this preclinical population may be optimal for detecting cognitive changes, the emergence of symptoms indicative of MCI due to AD, and amyloid-related biomarker changes.

A non-carrier group from the same kindred in Colombia will also be enrolled. The primary rationale for including non-carriers is to maintain blinding to genotype status to study participants, blinded study site personnel, and the Sponsor. The community standard in Colombia is to not be apprised of one's mutation carriage status in either research or clinical contexts. The inclusion of mutation non-carriers will also provide a genetic kindred control against which to explore the rate of clinical progression and biomarker changes in ADAD mutation carriers. The mutation non-carriers will be dosed only with placebo.

3.3 OUTCOME MEASURES

3.3.1 Primary Efficacy Outcome Measure*s*

The primary efficacy outcome measure*s* for this study *are* as follows:

- Annualized rate of change on the API ADAD Composite Cognitive Test total score, which is computed from the following five cognitive test scores:
  - Word List: Recall (Rosen et al. 1984; Morris et al. 1989; Mohs et al. 1997)
  - Multilingual Naming Test (Gollan et al. 2012)
  - MMSE (Folstein et al. 1975) (for orientation to time)
  - CERAD Constructional Praxis (Morris et al. 1989) (a measure of visuospatial ability)
  - Raven's Progressive Matrices (Raven 1976) (a measure of nonverbal fluid reasoning and visuospatial abilities), *Set A*
- *Annualized rate of change on the FCSRT Cueing Index*

3.3.2 Secondary Efficacy Outcome Measures

The final selection of secondary outcome measures will be based on the basis of baseline data from the study population, relevant completed trials, and other external data that will emerge prior to database unblinding. The selected outcomes will be provided in the SAP to be submitted prior to database unblinding. Secondary outcome measures will be selected from the following:

3.3.2.1 Clinical

- Time to progression from preclinical AD to MCI due to AD (Albert et al. 2011) or from preclinical AD to dementia due to AD (McKhann et al. 2011) (see Section 4.5.1 [o])
- Time to progression to non-zero in the Clinical Dementia Rating (CDR) Scale global score (Morris 1993)
- Annualized rate of change in the CDR Scale–Sum of Boxes (Morris 1993)
- Annualized rate of change in a measure of overall neurocognitive functioning: Repeatable Battery for the Assessment of Neuropsychological Status (RBANS) (Randolph 1998)

3.3.2.2 Biomarkers

- Imaging biomarkers
  - Annualized rate of change in mean cerebral fibrillar amyloid accumulation using florbetapir PET from a predefined ROI
  - Annualized rate of change in regional cerebral metabolic rate of glucose (CMRgl) using FDG-PET in a predefined ROI
  - Annualized rate of change in volumetric measurements using MRI
- CSF biomarkers
  - Annualized rate of change in a tau-based CSF biomarker

3.3.3 Safety Outcome Measures

Safety will be assessed through regular neurological and physical examinations (including vital signs), prospective suicidality assessment (copyright Pfizer Inc. and Janssen Alzheimer Immunotherapy; used with permission), laboratory tests, ECGs, and MRI assessments. In addition, the following data will be collected and analyzed:

- Frequency and severity of treatment-emergent AEs and SAEs
- Withdrawals due to AEs
- Incidence of injection reactions and IRRs
- Incidence of anti-crenezumab antibodies
- *Incidence of treatment-emergent a*dverse events of special interest (AESI), *including:*
  - ARIA-E (cerebral VE, sulcal effusion)
  - ARIA-H (superficial central nervous system [CNS] siderosis, cerebral microhemorrhages)
  - cerebral macrohemorrhages
  - pneumonia

3.3.4 Pharmacokinetic/Pharmacodynamic Outcome Measures

The PK outcome measures are CSF and serum crenezumab concentrations at protocol-specified timepoints. In serum, trough serum crenezumab concentrations at steady state ($C_{trough,\ ss}$) will be assessed. Additional PK analyses may be conducted, as appropriate.

The PD outcome measures are as follows: plasma Aβ1-40 and Aβ1-42 concentrations.

3.3.5 Exploratory Outcome Measures

Exploratory outcome measures are listed below. In addition, any endpoints (among those listed in Section 3.3.2) that are not prespecified in the SAP as secondary outcome measures may be analyzed as exploratory outcome measures.

3.3.5.1 Clinical

- Changes from baseline over time in the following cognitive measures:
  - Trail Making Test (Armitage 1946)
  - Mini-Mental State Exam (MMSE) (Folstein et al. 1975)
  - Repeatable Battery for the Assessment of Neuropsychological Status (RBANS) Index Scores (Randolph 1998)
  - Scores of each of the components of the API ADAD Composite Cognitive Test Battery
  - *Preclinical Alzheimer's Cognitive Composite (PACC) (Donohue et al. 2014)), which for this trial will include the FCSRT free and cued recall, MMSE, RBANS story recall, and RBANS coding score.*
  - Clinical endpoints not examined in secondary outcome measures
- Changes in the Neuropsychiatric Inventory (NPI) (Cummings et al. 1994; Cummings 1997) total score, items, and factors
- Changes in the Geriatric Depression Scale (GDS) (Sheikh and Yesavage 1986) total score
- Changes in Functional Assessment Staging of Alzheimer's Disease (FAST) (Sclan and Reisberg 1992) total score
- Changes in Subjective Memory Checklist (Acosta-Baena et al. 2011)

3.3.5.2 Fluid Biomarkers

- CSF levels of Aβ species
- Changes in other blood and CSF measures

3.3.5.3 Imaging Biomarkers

- Analysis of ROIs not selected in secondary outcome measures
- Other imaging outcome measures and analytic methods, alone, or in conjunction with other imaging modalities not explored in secondary outcome measures

- Change in tau burden over time, as measured by GTP1 PET in an optional substudy (GN28352-1/ BN40199)

3.3.5.4 Other Outcome Measures

- Changes in primary, secondary, and exploratory outcomes in mutation non-carriers treated with placebo
- Comparisons of clinical and biomarker outcomes between carriers treated with placebo and non-carriers
- Changes in primary, secondary, and exploratory outcomes in carriers and non-carriers as functions of APOE genotype and other genetic variations
- Short-term changes in imaging measures as functions of initiation (e.g., baseline to 12 weeks)
- Analyses of outcome measures in relation to one another and in relation to baseline characteristics

3.4 SAFETY PLAN

See Section 5 (Assessment of Safety) for complete details of the safety evaluation for this study.

Crenezumab has not been approved for use by global health authorities and is currently in clinical development. Thus, the entire safety profile is not known at this time. The crenezumab safety information is based on results from nonclinical and clinical studies, as well as published data on similar molecules. The safety monitoring for this study is designed to ensure participant safety and will include specific eligibility criteria and monitoring assessments. Any AEs/SAEs or any other abnormalities (e.g., laboratory assessments, radiographs, physical examination findings) should be managed by the site investigators, as medically appropriate (unless otherwise specified in the protocol).

The safety of participants in this trial will be ensured through the use of stringent inclusion and exclusion criteria and through close monitoring of participants. Investigators will assess the occurrence of AEs and SAEs at all participant evaluation timepoints during the study. All AEs and SAEs, whether volunteered by the participant, discovered by study personnel during questioning, or detected through physical examination, laboratory tests, or other means, will be recorded in the participant's medical record and on the appropriate AE or SAE electronic Case Report Form (eCRF).

Each recorded AE or SAE will include a description of its duration (i.e., start and end dates), severity, seriousness according to regulatory criteria, if applicable, and suspected relationship to the investigational product, as well as any actions taken.

Participants will be followed carefully for AEs during the study, including a 4-week postdose visit and a final safety follow-up visit 16 weeks after the last dose of study drug (for the list of study assessments, see the study flowcharts in Appendix A-1 to

Appendix A-4). AEs will be graded according to severity using the NCI CTCAE, Version 4.0.

An Independent Data Monitoring Committee (iDMC) formerly known as the Data and Safety Monitoring Board (DSMB) will be used for this study.

Please refer to the latest Crenezumab Investigator Brochure for additional safety information.

3.4.1 Immunogenicity

Immunogenicity, as determined by the presence of positive response in the ADA enzyme-linked immunosorbent assay (ELISA), will be assessed at specified timepoints during the course of the study. Additional characterizations will be performed if necessary.

3.4.2 Amyloid-Related Imaging Abnormalities

Amyloid-related imaging abnormalities are a spectrum of image abnormalities detected on MRI. Two types of ARIA have been described: ARIA-E refers to the MRI alterations thought to represent vasogenic edema and related extravasated fluid phenomena, and ARIA-H refers to the MRI alterations attributable to microhemorrhages and leptomeningeal hemosiderosis.

The occurrence of imaging abnormalities believed to represent cerebral VE has been reported in association with the investigational use of compounds that are intended to treat AD by reducing Aβ in the brain. These imaging abnormalities (Salloway et al. 2009; Sperling et al. 2011a) have, in the majority of instances, been asymptomatic, and their presence has been detected by brain MRI. Symptoms, when present in association with such imaging abnormalities, have been reported to include headache, worsening cognitive function, alteration of consciousness, seizures, unsteadiness, and vomiting.

The clinical significance of ARIA-H is not yet fully understood.

When anti-Aβ antibodies bind to amyloid deposited around blood vessels, an FcγR-mediated immune response may be elicited, compromising vascular integrity and resulting in ARIA-E or ARIA-H. Crenezumab, a human IgG4, has reduced FcγR binding compared with IgG1/2 and, thus, has reduced effector function that theoretically might lower the risk of ARIA-E. This has been supported by emerging safety data observed in both clinical and nonclinical studies conducted to date. A lack of binding to vascular amyloid, noted following in vivo dosing in PS2APP transgenic mice (Study 15-2817B), may additionally reduce the risk of ARIA.

To minimize the potential safety risks associated with ARIA-E and ARIA-H in this study, participants will be screened for ARIA-E and ARIA-H before enrollment and will be monitored for ARIA-E and ARIA-H by MRI during treatment (see Section 3.4.2.1).

Given the uncertainty of risk and concerns about cerebral amyloid angiopathy (CAA) severity, participants with superficial siderosis of the CNS, more than four cerebral microhemorrhages, or evidence of a prior cerebral macrohemorrhage at screening will be excluded from this study.

An overview of the monitoring and management guidelines for ARIA-E and ARIA-H is provided in the following sections.

The cerebral microhemorrhage thresholds for study entry and treatment discontinuation allow for imaging measurement variability to be taken into account and reflect the uncertainty regarding the clinical relevance of small numbers of cerebral microhemorrhages. This approach is intended to minimize potential safety risks associated with cerebral microhemorrhages but would not preclude participants from receiving potentially beneficial treatment (see Section 3.4.2.2).

3.4.2.1 Monitoring for Amyloid-Related Imaging Abnormalities

In this Phase II study, a safety monitoring plan has been designed to mitigate and monitor the potential risk of ARIA-E and ARIA-H associated with anti-Aβ immunotherapy. This plan consists of the following key elements:

- Participants with evidence of one or more of the following abnormalities assessed by MRI during screening will be excluded from the trial:
  - Significant cerebral vascular pathology
  - Superficial siderosis of the CNS
  - More than four cerebral microhemorrhages
  - Evidence of a prior cerebral macrohemorrhage
- Participants enrolled in the study will regularly undergo neurological examinations to evaluate for any neurologic signs or symptoms that are suggestive of the presence of the following:

  Cerebral VE (e.g., confusion, dizziness, vomiting, and lethargy), superficial siderosis of the CNS (e.g., sensorineural deafness, cerebellar ataxia, and pyramidal signs), or cerebral microhemorrhages (e.g., transient or permanent focal motor or sensory impairment, ataxia, aphasia, and dysarthria)
- Brain MRI exams (including fluid-attenuated inversion recovery [FLAIR] and T2*-weighted gradient-recalled echo [GRE] sequences) will be performed for safety monitoring approximately every 3 months for the first year and approximately every 6 months thereafter to detect potential safety risks noted below. If switching to 60 mg/kg IV Q4W, MRI scans will be performed at 3 months and 6 months after initiating the higher dose and then every 24–28 weeks thereafter to detect the following: ARIA-E, ARIA-H, and cerebral macrohemorrhages (see study flowchart in Appendix A-1, Appendix A-2, Appendix A-3, and Appendix A-4 for MRI schedule).
- All safety MRI scans will be read for the evaluation of ARIA-E, ARIA-H, and cerebral macrohemorrhages by independent radiologists at a central reading facility.

- Regardless of severity, all events of cerebral VE, sulcal effusion, superficial siderosis of the CNS, and cerebral micro- or macro-hemorrhage that occur at any time after receiving study drug are considered to be AESI and will be reported in an expedited manner (see Section 5.4).

3.4.2.2 Management of Amyloid-Related Imaging Abnormalities

Amyloid-Related Imaging Abnormalities–Edema/Effusion (ARIA-E)

The following rules for the management of MRI-related findings apply for ARIA-E.

- Asymptomatic ARIA-E with Size < 4 Barkhof Grand Total Score (BGTS) (Barkhof et al. 2013)
  - Continue study drug at the same dose (i.e., 60 mg/kg crenezumab).
  - Repeat MRI 4 weeks later.
  - If ARIA-E is stable or decreased, continue study drug and monthly MRI monitoring until event resolves. Once ARIA-E completely resolves, conduct MRI monitoring as per study flowchart.
  - If ARIA-E increases (BGTS ≥4) or symptoms develop, refer to the rules below.
- Symptomatic ARIA-E (of any size) or asymptomatic ARIA-E with size ≥4 BGTS:
  - Temporarily interrupt study drug and implement monthly MRI monitoring.
  - Once symptoms and ARIA-E resolve, reintroduce study drug and perform an MRI after 4 weeks of dosing. If no new MRI abnormality is detected, resume MRI monitoring as per the study flowchart.
- For any new onset of ARIA-E:
- Treat the same as the first event, on the basis of symptoms and BGTS.
- If a participant is diagnosed with three recurrent, symptomatic ARIA-E events or exacerbations of previous events, permanently discontinue the study drug.
- Implement monthly MRI monitoring until resolution of both symptoms and ARIA-E.
- As per the protocol, maintain the participant in the study until study end and perform assessments as per the study flowcharts.

Amyloid-Related Imaging Abnormalities–Hemosiderin Deposition (ARIA-H)

The following dose adjustments and discontinuation rules for MRI-related findings will apply to ARIA-H:

- <u>Dose reduction:</u> Participants who develop > 8 amyloid-related imaging abnormalities-hemosiderin deposition cumulatively will receive a lower dose of the study drug. Cumulative is the sum of ARIA-H at baseline and newly detected ARIA-H during the study.
- <u>Study drug discontinuation:</u> Participants who develop > 10 ARIA-H cumulatively will be permanently discontinued from the study drug. Cumulative is the sum of ARIA-H at baseline and newly detected ARIA-H during the study.

The Sponsor believes that the above rules, coupled with frequent MRI monitoring and collection of CNS symptoms, will ensure safety while trying to maximize benefit for the participants.

3.4.3 Pneumonia

In the RPCP of Phase II clinical trials in mild to moderate AD (ABE4869g and ABE4955g) there was an imbalance in the rates of TLRTI (including preferred terms of pneumonia, LRTI, and bronchitis) driven by pneumonia. However, this imbalance was not replicated in the pooled data of the two large Phase III Studies (BN29552 and BN29553) where the pneumonia cases were balanced between the active treatment and placebo arms (13 and 15 cases).

There is no clinical evidence of immunosuppression (e.g., effects on white blood cells or neutrophils; or pattern of infection type, e.g., opportunistic infections). In addition, no evidence of immunosuppression or of a lung toxicity effect has been observed in preclinical studies.

Based on the currently available data, a causal relationship between the drug and events of pneumonia cannot be established or ruled out. Pneumonia is considered a potential risk to be monitored closely, and is added to this protocol as an AESI.

Please refer to the latest Crenezumab Investigator' Brochure for further information on the incidence of pneumonia in all completed and ongoing studies.

3.4.3.1 Monitoring for Pneumonia

- It is of paramount importance to carefully document any pneumonia cases and other serious respiratory infections that occur in this study, by means of providing all relevant information required by the eCRF.
- Participants with signs and/or symptoms suggestive of pneumonia (e.g., cough, fever, pleuritic chest pain) should undergo chest radiography for confirmation of diagnosis. Participants should be treated as per standard treatment care and any change to study drug should be considered within a benefit–risk assessment frame, and in agreement with the Medical Monitor.
- A chest X-ray is required under the following circumstances:
  - Serious and non-serious pneumonia
  - Serious and non-serious LRTIs
  - Serious upper respiratory tract infections
- In addition, whenever possible, additional relevant investigations should be conducted (e.g., WBC counts, pathogen identification by means of hemocultures, bronchoalveolar lavage).

3.4.4 Injection and Infusion Reactions

Administration of monoclonal antibodies may be associated with local reactions at the site of injection or infusion. Systemic injection/infusion reactions as well as hypersensitivity or anaphylactic reactions may also occur.

Infusion and injection-related reactions are defined as any AE that occurs during or within 24 hours after the study drug injection of infusion.

No relationship between treatment dose and the occurrence of infusion/injection related reactions has been observed. The majority of infusion-related reactions in all ongoing and completed studies with crenezumab were of mild to moderate severity.

3.4.4.1 Systemic Injection and Infusion-Related Reactions

Crenezumab will be administered to participants under close medical supervision in a setting with access to appropriate emergency equipment and staff who are trained to monitor and respond to medical emergencies.

In the event that a participant experiences a mild IRR, the infusion will be halted. Once the reaction has resolved, the infusion rate will be resumed at half of the most recently used rate (e.g., 3–1.5 mL/min).

Participants who experience a moderate IRR (e.g., fever or chills) should have the infusion stopped immediately and should receive aggressive symptomatic treatment. The infusion should not be restarted before all symptoms have disappeared, and then it should be restarted at half the initial rate.

Participants who experience serious or severe hypersensitivity reactions (e.g., hypotension, mucosal involvement) should not receive additional study drug. In addition, in any case of anaphylaxis, anaphylactoid reaction, serious or severe hypersensitivity reaction, ADA and concurrent PK samples should be collected as close as possible to the event, and then at 4 and 16 weeks post-dose (see study flowcharts for further details). Participants who receive the SC formulation of study drug will be observed for a minimum of 15 minutes following all injections.

Participants who receive IV formulation of study drug, will be observed for a minimum of 60 minutes following all infusions.

3.4.5 Additional Safety Monitoring

Laboratory tests, including hematology, chemistry, coagulation, urinalysis, and ADAs will be performed throughout the study. ECGs will be recorded regularly, including at baseline. Physical and neurological examinations will be performed throughout the study to assess AEs, particularly any neurological events that are not consistent with normal disease progression. Suicidality assessments will be used to measure prospective suicidality (see Appendix B). If the suicidality assessment reveals a concern

about a significant level of suicidality, the participant will undergo a more detailed assessment by a qualified clinician who has experience in the evaluation of suicidal ideation and behavior, either at the site or by referral to an outside clinician.

Serum pregnancy testing will be performed at screening for all women of childbearing potential (see the footnotes to the study flowcharts in Appendix A-1, Appendix A-2, Appendix A-3, and Appendix A-4). Urine pregnancy testing will be performed prior to study drug administration or PET scan for all women of childbearing potential. Pregnancy testing will apply to all women of childbearing potential unless documented (by medical records or physician's note) to be surgically sterile or postmenopausal, as defined in International Council for Harmonisation (ICH) Guidance M3 (R2), Section 11.2 (12 months with no menses without an alternative medical cause). If urine pregnancy testing returns a positive result, study drug will be held until pregnancy is verified. If pregnancy is confirmed, the participant will not receive further doses of study drug.

The incidence and nature of AEs, SAEs, and laboratory abnormalities will be assessed on a regular basis by the iDMC. The committee will recommend changes to study conduct or study termination, as required. The responsibilities and operation of the committee will be documented in the iDMC Charter.

3.5 STUDY PARTICIPANTS AND ANALYSIS GROUPS

The primary efficacy analysis will be based on a modified intent-to-treat (mITT) population. This consists of participants who are mutation carriers treated with any amount of study drug. Participants in the efficacy analysis population will be grouped based on treatment assigned.

Safety assessments will be performed for safety-evaluable participants, defined as all randomized participants who have received at least one dose of study drug. Because of the importance of assessing safety on the basis of actual drug received by a participant (especially with respect to cerebral VE, superficial siderosis of the CNS, and cerebral micro- or macrohemorrhages), participants will be grouped for all safety analyses according to the treatment they actually received rather than the treatment assigned at randomization and mutation-carrier status.

3.6 CONTROL GROUPS

Mutation carriers who receive placebo treatment will comprise the primary control comparison group.

3.7 MINIMIZATION OF BIAS

The trial is a Phase II, single-center, randomized, double-blinded, placebo-controlled study of the effects of crenezumab on clinical outcomes and biomarkers, in ADAD mutation carriers, with a non-randomized study of the effects of placebo in carriers and non-carriers.

In Study Period A, eligible carrier participants for the randomized clinical trial will be randomized in a 1:1 ratio (active to placebo) using an interactive voice or web-based response system (IxRS) to prevent bias in treatment assignment. Randomization among carriers will be stratified by age, education, APOE4 carrier status (carrier vs. non-carrier), and baseline CDR zero versus CDR non-zero in order to maximize treatment group comparability. In Study Period B, all carrier participants will receive crenezumab and noncarrier participants will receive placebo.

Participants, study site personnel who will evaluate participant status, contract research organization (CRO) personnel who will review participant eCRFs, other Sponsor agents (with the exception of the IxRS provider), and Sponsor personnel involved in study conduct will be blinded to treatment assignments through the end of the study. Because of the physical characteristics of the study drug, investigators and study drug administrators should implement strategies to maintain the blind, which are outlined in the pharmacy manual. Pharmacists and drug administrators will be unblinded to study drug allocation due to slight variations in color of active drug and placebo. Pharmacists and study drug administrators will not participate in any portion of the study outside requirements of drug preparation and administration. For further details, please refer to the pharmacy manual.

The investigator will be unblinded to study drug allocation only if the identity of the study drug is essential for participant management in the case of an SAE. The investigator will contact the Sponsor, or medical designee, to discuss the medical details and options for unblinding. The iDMC will be unblinded to treatment assignment (see Section 3.9).

3.8 ETHICAL CONSIDERATIONS

This study will comply with laws and guidelines as discussed in the following sections: Compliance with Laws and Regulations (see Section 3.10), Informed Consent Form (see Section 6.3), Communication with the Institutional Review Board (IRB) or Ethics Committee (EC) (see Section 6.4), and Disclosure of Data (see Section 6.10).

Currently, there is no approved treatment to delay or prevent the onset of symptoms of AD. Crenezumab is an investigational drug that is being studied in patients at high risk for AD due to an autosomal-dominant genetic mutation (*PSEN1 E280A*). This is the first preclinical trial in individuals who do not have MCI or dementia but are at high risk for developing clinical AD. The inclusion of two placebo groups is considered necessary and justifiable in the double-blind design of this study. A placebo mutation carrier group is necessary to allow for unbiased assessment of efficacy, safety, and tolerability of crenezumab. In order to maintain genotype blind and to have a genetic kindred control, a cohort of ADAD mutation non-carriers will also be enrolled into the study from the kindreds in a double-blinded fashion and placed only on placebo. All participants in the study will be allowed standard-of-care medications for AD (e.g., memantine and acetylcholinesterase inhibitors) if they develop symptomatic AD.

3.9 ADMINISTRATIVE STRUCTURE

The study is sponsored by F. Hoffmann-La Roche Ltd (hereafter referred to as Roche). A joint leadership team comprised of the Banner Alzheimer's Institute and Roche will be responsible for governance, operating in partnership with the Neurosciences Group of the University of Antioquia. Crenezumab was in-licensed from AC Immune and is being developed by Roche. This trial will be managed by a primary CRO. A total of up to 300 participants will be enrolled in Colombia.

The randomization of participants will be managed by a central IxRS vendor using dynamic hierarchical randomization.

All laboratory tests of blood specimens will be performed by central laboratories or Roche, as appropriate.

A central imaging reader will determine the presence of cerebral VE and sulcal effusion by FLAIR MRI, and the presence of superficial siderosis of the CNS and cerebral micro- or macrohemorrhages by T2*-weighted GRE MRI scans.

An independent Data Monitoring Committee (iDMC) formerly referred to as the Data and Safety Monitoring Board (DSMB) will be utilized during the conduct of this trial to periodically review unblinded safety and efficacy data. In addition to periodic review of safety and efficacy data, the iDMC may make recommendations regarding study conduct. Details of the iDMC membership and roles are detailed in the iDMC Charter.

3.10 COMPLIANCE WITH LAWS AND REGULATIONS

This study will be conducted in accordance with the U.S. Food and Drug Administration (FDA) regulations, the International Council for Harmonisation (ICH) E6 Guideline for Good Clinical Practice (GCP), and applicable local, state, and federal laws, as well as other applicable country laws.

4. MATERIALS AND METHODS

4.1 PARTICIPANTS

4.1.1 Participant Selection

Written informed consent will be obtained prior to initiation of any study procedure. All participants volunteering and eligible for participation in the study will be screened for conformance with the following inclusion and exclusion criteria. In Study Period A, approximately 200 participants who are *PSEN1 E280A* mutation carriers will be randomized to receive crenezumab or placebo; approximately 100 mutation non-carriers will be enrolled and will receive only placebo.

4.1.2 Inclusion Criteria

Participants must meet the following criteria for study entry:

1. Signed and dated written informed consents obtained from the participant and study partner in accordance with local regulations prior to the initiation of any protocol required procedures
2. Membership in *PSEN1 E280*A mutation carrier kindred
3. Agrees to conditions of, and is willing to undergo, genetic testing (e.g., APOE, *PSEN1 E280A*, and other genetic testing)
4. *PSEN1 E280A* mutation carrier or non-carrier status has been confirmed prior to or during the screening period
5. Males and females, age $\geq$30 years and $\leq$60 years
6. MMSE of $\geq$24 for participants with less than 9 years of education or MMSE of $\geq$26 for participants with 9 or more years of education
7. Does not meet criteria for dementia due to AD (McKhann et al. 2011)
8. Does not meet criteria for MCI due to AD (Albert 2011) as defined by the following:
    - Cognitive concern in the judgment of the investigator, based in part on the average Subjective Memory Checklist score $>$22 (average of participant and study partner component scores)
    - Word List: Recall $<$3 for participants with less than 9 years of education
    - Word List: Recall $<$5 for participants with 9 or more years of education
    - Preservation of independence in functional activities in the judgment of the investigator, based in part on review of the Functional assessment staging (FAST) (Sclan and Reisberg 1992)
9. Adequate vision and hearing in the investigator's judgment to be able to complete testing
10. If female, and not documented (by medical records or physician's note) to be surgically sterile (absence of ovaries and/or uterus or tubal ligation) or postmenopausal (as defined in Section 3.4.5), willing to undergo pregnancy tests at protocol-specific timepoints
11. For women who are not documented (by medical records or physician's note) to be surgically sterile (absence of ovaries and/or uterus or tubal ligation) or postmenopausal (as defined in Section 3.4.5), agreement to remain abstinent or use two adequate methods of contraception, including at least one method with a failure rate of $<$1% per year (e.g., hormonal implants, combined oral contraceptives, vasectomized partner) during the treatment period and for at least 16 weeks (approximately 5 half-lives) after the last dose of study drug
12. For men of reproductive potential with partners of childbearing potential (i.e., women who are not surgically sterile and are not postmenopausal, as defined in Section 3.4.5), agreement to remain abstinent or use a condom as a method of

contraception during the treatment period and for at least 8 weeks (approximately 2 half-lives) after the last dose of study drug

For male participants, a documented successful vasectomy (by medical records or physician documentation) is an acceptable alternative form of birth control.

13. Study partner who agrees to participate in the study and is capable of and willing to:
    - Accompany the participant to all required visits, as specified in the footnotes to the study flowcharts provided in Appendix A-1 and Appendix A-2 for SC administration and Appendix A-3 and Appendix A-4 for IV administration
    - Provide information for required telephone assessments
    - Spend sufficient time with the participant to be familiar with his/her overall function and behavior, and be able to provide adequate information about the participant including:
        - Knowledge about domestic activities, hobbies, routines, social skills and basic activities of daily life
        - Work and educational history
        - Cognitive performance, including memory abilities, language abilities, temporal and spatial orientation, judgment, and problem solving
        - Emotional and psychological state
        - General health status
14. Participant and study partner have evidence of the following:
    - Adequate premorbid functioning (e.g., intellectual, visual, and auditory)
    - Fluency in, and able to read, the language in which study assessments are administered
15. Willing and able to undergo neuroimaging (PET and MRI)
16. In the judgment of the investigator, no clinically significant thyroid dysfunction or B12 deficiency, as determined by the following criteria:
    - Serum TSH and B12 levels within normal or expected ranges for the testing laboratory or, if TSH or B12 values are out of range, they are judged by the investigator not to be clinically significant.
    - If the participant is undergoing thyroid replacement therapy, TSH levels must either be within normal or expected ranges for the testing laboratory or, if TSH values are out of range, they do not require any therapeutic actions (treatment or surveillance).
    - If the participant is receiving vitamin B12 injections or oral vitamin B12 therapy, B12 levels must be at or above the lower limit of normal for the testing laboratory or, if B12 values are out of range, they do not require any therapeutic actions (treatment or surveillance).
17. In good general health with no known co-morbidities expected to interfere with participation in the study

18. In the opinion of the investigator, the participant and study partner will be in accordance with the following:
    - Able to comply with the protocol
    - Able to participate in all scheduled evaluations
    - Have a high probability of completing all required tests and the study

4.1.3 Exclusion Criteria

Participants who meet any of the following criteria will be excluded from study entry:

1. Significant medical, psychiatric, or neurological condition or disorder documented by history, physical, neurological, laboratory, or ECG examination that would place the participant at undue risk in the investigator's judgment or impact the interpretation of efficacy
2. History of stroke
    - Participants with a history of transient ischemic attack may be enrolled if the event occurred ≥2 years prior to screening.
3. History of severe, clinically significant (persistent neurological deficit or structural brain damage) CNS trauma (e.g., cerebral contusion)
4. Body weight <45 or >120 kg
5. History or presence of atrial fibrillation that poses a risk for future stroke in the investigator's judgment
6. Clinically significant laboratory or ECG abnormalities (e.g., abnormally prolonged or shortened QTc interval) in the investigator's judgment
7. Current presence of bipolar disorder or other clinically significant major psychiatric disorder according to DSM-IV-TR or symptom (e.g., hallucinations, agitation, paranoia) that could affect the participant's ability to complete evaluations
    - Recorded as an AE if diagnosed after study enrollment
8. Clinically significant depression, based in part by a Geriatric Depression Scale (short form GDS) (15-point scale) score >9 at screening
9. History of seizures (excluding febrile seizures of childhood, or other isolated seizure episodes that were not due to epilepsy in the judgment of the investigator, and required at most time-limited anticonvulsant treatment, and which occurred more than 7 years prior to the screening visit)
10. Myocardial infarction within 2 years, congestive heart failure, atrial fibrillation, or uncontrolled hypertension
11. Women who are pregnant or intend to become pregnant during the conduct of this trial
12. Women who are nursing infants or intend to nurse infants during the conduct of this trial
13. History of cancer within 5 years with the exception of basal cell carcinoma, squamous cell cancer of the skin that has been previously excised with clean

margins, or any cancer that is not actively being treated with anti-cancer drugs or radiotherapy, as well as cancers that are considered to have a low probability of recurrence (with supporting documentation of this from the treating oncologist if possible)

14. Clinically significant infection within the last 30 days prior to screening (e.g., chronic, persistent, or acute infection [e.g., upper respiratory infection, urinary tract infection])
15. Brain MRI imaging results at baseline showing any of the following:
    - Evidence of any ARIA-E (cerebral VE, sulcal effusion), infection, significant cerebral vascular pathology, clinically significant lacunar infarct in a region important for cognition or multiple lacunes or a cortical infarct or focal lesions of clinical significance
    - More than four cerebral microhemorrhages (lesions with diameter $\leq$ 10 mm), regardless of their anatomical location or diagnostic characterization as "possible" or “definite”
    - Single area of superficial siderosis of the CNS or evidence of a prior cerebral macrohemorrhage (lesion with diameter $>$ 0 mm)
16. Clinically significant screening blood or urine laboratory abnormalities requiring further evaluation or treatment, including the following:
    - Impaired hepatic function, as indicated by transaminases $> 2\times$ the upper limit of normal (ULN) or abnormalities in synthetic function tests judged by the investigator to be clinically significant
    - Impaired coagulation (aPTT $> 1.2\times$ ULN)
    - Platelet count $<$ 100,000/μL
    - Glycosylated hemoglobin (HbA_{1c}) $>$ 8.0%
17. Positive urine test for drugs of abuse at screening
    - One additional screening may be performed; a positive test (except for cannabinoids) will result in exclusion (see Section 4.5.2). Results of cannabinoid assays will not be used for the determination of eligibility.
18. History of alcohol or substance dependence within the previous two years (DSM-IV TR criteria)
19. Use of any other medications with the potential to significantly affect cognition (including, but not limited to, sedatives, narcotics [e.g., opiates/opioids], hypnotics, over-the-counter [OTC] sleeping aids, sedating anti-allergy medications)
    - Intermittent or short-term use of these medications may be allowed if deemed medically necessary for the treatment of a non-excluded medical condition with approval from the Medical Monitor (refer to Section 4.4.1.2 for additional details). In addition, use of tricyclic antidepressants or benzodiazepines will be permitted if used in stable, low doses for the treatment of a non-excluded medical condition with approval from the Medical Monitor (refer to Section 4.4 for additional details).

20. Use of typical anti-psychotics or barbiturates
21. Use of non–anti-cholinergic antidepressant medications or atypical anti-psychotics unless maintained on a stable dose regimen for at least 6 weeks prior to screening
22. Use of any FDA/INVIMA-approved medications for treatment of LOAD at screening/baseline
    - Cholinesterase inhibitors and/or memantine are prohibited during the study except in participants enrolled in the study who develop AD dementia. These participants will be permitted to take approved AD treatments (cholinesterase inhibitors and/or memantine).
23. Use of anti-coagulant medication (e.g., heparanoids, heparin, warfarin, thrombin inhibitors, Factor Xa inhibitors), or known coagulopathy or platelet count $<$100,000 cells/μL within 4 weeks of the screening visit
    - Documentation required for normal PT, PTT, and INR during screening for discontinued anti-coagulants
    - Anti-platelet medications (e.g., aspirin, clopidigrel, dipyridamole) are permitted if on a stable dose for 4 or more weeks prior to screening.
    - Short-term, peri-operative use of anti-coagulants may not result in discontinuation from the study; however, any such use must be discussed with the Medical Monitor.
24. Treatment with any biologic therapy within five half-lives or 3 months prior to screening, whichever is longer, with the exception of routinely recommended vaccinations, which are allowed
25. Use of anti-seizure medication (except in childhood for febrile seizures or if used for non-seizure indications), anti-Parkinsonian, or stimulant (e.g., methylphenidate) medications
26. Use of investigational drug, device, or experimental medication within 60 days (or five half-lives, whichever is longer) of the screening visit
27. Previous treatment with crenezumab (MABT5102A) or any other therapeutic that targets Aβ
28. History of severe allergic, anaphylactic, or other hypersensitivity reactions to chimeric, human, or humanized antibodies or fusion proteins
29. Contraindication to MRI scan procedures, possibly including, but not limited to, the following:
    - Pacemakers, implantable cardioverter defibrillators, cochlear implants, cerebral aneurysm clips, implanted infusion pumps, implanted nerve stimulators, metallic splinters in the eye, other magnetic, electronic, or mechanical implants; metal fragments or foreign objects in the skin or body
    - Clinically significant claustrophobia that would contraindicate a brain MRI scan
    - Clinical history or examination finding that, in the judgment of the investigator, would pose a potential hazard in combination with MRI

30. Contraindication to PET scan procedures, possibly including, but not limited to, the following:
    - Current or recent (within 12 months prior to screening) participation in studies involving radioactive agents, such that the total research-related radiation dose to the participant in any given year would exceed the limits set forth in the U.S. Code of Federal Regulations (CFR) Title 21 Section 361.1 (http://www.accessdata.fda.gov/scripts/cdrh/cfdocs/cfcfr/CFRSearch.cfm?fr=361.1)
31. Abnormal findings that, in the opinion of the investigator, may affect the participant's response to the radiopharmaceutical and related testing procedures required for PET scans
32. Unable to read and write in Spanish
33. Any other clinical contraindications for safety or participation in lumbar punctures (optional) at the clinical judgment of the investigator

4.2 METHOD OF TREATMENT ASSIGNMENT AND BLINDING

Both *PSEN1 E280A* mutation carriers and non-carriers are being enrolled. The majority of individuals in the *PSEN1 E280A* kindred from Colombia do not wish to know their *PSEN1* genotype. In most cases, the participant will not know whether he or she is a mutation carrier or a non-carrier. Participants who have their mutation carrier status disclosed by a physician in conjunction with genetic counseling outside of the auspices of this study and are mutation carriers will be eligible for the study. Genetic disclosure performed independent of this study is not required for a mutation carrier to be eligible to participate. Non-carriers who receive information about their genetic status prior to the study will not be included. In view of methods being used to balance groups at enrollment, some otherwise potentially eligible participants may not be included in the study. In cases of genetic disclosure performed independent of this study, some otherwise potentially eligible non-carriers may also not be included in the study.

Participants, blinded study personnel, and the Sponsor will not know if a participant has been assigned to the active treatment or one of the two placebo treatment groups in Study Period A. In Study Period B, participants and blinded study personnel will continue to be blinded to treatment assignment (following database lock for Study Period A, selected members of the Sponsor team may become unblinded for the purpose of analysis and interpretation of the Period A data).

In Study Period A, a dynamic randomization design will be used with treatment allocation assigned by the IxRS vendor. Mutation carriers will be randomized to crenezumab and placebo arms in a 1:1 ratio. A smaller group of mutation non-carriers will be assigned to placebo to conduct the study, such that ADAD family members are not required to receive information about their genetic risk and to help distinguish those changes related to the predisposition to AD from those associated with normal aging in the two placebo

groups (in Study Period B, all carrier participants will be assigned crenezumab by the IxRS vendor and noncarrier participants will be assigned placebo).

While PK samples should be collected from participants assigned to the placebo arms to maintain the blinding of treatment assignment, PK assay results for these participants are generally not needed for the safe conduct or proper interpretation of this trial. A defined set of personnel responsible for performing PK assays and not otherwise involved in the conduct of the trial will be unblinded to participants' treatment assignments to identify appropriate PK samples to be analyzed. Samples from participants assigned to the placebo arms will not be analyzed except by request (i.e., to evaluate a possible error in dosing). In addition, PK and plasma PD assay results will not be released to blinded personnel until the study is unblinded.

If unblinding is necessary for participant management (e.g., in the case of an SAE for which participant management might be affected by knowledge of treatment assignment), the investigator will be able to break the treatment code by contacting the IxRS. Treatment codes should not be broken except in emergency situations. If the investigator wishes to know the identity of the study drug for any other reason, he or she should contact the Medical Monitor directly. The investigator should document and provide an explanation for any premature unblinding (e.g., accidental unblinding, unblinding due to an SAE).

For regulatory reporting purposes, and if required by local health authorities, the Sponsor will break the treatment code for all serious, unexpected, and suspected adverse reactions that are considered by the investigator or Sponsor to be related to study drug.

4.3 STUDY TREATMENT

4.3.1 Crenezumab and Placebo

4.3.1.1 Formulation

Crenezumab is produced using Chinese hamster ovary cells—purified and formulated. It is subjected to a series of quality-control tests to confirm its identity, purity, potency, quality, and sterility. The drug product is a sterile, preservative-free liquid intended for SC or IV administration and is formulated as 180 mg/mL crenezumab in 200 mM arginine succinate, 0.05% (w/v) polysorbate 20, pH 5.5. Placebo will consist of the same formulation but without the active drug. Placebo and active drug are supplied in 6-mL glass vials, with 4 mL of liquid per vial.

For further details, see the Crenezumab Investigator's Brochure and the Study GN28352 pharmacy manual.

4.3.1.2 Dosage, Administration, and Storage

Crenezumab will be administered to participants under close medical supervision in a setting with access to appropriate emergency equipment and staff who are trained to monitor and respond to medical emergencies.

SC Dosing: Crenezumab has been formulated for SC injection, and no dilution of the drug product or placebo is necessary prior to administration.

Administration: Study drug will be administered SC into the back of the arm, the thigh, or the abdomen via polypropylene syringe. The total volume of drug will be 2 × 2.0 mL (2 × 360 mg), administered in two injections. Vital signs will be measured prior to the injection. Study participants will be observed for a minimum of 30 minutes following the first four administrations of study drug (180 mg/mL formulation) and for a minimum of 15 minutes subsequently.

IV dosing: IV infusions will be administered by appropriately trained staff in the clinic.

The IV drug or placebo will be prepared and infused from the IV bag by infusion pump. The bag size, drug preparation, and infusion rates are all described in the pharmacy manual guidance. For the IV dose calculation, the participant's weight should be used. If the current weight changes by ≥ 10% from the previous reference weight, the current weight should become the new reference weight for subsequent dosing. If the participant's weight changes again by ≥ 10% from the reference weight, the IV dose should again be recalculated. See the pharmacy manual for further information.

Administration: Vital signs will be measured just prior to study drug administration, at the end of the IV infusion, and ≥ 60 minutes after the end of the infusion.

Study participants will be observed for a minimum of 60 minutes following IV administration of study drug.

Participants who experience a moderate IRR (e.g., fever or chills) should have the infusion stopped immediately and should receive aggressive symptomatic treatment. The infusion should not be restarted before all symptoms have disappeared, and then it should be restarted at half the initial rate.

Participants who experience serious or severe hypersensitivity reactions (e.g., hypotension, mucosal involvement) should not receive additional study drug. In addition, in any case of anaphylaxis, anaphylactoid reaction, serious or severe hypersensitivity reaction, ADA and concurrent PK samples should be collected as close as possible to the event, and then at 4 and 16 weeks post-dose (see the study flowcharts for further details).

Collection of all relevant clinical data including vital signs, any change to infusion speeds, etc., should be recorded on the eCRF.

Storage: Vials of study drug should be refrigerated at 2°C–8°C and protected from direct sunlight. Vials should not be frozen or stored at room temperature. Additional storage details are provided in the pharmacy manual. The pharmacy manual addresses procedures for temperature excursions.

4.3.1.3 Dosage Modification

Participants with a serious, life-threatening AE or who meet the criteria for dose discontinuation in Section 3.4.4 must not receive additional doses of study drug (crenezumab or placebo) but will be invited to, and should, remain in the study and complete study assessments per the protocol (see the study flowcharts in Appendix A-1, Appendix A-2, Appendix A-3, and Appendix A-4) if this is deemed to be safe and clinically appropriate by the investigator.

Participants who experience serious or severe hypersensitivity reactions (e.g., hypotension, mucosal involvement) should not receive additional study drug. In addition, in any case of anaphylaxis, anaphylactoid reaction, serious or severe hypersensitivity reaction, ADA and concurrent PK samples should be collected as close as possible to the event, and then at 4 and 16 weeks post-dose (see the study flowcharts for further details).

Study drug will be held for participants experiencing other clinically significant neurologic deterioration attributed to the study drug, as judged by the investigator, or other AEs (including ARIA-E is associated with neurologic symptoms or if BGTS $>$4, without symptoms). Status will be assessed by MRI follow-up every 4 weeks until symptoms and ARIA-E resolve. Reintroduce study drug and perform an MRI scan after 4 weeks of dosing. If no new ARIA-E is detected, resume MRI monitoring as per study flowcharts.

Any new onset of ARIA-E: Treat the same as the first event, on the basis of symptoms and Barkhof Grand Total Score. However, in the case where a participant is diagnosed with three recurrent, symptomatic ARIA-E events or exacerbations of previous events, permanently discontinue the study drug. Implement monthly MRI monitoring until resolution of both symptoms and ARIA-E.

ARIA-H: Dose reduction: Participants who develop $>$8 ARIA-H cumulatively will receive a lower dose of the study drug. Cumulative is the sum of ARIA-H at baseline and newly detected ARIA-H during the study.

Study drug discontinuation: Participants who develop $>$10 ARIA-H deposition cumulatively will be permanently discontinued from the study drug. Cumulative is the sum of amyloid-related imaging abnormalities-hemosiderin deposition at baseline and newly detected ARIA-H during the study.

The participant will be invited to remain in the study and complete study assessments per the protocol. All such cases should be discussed with the Medical Monitor.

4.3.2 PET Tracers

All patients that are enrolled using PET amyloid evaluation and those in the optional tau PET substudy will be assessed using appropriate PET ligands.

Details of the optional tau PET substudy are described in a separate protocol.

The florbetapir and GTP1 PET ligands used in the context of this study (and the associated substudy) have been designated as investigational products.

For information on the safety profile (reference safety information) of and reporting requirements for the PET ligands administered in this study (and the associated substudy), please refer to Section 5.7.

4.4 CONCOMITANT AND EXCLUDED THERAPIES

4.4.1 Concomitant Therapy

Concomitant therapy includes any prescription medications or OTC preparations being taken by a participant at any time from screening through the last study visit.
All concomitant medications should be reported to the investigator and recorded in the eCRF. The following is a list of medications that are permitted, permitted under special circumstances with Medical Monitor approval, and prohibited therapies.

4.4.1.1 Permitted Therapies

The following are permitted therapies:

- Stable doses of maintenance medications except for medications that may significantly affect cognition (see below)
- During the study, any treatment deemed necessary by the investigator for the management of a participant's disease, with the following exception:

  Participants requiring initiation of excluded therapies may not be able to receive further doses of study drug if initiation of the excluded medication increases the risk of receiving study drug (such as anticoagulants).

- Non-tricyclic antidepressants (including trazodone, selective serotonin reuptake inhibitors, and serotonin norepinephrine reuptake inhibitors), benzodiazepines, and atypical anti-psychotic medications if the participant is on a stable dose regimen for at least 6 weeks prior to screening

  Initiation during the trial is permitted if necessary to treat an AE and is expected to have stable dose regimen for at least 6 weeks prior to any cognitive and functional assessments.

- Short- or intermediate-acting benzodiazepines for the mandatory MRI procedure and the amyloid PET scan if the participant requires it

  Some of the imaging data (e.g., functional MRI data) would be excluded from relevant exploratory analyses.

- OTC stimulants such as caffeine, nicotine, and those found in cold and flu preparations (e.g., pseudoephedrine)
- Anti-platelet therapies during study conduct if the dose and dose regimen have been stable for 4 or more weeks prior to screening and are expected to remain stable after screening

    Initiation of antiplatelet therapy during the trial is permitted if needed to treat an AE.

4.4.1.2 Permitted Therapies under Special Circumstances (Requires Medical Monitor Approval)

- The following are permitted therapies under special circumstance and require Medical Monitor approval:
- Short-term or intermittent use of medications that may significantly affect cognition (including, but not limited to, sedatives, narcotics [e.g., opiates/opioids], hypnotics, anti-psychotic medications, OTC sleeping aids, sedating anti-allergy medications)

    These medications should, if possible, be suspended for 2 days or five drug-elimination half-lives, whichever is longer, prior to any cognitive assessment, FDG-PET scan, or MRI scan.

- Anti-cholinergic antidepressants, such as tricyclic antidepressants, and benzodiazepines, only if used at low doses that are stable for at least 6 weeks prior to screening, and if deemed medically necessary for the treatment of a non-excluded medical condition
- Non-sedating antiseizure drugs (e.g., pregabalin) use for a non-seizure indication
- Anticoagulation therapy (e.g., temporary usage during surgery, for treatment of deep vein thrombosis)

    In these circumstances, appropriate safety assessments should be made if occurring prior to a lumbar puncture. The investigator should discuss with the Medical Monitor all individual participant cases that require anticoagulant therapy.

4.4.1.3 Prohibited Therapies

The following are prohibited therapies:

- Typical anti-psychotic medications
- Barbiturates
- Anti-seizure medication for seizure indication

    Non-sedating antiseizure drugs (e.g., pregabalin) use for non-seizure indication are permitted with Medical Monitor approval.

- Anti-Parkinsonian medications
- Prescription stimulant medications (e.g. methylphenidate, dextroamphetamine, etc)

    OTC stimulants, such as caffeine, nicotine, and those found in cold and flu preparations (e.g., pseudoephedrine), are allowed.

- Long term use of anticoagulants
- Cholinesterase inhibitors and/or memantine, with the following exception:

  Participants enrolled in the study who develop AD dementia will be permitted to take approved AD treatments (cholinesterase inhibitors and/or memantine).

During the study, participants will be permitted to receive any treatment deemed necessary by the investigator for the management of their disease. However, participants requiring commencement of excluded therapies may not be able to receive further doses of study drug if initiation of the excluded medication increases the risk of receiving study drug (such as anticoagulants). Participants who are withdrawn from study drug will be invited to continue to be followed at the evaluation visits for safety, clinical and biomarker assessments, selected PK, exploratory PD, and ADA status, as specified in the study flowchart (see Appendix A-1, Appendix A-2, Appendix A-3, and Appendix A-4).

4.5 STUDY ASSESSMENTS

4.5.1 Definitions of Study Assessments

a. Vital Signs

Vital signs will include measurements of heart rate, respiratory rate, temperature, and systolic and diastolic blood pressure while the participant has been supine for ≥3 minutes. In addition, height should be obtained only at screening. Weight should be obtained at screening and at weeks indicated on the appropriate study flowchart or early termination. On dosing days, record vital signs prior to the SC injection. For IV administration, vital signs will be measured just prior to study drug administration, at the end of the IV infusion, and ≥60 minutes after the end of infusion.

b. Physical Examination

A complete physical examination should include an evaluation of the head, eye, ear, nose, throat, and the cardiovascular, dermatological, musculoskeletal, respiratory, and gastrointestinal systems. Changes from baseline abnormalities should be recorded at each subsequent physical examination. New or worsened abnormalities should be recorded as AEs if considered clinically significant in the investigator's judgment.

c. Neurological Examination

A complete neurological examination should include the evaluation of consciousness, cranial nerves, motor and sensory system, coordination and gait, and reflexes. Changes from baseline abnormalities should be recorded at each subsequent neurological examination. New or worsened abnormalities should be recorded as AEs if considered clinically significant in the investigator's judgment.

d. Electrocardiogram

To minimize variability in autonomic tone and heart rate, it is important that participants are resting quietly and in a supine position prior to recording ECGs. Blood draws and

other procedures should be avoided during the period immediately before ECG measurement, and activity should be controlled as much as possible to minimize variability due to the effects of physiologic stress. If possible, ECGs should be recorded on the same type of machine for each site involved in the study, and the same machine should be used for all ECGs for a specific participant. For safety monitoring purposes, the investigator must review, sign, and date all ECG tracings. Two paper copies of ECG tracings will be kept as part of the participant's permanent study file at the site for all timepoints. Single ECGs for only baseline, Week 4, and Week 12 should be submitted to the ECG vendor for all participants.

In an optional substudy, participants will have an additional ECG assessment prior to dosing at the Week 16 visit and 7 days (±3 days) following the Week 16 study drug administration in order to collect ECG results at steady state peak and trough drug levels. Triplicate ECGs at Baseline (two additional than for non-substudy participants), Week 16, and Week 17 will be submitted to the ECG vendor for all substudy participants, in addition to submitting the single ECG from Weeks 4 and 12. Two paper copies of ECG tracings will be kept as part of the participant's permanent study file at the site. Please refer to the ECG vendor manual for additional details.

During this substudy, each ECG assessment was to be accompanied by additional PK, PD, and exploratory plasma, serum, and RNA blood samples as well as vital signs (BP, HR, RR) after the ECG assessment (refer to Appendix A-1 [SC]) for additional details).

e. Pharmacokinetic Assessments

At specified timepoints, blood or (optional) CSF samples will be collected for measurement of serum or CSF crenezumab concentrations. On dosing days, PK blood samples will be collected prior to study drug administration unless otherwise specified.

f. Pharmacodynamic Assessments

Blood samples will be collected from all participants at baseline and other timepoints for determination of plasma total Aβ1-40 and total Aβ1-42 levels as a measure of PD response to crenezumab treatment. On dosing days, blood samples will be collected prior to study drug administration unless otherwise specified.

g. Laboratory Assessments

Samples for the following laboratory tests will be collected for analysis per the study flowcharts (see Appendix A-1, Appendix A-2, Appendix A-3, and Appendix A-4):

- Urine samples and blood samples will be obtained for screening and/or clinical safety laboratory tests, including hematology, serum chemistry, and urinalysis
  - Urinalysis: pH, protein, glucose, ketones, bilirubin, blood, specific gravity, leukocyte esterase, urobilinogen, and nitrite, as well as microscopic analysis on the basis of dipstick results

- Urine test for drugs of abuse, including amphetamines, barbiturates, benzodiazepines, cannabinoids, cocaine, and opioids
- Urine pregnancy test (for all women of childbearing potential unless documented by medical records or physician's note to be surgically sterile or postmenopausal as defined in Section 3.4.5) prior to study drug administration and PET scans unless there are two PET scans in 1 day

- Blood samples will be obtained in the fasting state at screening only. The tests will be performed by a central laboratory and will include the following:
  - Hematology: hematocrit, hemoglobin, RBC count, and WBC count with differential and platelets
  - Serum chemistry: sodium, potassium, BUN, bicarbonate, estimated glomerular filtration rate, calcium, AST, ALT, γ-glutamyl transpeptidase, total and direct bilirubin, alkaline phosphatase, lactate dehydrogenase, total protein, albumin, glucose, cholesterol, triglycerides, uric acid, creatine phosphokinase, HbA_{1c}, and, at screening only, vitamin B12, ultrasensitive TSH, and thyroxine
  - Coagulation: aPTT and INR
  - Serum β-hCG pregnancy test for all women of childbearing potential unless documented by medical records or physician's note to be surgically sterile or postmenopausal (defined in Section 3.4.5) will be performed at screening and to confirm a positive urine pregnancy test during the trial.
- All laboratory abnormalities assessed as clinically significant by the investigator should be reported as AEs (Section 5.3.1.d)
- All participants with clinically significant abnormal laboratory values will be followed until the event is resolved or stabilized, the participant is lost to follow up, or it has been determined that the study treatment or participation is not the cause of the event.

h. Immunogenicity Sampling

Blood samples will be collected to assess the possible development of ADAs in all participants as noted in the Study Flowchart (see Appendix A-1, Appendix A-2, Appendix A-3, and Appendix A-4) and in case of anaphylaxis, anaphylactoid, or serious hypersensitivity reactions. In addition, for any participant suspected of developing anaphylaxis, anaphylactoid, or serious hypersensitivity reactions warranting discontinuation of dosing, an ADA sample and a concurrent PK sample must be collected as close as possible to the event, and then at the 4-week ET visit and at the 16-week safety follow-up visit after the final dose. Serum samples will be analyzed for antibodies to crenezumab using a bridging ELISA. Samples may be tested for antibodies against other drug product substances.

i. Exploratory Blood Biomarker Assessments

Additional plasma, serum, and RNA samples will be collected from all participants at baseline and other timepoints to enable exploratory analysis of biomarkers, their response to crenezumab, and their association with clinical efficacy. These biomarkers

may include proteins of the amyloid pathway, inflammatory mediators, and other markers associated with the pathophysiology of AD. On dosing days, blood samples will be collected prior to study drug administration unless otherwise specified.

j. Blood Sample for Exploratory Genetic Assessments

Blood samples will be collected from participants for exploratory studies conducted by the investigators conducting this study and as a resource to other investigators in the scientific community. Samples may be used during this trial and up to 15 years after study results are reported to help improve the early detection and/or tracking of AD; to improve the understanding of the biology of AD and related disorders; for pharmacogenetic purposes; to help in the discovery of new treatments; and/or to provide a resource for genome-wide association, sequencing, epigenetic, and gene expression studies.

k. Genetic Sampling of ADAD and *APOE* Genotype

A blood sample will be collected from all participants at screening, and ADAD mutation and *APOE* genotyping will be performed prior to randomization.

l. Lumbar Puncture

Participation in the CSF Substudy is optional. If the participant chooses to participate in this substudy, CSF levels of Aβ1-42, total tau, phospho-tau-181, and other CSF biomarkers will be assessed to evaluate the treatment effect of crenezumab on these markers.

m. Imaging Assessments

Radiologic evaluation will be performed using a standardized MRI protocol at screening and for safety and efficacy assessments. The data acquisition protocol includes pulse sequences for use in clinical safety evaluation performed by a central reading facility. It also includes pulse sequences for secondary and exploratory efficacy analyses and for future use by members of the research community. Dosing will not be withheld if MRI scans are delayed or missed in the case where no new or unexplained neurological symptoms are present as confirmed by the treating clinician via standard medical assessments.

Cerebral amyloid burden and the effect of crenezumab on cerebral amyloid will be assessed using 18F florbetapir PET. Regional CMRgl and the effect of crenezumab on regional CMRgl will be assessed using FDG-PET. The rationale for including 12-week FDG-PET measures is to explore short-term changes in glucose metabolism and to address potentially confounding effects unrelated to possible target engagement or possible slowing of the disease process.

Use of cannabinoids is prohibited for the 24 hours prior to any FDG-PET scan or MRI scan.

The effects of low levels of ionizing radiation on a conceived fetus are unknown; there are no known data to suggest there is a risk. It is recommended, but not required, to use effective contraception during sexual intercourse occurring within 24 hours following a male or female participant receiving a florbetapir PET or FDG-PET scan.

Specific details about the acquisition, processing, clinical interpretation, and quantitative analysis of all imaging exams will be included in the imaging charter. In addition, each modality will have a procedures manual that will be provided to the scanning centers with specific instructions for the execution of each imaging exam.

n. Cognitive, Functional, and Behavioral Assessments

In this trial, a number of cognitive, functional, and behavioral assessments will be administered; some will be administered only to the participant, some only to the study partner, and some to both. These assessments will be administered by independent, blinded raters with either a psychometrician role or a global rater role. Suicidality assessment will be performed by a clinician qualified to perform such assessments. The psychometrician and global rater will not have access to study results other than those related to the specific assessments they administer. The investigator may access any necessary study results needed for the evaluation of criteria for MCI/AD dementia.

All cognitive, functional, and behavioral assessments administered to the participant are not to be performed immediately after dosing or any potentially stressful procedures (e.g., blood draws, imaging requiring a sedative). In addition, participants should not perform these tests while fasting. For each participant, best efforts should be made to have subsequent cognitive, functional, and behavioral assessments performed at the same time of day as the baseline assessments to minimize the effect of diurnal variation in cognitive performance. Best efforts should be made for assessments of a given participant or study partner to be administered by the same psychometrician and the same global rater at each of the study visits. This will minimize measurement error due to inter-rater variability.

A Study Partner Characterization Questionnaire (SPCQ) will be completed prior to the administration of the study partner assessments at each interaction at which study partner assessments are performed. The study partner must be present at the clinic for completion of the study partner assessments at screening and at baseline (Day 1). Subsequently, best efforts should be exerted to ensure that the study partner is present at all visits requiring study partner assessments. In rare instances, when the study partner cannot be present in person, the study partner-based assessments may be administered by telephone (see Section 4.5.4).

If, during the course of the trial, the investigator determines that the participant has lost capacity to provide informed consent to participate in research, the study partner will be required to accompany the participant to visits involving the study partner. It is strongly preferred that the designated study partner is the same individual throughout the entire

study. However, if the study partner role has been reassigned, and the original study partner again becomes available, the role should be reassigned to the original study partner.

If a participant is started on prolonged therapy with a permitted medication known to affect cognitive function (e.g., antidepressants, atypical antipsychotics) after enrollment into the trial, the dose of that medication must be maintained for at least 6 weeks prior to the administration of any cognitive assessments. If a participant is started on a short-term therapy with a permitted medication known to affect cognitive function after enrollment into the trial, that medication must be stopped 2 days or 5 half-lives, whichever is longer, prior to any cognitive, functional, or behavioral assessment. Use of cannabinoids is prohibited for the 24 hours prior to any cognitive assessment.

The cognitive test batteries include a Screening Battery, the API ADAD Composite Cognitive Test Battery, and an Extended Cognitive Battery. Additional functional and behavioral assessments and other self-reported questionnaires will also be administered.

Cutoff scores for some tests may be lower than what is traditionally used in U.S. studies because of differences in population demographics. Assessment scores obtained from participants with low levels of education should be interpreted thoughtfully.

Screening Battery

The Screening Battery consists of the Word List and the MMSE. The scores obtained on these assessments will be used in determining eligibility.

API ADAD Composite Cognitive Test Battery

The API ADAD Composite Cognitive Test Battery was chosen for its sensitivity to measure clinical change in this and other at-risk populations after controlling for practice effects. The battery consists of the Word List, Multilingual Naming Test, MMSE, CERAD Constructional Praxis; and Raven's Progressive Matrices.

Extended Cognitive Test Battery

The Extended Cognitive Test Battery consists of additional neuropsychological assessments that measure verbal and non-verbal episodic memory, executive function, psychomotor speed, and other cognitive abilities. The Extended Cognitive Test Battery consists of the Free and Cued Selective Reminding Task (FCSRT), the Trail Making Test, and the Repeatable Battery for the Assessment of Neuropsychological Status (RBANS).

Other Assessments

The following other assessments will be administered to the participant:

- Subjective Memory Checklist (SMC)

- Clinical Dementia Rating (CDR)
- Geriatric Depression Scale (GDS)
- Suicidality assessment

Study Partner Assessments

The following assessments will be administered to the study partner:

- Study Partner Characterization Questionnaire (SPCQ)
- Subjective Memory Checklist (SMC)
- Clinical Dementia Rating (CDR)
- Neuropsychiatric Inventory Questionnaire (NPI)
- Functional Assessment Staging Test (FAST)

See Appendix B for complete details regarding the neurocognitive, functional, and behavioral assessments.

o. Clinical Progression

Ascertainment of AD dementia or MCI progression will be made by the investigator. Any ascertainment of AD dementia or MCI progression will be reviewed by an external adjudication committee.

For AD dementia progression ascertainment, the following will be required:

- Investigator review of medical history; interview of participant and study partner that establishes significant deterioration in the participant's cognitive and functional status since baseline; and review of the following: CDR, Word List: Recall, MMSE total, RBANS, FAST, and NPI
  - Elective review of any other clinical and cognitive test results in rendering this judgment (except for the API ADAD Composite Cognitive Test total score or the CERAD total score)
- Documentation that dementia criteria are met
- Agreement by an external adjudication committee that the above criteria have been met

For MCI progression ascertainment, the following will be required:

- Cognitive concern in the judgment of the investigator, based in part on the average Subjective Memory Checklist score $>$22 (average of participant and study partner component scores)

- Objective evidence of impairment in the judgment of the investigator in one or more cognitive domains, on the basis of review of the following: CDR, Word List: Recall, MMSE total, and RBANS
  - Elective review of any other clinical and cognitive test results in rendering this judgment (except for the API *ADAD* Composite Cognitive Test *total* score or the CERAD total score)
- Preservation of functional abilities in the judgment of the investigator, based in part on review of the FAST
- Documentation that MCI criteria have been met
- Agreement by an external adjudication committee that the above criteria have been met

p. CDR Progression

When a change from the prior visit's global CDR score is recorded, a review of that participant's CDR scoring sheets from that visit will automatically be triggered and performed by the CRO responsible for quality assurance of cognitive assessments in order to confirm that the total CDR score was derived correctly.

q. Medical History

A complete medical history should include a negative or positive history for risk factors for pneumonia including asthma, chronic obstructive pulmonary disease (COPD), heart disease, and smoking; and any other potential risk factor considered significant in the investigator's judgment.

4.5.2 Screening and Pretreatment Assessments

Participants must sign an ICF before any screening evaluations or measurements are performed. Participants must satisfy all of the inclusion criteria and none of the exclusion criteria prior to randomization. Information regarding consented participants, who are not subsequently randomized, will be kept in a screening log.

All screening evaluations must be completed within 56 days and reviewed to confirm that participants meet all eligibility criteria before the first injection of study drug. Informed consent for participation in the study must be obtained before performing any study-specific screening tests or evaluations. ICFs for participants who are not subsequently enrolled will be maintained at the study site. In addition, the randomization in IxRS will not occur until the participant's eligibility has been confirmed by the Medical Monitor or designated staff.

Participants who are found to be ineligible for entry into the study, on the basis of inclusion/exclusion criteria, will not be re-screened with the following exceptions:

- Participants who fail to meet criteria based on TSH levels may be re-screened once after initiation of treatment for the underlying disease and maintenance of treatment at a stable dose for $\geq$1 month prior to Day 1.

- Participants who fail to meet criteria based on vitamin B12 levels may be re-screened once after initiation of treatment for the underlying disease and maintenance of treatment at a stable dose for ≥ 1 month prior to Day 1.
- Participants who test positive for drugs of abuse (except cannabinoids) in their urine at screening may be re-screened one additional time.
- Participants who are ineligible because of need for medical treatment of infection or need for medical treatment of minor, non-excluded medical condition(s) diagnosed during screening, or who have minor but clinically significant blood or urine laboratory abnormalities requiring intervention (for example, stopping a medication), may be re-screened once, after the condition(s) have stabilized and after doses of treatment medications for these condition(s) have been discontinued for at least 30 days, or have been stabilized for at least 30 days.
- Participants who are ineligible because of cognitive screening results may be re-screened once, but not earlier than 3 months after the first assessment. Screening evaluations must be repeated in these participants. Screening, pretreatment tests, and evaluations will be performed within 56 days prior to Day 1 of Week 1, unless otherwise specified.
- Participants who previously withdrew consent for study participation during the screening period, but are otherwise eligible, may be re-screened once, but not earlier than 3 months after the first assessment. Screening evaluations must be repeated in these participants. Screening, pretreatment tests, and evaluations will be performed within 56 days prior to Day 1 of Week 1, unless otherwise specified.

A participant may be re-screened once, for any of the exceptions just described, at which time the exception must be addressed. Upon screening, if a different exception arises, one additional re screening is possible, at which time all exceptions must be addressed; however, a participant may be re-screened a total of two times, at most, for the exceptions listed above.

In addition to this re-screen limit, participants who are ineligible because of unavailability of a study partner may be re-screened multiple times to attempt to find an available study partner, but not earlier than 1 month after the first assessment of the most recent screening period. Screening, pretreatment tests, and evaluations will be performed within 56 days prior to Day 1 of Week 1, unless otherwise specified.

Laboratory testing, cognitive/functional assessments or imaging assessments that are repeated for administrative or technical issues (e.g., breakage of a sample vial, not evaluable images) are not considered to constitute “re-screening.” In these cases, the screening window may be extended if approved by the Medical Monitor.

Please see the study flowchart provided in Appendix A-1 for the screening and pretreatment assessments.

4.5.3 Assessments During Treatment

All assessments will be performed on the day of the specified visit unless a time window is specified. Assessments scheduled on the day of study drug administration should be performed prior to study drug injection or infusion, unless otherwise noted. For SC administration, please see the study flowcharts provided in Appendix A-1 and Appendix A-2 for the schedule of treatment assessments. For participants who decide to switch to IV dosing, the study flowcharts provided in Appendix A-3 and Appendix A-4 should be followed, starting from the visit when the first IV dose is administered.

4.5.4 Remote Assessments

In the case of COVID-19 or similar events where in-person assessments are not possible, remote assessments may be allowed (Appendix A-1 and Appendix A-2). Any assessments that could not be collected remotely should be collected in-person as soon as feasible, even if out of the protocol defined assessment window, particularly those related to the primary outcome or safety assessments.

4.5.5 Study Completion

The common-close study design requires that all participants should remain on their randomized treatment allocation until the last participant completes their Week 260 visit (subject to practical considerations regarding scheduling of final study visits that are outlined below). An end of treatment or early termination visit (ET-A or ET-B), including all assessments scheduled per study flowchart should be completed at the end of the blinded treatment period, 4 weeks after the final dose. Following the completion of the randomized, placebo-controlled, double-blinded treatment period in Study Period A, participants will be offered the opportunity to continue to receive study drug until the results of the study are known and post trial access to crenezumab is started (via an OLE or other program) or development of crenezumab is discontinued. For participants who do not choose to continue study drug in Study Period B, a final safety follow-up visit will be completed 16 weeks after the final dose.

In order to minimize site burden and to facilitate scheduling, sites will be notified at least 4 months prior to the date of the common close and as such will be able to utilize upcoming planned visits to schedule the study completion assessments. Participants who enrolled early in the study and have been in the study longer should have their study completion visits scheduled earlier where possible. The Sponsor will provide guidance to sites to facilitate scheduling when they are notified that the end of study is approaching.

Please see the study flowchart provided in Appendix A-2 or Appendix A-4 for assessments to be performed at the study completion visit (ET-A or ET-B) and the safety follow-up visit.

4.5.6 Early Termination Visit

Participants who discontinue from blinded treatment prior to completion of Study Period A (early treatment discontinuation) will be asked to stay in the study for continued safety and efficacy assessments at selected evaluation visits following completion of an early termination visit (see study flowchart in Appendix A-1, Appendix A-2, Appendix A-3, and Appendix A-4). Participants who discontinue from the study (early termination), or who discontinue from blinded treatment and elect to no longer remain in the study, will be asked to return to the clinic for an early termination visit (see the study flowchart in Appendix A-2 for participants receiving SC study drug and Appendix A-4 for participants receiving IV study drug). The early termination visit should be 4 weeks from last dose. In addition, a final safety follow-up visit should be completed 16 weeks after last dose to ensure adequate washout of study drug but may be scheduled sooner if needed.

Please see the study flowchart provided in Appendix A-2 or Appendix A-4 for assessments to be performed at the study completion/early termination visit.

Participants that are study drug non-compliant may be withdrawn from study treatment. As standard guidance, this refers to the following: Participant misses more than two doses within a single, 6-month period; a one-time exception of three missed doses within a 6-month period will be allowed. In rare circumstances (e.g., hospitalization), the Medical Monitor has the discretion to allow additional missed doses.

In order to maximize the opportunity for all active trial participants to receive the higher dose (60 mg/kg IV) of study drug, participants who have withdrawn from study treatment but remain in the study for safety and efficacy assessments will be permitted a one-time opportunity to return to the study and to receive the higher IV dose, provided they did not discontinue treatment due to an AE related to study drug, the investigator judges it is safe to restart study drug, and the participant consents to receive IV administration of study drug going forward. If the participant has not completed a complex visit within 6 months of restarting study drug, a complex visit should be completed prior to reinitiating study drug. The investigator may also order unscheduled laboratory assessments as necessary to ensure safety and appropriateness to resume study drug.

If a participant discontinues from the study (early termination or end of treatment, [ET]) or discontinues from study drug treatment and elects to no longer stay in the study, ET Visit A should be completed if the last visit occurred more than 12 weeks after functional, behavioral and cognitive assessments were performed; ET Visit B should be performed if the last visit occurred within 12 weeks of functional, behavioral and cognitive assessments. The final safety follow-up visit should be scheduled 16 weeks from last dose.

Study Period B: if a participant enters Study Period B, but wishes to stop study treatment before the end of Study Period B, a safety follow-up visit should be completed 16 weeks from the last dose.

4.5.7 Follow-Up Assessments

Participants who complete the treatment phase of the study will return to the clinic for an end of treatment visit 4 weeks after the last dose (ET-A or ET-B). Following the completion of Study Period A, participants will be offered the opportunity to continue to receive study drug until the results of the study are known and post trial access to crenezumab is started (via an OLE or other program) or development of crenezumab is discontinued. (see Section 4.5.8). For those participants that do not choose to continue study drug in Study Period B, a final safety follow-up visit will occur 16 weeks after the last dose (see Appendix A-2 or Appendix A-4).

Any AE (including a clinically significant laboratory abnormality) that is unresolved at the end of the study will continue to be followed until the event is resolved or stabilized, the participant is lost to follow up, or it has been determined that the study treatment or participation is not the cause of the event. Every effort should be made to follow all SAEs considered to be related to study drug or study procedures until a final outcome can be reported.

Please see the study flowchart provided in Appendix A-2 or Appendix A-4 for specified follow-up assessments.

4.5.8 Study Period B visits and Assessments

All assessments will be performed on the day of the specified visit unless a time window is specified. Assessments scheduled on the day of study drug administration should be performed prior to study drug injection or infusion, unless otherwise noted. Study conduct and procedures during Study Period B are the same as for Study Period A (see Section 4.5.1), though the frequency may be different. For SC administration, please see the study flowcharts provided in Appendix A-5. For IV administration, please see the study flowchart provided in Appendix A-6. If participants terminate participation in Study Period B, or Study Period B is terminated by the Sponsor due to discontinuation of the development of crenezumab, or participants complete Study Period B and do not enter an OLE or otherwise continue on crenezumab, participants will be asked to complete a final safety follow-up visit 16 weeks after their last dose of study drug (Appendix A-5 and A-6).

4.5.9 Samples for Roche and Other Clinical Repositories

4.5.9.1 Overview of the Biological Sample Repositories

Blood and CSF samples will be stored at two central sample repositories, the Roche Clinical Repository (RCR) and the National Cell Repository for Alzheimer's Disease (NCRAD). NCRAD is a national repository funded by the National Institute on Aging in the United States. The RCR and NCRAD are facilities used for the long-term storage of

human biologic specimens, including body fluids, solid tissues, and derivatives thereof (e.g., DNA, RNA, proteins, peptides). The collection and analysis of repository specimens will facilitate the rational design of new pharmaceutical agents and the development of diagnostic tests, which may allow for individualized drug therapy for patients in the future. Additionally, research on the basis of these samples may lead to an enhanced understanding of AD and related disorders.

Specimens for the biological sample repositories will be collected from participants who consent to participate in this study. Repository specimens may be used to achieve the following objectives:

- To help improve the early detection of AD
- To increase knowledge and understanding of AD biology and risk factors
- To provide a resource for genome-wide association, sequencing, epigenetic, and gene expression studies related to AD or crenezumab
- To develop biomarker or diagnostic assays for AD and related disorders and establish the performance characteristics of these assays
- To study the association of biomarkers with efficacy, AEs, or disease progression
- To study drug response, including drug effects and the processes of drug absorption and disposition

4.5.9.2 Future Research

Within 15 years after the trial is completed, the specimens in the biological sample repositories that are collected with the participants' informed consent will be made available for objectives described in Section 4.5.9.1. The implementation and use of the repository specimens is governed by a biological sample oversight committee (composed of trial leadership) in accordance with policies and procedures to ensure the appropriate use of the specimens. Sample and data sharing will be prioritized on the basis of scientific considerations and in relationship to the volume of samples available. Samples may be shared with researchers and laboratories working with Roche, Banner Alzheimer's Institute, and the University of Antioquia.

4.5.9.3 Sample Collection

The following samples will be collected for identification of dynamic (non-inherited) biomarkers to pursue the objectives described above in Section 4.5.9.1:

- Plasma and serum
- Whole blood for RNA extraction
- Cerebrospinal fluid

The following samples will be collected for identification of genetic (inherited) biomarkers to pursue the objectives described above in in Section 4.5.9.1

- Whole blood for DNA extraction

Leftover blood, serum, plasma, and CSF samples and any derivatives thereof (e.g., DNA, RNA, proteins, peptides) will be collected to pursue the objectives described above in Section 4.5.9.1.

Biological sample repository specimens will be stored for 15 years after the final study report.

The dynamic biomarker specimens will be subject to the confidentiality standards described in Section 6.10. The genetic biomarker specimens will undergo additional processes to ensure confidentiality, as described below.

4.5.9.4 Confidentiality

Given the sensitive nature of genetic data, Roche and NCRAD have implemented additional processes to ensure participant confidentiality for genetic specimens and associated data. Each specimen is "double-coded" by replacing the participant identification number with a new independent number. Data generated from the use of these specimens and all clinical data transferred from the clinical database and considered relevant are also labeled with this same independent number. A "linking key" between the participant identification number and this new independent number is stored in a secure database system. Access to the linking key is restricted to authorized individuals and is monitored by audit trail.

Data generated from RCR and NCRAD specimens must be available for inspection upon request by representatives of national and local health authorities, and Roche monitors, representatives, and collaborators, as appropriate.

Participant medical information associated with RCR and NCRAD specimens is confidential and may only be disclosed to third parties as permitted by the Informed Consent Form (or separate authorization for use and disclosure of personal health information) signed by the participant, unless permitted or required by law.

The aggregate results of any research conducted using repository samples will be available in accordance with the effective Roche policy on study data publication except where agreed.

4.5.9.5 Participation in the RCR and NCRAD

The investigator or authorized designee will explain to each participant the objectives, methods, and potential hazards of contributing samples to the sample repositories. Participants will be told that they are free to withdraw their specimens at any time and for any reason during the storage period. A separate, specific signature will be required to document a participant’s agreement to provide CSF specimens by signing the Optional CSF Substudy Informed Consent.

In the event of an RCR and NCRAD participant's death or loss of competence, the participant's specimens and data will continue to be used as part of the RCR and NCRAD research.

4.5.9.6 Withdrawal from the RCR and NCRAD

Participants have the right to withdraw their samples at any time for any reason. After withdrawal of consent, any remaining samples will be destroyed or will no longer be linked to the participant. However, if RCR samples have been tested prior to withdrawal of consent, results from those tests will remain as part of the overall research data. If a participant wishes to withdraw consent to the testing of his or her samples, the investigator must inform the Medical Monitor in writing of the participant's wishes using the Repository Subject Withdrawal Form and, if the trial is ongoing, must enter the date of withdrawal on the Repository Research Sample Withdrawal of Informed Consent eCRF. The participant will be provided with instructions on how to withdraw consent after the trial is closed. A participant's withdrawal from Study GN28352 does not, by itself, constitute withdrawal of specimens from the repositories. Likewise, a participant's withdrawal from the repositories does not constitute withdrawal from Study GN28352.

4.5.9.7 Monitoring and Oversight

Repository samples will be tracked in a manner consistent with Good Clinical Practice by a quality-controlled, auditable, and appropriately validated laboratory information management system, to ensure compliance with data confidentiality as well as adherence to authorized use of specimens as specified in this protocol and in the Informed Consent Form. Study monitors and auditors will have direct access to appropriate parts of records relating to individual participation in the repositories for the purposes of verifying the data provided. The site will permit monitoring, audits, IRB/EC review, and health authority inspections by providing direct access to source data and documents related to the repository samples.

4.6 PARTICIPANT DISCONTINUATION

Participants may withdraw consent or be withdrawn from the study at any time (see criteria below). Every effort should be made to obtain information on participants who discontinue early or are withdrawn from the study (refer to Section 4.5.6 on Early Discontinuation Visit, above). If a participant withdraws for reasons related to an SAE, every effort will be made to follow the participant until resolution or stabilization of the event. The reason for withdrawal or early discontinuation should be recorded on the appropriate eCRF page.

Reasons for withdrawal from the study may include, but are not limited to, the following:

- Participant withdrawal of consent at any time
- Any medical condition that the investigator determines may jeopardize the participant's safety if he or she continues in the study
- Investigator determines it is in the best interest of the participant

- Participant becomes pregnant

See Study Flowchart provided in Appendix A-1, Appendix A-2, Appendix A-3, and Appendix A-4 for assessments that are to be performed for participants who prematurely withdraw from the study during the treatment period (Appendix A-5 and Appendix A-6).

Participants who discontinue from the study will not be allowed to re-enter the study.

The investigator has the right to discontinue a participant from the study for any medical condition that the investigator determines may jeopardize the participant's safety if he or she continues in the study; for reasons of noncompliance (e.g., missed doses, visits); if the participant becomes pregnant; or if the investigator determines it is in the best interest of the participant.

4.7 STUDY DISCONTINUATION

Roche and Banner Alzheimer's Institute have the right to terminate this study at any time. Reasons for terminating the study may include, but are not limited to, the following:

- The incidence or severity of AEs in this or other studies indicates a potential health hazard to participants.
- Participant enrollment is unsatisfactory.
- Data recording is inaccurate or incomplete.

4.8 POST-TRIAL ACCESS TO STUDY DRUG

The Sponsor will offer post-trial access to the study drug (crenezumab) free of charge to eligible participants in accordance with the Roche Global Policy on Continued Access to Investigational Medicinal Product, as outlined below.

A participant will be eligible to receive study drug after completing the Study if all of the following conditions are met:

- The participant has a life-threatening or severe medical condition and requires continued study drug treatment for his or her well-being.
- There are no appropriate alternative treatments available to the participant.
- The participant and his or her doctor comply with and satisfy any legal or regulatory requirements that apply to them.

A participant will not be eligible to receive study drug after completing the study if any of the following conditions are met:

- The study drug is commercially marketed in the participant's country and is reasonably accessible to the participant (e.g., is covered by the participant's insurance or wouldn't otherwise create a financial hardship for the participant).
- The Sponsor has discontinued development of the study drug or data suggest that the study drug is not effective for ADAD.

- The Sponsor has reasonable safety concerns regarding the study drug as treatment for AD or ADAD.
- Provision of study drug is not permitted under the laws and regulations of the participant's country.

The Roche Global Policy on Continued Access to Investigational Medicinal Product is available at the following Web site:

http://www.roche.com/policy_continued_access_to_investigational_medicines.pdf

Roche does not have a specific plan to provide crenezumab or other study interventions to participants who withdraw from the study early (prior to completion of Study Period A). Participation in, or completion of, Study Period B is not required.

4.9 ASSAY METHODS

- Total serum levels of crenezumab will be measured using a validated ELISA.
- ADAs in serum will be measured using a validated bridging ELISA.
- Total plasma levels of Aβ1-40 and Aβ1-42 will be measured using qualified assays.
- Levels of Aβ1-42, phospho-tau-181, and total tau will be measured in CSF using qualified assays.

4.10 STATISTICAL METHODS

Analyses planned for the study include the following:

- An interim analysis of the cohort of carriers may occur at the Sponsor's discretion.
- A primary analysis will occur after all participants have completed the Week 260 assessment, i.e., at the end of Study Period A.

4.10.1 Analysis of the Conduct of the Study

The number of participants who enroll, discontinue from treatment, discontinue from study, and complete the study will be tabulated by treatment group. Reasons for early discontinuation of treatment or early termination from the study will be listed and summarized by treatment group. Any eligibility criteria and other major protocol deviations will also be summarized by treatment group.

4.10.2 Analysis of Treatment Group Comparability

Demographic and baseline characteristics such as age, sex, race, *APOE4* status, and baseline cognitive scores will be summarized for all randomized participants by treatment group using descriptive statistics.

4.10.3 Efficacy Analyses

Primary efficacy analysis *for the primary endpoint family* in Study Period A will be based on a mITT population. The mITT population is defined as participants who are

mutation carriers treated with any amount of study drug. Participants in the efficacy analysis population will be grouped based on treatment assigned at randomization.

If the Sponsor decides to initiate an OLE study, efficacy data from Study Period B could be used in the OLE Study to contribute to delayed start analyses *and time-to-event analyses.*

a. Primary Efficacy Endpoints

The primary efficacy analysis will be performed on the mITT population. The primary endpoint *family*, *1)* annualized rate of change on the API ADAD Composite Cognitive Test *Battery* total score *and 2) annualized rate of change on the FCSRT Cueing Index*, will be assessed using a mixed effects (ME) model. The model will be used to test the treatment effect on annualized rate of change adjusting for selected baseline characteristics and randomization factors. Statistical tests for the primary endpoint*s* will be two-sided and performed at *an overall* 0.05 level*: α=0.04 for testing the treatment effect on the annualized rate of change on the API ADAD Composite Cognitive Test Battery total score; alpha=0.01 for testing the treatment effect on the annualized rate of change on the FCSRT Cueing Index.* Estimated treatment effects and confidence intervals will also be provided as an aid to interpretation of study results.

In order to maximize the power, data beyond each participant's Week 260 assessment through the unblinding of the study will be included in the primary analysis or its sensitivity analysis.

All details of the prespecified statistical analyses for the primary efficacy endpoints will be provided in the SAP.

b. Secondary Efficacy Endpoints

The final selection of secondary efficacy endpoints will be on the basis of baseline data from the study population, ongoing clinical trials, and other external data that will emerge prior to database unblinding. The selected secondary endpoints will be provided in the SAP to be submitted prior to database unblinding. Secondary endpoints will be selected from the following:

Clinical

- Time to progression to MCI due to AD (Albert et al. 2011) or dementia due to AD (McKhann et al. 2011) (see Section 4.5.1. for criteria)
- Time to progression to non-zero in the CDR global score (Morris 1993)
- Annualized rate of change in CDR-SOB (Morris 1993)
- Annualized rate of change in a measure of overall neurocognitive functioning: Repeatable Battery for the Assessment of Neuropsychological Status (RBANS) (Randolph 1998)

Imaging Biomarkers

- Annualized rate of change in mean cerebral fibrillar amyloid accumulation using florbetapir PET
- Annualized rate of change in regional CMRgl using FDG-PET
- Annualized rate of change in volumetric measurements using MRI

CSF Biomarkers

- Annualized rate of change in a tau-based CSF biomarker

Imaging biomarkers, CSF biomarkers, and CDR-SOB will be analyzed using mixed effects models in the same manner as the primary efficacy outcome measure. The time to progression outcome measures (time to MCI progression and time to non-zero in the CDR global score) will be analyzed using survival analysis methods.

Estimated treatment effects, confidence intervals and p-values will be provided for all selected secondary endpoints. Details of prespecified statistical analyses of the secondary efficacy endpoints will be provided in the SAP.

4.10.4 Safety Analysis

The safety analysis population in Study Period A will consist of all carrier participants with at least one dose of study medication. The participants in this population will be grouped according to treatment received.

If the Sponsor decides to initiate an OLE study, safety data from Study Period B will also be used to contribute to the safety analysis in the OLE study. In this case, the safety analysis population in Study Period B will be grouped according to the treatment received in Study Period B. Safety assessments will be on the basis of AE reports, the results of vital signs, physical examinations, MRI evaluations, immunogenicity as measured by ADAs, and clinical laboratory tests.

Observed toxicities and AEs will be coded and tabulated using the Medical Dictionary for Regulatory Activities (MedDRA); the frequencies of participants who receive active medication and those who receive placebo will be compared using descriptive statistics and reviewed for potential clinical significance and importance. Treatment-emergent AE tabulations will be provided overall, by severity, for serious AEs, and further subcategorized by relationship to study drug.

4.10.4.1 Adverse Events

AEs will be recorded from the time informed consent is given until 16 weeks following the last administration of any investigational product, or a participant completes the study or discontinues prematurely, whichever occurs last (see Section 5.2.1 for details). AEs reported during the screening period, the treatment period in Study Period A, and the 16-week follow-up period will be summarized separately. Verbatim descriptions of AEs will be coded and analyzed using appropriate thesaurus terms. A

treatment-emergent AE is defined as any AE reported on or after the first dose of study drug.

AEs occurring during Study Period B will be summarized in the final CSR.

4.10.4.2 Clinical Laboratory Evaluations

Clinical laboratory data (serum chemistry, hematology evaluations including CBC with differential and platelet counts, and urinalysis values) and ECG data will be summarized over time by descriptive statistics by treatment group.

4.10.4.3 MRI Evaluations

Neuroradiologic evaluation with respect to the occurrence of cerebral VE, superficial siderosis of the CNS, and cerebral micro- or macrohemorrhages will be performed during the treatment period and will be summarized by treatment group using descriptive statistics.

4.10.4.4 Vital Signs

Vital signs (pulse rate, blood pressure, body temperature, and respiratory rate) and changes from baseline will be summarized by treatment group.

4.10.4.5 ECGs

ECG data and changes from baseline for relevant ECG intervals will be summarized by treatment group.

4.10.4.6 Anti-Drug Antibodies

The number and percentage of participants with confirmed positive ADAs will be reported for each treatment group.

4.10.5 Pharmacokinetic and Pharmacodynamic Analyses

Mean serum and CSF crenezumab concentration versus time data will be tabulated and plotted. In serum, $C_{trough,ss}$ will be tabulated and summarized (e.g., mean, standard deviation, minimum, and maximum). Additional PK analyses may be conducted as appropriate.

Mean plasma total Aβ1-40 and Aβ1-42 concentrations vs. time will be tabulated and plotted. The relationship between crenezumab and total Aβ1-40 and Aβ1-42 concentrations will be tabulated and plotted to explore the peripheral PK/PD relationship.

The schedule for PK and PD analyses will be independent of the safety and efficacy endpoints.

4.10.6 Exploratory Analyses

The exploratory efficacy outcome measures (see Section 3.3.5) will be analyzed in a similar manner to the secondary efficacy outcome measures. Details of prespecified statistical analyses of the exploratory endpoints will be provided in the SAP.

4.10.7 Handling of Missing Data

All efforts will be made to minimize missing data. Missing values will not be imputed since mixed effects models are robustly capable of handling missing data when data are missing at random. Various sensitivity analyses to support the primary analysis will use alternative methods of handling missing values. Details will be provided in the SAP.

4.10.8 Determination of Sample Size

This study will enroll up to 300 participants: up to 200 participants will be enrolled in the carrier cohort and up to 100 participants will be enrolled in the non-carrier cohort.

Participants in the carrier cohort will be randomized in a 1:1 randomization ratio of active to placebo participants. The study *was originally* powered to compare the mean change from baseline over 260 weeks in the API ADAD Composite Cognitive Test total score between the active group and the placebo. Assuming a 25% dropout rate, two-sided testing at *an* overall 0.05 level, a placebo group CV of 65% for the Week 260 change scores (= 100% × standard deviation of placebo participant change scores/mean of placebo participant change scores) and 100 participants per arm, the study will have at least 80% power to detect a true effect of 30% reduction of the mean decline in the placebo group.

Participants in the non-carrier cohort will all receive placebo and will be included in the study in order to maintain the genotype blind.

The study was originally powered based on the API ADAD Composite Cognitive Test Battery total score. However, due to concerns about learning effects and general lack of sensivity to change in a cognitively normal population, before study unblinding, the Sponsor performed a blinded power analysis on the original primary endpoint API ADAD Composite Cognitive Test Battery Total Score and the secondary clinical endpoints. The Sponsor decided to promote the FCSRT Cueing Index from a secondary endpoint to part of a primary endpoint family (along with the API ADAD Composite) due to its favorable statistical properties and clinical relevance as a sensitive measure of episodic memory dysfunction-- the hallmark of emerging symptomatic AD and the most commonly observed, early, and consistent neuropsychological marker of AD.(Tounsi et al. 1999; Caselli et al. 2020; Bateman et al. 2012) In the statistical analysis plan (SAP), the Sponsor will provide details of the blinded power analysis along with the evidence of type I error control.

4.10.9 Interim Analysis

To adapt to information that may emerge during the course of this study, the Sponsor may choose to conduct one or more interim analyses. Sites are to be informed prior to an interim analysis. Below are the specifications to ensure the study continues to meet the highest standards of integrity when an optional interim analysis is executed.

If an interim analysis is conducted, the Sponsor will remain blinded. The interim analysis will be conducted by an external statistical group and reviewed by the iDMC. Interactions between the iDMC and Sponsor will be carried out as specified in the iDMC Charter.

The decision to conduct the optional interim analysis, along with the rationale, timing, and statistical details for the analysis, will be documented in the SAP. The iDMC Charter will be updated to document potential recommendations the iDMC can make to the Sponsor as a result of the analysis (e.g., stop the study for positive efficacy, stop the study for futility), and the iDMC Charter will also be made available to relevant health authorities.

If there is a potential for the study to be stopped for positive efficacy as a result of the interim analysis, the type I error rate of the primary analysis will be controlled to ensure statistical validity is maintained. If the study continues beyond the interim analysis, the critical value at the final analysis would be adjusted accordingly to maintain the protocol-specified overall type I error rate, per standard Lan-DeMets methodology.

4.11 DATA QUALITY ASSURANCE

The data will be collected via Electronic Data Capture (EDC) using eCRFs. The site will be responsible for data entry into the EDC system. In the event of discrepant data, the Sponsor or the clinical research organization (CRO) will request data clarification from the sites, which the sites will resolve electronically in the EDC system.

Banner and Roche will perform oversight of the data management of this trial. Roche will produce an EDC Study Specification document that describes the quality checking to be performed on the data. Data from the central laboratory and central imaging reader will be sent directly to Roche, using Roche's standard procedures to handle and process the electronic transfer of these data.

The eCRFs and correction documentation will be maintained in the EDC system's audit trail. System backups for data stored at Roche and records retention for the study data will be consistent with the Roche's standard procedures.

5. ASSESSMENT OF SAFETY

5.1 SAFETY PARAMETERS AND DEFINITIONS

Safety assessments will consist of monitoring and recording AEs, SAEs, and AESI; measurement of protocol-specified hematology, clinical chemistry, and urinalysis variables; measurement of protocol-specified vital signs; and other protocol-specified tests that are deemed critical to the safety evaluation of the study drug(s).

Roche or its designee is responsible for reporting relevant SAEs to the Competent Authority, other applicable regulatory authorities, and participating investigators, in

accordance with ICH guidelines, FDA regulations, European Clinical Trials Directive (Directive 2001/20/EC), and/or local regulatory requirements.

Roche or its designee is responsible for reporting unexpected fatal or life-threatening events associated with the use of the study drug to the regulatory agencies and competent authorities by telephone or fax within 7 calendar days after being notified of the event. Roche or its designee will report other relevant SAEs associated with the use of the study medication to the appropriate competent authorities (according to local guidelines), investigators, and central IRBs/ECs (except in the United States where investigators are responsible for reporting to their IRBs per local requirements) by a written safety report within 15 calendar days of notification.

5.1.1 Adverse Events

An AE is any unfavorable and unintended sign, symptom, or disease temporally associated with the use of an investigational product or other protocol-imposed intervention, regardless of attribution. For this protocol, all products not approved for marketing in Colombia will be considered an investigational product. This includes PET tracers.

This includes the following:

- AEs not previously observed in the participant that emerge during the protocol-specified AE reporting period
- Complications that occur as a result of protocol-mandated interventions (e.g., invasive procedures such as biopsies)
- AEs that occur prior to assignment of the first study investigational product (study drug and/or PET ligand) that are related to a protocol-mandated intervention (e.g., invasive procedures such as biopsies, medication washout, or no treatment run-in). See Section 5.2.1 for reporting requirements for these AEs.
- Preexisting medical conditions, other than AD, judged by the investigator to have worsened in severity or frequency or changed in character during the protocol-specified AE reporting period

Unchanged, chronic conditions that are present at baseline are NOT AEs and should not be recorded on the Adverse Event eCRF. An exacerbation or worsening of a chronic condition should be recorded as an AE. A preexisting condition should be recorded on the General Medical History and Baseline Conditions eCRF.

5.1.2 Serious Adverse Event

An SAE is any AE that meets any of the following:

- Fatal (i.e., the AE actually causes or leads to death)
- Life threatening (i.e., the AE, in the view of the investigator, places the participant at immediate risk of death)

- Requires or prolongs inpatient hospitalization
- Results in persistent or significant disability/incapacity (i.e., the AE results in substantial disruption of the participant's ability to conduct normal life functions)
- A congenital anomaly/birth defect in a neonate/infant born to a mother exposed to the investigational product(s)
- Considered a significant medical event by the investigator (e.g., may jeopardize the participant or may require medical/surgical intervention to prevent one of the outcomes listed above)

All AEs that do not meet any of the criteria for serious should be regarded as **non-serious AEs**.

The terms "severe" and "serious" are not synonymous. Severity refers to the intensity of an AE (as in mild, moderate, or severe pain); the event itself may be of relatively minor medical significance (such as severe headache). "Serious" is a regulatory definition and is on the basis of participant or event outcome or action criteria usually associated with events that pose a threat to a participant's life or vital functions. Seriousness (not severity) serves as the guide for defining regulatory reporting obligations.

Severity and seriousness should be independently assessed when recording AEs and SAEs on the eCRF.

5.1.3 Protocol-Defined Events of Special Interest/Non-Serious Expedited Adverse Events

The following events are events of special interest and will need to be reported to the Sponsor expeditiously (i.e., no more than 24 hours after learning of the event; see Section 5.4 for reporting instructions), irrespective of regulatory seriousness criteria:

- ARIA-E (cerebral VE or sulcal effusion) (see Section 3.4.2)
- ARIA-H
  - Superficial siderosis of the CNS (see Section 3.4.2)
  - Cerebral microhemorrhages (see Section 3.4.2)
- Macrohemorrhages (see Section 3.4.2)
- Occurrence of pneumonia verified by imaging (e.g., chest X-ray) (see Section 3.4.3)
- The finding of an elevated ALT or AST $>3\times$ ULN in combination with either an elevated total bilirubin ($>2\times$ ULN) or clinical jaundice in the absence of cholestasis or other causes of hyperbilirubinemia is considered to be an indicator of severe liver injury. Therefore, investigators must report as an AE cases of potential drug-induced liver injury that include the occurrence of either of the following:
  - Treatment-emergent ALT or AST $>3\times$ ULN in combination with total bilirubin $>2\times$ ULN
  - Treatment-emergent ALT or AST $>3\times$ ULN in combination with clinical jaundice

- Suspected transmission of an infectious agent by the investigational product, as defined below
  - Any organism, virus, or infectious particle (e.g., prion protein transmitting transmissible spongiform encephalopathy), pathogenic or non-pathogenic, is considered an infectious agent. A transmission of an infectious agent may be suspected from clinical symptoms or laboratory findings that indicate an infection in a participant exposed to a medicinal product. This term applies only when a contamination of the study drug is suspected.

5.2 METHODS AND TIMING FOR CAPTURING AND ASSESSING SAFETY PARAMETERS

The investigator is responsible for ensuring that all AEs and SAEs (as defined in Section 5.1) are recorded on the eCRF and reported to the Sponsor in accordance with protocol instructions.

5.2.1 Adverse Event Reporting Period

After informed consent, but prior to the first administration of any of the investigational products (study drug and/or PET ligand), only SAEs caused by a protocol-mandated intervention will be collected (e.g., SAEs related to invasive procedures such as biopsies, medication washout, or no treatment run-in). During this period, non-serious AEs are not collected. If a non-serious AE occurs during this period and continues to Day 1, the event can then be recorded as a baseline condition.

After initiation of the first investigational product (study drug and/or PET ligand), all AEs and SAEs, regardless of attribution, will be collected until 16 weeks following the last administration of any investigational product (study drug and/or PET ligand) or study discontinuation/termination, whichever is later. After this 16-week follow-up period, investigators should report only SAEs that are considered to be related to a previously administered investigational product (see Section 5.6).

5.2.2 Eliciting Adverse Events

A consistent methodology of non-directive questioning for eliciting AEs at all participant evaluation time points should be adopted. Examples of non-directive questions include:

“How have you felt since your last clinic visit?”

“Have you had any new or changed health problems since you were last here?”

5.2.3 Assessment of Severity and Causality of Adverse Events

Investigators will seek information on AEs and SAEs at each participant contact. All AEs and SAEs, whether reported by the participant or noted by authorized study personnel, will be recorded in the participant’s medical record and on the Adverse Event eCRF.

For each AE and SAE recorded on the applicable eCRF, the investigator will make an assessment of seriousness (see Section 5.1.2 for seriousness criteria), severity and causality.

Table 1 provides guidance for grading AE severity, and Table 2 provides guidance for assessing the causal relationship to the investigational product(s).

The AE grading (severity) scale found in the NCI CTCAE (Version 4.0) will be used for assessing AE severity.

Table 1 Adverse Event Grading (Severity) Scale

Grade	Severity	Alternate Description [a]
1	Mild (apply event-specific NCI CTCAE grading criteria)	Asymptomatic or mild symptoms; clinical or diagnostic observations only; intervention not indicated
2	Moderate (apply event-specific NCI CTCAE grading criteria)	Minimal, local, or non-invasive intervention indicated; limiting age-appropriate instrumental ADL [b]
3	Severe (apply event-specific NCI CTCAE grading criteria)	Severe or medically significant but not immediately life threatening; hospitalization or prolongation of hospitalization indicated; disabling; limiting self-care ADL [c]
4	Very severe, life threatening, or disabling (apply event-specific NCI CTCAE grading criteria)	Life-threatening consequences; urgent intervention indicated
5	Death related to AE	

ADL = activities of daily living; AE = adverse event; CTCAE = Common Terminology Criteria for Adverse Events; NCI = National Cancer Institute.

The NCI CTCAE (Version 4.0) can be found at: http://ctep.cancer.gov/protocolDevelopment/electronic_applications/ctc.htm#ctc_40

Note: Regardless of severity, some events may also meet regulatory seriousness criteria. Refer to definition of a serious AE (see Section 5.1.2).

[a] Use these alternative definitions for Grade 1, 2, 3, and 4 events when the observed or reported AE is not in the NCI CTCAE listing.

[b] Instrumental ADL refers to preparing meals, shopping for groceries or clothes, using the telephone, managing money, etc.

[c] Self-care ADL refer to bathing, dressing and undressing, feeding self, using the toilet, taking medications, and not bedridden.

To ensure consistency of causality assessments, investigators should apply the general guidelines in Table 2.

Table 2 Causal Attribution Guidance

**Is the AE/SAE suspected to be caused by the investigational product on the basis of facts, evidence, science-based rationales, and clinical judgment?**	
YES	There is a plausible temporal relationship between the onset of the AE and administration of the investigational product, and the AE cannot be readily explained by the participant's clinical state, intercurrent illness, or concomitant therapies; and/or the AE follows a known pattern of response to the investigational product; and/or the AE abates or resolves upon discontinuation of the investigational product or dose reduction and, if applicable, reappears upon re-challenge.<br>**Investigators should apply facts, evidence, or rationales on the basis of scientific principles and clinical judgment to support a causal/contributory association with an investigational product.**
NO	**AEs will be considered related, unless they fulfill the criteria as specified below.**<br>Evidence exists that the AE has an etiology other than the investigational product (e.g., preexisting medical condition, underlying disease, intercurrent illness, or concomitant medication); and/or the AE has no plausible temporal relationship to administration of the investigational product (e.g., cancer diagnosed 2 days after first dose of study drug).<br>Note: The investigator's assessment of causality for individual AE reports is part of the study documentation process. Regardless of the "Yes" or "No" causality assessment for individual AE reports, the Sponsor will promptly evaluate all reported SAEs against cumulative product experience to identify and expeditiously communicate possible new safety findings to investigators and applicable regulatory authorities. **Attribution of SAEs will be reviewed on an ongoing basis, and may be changed as additional clinical data emerges (e.g., reversibility of AE, new clinical findings in participant with AE, effects of retreatment, AEs in other participants)**.

5.3 PROCEDURES FOR RECORDING ADVERSE EVENTS

5.3.1 Recording Adverse Events on the eCRF

Investigators should use correct medical terminology/concepts when recording AEs or SAEs on the eCRF. Avoid colloquialisms and abbreviations.

There is one eCRF page for recording AEs or SAEs.

Only one medical concept should be recorded in the event field on the Adverse Event eCRF.

a) Diagnosis Versus Signs and Symptoms

If known, a diagnosis should be recorded on the eCRF rather than individual signs and symptoms (e.g., record only liver failure or hepatitis rather than jaundice, asterixis, and elevated transaminases). However, if a constellation of signs and/or symptoms cannot be medically characterized as a single diagnosis or syndrome at the time of reporting, each individual event should be recorded as an AE or SAE on the eCRF. If a diagnosis is subsequently established, it should be reported as follow-up information.

Diagnoses for ARIA-E, ARIA-H, and cerebral macrohemorrhage will be made on the basis of MRI by the central reader.

b) Adverse Events Occurring Secondary to Other Events

In general, AEs occurring secondary to other events (e.g., cascade events or clinical sequelae) should be identified by their primary cause. For example, if severe diarrhea is known to have resulted in dehydration, it is sufficient to record only diarrhea as an AE or SAE on the eCRF.

However, medically significant AEs occurring secondary to an initiating event that are separated in time should be recorded as independent events on the eCRF. For example, if a severe gastrointestinal hemorrhage leads to renal failure, both events should be recorded separately on the eCRF.

c) Persistent or Recurrent Adverse Events

A persistent AE is one that extends continuously, without resolution between participant evaluation time points. Such events should only be recorded once in the eCRF unless their severity increases. If a persistent AE becomes more severe, it should be recorded again on the Adverse Event eCRF. The initial (less severe) adverse event report should be updated to indicate that the event resolved on the date just prior to the day the event became more severe. If a persistent AE becomes serious, it should then be recorded as a separate event on the Adverse Event eCRF and reported to the Sponsor immediately (i.e., no more than 24 hours after learning that the event became serious; see Section 5.4.3 for reporting instructions). The initial (non-serious) adverse event report should be updated to indicate that the event resolved on the date just prior to the day the event became serious). If the severity of the previously reported SAE changes, it should be reported as follow-up to the previous SAE, not as a new event.

A recurrent AE is one that occurs and resolves between participant evaluation time points and subsequently recurs. All recurrent AEs should be recorded on the Adverse Event eCRF.

d) Abnormal Laboratory Values

Only clinically significant laboratory abnormalities that require active management will be recorded as AEs or SAEs on the eCRF (e.g., abnormalities that require investigational product dose modification, discontinuation of study treatment, more frequent follow-up assessments, further diagnostic investigation, etc.).

If the clinically significant laboratory abnormality is a sign of a disease or syndrome (e.g., alkaline phosphatase and bilirubin 5 × the ULN associated with cholecystitis), only the diagnosis (e.g., cholecystitis) needs to be recorded on the Adverse Event eCRF.

If the clinically significant laboratory abnormality is not a sign of a disease or syndrome, the abnormality itself should be recorded as an AE or SAE on the eCRF. If the laboratory abnormality can be characterized by a precise clinical term, the clinical term should be recorded as the AE or SAE. For example, an elevated serum potassium level of 7.0 mEq/L should be recorded as “hyperkalemia.”

Observations of the same clinically significant laboratory abnormality from visit to visit should not be repeatedly recorded as AEs or SAEs on the eCRF, unless their severity, seriousness, or etiology changes.

e) Deaths

All deaths that occur during the protocol-specified AE reporting period (see Section 5.2.1), regardless of attribution, will be recorded on an eCRF and expeditiously reported to the Sponsor. This includes death attributed to progression of Alzheimer’s disease.

When recording a death, the event or condition that caused or contributed to the fatal outcome should be recorded as the single medical concept on the eCRF. If the cause of death is unknown and cannot be ascertained at the time of reporting, record “Unexplained Death” on the eCRF.

If the death is attributed to progression of Alzheimer’s disease, record Alzheimer’s disease Progression” as the SAE term on the eCRF.

f) Preexisting Medical Conditions

A preexisting medical condition is one that is present at the start of the study. Such conditions should be recorded on the Medical and Surgical History eCRF.

A preexisting medical condition should be recorded as an AE or SAE only if the frequency, severity, or character of the condition worsens during the study. When recording such events on an Adverse Event eCRF, it is important to convey the concept that the preexisting condition has changed by including applicable descriptors (e.g., “more frequent headaches”).

g) **Development or Worsening of Alzheimer's Disease**

Emergence of cognitive symptoms and/or functional impairment during the study or other events that are clearly consistent with the expected pattern of progression of Alzheimer's disease should not be recorded as AEs. These data will be captured as efficacy assessment data only. In most cases, the expected pattern of progression will be on the basis of established criteria for MCI (Albert et al. 2011) or dementia (McKhann et al. 2011). If there is any uncertainty as to whether an event is due to disease progression, or if the progression is unexpected or thought to be treatment related, it should be reported as an AE.

h) **Hospitalization, Prolonged Hospitalization, or Surgery**

Any AE that results in hospitalization or prolonged hospitalization should be documented and reported as an SAE unless specifically instructed otherwise in this protocol.

There are some hospitalization scenarios that do not require reporting as an SAE when there is no occurrence of an AE. These scenarios include a planned hospitalization or prolonged hospitalization to:

- Perform an efficacy measurement for the study
- Undergo a diagnostic or elective surgical procedure for a preexisting medical condition that has not changed
- Receive scheduled therapy for the target disease of the study

i) **Pregnancy**

If a female participant becomes pregnant while receiving investigational therapy or within 16 weeks after the last dose of investigational product (crenezumab/placebo or florbetapir), a Pregnancy Report eCRF should be completed within 24 hours of learning of the pregnancy. A pregnancy report will automatically be generated and sent to Roche Drug Safety by the EDC system. Pregnancy should not be recorded on the Adverse Event eCRF. In the event the EDC system is unavailable, a paper Pregnancy Report form and Pregnancy Fax Coversheet should be completed and faxed to Roche's Drug Safety Department, or its designee, at the fax numbers listed in Section 5.4.3.

j) **Pregnancies in Female Partners of Male Participants**

Male participants will be instructed through the Informed Consent Form to immediately inform the investigator if their partner becomes pregnant after the first dose of either crenezumab/placebo or florbetapir has been administered or within 8 weeks after the last dose of crenezumab/placebo or florbetapir has been administered. A Pregnancy Report eCRF should be completed by the investigator immediately (i.e., no more than 24 hours after learning of the pregnancy) and submitted via the EDC system. Attempts should be made to collect and report details of the course and outcome of any pregnancy in the partner of a male participant exposed to investigational product. The pregnant partner will need to sign an Authorization for Use and Disclosure of Pregnancy

Health Information to allow for follow-up on her pregnancy. Once the authorization has been signed, the investigator will update the Pregnancy Report eCRF with additional information on the course and outcome of the pregnancy. An investigator who is contacted by the male participant or his pregnant partner may provide information on the risks of the pregnancy and the possible effects on the fetus, to support an informed decision in cooperation with the treating physician and/or obstetrician. In the event that the EDC system is unavailable, follow reporting instructions provided in Section 5.3.1.i.

k) Congenital Anomalies/Birth Defects

Any congenital anomaly/birth defect in a child born to a female participant exposed to study drug or the female partner of a male participant exposed to study drug should be classified as an SAE, recorded on the Adverse Event eCRF, and reported to the Sponsor immediately (i.e., no more than 24 hours after learning of the event; see Section 5.4.3).

l) Abortions

A spontaneous abortion should be classified as an SAE (as the Sponsor considers abortions to be medically significant), recorded on the Adverse Event eCRF, and reported to the Sponsor immediately (i.e., no more than 24 hours after learning of the event; see Section 5.4.3).

If a therapeutic or elective abortion was performed because of an underlying maternal or embryofetal toxicity, the toxicity should be classified as an SAE, recorded on the Adverse Event eCRF, and reported to the Sponsor immediately (i.e., no more than 24 hours after learning of the event; see Section 5.4.3). A therapeutic or elective abortion performed for reasons other than an underlying maternal or embryofetal toxicity is not considered an AE.

All abortions should be reported as pregnancy outcomes on the paper Clinical Trial Pregnancy Reporting Form.

5.4 EXPEDITED REPORTING REQUIREMENTS FOR SERIOUS ADVERSE EVENTS (AND PROTOCOL-DEFINED EVENTS OF SPECIAL INTEREST)

5.4.1 Reporting Requirements for Fatal/Life-Threatening Serious Adverse Events Related to Investigational Product

Any life-threatening (i.e., imminent risk of death) or fatal AE that is attributed by the investigator to the investigational product will be telephoned to the Medical Monitor immediately, followed by submission of written case details on the SAE reporting form within 24 hours as described in Section 5.4.3.

5.4.2 Emergency Medical Contacts

PRA Medical Monitor Contact No.:	+1 (866) 326-5053 (24 hours)
Alternative Medical Monitor:	[redacted], M.D., M.A.S.
Mobile Telephone No.:	[redacted]
Fax Telephone No.:	

To ensure the safety of study participants, an Emergency Medical Call Center Help Desk will access the Roche Medical Emergency List, escalate emergency medical calls, provide medical translation service (if necessary), connect the investigator with a Roche Medical Responsible (listed above and/or on the Roche Medical Emergency List), and track all calls. The Emergency Medical Call Center Help Desk will be available 24 hours per day, 7 days per week. Toll-free numbers for the Help Desk, as well as Medical Monitor and Medical Responsible contact information, will be distributed to all investigators.

5.4.3 Reporting Requirements for All SAEs and Protocol-Defined Events of Special Interest

Investigators will submit reports of all SAEs and the Protocol-Defined Events of Special Interest/Non-Serious Expedited Adverse Events (see Section 5.1.3), regardless of attribution, to Roche within 24 hours of learning of the events. For initial SAE and protocol-defined events of special interest reports, investigators should record all case details that can be gathered within 24 hours on an eCRF and submit the report via the EDC system. A report will be generated and sent to Roche’s Drug Safety by the EDC system.

In the event the EDC system is unavailable, a completed Serious/Non-Serious Expedited Adverse Event paper reporting form and fax coversheet should be faxed immediately upon completion to Roche’s Drug Safety Department, or its designee, at the fax numbers indicated below. Once the EDC system is available, all information will need to be entered and submitted via the EDC system.

Fax No.: 1 (888) 772-6919

Relevant follow-up information should be submitted to Roche’s Drug Safety Department or its designee as soon as it becomes available and/or upon request.

5.5 TYPE AND DURATION OF FOLLOW-UP OF PARTICIPANTS AFTER ADVERSE EVENTS

The investigator should follow all unresolved AEs and SAEs until the events are resolved or stabilized, the participant is lost to follow-up, or it has been determined that the investigational product or participation is not the cause of the AE/SAE. Resolution of AEs and SAEs (with dates) should be documented on the Adverse Event eCRF and in the participant’s medical record to facilitate source data verification (SDV).

For some SAEs, the Sponsor or its designee may follow up by telephone, fax, electronic mail, and/or a monitoring visit to obtain additional case details deemed necessary to appropriately evaluate the SAE report (e.g., hospital discharge summary, consultant report, or autopsy report).

5.6 POST-STUDY ADVERSE EVENTS

At the last scheduled visit, the investigator should instruct each participant to report to the investigator any subsequent SAEs that the participant's personal physician believes could be related to prior investigational product treatment.

The investigator should notify the study Sponsor of any death or other SAE occurring at any time after a participant has discontinued or terminated study participation if considered to be related to prior investigational product treatment. The Sponsor should also be notified if the investigator should become aware of the development of cancer or of a congenital anomaly in a subsequently conceived offspring of a participant that participated in this study. The investigator should report these events to Roche's Drug Safety on the study eCRF.

If the study eCRF is no longer available, the investigator should report the event directly to the Roche Colombian Affiliate via phone at +57 4178860.

5.7 EXPEDITED REPORTING TO HEALTH AUTHORITIES, INVESTIGATORS, INSTITUTIONAL REVIEW BOARDS, AND ETHICS COMMITTEES

The Sponsor will promptly evaluate all SAEs and AEs of special interest against cumulative product experience to identify and expeditiously communicate possible new safety findings to investigators, IRBs, ECs, and applicable health authorities based on applicable legislation.

To determine reporting requirements for single adverse event cases, the Sponsor will assess the expectedness of these events through use of the reference safety information in the documents listed below:

Drug	Document
Crenezumab	Crenezumab Investigator's Brochure
Florbetapir	Florbetapir Investigator's Brochure
GTP1	GTP1 Investigator's Brochure

The Sponsor will compare the severity of each event and the cumulative event frequency reported for the study with the severity and frequency reported in the applicable reference document.

Reporting requirements will also be based on the investigator's assessment of causality and seriousness, with allowance for upgrading by the Sponsor as needed.

6. INVESTIGATOR REQUIREMENTS

6.1 STUDY INITIATION

Before the start of this study and any study-related procedures at a specific site, the following documents must be on file with Roche or a Roche representative:

- U.S. FDA Form 1572 for each site (for all studies conducted under U.S. Investigational New Drug [IND] regulations), signed by the Principal Investigator
  - The names of any subinvestigators must appear on this form. Investigators must also complete all regulatory documentation as required by local and national regulations.
- Current curricula vitae and evidence of licensure of the Principal Investigator and all subinvestigators
- Complete financial disclosure forms for the Principal Investigator and all subinvestigators listed on the U.S. FDA Form 1572
- Federalwide Assurance number or IRB statement of compliance
- Written documentation of IRB/EC approval of the protocol (identified by protocol number or title and date of approval) and Informed Consent Form (identified by protocol number or title and date of approval)
- A copy of the IRB/EC-approved Informed Consent Form
  - Roche or its designee must review any proposed deviations from the sample Informed Consent Form.
- Current laboratory certification of the laboratory performing the analysis (if other than a Roche-approved central laboratory), as well as current references ranges for all laboratory tests
- A Clinical Research Agreement signed and dated by the study site
- Investigator Brochure Receipt signed and dated by the Principal Investigator
- Certified translations of an approved Informed Consent Form, and any other written information to be given to the participant (when applicable), IRB/EC approval letters, and pertinent correspondence
- A Protocol Acceptance Form signed and dated by the Principal Investigator

6.2 STUDY COMPLETION

The following data and materials are required by Roche before a study can be considered complete or terminated:

- Laboratory findings, clinical data, and all special test results from screening through the end of the study follow-up period
- All laboratory certifications for laboratories performing the analysis (is other than Roche-approved central laboratory), as well as current normal laboratory ranges for all laboratory tests

- eCRFs (including queries) properly completed by appropriate study personnel and electronically signed and dated by the investigator
- Completed Drug Accountability Records (Retrieval Record, Drug Inventory Log)
- Copies of protocol amendments and IRB/EC approval/notification, if appropriate
- A summary of the study prepared by the Principal Investigator (IRB summary close letter is acceptable)
- All essential documents (e.g., curriculum vitae for each Principal Investigator and subinvestigator, U.S. FDA Form 1572 for each site)
- A signed and dated Protocol Amendment Acceptance Form(s) (if applicable)
- Updated financial disclosure forms for the Principal Investigator and all subinvestigators listed on the U.S. FDA Form 1572 (applicable for 1 year after the last participant has completed the study)

6.3 INFORMED CONSENT FORM

Roche’s Sample Informed Consent Form will be provided to each site. Roche or its designee must review and approve any proposed deviations from the Sample Informed Consent Form or any alternate consent forms proposed by the site (collectively, the “Consent Forms”) before IRB/EC submission. Participants must be re-consented to the most current version of the Consent Forms during their participation in the study. The final IRB/EC-approved Consent Forms must be provided to Roche for regulatory purposes.

The Consent Forms must be signed by the participant before his or her participation in the study. The case history for each participant shall document the informed consent process and that written informed consent was obtained prior to participation in the study. The Informed Consent Form should be revised whenever there are changes to procedures outlined in the informed consent or when new information becomes available that may affect the willingness of the individual to participate. For any updated or revised Consent Forms, the case history for each participant shall document the informed consent process and that written informed consent was obtained for the updated/revised Consent Form from the participant or his/her representative for continued participation in the study.

If, during the course of the trial, a participant develops dementia and loses his/her capacity to consent, the most recently signed consent form prior to loss of capacity will remain active. Any revisions to the consent form will require re-consent by the participant’s representative (legally authorized representative when available, else temporary representative) as well as assent from the participant as per site standard operating procedure TR-Pr-058.

All signed and dated Consent Forms, and documentation of verbal consent, if applicable, must remain in each participant’s study file and must be available for verification by

study monitors at any time. A copy of each signed Consent Form must be provided to the participant or the participant's legally authorized representative. If applicable, it will be provided in a certified translation of the local language.

If the site utilizes a separate Authorization Form for participant authorization to use and disclose personal health information under the U.S. Health Insurance Portability and Accountability Act (HIPAA) regulations, the review, approval, and other processes outlined above apply except that IRB/EC review and approval may not be required per study site policies.

6.4 COMMUNICATION WITH THE INSTITUTIONAL REVIEW BOARD OR ETHICS COMMITTEE

This protocol, the Informed Consent Forms, any information to be given to the participant and relevant supporting information must be submitted to the IRB/EC by the Principal Investigator for review and approval before the study is initiated. In addition, any participant recruitment materials must be approved by the IRB/EC.

The Principal Investigator is responsible for providing written summaries of the status of the study to the IRB/EC annually or more frequently in accordance with the regulatory requirements and policies and procedures established by the IRB/EC. Investigators are also responsible for promptly informing the IRB/EC of any protocol changes or amendments and of any unanticipated problems involving risk to human participants or others.

In addition to the requirements to report protocol-defined AEs to the Sponsor, investigators are required to promptly report to their respective IRB/EC all unanticipated problems involving risk to human participants. Some IRBs/ECs may want prompt notification of all SAEs, whereas others require notification only about events that are serious, assessed to be related to study treatment, and are unexpected. Investigators may receive written IND safety reports or other safety-related communications from Roche. Investigators are responsible for ensuring that such reports are reviewed and processed in accordance with regulatory requirements and with the policies and procedures established by their IRB/EC and archived in the site's Study File.

6.5 STUDY MONITORING REQUIREMENTS

Site visits will be conducted by an authorized Roche representative to inspect study data, participants' medical records, and eCRFs. The Principal Investigator will permit Roche monitors/representatives and collaborators, the U.S. FDA, other regulatory agencies, Institutional Review Boards, and the respective national or local health authorities to inspect facilities and records relevant to this study.

6.6 ELECTRONIC CASE REPORT FORMS

Electronic case report forms (eCRFs) are to be completed using the Medidata RAVE® EDC system. Sites will receive training and a manual for appropriate eCRF completion.

eCRFs will be submitted electronically to Roche and should be handled in accordance with instructions from Roche.

All eCRFs should be completed by designated, trained examining personnel or the study coordinator as appropriate. The eCRF should be reviewed and electronically signed and dated by the investigator.

In addition, at the end of the study, the investigator will receive participant data for his or her site in a readable format on a compact disc that must be kept with the study records.

6.7 SOURCE DATA DOCUMENTATION

Study monitors will perform ongoing SDV to confirm that critical protocol data (i.e., source data) entered into the eCRFs by authorized site personnel are accurate, complete, and verifiable from source documents.

Source documents are where participant data are recorded and documented for the first time. They include, but are not limited to, hospital records, clinical and office charts, laboratory notes, memoranda, participant diaries or evaluation checklists, pharmacy dispensing records, recorded data from automated instruments, copies of transcriptions that are certified after verification as being accurate and complete, microfiche, photographic negatives, microfilm or magnetic media, X-rays, participant files, and records kept at the pharmacy, laboratories, and medico-technical departments involved in a clinical trial.

Source documents that are required to verify the validity and completeness of data entered into the eCRFs must never be obliterated or destroyed.

To facilitate SDV, the investigator(s) and institution(s) must provide the Sponsor direct access to applicable source documents and reports for trial-related monitoring, Sponsor audits, and IRB/EC review. The investigational site must also allow inspection by applicable regulatory authorities.

6.8 USE OF COMPUTERIZED SYSTEMS

When clinical observations are entered directly into an investigational site's computerized medical record system (i.e., in lieu of original hardcopy records), the electronic record can serve as the source document if the system has been validated in accordance with FDA requirements pertaining to computerized systems used in clinical research. An acceptable computerized data collection system (for clinical research purposes) would be one that (1) allows data entry only by authorized individuals; (2) prevents the deletion or alteration of previously entered data and provides an audit trail for such data changes (e.g., modification of file); (3) protects the database from tampering; and (4) ensures data preservation.

If a site's computerized medical record system is not adequately validated for the purposes of clinical research (as opposed to general clinical practice), applicable hardcopy source documents must be maintained to ensure that critical protocol data entered into the eCRFs can be verified.

6.9 STUDY MEDICATION ACCOUNTABILITY

All study drug required for completion of this study will be provided by Roche. The recipient will acknowledge receipt of the drug by returning the appropriate documentation form indicating shipment content and condition. Damaged supplies will be replaced.

Accurate records of all study drug received at, dispensed from, returned to and disposed of by the study site should be recorded by using the Drug Inventory Log.

Study drug will either be disposed of at the study site according to the study site's institutional standard operating procedure or returned to Roche with the appropriate documentation, as determined by the study site. If the study site chooses to destroy study drug, the method of destruction must be documented.

Roche must evaluate and approve the study site's drug destruction standard operating procedure prior to the initiation of drug destruction by the study site.

6.10 DISCLOSURE OF DATA

Participant medical information obtained by this study is confidential and may only be disclosed to third parties as permitted by the Informed Consent Form (or separate authorization to use and disclose personal health information) signed by the participant or unless permitted or required by law.

Data and samples arising from assessments prior to the treatment period may be shared prior to the end of the study subject to appropriate authorization to use such data and subject to appropriate provisions to maintain confidentiality, including that of mutation carrier status.

Medical information may be given to a participant's personal physician or other appropriate medical personnel responsible for the participant's welfare for treatment purposes.

Genetic status will be made available for those participants who desire this information after Study Period A is complete and results are available. Genetic counseling and disclosure of PSEN1 mutation results will be provided by an independent group specializing in genetic testing and counseling.

If a participant learns that he/she is not a PSEN1 mutation carrier during Study Period B, he/she will be withdrawn from Study Period B since placebo will not be administered to known non-carriers.

Data generated by this study must be available for inspection upon request by representatives of the U.S. FDA and other regulatory agencies, national and local health authorities, Roche monitors/representatives and collaborators, and the IRB/EC for each study site, if appropriate.

6.11 RETENTION OF RECORDS

U.S. FDA regulations (21 CFR §312.62[c]) and the ICH Guideline for GCP (see Section 4.9 of the guideline) require that records and documents pertaining to the conduct of this study and the distribution of investigational drug, including eCRFs, consent forms, laboratory test results, and medication inventory records, must be retained by the Principal Investigator for 2 years after the last marketing application approval in an ICH region or after at least 2 years have elapsed since formal discontinuation of clinical development of the investigational product. All state and local laws for retention of records also apply.

No records should be disposed of without the written approval of Roche. Written notification should be provided to Roche for transfer of any records to another party or moving them to another location.

For studies conducted outside the United States under a U.S. IND, the Principal Investigator must comply with the record retention requirements set forth in the U.S. FDA IND regulations and the relevant national and local health authorities, whichever is longer.

7. REFERENCES

Acosta-Baena N, Sepulveda-Falla D, Lopera-Gomez CM, et al. Pre-dementia clinical stages in presenilin 1 E280A familial early-onset Alzheimer's disease: a retrospective cohort study. Lancet Neurol 2011;10:213–20.

Adolfsson O, Pihlgren M, Toni N, et al. An effector-reduced anti–β-amyloid (Aβ) antibody with unique aβ binding properties promotes neuroprotection and glial engulfment of Aβ. J Neurosci 2012;32:9677–89.

Aguirre-Acevedo DC, Gómez RD, Moreno S, et al. Validity and reliability of the CERAD Col Neuropsychological Battery. Rev Neurol 2007;45:655–60.

Albert MS, DeKosky ST, Dickson D, et al. The diagnosis of mild cognitive impairment due to Alzheimer's disease: recommendations from the National Institute on Aging–Alzheimer's Association workgroups on diagnostic guidelines for Alzheimer's disease. Alzheimers Dement 2011;7:270–79.

Armitage SG. An analysis of certain psychological tests used for the evaluation of brain injury. Psychological Monographs 1946;60:i–48.

Ayutyanont N, Langbaum JB, Hendrix SB, et al. The Alzheimer's Prevention Initiative composite cognitive test score: sample size estimates for the evaluation of preclinical Alzheimer's disease treatments in presenilin 1 E280A mutation carriers. J Clin Psychiatry2014;75:652–60.

Barkhof F, Daams M, Scheltens P, et al. An MRI rating scale for amyloid-related imaging abnormalities with edema or effusion. Am J Neuroradiol 2013;34:1550–5.

Bateman RJ, Aisen PS, De Strooper B, et al. Autosomal-dominant Alzheimer's disease: a review and proposal for the prevention of Alzheimer's disease. Alzheimers Res Ther 2011;2:35.

Bateman RJ, Xiong C, Benzinger TL, et al. Clinical and biomarker changes in dominantly inherited Alzheimer's disease 2012;367:795-804.

Brookmeyer R, Gray S, Kawas C et al. Projections of Alzheimer's disease in the United States and the public health impact of delaying disease onset. Am J Public Health 1998;88:1337–42.

Buschke H. Cued recall in amnesia. J Clin Neuropsychol 1984;6:433–40.

Carlson C, Estergard W, Oh J, et al. Prevalence of asymptomatic vasogenic edema in pretreatment Alzheimer's disease study cohorts from phase 3 trials of semagacestat and solanezumab. Alzheimers Dement 2011;7:396–401.

Caselli RJ, Langlais BT, Dueck AC, et al. Neuropsychological decline up to 20 years before incident mild cognitive impairment. Alzheimers Dement 2020;16:512-523.

Chong CP, Street PR. Pneumonia in the elderly: a review of the epidemiology, pathogenesis, microbiology, and clinical features. Southern Med J 2008;101:1141–45.

Corrada MM, Brookmeyer R, Paganini-Hill A, et al. Dementia incidence continues to increase with age in the oldest old: the 90+ study. Ann Neurol 2010;67:114–21.

Cummings JL. The Neuropsychiatric Inventory: assessing psychopathology in dementia patients. Neurology 1997;48:S10–S16.

Cummings JL, Mega M, Gray K, et al. The Neuropsychiatric Inventory: comprehensive assessment of psychopathology in dementia. Neurology 1994;44:2308–14.

Deng R, Iyer S, Theil FP, et al. Projecting human pharmacokinetics of therapeutic antibodies from nonclinical data. What have we learned? MAbs 2011;31:61–66.

Dirks NL, Meibohm B. Population pharmacokinetics of therapeutic monoclonal antibodies. Clin Pharmacokinet 2010;49:633–59.

Donohue MC, Sperling RA, Salmon DP, et al. The preclinical Alzheimer cognitive composite: measuring amyloid-related decline. JAMA neurology. 2014 71:961-70.

Evans DA, Funkenstein HH, Albert MS, et al. Prevalence of Alzheimer's disease in a community population of older persons. Higher than previously reported. JAMA 1989;262:2551–6.

Fleisher AS, Chen K, Quiroz YT, et al. Florbetapir PET analysis of amyloid-β deposition in the presenilin 1 E280A autosomal dominant Alzheimer's disease kindred: a cross-sectional study. Lancet Neurol 2012;11:1057–65.

Fleisher AS, Chen K, Quiroz YT, et al. Associations between biomarkers and age in the presenilin 1 E280A autosomal dominant Alzheimer disease kindred: a cross-sectional study. JAMA Neurol 2015;72:316–24.

Fuller JP, Stavenhagen JB, Teeling JL. New roles for Fc receptors in neurodegeneration—the impact on immunotherapy for Alzheimer's disease. Front Neurosci 2014;8:1–10.

Folstein MF, Folstein SE, McHugh PR. "Mini-mental state." A practical method for grading the cognitive state of patients for the clinician. J Psychiatr Res 1975;12:189–98.

Gollan TH, Weissberger G, Runnqvist E, et al. Self-ratings of spoken language dominance: A multi-lingual naming test (MINT) and preliminary norms for young and aging Spanish-English bilinguals. Bilingualism: Language and Cognition 2012;15:594–615.

Goos JD, Henneman WJ, Sluimer JD, et al. Incidence of cerebral microbleeds: a longitudinal study in a memory clinic population. Neurology 2010;74:1954–60.

Greenberg SM, Vernooij MW, Cordonnier C, et al. Cerebral microbleeds: a guide to detection and interpretation. Lancet Neurol 2009;8:165–74.

Grober E and Buschke H. Genuine memory deficits in dementia. Dev Neuropsychol 1987;3:13–36.

Hardy J, Selkoe DJ. The amyloid hypothesis of Alzheimer's disease: progress and problems on the road to therapeutics. Science 2002;297:353–6.

Heneka MT, Carson MJ, El Khoury J, et al. Neuroinflammation in Alzheimer’s disease. Lancet Neurol 2015;14:388–405.

Henley DB, Sundell KL, Sethuraman G, et al. Adverse events and dropouts in Alzheimer’s disease studies: what can we learn. Alzheimer Dement 2014;11:24–31.

Kaufer DI, Cummings JL, Ketchel P, et al. Validation of the NPI-Q, a brief clinical form of the Neuropsychiatric Inventory. J Neuropsychiatry Clin Neurosci 2000;12:233–9.

Koffie RM, Meyer-Luehmann M, Hashimoto T, et al. Oligomeric amyloid β associates with postsynaptic densities and correlates with excitatory synapse loss near senile plaques. PNAS 2009;106:4012–17.

Kramp VP and Herrling P. List of drugs in development for neurodegenerative diseases: update June 2010. Neurodegener Dis 2011;8:44–94.

Langbaum JB, Hendrix SB, Ayutyanont N, et al. An empirically derived composite cognitive test score with improved power to track and evaluate treatments for preclinical Alzheimer's disease. Alzheimers Dement advance online publication 21 April 2014. doi: 10.1016/j.jalz.2014.02.002.

Liu P, Reed MN, Kotilinek LA, et al. Quaternary structure defines a large class of amyloid-β oligomers neutralized by sequestration. Cell Rep 2015;11:1760–71.

McKhann GM, Knopman DS, Chertkow H, et al. The diagnosis of dementia due to Alzheimer's disease: Recommendations from the National Institute on Aging and the Alzheimer's Association workgroup. Alzheimers Dement 2011;7:263–9.

Mohs RC, Knopman D, Petersen RC, et al. Development of cognitive instruments for use in clinical trials of antidementia drugs: additions to the Alzheimer’s Disease Assessment Scale that broaden its scope. Alzheimer Dis Assoc Disord 1997;11(S2):S13–S21.

Morris JC. The Clinical Dementia Rating (CDR): current version and scoring rules. Neurology 1993;43:2412–14.

Morris JC, Heyman A, Mohs RC, et al. The Consortium to Establish a Registry for Alzheimer's Disease (CERAD). Part I. Clinical and neuropsychological assessment of Alzheimer's disease. Neurology 1989;39:1159–65.

Mould DR, Sweeney KR. The pharmacokinetics and pharmacodynamics of monoclonal antibodies—mechanistic modeling applied to drug development. Curr Opin Drug Discov Develop 2007;10:84–96.

Oddo S, Billings L, Kesslak JP, et al. Abeta immunotherapy leads to clearance of early, but not late, hyperphosphorylated tau aggregates via the proteasome. Neuron 2004;43:321–32.

Ostrowitzki S, Dentula D, Thurfiell L, et al. Mechanism of amyloid removal in patients with Alzheimer disease treated with gantenerumab. Arch Neurol 2012:69:198–207.

Randolph C. Repeatable Battery for the Assessment of Neuropsychological Status. San Antonio, TX: The Psychological Coporation, 1998.

Raven JC. Revised Manual for Raven's Progressive Matrices and Vocabulary Scale. New York: The Psychological Corporation, Harcourt Brace Jovanovich, Inc., 1976.

Reiber H, Felgenhauer K. Protein transfer at the blood cerebrospinal fluid barrier and the quantitation of the humoral immune response within the central nervous system. Clin Chim Acta 1987;163:319–28.

Reiman EM, Langbaum JBS. Brain imaging in the evaluation of putative Alzheimer's disease slowing, risk-reducing and prevention therapies. In: Jagust WJ, D'Esposito M (editors). Imaging the aging brain. New York: Oxford University Press, 2009;319–50.

Reiman EM, Langbaum JBS, Tariot PN. Alzheimer's Prevention Initiative: a proposal to evaluate presymptomatic treatments as quickly as possible. Biomark Med 2010;4:3–14.

Reisberg B. Functional assessment staging (FAST). Psychopharmacol Bull 1988;24:653–9.

Reisberg B, Ferris SH, de Leon MJ, et al. The Global Deterioration Scale for assessment of primary degenerative dementia. Am J Psychiatry 1982;139:1136–9.

Reisberg B, Schneck M, Ferris S, et al. The Brief Cognitive Rating Scale (BCRS): findings in primary degenerative dementia. Psychopharmacol Bull 1983;47:47–50.

Rosen WG, Mohs RC, Davis KL. A new rating scale for Alzheimer's disease. Am J Psychiatry 1984;141:1356–64.

Salloway S, Sperling R, Fox NC, et al. Two Phase 3 trials of bapineuzumab in mild-to-moderate Alzheimer's disease. N Engl J Med 2014;370:322–33.

Salloway S, Sperling R, Gilman S, et al. A phase 2 multiple ascending dose trial of bapineuzumab in mild to moderate Alzheimer disease. Neurology 2009; 73:2061–70.

Sclan SG, Reisberg B. Functional assessment staging (FAST) in Alzheimer's disease: reliability, validity, and ordinality. Int Psychogeriatr 1992;4:55–69.

Selkoe DJ. Soluble oligomers of the amyloid beta-protein impair synaptic plasticity and behavior. Behav Brain Res 2008;192:106–13.

Sevigny J, Chiao P, Williams L, et al. Randomized, doubleblind, placebo-controlled, Phase Ib study of Aducanumab (BIIB037), an anti-A! monoclonal antibody, in patients with prodromal or mild Alzheimer's disease: Interim results by disease stage and ApoE 14 status [poster]. [Resource available on the Internet]. April 2015 [cited: June 2016]. Available from:

http://www.biogenconferences.com/AAN2015/BART/BART_Sevigny_PhIB_subgp_IA2.pdf.

Sheikh JI and Yesavage JA. Geriatric Depression Scale (GDS): Recent evidence and development of a shorter version. Clinical Gerontologist: J Aging Mental Health 1986;5:165–73.

Sperling RA, Aisen PS, Beckett LA, et al. Toward defining the preclinical stages of Alzheimer's disease: Recommendations from the National Institute on Aging and the Alzheimer's Association workgroup. Alzheimers Dement 2011b;7:280–92.

Sperling RA, Jack CR Jr, Black SE, et al. Amyloid-related imaging abnormalities in amyloid-modifying therapeutic trials: recommendations from the Alzheimer's Association Research Roundtable Workgroup. Alzheimers Dement 2011a;7:367–85.

Sperling R, Salloway S, Brooks DJ, et al. Amyloid-related imaging abnormalities in patients with Alzheimer's disease treated with bapineuzumab: a retrospective analysis. Lancet Neurol 2012;11:241–9.

Tounsi H, Deweer B, Ergis AM, et al. Sensitivity to semantic cuing: an index of episodic memory dysfunction in early Alzheimer disease. Alzheimer Dis Assoc Discord 1999;13:38-46.

Ultsch M, Li B, Maurer T, et al. Structure of crenezumab complex with Aβ shows loss of β-hairpin. Sci Rep 2016;1–11 (resource available on the Internet). Available at: https://www.ncbi.nlm.nih.gov/pmc/articles/PMC5171940/pdf/srep39374.pdf

Vernooij MW, van der Lugt A, Ikram MA, et al. Prevalence and risk factors of cerebral microbleeds: the Rotterdam Scan Study. Neurology 2008;70(14):1208–14.

Wilcock DM, et al. Deglycosylated anti-amyloid-beta antibodies eliminate cognitive deficits and reduce parenchymal amyloid with minimal vascular consequences in aged amyloid precursor protein transgenic mice. J Neurosci 2006;26:5340–46.

Xing B, Bachstetter AD, Van Eldik LJ. Microglial p38α MAPK is critical for LPS-induced neuron degeneration, through a mechanism involving TNFa. Mol Neurodegener 2011;6:84.

Yakushiji Y, Nishiyama M, Yakushiji S, et al. Brain microbleeds and global cognitive function in adults without neurological disorder. Stroke 2008;39:3323–8.

Appendix A-1
(SC Dosing) Study Flowchart (Screening–Week 104 [ij])

Timepoint	Screen	Base-line	W2	W4	W6-W10 (Q2W)	W12	W14	W16	W18–W22 (Q2W)	W24	W26–W34 (Q2W)	W36	W38–W50 (Q2W)	W52	W54–W74 (Q2W)	W76	W78–W102 (Q2W)	W104
Visit Window	Days –56 to –1	Day 1	± 5d	± 5d	± 5d	± 5d	± 5d	± 5d	± 5d	± 5d	± 5d	± 5d	± 5d	± 5d	± 5d	± 5d	± 5d	± 5d
Signed informed consent [a]	x																	
Review of eligibility criteria	x	x [b]																
IxRS	x [c]	x [c]																
Medical history	x																	
Physical examination	x	x				x				x		x		x		x		x
Height and weight [d]	x									x				x		x		x
Neurological examination	x	x				x				x		x		x		x		x
Evaluate criteria for MCI/AD	x									x				x		x		x
GDS [e, f]	x									x				x		x		x
SMC [e, g, f]	x									x				x		x		x
Screening Battery [e, h]	x																	
Composite Cognitive Battery [e, i]		x				x				x				x		x		x
Extended Cognitive Battery [e, j]		x								x				x		x		x
SPCQ [f, k, f]	x	x								x				x		x		x
CDR [e, g, l, f]	x									x				x		x		x
FAST [g, f]	x									x				x		x		x
NPI [g, f]		x								x				x		x		x

Appendix A-1
(SC Dosing) Study Flowchart (Screening–Week 104[jj]) (cont.)

Timepoint	Screen	Base-line	W2	W4	W6-W10 (Q2W)	W12	W14	W16	W18–W22 (Q2W)	W24	W26–W34 (Q2W)	W36	W38–W50 (Q2W)	W52	W54–W74 (Q2W)	W76	W78–W102 (Q2W)	W104
Visit Window	Days –56 to –1	Day 1	± 5d	± 5d	± 5d	± 5d	± 5d	± 5d	± 5d	± 5d	± 5d	± 5d	± 5d	± 5d	± 5d	± 5d	± 5d	± 5d
Suicidality assessment [e, f]		x				x				x		x		x	W64	x	W92	x
Urinalysis [m]	x	x		x		x				x		x		x		x		x
Chemistry [n]	x	x		x		x				x		x		x		x		x
Hematology [o]	x	x		x		x				x		x		x		x		x
Coagulation [p]	x	x		x		x				x		x		x		x		x
DNA (APOE, PSEN1) [q]	x																	
12-lead ECG [r]	x	x		x		x		x [s]		x				x				x
PK sample (serum) [t, u]		x		x		x		x [s]		x		x		x		x		x
PD sample (plasma) [t]	x	x		x		x		x [s]		x		x		x		x		x
Exploratory serum, plasma, RNA samples [t]		x		x		x		x [s]		x		x		x		x		x
ADA sample [u]		x								x				x		x		x
Urine screen for drugs of abuse [v]	x													x				x
Serum pregnancy test [w]	x																	
Urine pregnancy test [w]		x	x	x	x	x	x	x	x	x	x	x	x	x	x	x	x	x
Vital signs [x]	x	x	x	x	x	x	x	x	x	x	x	x	x	x	x	x	x	x
Dispense study medication [y, z, aa]		x	x	x	x	x	x	x	x	x	x	x	x	x	x	x	x	x
Concomitant medication review [f]	x	x	x	x	x	x	x	x	x	x	x	x	x	x	x	x	x	x
Adverse event review [f]		x	x	x	x	x	x	x	x	x	x	x	x	x	x	x	x	x
Brain MRI [bb]	x [cc]					x				x		x		x		x		x

Appendix A-1
(SC Dosing) Study Flowchart (Screening–Week 104 [jj]) (cont.)

Timepoint	Screen	Base-line	W2	W4	W6-W10 (Q2W)	W12	W14	W16	W18–W22 (Q2W)	W24	W26–W34 (Q2W)	W36	W38–W50 (Q2W)	W52	W54–W74 (Q2W)	W76	W78–W102 (Q2W)	W104
Visit Window	Days –56 to –1	Day 1	± 5d	± 5d	± 5d	± 5d	± 5d	± 5d	± 5d	± 5d	± 5d	± 5d	± 5d	± 5d	± 5d	± 5d	± 5d	± 5d
Fibrillar amyloid PET [w, dd]	x [ee]																	x
FDG-PET [w, bb, dd, ff]	x [ee]					x												x
Lumbar puncture for CSF sample (optional) [gg]		x [hh]																x
DNA Repository sample		x [ii]																

AD=Alzheimer's disease; ADA=anti-drug antibody; APOE=apolipoprotein E; CDR=Clinical Dementia Rating; CSF=cerebrospinal fluid; d=day; ET=early termination; ECG=electrocardiogram; FAST=Functional Assessment Staging of Alzheimer's Disease; FCSRT=Free and Cued Selective Reminding Test; FDG=fluorodeoxyglucose; GDS=Geriatric Depression Scale; IxRS=interactive voice or web-based response system; LP=lumbar puncture; MCI=mild cognitive impairment; MMSE=Mini-Mental State Examination; MRI=magnetic resonance imaging; NPI=Neuropsychiatric Inventory; PD=pharmacodynamic; PET=positron emission tomography; PK=pharmacokinetic; *PSEN1*=presenilin-1 (gene); Q2W=every 2 weeks; RBANS=Repeatable Battery for the Assessment of Neuropsychological Status; Screen=screening; SMC=Subjective Memory Checklist; SPCQ=Study Partner Characterization Questionnaire; TMT=Trail-Making Test; W=week.

Note: On treatment days, all assessments should be performed prior to dosing unless otherwise noted.

a Obtain informed consent prior to conducting any assessments. Informed Consent Forms must be signed by the participant and study partner.

b Review study criteria to ensure that the participant still qualifies to participate in the study. All baseline assessments must be completed before first study drug administration (Day 1).

c At screening, obtain participant screening number assignment through IxRS. On Day 1, obtain participant randomization number and kit assignment number through IxRS.

d Height should be obtained only at screening. Weight should be obtained at screening and all other indicated visits noted in the study flowchart.

e Cognitive and selected other assessments (GDS, SMC, CDR, and suicidality assessment) should not be administered to the participant immediately after dosing or any potentially stressful procedures (e.g., blood draws, LPs, imaging). Also, participants should not perform these assessments while fasting. For each participant, best efforts should be exerted to perform subsequent assessments at the same time of day as the baseline assessments.

f In rare instances, these assessments permitted by phone or video technology (see Section 4.5.4).

[g] The SPCQ, CDR (study partner portion), FAST, SMC, and NPI should be administered to the same study partner throughout the study. The study partner must be present at the clinic for these assessments at screening and baseline, and best efforts should be exerted in order for the study partner to be present for all other visits requiring study partner assessments. In rare instances, for which the study partner cannot be present in person, these assessments may be administered by telephone. See Appendix B for additional details regarding study partner considerations.

[h] The Screening Battery should be administered in the following order: MMSE, Word List: Memory, GDS, Word List: Recall, Word List: Recognition. Note: the GDS is not part of the Screening Battery but is inserted as a distracter and to create a delay for Word List: Recall.

[i] The API ADAD Composite Cognitive Test Battery should be administered in the following order: MMSE, Multilingual Naming Test, Word List: Memory, CERAD Constructional Praxis, Word List: Recall, Word List: Recognition, Raven's Progressive Matrices–Set A.

[j] The Extended Cognitive Battery should be administered after the API ADAD Composite Cognitive Test Battery.

[k] Completed prior to the administration of all other study partner assessments at each visit.

[l] Study partner portion of the CDR must be administered prior to the participant portion of the CDR.

[m] Includes pH, protein, glucose, ketones, bilirubin, blood, specific gravity, leukocyte esterase, urobilinogen, and nitrite, as well as microscopic analysis on the basis of dipstick results.

[n] Includes sodium, potassium, BUN, bicarbonate, estimated glomerular filtration rate, calcium, AST, ALT, γ-glutamyl transpeptidase, total and direct bilirubin, alkaline phosphatase, lactate dehydrogenase, total protein, albumin, glucose, cholesterol, triglycerides, uric acid, creatine phosphokinase, and glycosylated hemoglobin. At screening, this sample will also include vitamin B12, ultrasensitive thyroid-stimulating hormone, and thyroxine. Participants will be fasting (water only) for blood tests only at screening; fasting is not required at all visits.

[o] Includes hematocrit, hemoglobin, RBC count, WBC count with differential and platelets. Participants will be fasting (water only) for blood tests only at screening; fasting is not required at all visits.

[p] Includes aPTT and INR. Participants will be fasting (water only) for blood tests only at screening; fasting is not required at all timepoints.

[q] Sample should be taken immediately after consent and assignment of a screening number to ensure adequate time for testing and reporting of results.

[r] Routine ECGs will be calculated and read locally; the exception is the optional ECG substudy at Weeks 16–17, for which the additional ECG data will only be processed centrally. Baseline ECGs for the optional ECG substudy participants will be read locally and also processed centrally. Single ECGs at baseline, Week 4, and Week 12 should be submitted to the vendor for all participants.

[s] At Week 16, and 7 days ($\pm$3 days) following the Week 16 study drug administration, a subset of participants will undergo an additional ECG assessment. Each ECG assessment in this ECG substudy will be accompanied by additional PK, PD, and exploratory plasma, serum, and RNA blood samples as well as vital signs (BP, HR, RR) after the ECG assessment (order of assessments: ECG, vital signs, blood draws, and study drug administration). Triplicate ECGs are required only for ECG substudy participants, and only at baseline, Week 16, and 7 days ($\pm$3 days) following the Week 16 study drug administration.

[t] Collect PK, PD, and exploratory serum, plasma, and RNA samples prior to study drug administration.

[u] Participants who experience serious or severe hypersensitivity reactions (e.g., hypotension, mucosal involvement) should not receive additional study drug. In addition, in any case of anaphylaxis, anaphylactoid reaction, serious or severe hypersensitivity reaction, ADA and concurrent PK samples should be collected as close as possible to the event, and then at the 4-week ET visit and at the 16-week safety follow-up visit after the final dose (see schedules of assessments for further details).

[v] Includes amphetamines, barbiturates, benzodiazepines, cannabinoids, cocaine, and opioids.

[w] Serum pregnancy test must be administered and have a negative result prior to enrollment into the study. Urine pregnancy test must be administered and have a negative result prior to any study drug administration. If the participant has discontinued study drug but is continuing with study assessments, the urine pregnancy test may be deferred except for visits during which FDG-PET or florbetapir PET scans are administered. Serum and urine pregnancy testing will apply to all women unless documented (by medical records or physician's note) to be surgically sterile or postmenopausal as defined in Section 3.4.5.

[x] Vital signs will include measurements of temperature, heart rate, respiratory rate, and systolic and diastolic blood pressure while the participant has been supine for ≥3 minutes. On study drug administration days, record vital signs prior to the injection.

[y] Although visit windowing for dosing days allows for ±5 days around any expected visit day, the interval between study drug administrations from one biweekly visit to the following biweekly visit must be between 8 and 20 days.

[z] Participants will be observed for a minimum of 30 minutes for the first four dose administrations and 15 minutes for subsequent dose administrations, following injection of study drug.

[aa] During the treatment period: Participants who develop >8 amyloid-related imaging abnormalities–hemosiderin deposition cumulatively will receive a lower dose of the study drug. Participants who develop >10 amyloid-related imaging abnormalities–hemosiderin deposition cumulatively will be permanently discontinued from the study drug. Cumulative is the sum of amyloid-related imaging abnormalities–hemosiderin deposition at baseline and newly detected amyloid-related imaging abnormalities–hemosiderin deposition during the study.

[bb] If MRI and FDG-PET scans are performed on the same day, the FDG-PET scan should be performed first. A urine pregnancy test must be administered and have a negative result prior to any PET scan. It is recommended, but not required, to use effective contraception during sexual intercourse occurring within 24 hours following a male or female participant receiving a PET scan.

[cc] Screening MRI is to occur >10 days prior to randomization to ensure adequate time for MRI interpretation prior to randomization, and also before the optional lumbar puncture, with sufficient time to allow review for possible contraindications to LP.

[dd] ET FDG-PET and fibrillar amyloid PET scans should only be performed if the investigator has determined that the total radiation exposure from the ET FDG-PET and fibrillar amyloid PET scans does not exceed local safety guidelines.

[ee] FDG-PET and fibrillar amyloid PET should be performed after the participant is deemed eligible on the basis of all other screening assessments. Although part of the screening period, eligibility is not dependent upon FDG-PET and fibrillar amyloid PET results.

[ff] Participant must be fasting (water only) for a minimum of 4 hours prior to the scan. FDG-PET should be performed a minimum of 12 hours and a maximum of 7 days apart from the accompanying fibrillar amyloid PET scan. For each participant, best efforts should be exerted to perform subsequent FDG-PET scans at the same time of day as the baseline scan.

[gg] It is strongly preferred that the LP be performed in the morning. Participants will be required to stay in the clinic or hospital for approximately 1 hour after the LP for safety follow-up. For each participant, best efforts should be exerted to perform subsequent LPs at the same time of day as the baseline LP. See protocol (Section 4.5.1 [l]) for additional details regarding LP.

[hh] If the participant agrees to undergo optional LP, CSF sample should be drawn a) after the participant is deemed eligible on the basis of all other screening assessments, b) after the participant is deemed eligible on the basis of MRI, and c) at least 72 hours prior to the first study drug administration.

[ii] This sample may be collected during a subsequent visit if it cannot be collected at baseline.

[jj] For participants who discontinue study drug or the study prior to study completion, see ET-A and ET-B in Appendix A-2 along with corresponding footnotes.

Appendix A-2
(SC Dosing) Study Flowchart (Week 106–End of Study(Period A)

Timepoint	From W106 (all weeks except those specified to the right)	W116, 144, 168, 196, 220, 248, 272, 300, 324, 352, 376, 404, 428	W 128 [a]	W 156 [a]	W 180 [a]	W 208 [a]	W 232 [a]	W 260 [a]	W 284 [a]	W 312 [a]	W 336 [a]	W 364 [a]	W 388 [a]	W 416 [a]	ET-A Study Com-pletion Visit [a, b]	ET-B Study Com-pletion Visit [a, c]	Safety Follow-up 16W last dose [d]
	(Q2W)	(Q2W)	Q2W	Q2W	Q2W	Q2W	Q2W	Q2W	Q2W	Q2W	Q2W	Q2W	Q2W	Q2W			
Visit Window	± 5d	± 5d	± 5d	± 5d	± 5d	± 5d	± 5d	± 5d	± 5d	± 5d	± 5d	± 5d	± 5d	± 5d	± 7d	± 7d	± 7d
Physical examination			x	x	x	x	x	x	x	x	x	x	x	x	x	x	
Weight [e]			x	x	x	x	x	x	x	x	x	x	x	x	x	x	
Neurological examination			x	x	x	x	x	x	x	x	x	x	x	x	x	x	
Evaluate criteria for MCI/AD			x	x	x	x	x	x	x	x	x	x	x	x	x		
GDS [f, g]			x	x	x	x	x	x	x	x	x	x	x	x	x		
Subjective Memory Checklist [f, h]			x	x	x	x	x	x	x	x	x	x	x	x	x		
Composite Cognitive Battery [f, j]			x	x	x	x	x	x	x	x	x	x	x	x	x	x	
Extended Cognitive Battery [f, k]			x	x	x	x	x	x	x	x	x	x	x	x	x		
SPCQ [h, i, g]			x	x	x	x	x	x	x	x	x	x	x	x	x		
CDR [f, h, l, g]			x	x	x	x	x	x	x	x	x	x	x	x	x		
FAST [h, g]			x	x	x	x	x	x	x	x	x	x	x	x	x		
NPI [h, g]			x	x	x	x	x	x	x	x	x	x	x	x	x		
Suicidality assessment [f,g]		x	x	x	x	x	x	x	x	x	x	x	x	x	x	x	
Urinalysis [m]			x	x	x	x	x	x	x	x	x	x	x	x	x	x	
Chemistry [n]			x	x	x	x	x	x	x	x	x	x	x	x	x	x	

Appendix A-2
(SC Dosing) (Week 106–End of Study Period A) (cont.)

Timepoint	From W106 (all weeks except those specified to the right)	W116, 144, 168, 196, 220, 248, 272, 300, 324, 352, 376, 404, 428	W 128 [a]	W 156 [a]	W 180 [a]	W 208 [a]	W 232 [a]	W 260 [a]	W 284 [a]	W 312 [a]	W 336 [a]	W 364 [a]	W 388 [a]	W 416 [a]	ET-A Study Com-pletion Visit [a, b]	ET-B Study Com-pletion Visit [a, c]	Safety Follow-up 16W last dose [d]
	(Q2W)	(Q2W)	Q2W	Q2W	Q2W	Q2W	Q2W	Q2W	Q2W	Q2W	Q2W	Q2W	Q2W	Q2W			
Visit Window	± 5d	± 5d	± 5d	± 5d	± 5d	± 5d	± 5d	± 5d	± 5d	± 5d	± 5d	± 5d	± 5d	± 5d	± 7d	± 7d	± 7d
Hematology [o]			x	x	x	x	x	x	x	x	x	x	x	x	x	x	
Coagulation [p]			x	x	x	x	x	x	x	x	x	x	x	x	x	x	
12-lead ECG [q]				x		x		x		x		x		x	x	x	
PK sample (serum) [r, s]			x	x	x	x	x	x	x	x	x	x	x	x	x	x	x
PD sample (plasma) [r]			x	x	x	x	x	x	x	x	x	x	x	x	x	x	x
Exploratory serum, plasma, RNA samples [r]			x	x	x	x	x	x	x	x	x	x	x	x	x	x	
ADA sample [r]			x	x	x	x	x	x	x	x	x	x	x	x	x	x	x
Urine screen for drugs of abuse [t]				x		x		x		x		x		x	x	x	
Urine pregnancy test [u]	x	x	x	x	x	x	x	x	x	x	x	x	x	x	x	x	x
Vital signs [v]	x	x	x	x	x	x	x	x	x	x	x	x	x	x	x	x	
Dispense study medication [u, w, x, y]	x	x	x	x	x	x	x	x	x	x	x	x	x	x			
Concomitant medication review [g]	x	x	x	x	x	x	x	x	x	x	x	x	x	x	x	x	x
Adverse event review [g]	x	x	x	x	x	x	x	x	x	x	x	x	x	x	x	x	x

Appendix A-2
(SC Dosing) (Week 106–End of Study Period A) (cont.)

Timepoint	From W106 (all weeks except those specified to the right)	W116, 144, 168, 196, 220, 248, 272, 300, 324, 352, 376, 404, 428	W 128 [a]	W 156 [a]	W 180 [a]	W 208 [a]	W 232 [a]	W 260 [a]	W 284 [a]	W 312 [a]	W 336 [a]	W 364 [a]	W 388 [a]	W 416 [a]	ET-A Study Com-pletion Visit [a, b]	ET-B Study Com-pletion Visit [a, c]	Safety Follow-up 16W last dose [d]
	(Q2W)	(Q2W)	Q2W	Q2W	Q2W	Q2W	Q2W	Q2W	Q2W	Q2W	Q2W	Q2W	Q2W	Q2W			
Visit Window	± 5d	± 5d	± 5d	± 5d	± 5d	± 5d	± 5d	± 5d	± 5d	± 5d	± 5d	± 5d	± 5d	± 5d	± 7d	± 7d	± 7d
Brain MRI [z]			x [aa]	x [aa]	x [aa]	x [aa]	x [aa]	x [aa]	x [aa]	x [aa]	x [aa]	x [aa]	x [aa]	x [aa]	x [aa]	x [aa]	
F brillar amyloid PET [x,ff]								x							x [dd]	x [dd]	
FDG-PET [z, aa, bb, ff]								x							x [dd]	x [dd]	
Lumbar puncture for CSF sample (optional) [ee]								x							x [dd]	x [dd]	

AD = Alzheimer's disease; ADA = anti-drug antibody; CDR = Clinical Dementia Rating; CSF = cerebrospinal fluid; d = day; ECG = electrocardiogram; ET = early termination; FAST = Functional Assessment Staging of Alzheimer's Disease; FDG = fluorodeoxyglucose; GDS = Geriatric Depression Scale; IxRS = interactive voice or web-based response system; MCI = mild cognitive impairment; MRI = magnetic resonance imaging; NPI = Neuropsychiatric Inventory; PD = pharmacodynamic; PET = positron emission tomography; PK = pharmacokinetic; Q2W = every 2 weeks; RBANS = Repeatable Battery for the Assessment of Neuropsychological Status; Screen = screening; SMC = Subjective Memory Checklist; SPCQ = Study Partner Characterization Questionnaire; TMT = Trail-Making Test; W = week.

Note: On treatment days, all assessments should be performed prior to dosing unless otherwise noted.

a Participants who discontinue from study drug treatment (early discontinuation) will not receive further doses of study drug, but should be asked to stay in the study for subsequent safety, cognitive, and biomarker assessments at complex visits, including at Weeks 24, 36, 52, 76, 104, 128, 156, 180, 208, 232, 260, and every 24-28 weeks thereafter until the study ends. In addition, the suicidality assessment, review of concomitant medications and adverse events should be collected at Weeks 12, 64, 92, 116, 144, 168, 196, 220, 248, and every 24-28 weeks until the study ends; these assessments may be completed by telephone (but it is preferred they be completed in person). If a phone visit it completed during these weeks, vital signs assessments may be omitted.

b If a participant discontinues from the study (ET) or discontinues from study drug treatment and elects to no longer stay in the study, ET Visit A should be completed if the last visit occurred more than 12 weeks after functional, behavioral and cognitive assessments were performed;

c If a participant discontinues from the study (ET) or discontinues from study drug treatment and elects to no longer stay in the study, ET Visit B should be performed if the last visit occurred within 12 weeks of functional, behavioral, and cognitive assessments.

d Safety follow-up at 16 weeks is only applicable for those participants that do not consent to the continued access study.

e Weight should be obtained at all weeks marked on the study flowchart.

Appendix A-2
(SC Dosing) (Week 106–End of Study Period A) (cont.)

[f] Cognitive and selected other assessments (GDS, SMC, CDR, and suicidality assessment) should not be administered to the participant immediately after dosing or any potentially stressful procedures (e.g., blood draws, LP, imaging). Also, participants should not perform these assessments while fasting. For each participant, best efforts should be exerted to perform subsequent assessments at the same time of day as the baseline assessments.

[g] In rare instances, these assessments permitted by phone or video technology (See Section 4.5.4).

[h] The SPCQ, CDR (study partner portion), FAST, SMC, and NPI should be administered to the same study partner throughout the study. Best efforts should be exerted in order for the study partner to be present for all other visits requiring study partner assessments. In rare instances, for which the study partner cannot be present in person, these assessments may be administered by telephone. See Appendix B for additional details regarding study partner considerations.

[i] Completed prior to the administration of all other study partner assessments at each visit.

[j] The API ADAD Composite Cognitive Test Battery should be administered in the following order: MMSE, Multilingual Naming Test, Word List: Memory, CERAD Constructional Praxis, Word List: Recall, Word List: Recognition, Raven's Progressive Matrices–Set A.

[k] The Extended Cognitive Battery should be administered after the API ADAD Composite Cognitive Test Battery.

[l] Study partner portion of the CDR must be administered prior to the participant portion of the CDR.

[m] Includes pH, protein, glucose, ketones, bilirubin, blood, specific gravity, leukocyte esterase, urobilinogen, and nitrite, as well as microscopic analysis on the basis of dipstick results.

[n] Includes sodium, potassium, BUN, bicarbonate, estimated glomerular filtration rate, calcium, AST, ALT, γ-glutamyl transpeptidase, total and direct bilirubin, alkaline phosphatase, lactate dehydrogenase, total protein, albumin, glucose, cholesterol, triglycerides, uric acid, creatine phosphokinase, and glycosylated hemoglobin. At screening, this sample will also include vitamin B12, ultrasensitive thyroid-stimulating hormone, and thyroxine. Participants will be fasting (water only) for blood tests only at screening; fasting is not required at all visits.

[o] Includes hematocrit, hemoglobin, RBC count, WBC count with differential and platelets. Participants will be fasting (water only) for blood tests only at screening; fasting is not required at all visits.

[p] Includes aPTT and INR. Participants will be fasting (water only) for blood tests only at screening; fasting is not required at all timepoints.

[q] Routine ECGs will be calculated and read locally.

[r] Collect PK, PD, and exploratory serum, plasma, and RNA samples prior to study drug administration.

[s] Participants who experience serious or severe hypersensitivity reactions (e.g., hypotension, mucosal involvement) should not receive additional study drug. In addition, in any case of anaphylaxis, anaphylactoid reaction, serious or severe hypersensitivity reaction, ADA, and concurrent PK samples should be collected as close as possible to the event, and then at 4 and 16 weeks post-dose (see schedules of assessments for further details).

[t] Includes amphetamines, barbiturates, benzodiazepines, cannabinoids, cocaine, and opioids.

[u] Urine pregnancy test must be administered and have a negative result prior to any study drug administration. If the participant has discontinued study drug but is continuing with study assessments, the urine pregnancy test may be deferred except for visits during which FDG-PET or florbetapir PET are administered. Serum and urine pregnancy testing will apply to all women unless documented (by medical records or physician's note) to be surgically sterile or postmenopausal as defined in Section 3.4.5.

[v] Vital signs will include measurements of temperature, heart rate, respiratory rate, and systolic and diastolic blood pressure while the participant has been supine for $\geq$ 3 minutes. On study drug administration days, record vital signs prior to study drug administration.

[w] Although visit windowing for dosing days allows for $\pm$ 5 days around any expected visit day, the interval between study drug administrations from one biweekly visit to the following biweekly visit must be between 8 and 20 days.

[x] During the treatment period: Participants who develop $>$ 8 amyloid-related imaging abnormalities hemosiderin deposition cumulatively will receive a lower dose of study drug. Participants who develop $>$ 10 amyloid-related imaging abnormalities–hemosiderin deposition cumulatively will be permanently discontinued from study drug. Cumulative is the sum of amyloid-related imaging abnormalities hemosiderin deposition at baseline and newly detected amyloid-related imaging abnormalities–hemosiderin deposition during the study.

[y] Participants will be observed for a minimum of 15 minutes following SC crenezumab injections.

[z] If MRI and FDG-PET scans are performed on the same day, the FDG-PET scan should be performed first.

[aa] If a participant transitions from SC to IV (higher dose) administration, participants should receive an MRI scan after 3 and 6 months of initiation of SC treatment. Thereafter MRI should be scheduled every 24−28 weeks until end of study or ET.

[bb] Urine pregnancy test must be administered and have a negative result prior to a PET scan. It is recommended, but not required, to use effective contraception during sexual intercourse occurring within 24 hours following a male or female participant receiving a florbetapir PET or FDG-PET scan.

[cc] Participant must be fasting (water only) for a minimum of 4 hours prior to the scan. FDG-PET should be performed a minimum of 12 hours and a maximum of 7 days apart from the accompanying fibrillar amyloid PET scan. For each participant, best efforts should be exerted to perform subsequent FDG-PET scans at the same time of day as the baseline scan.

[dd] The LP for CSF, FDG-PET, and amyloid PET scans is not required at ET (A or B) if they have been done within 24 weeks of ET.

[ee] It is strongly preferred that the LP be performed in the morning. Participants will be required to stay in the clinic or hospital for approximately 1 hour after the LP for safety follow-up. For each participant, best efforts should be exerted to perform subsequent LPs at the same time of day as the baseline LP. See protocol (Section 4.5.1 [l]) for additional details regarding LP.

[ff] FDG-PET and fibrillar amyloid PET scans should be performed only if the investigator has determined that the total radiation exposure from the ET FDG-PET and fibrillar amyloid PET scans does not exceed local safety guidelines.

Appendix A-3
(IV Dosing) Study Flowchart (First IV Dosing–Week 104 [bb])

Timepoint (starting with study visit at which IV dosing was initiated)	W 24	W 28	W 32	W 36	W 40	W 44	W 48	W 52	W 56	W 60	W 64	W 68	W 72	W 76	W 80	W 84	W 88	W 92	W 96	W 100	W 104
	Q4W	Q4W	Q4W	Q4W	Q4W	Q4W	Q4W	Q4W	Q4W	Q4W	Q4W	Q4W	Q4W	Q4W	Q4W	Q4W	Q4W	Q4W	Q4W	Q4W	Q4W
Visit Window	±5d	±5d	±5d	±5d	±5d	±5d	±5d	±5d	±5d	±5d	±5d	±5d	±5d	±5d	±5d	±5d	±5d	±5d	±5d	±5d	±5d
Physical examination	x			x				x						x							x
Weight [a]	x	x	x	x	x	x	x	x	x	x	x	x	x	x	x	x	x	x	x	x	x
Neurological examination	x			x				x						x							x
Evaluate criteria for MCI/AD	x							x						x							x
GDS [b, c]	x							x						x							x
SMC [b, c]	x							x						x							x
Composite Cognitive Battery [b, d]	x							x						x							x
Extended Cognitive Battery [b, e]	x							x						x							x
SPCQ [g, h, c]	x							x						x							x
CDR [b, f, h, c]	x							x						x							x
NPI [h, c]	x							x						x							x
FAST [h, c]	x							x						x							x
Suicidality assessment [b, c]	x			x				x			x			x			x				x
Urinalysis [i]	x			x				x						x							x
Chemistry [j]	x			x				x						x							x
Hematology [k]	x			x				x						x							x
Coagulation [l]	x			x				x						x							x

Appendix A-3
(IV Dosing) Study Flowchart (First IV Dosing–Week 104 [bb]) (cont.)

Timepoint (starting with study visit at which IV dosing was initiated)	W 24	W 28	W 32	W 36	W 40	W 44	W 48	W 52	W 56	W 60	W 64	W 68	W 72	W 76	W 80	W 84	W 88	W 92	W 96	W 100	W 104
	Q4W	Q4W	Q4W	Q4W	Q4W	Q4W	Q4W	Q4W	Q4W	Q4W	Q4W	Q4W	Q4W	Q4W	Q4W	Q4W	Q4W	Q4W	Q4W	Q4W	Q4W
Visit Window	±5d	±5d	±5d	±5d	±5d	±5d	±5d	±5d	±5d	±5d	±5d	±5d	±5d	±5d	±5d	±5d	±5d	±5d	±5d	±5d	±5d
12-lead ECG [m]	x							x													x
PK sample (serum) [n, o]	x			x				x						x							x
PD sample (plasma) [n]	x			x				x						x							x
Exploratory serum, plasma, RNA samples [n]	x			x				x						x							x
ADA sample [o]	x							x						x							x
Urine screen for drugs of abuse [p]								x													x
Urine pregnancy test [q]	x	x	x	x	x	x	x	x	x	x	x	x	x	x	x	x	x	x	x	x	x
Vital signs [r]	x	x	x	x	x	x	x	x	x	x	x	x	x	x	x	x	x	x	x	x	x
Dispense study medication [q, s, t, u]	x	x	x	x	x	x	x	x	x	x	x	x	x	x	x	x	x	x	x	x	x
Concomitant medication review [c]	x	x	x	x	x	x	x	x	x	x	x	x	x	x	x	x	x	x	x	x	x
Adverse event review [c]	x	x	x	x	x	x	x	x	x	x	x	x	x	x	x	x	x	x	x	x	x
Brain MRI [y]	(x) [v]	(x) [v]	(x) [v]	(x) [v]	(x) [v]	(x) [v]	(x) [v]	(x) [v]	(x) [v]	(x) [v]	(x) [v]	(x) [v]	(x) [v]	(x) [v]	(x) [v]	(x) [v]	(x) [v]	(x) [v]	(x) [v]	(x) [v]	(x) [v]
Fibrillar amyloid PET [w, x]																					x
FDG-PET [w, x, y, z]																					x
Lumbar puncture for CSF sample (optional) [aa]																					x

Appendix A-3
(IV Dosing) Study Flowchart (First IV Dosing–Week 104 [bb]) (cont.)

AD = Alzheimer's disease; ADA = anti-drug antibody; CDR = Clinical Dementia Rating; CSF = cerebrospinal fluid; d = day; ET =early termination; ECG = electrocardiogram; FAST =Functional Assessment Staging of Alzheimer's Disease; FCSRT = Free and Cued Selective Reminding Test; FDG = fluorodeoxyglucose; GDS = Geriatric Depression Scale; IxRS = interactive voice or web-based response system; LP = lumbar puncture; MCI = mild cognitive impairment; MMSE = Mini-Mental State Examination; MRI = magnetic resonance imaging; NPI = Neuropsychiatric Inventory; PD = pharmacodynamic; PET = positron emission tomography; PK = pharmacokinetic; Q4W=every 4 weeks; RBANS = Repeatable Battery for the Assessment of Neuropsychological Status; Screen = screening; SMC = Subjective Memory Checklist; SPCQ = Study Partner Characterization Questionnaire; TMT = Trail-Making Test; W = week.

Note: On treatment days, all assessments should be performed prior to dosing unless otherwise noted.

[a] Weight should be obtained at all weeks as marked on the study flowchart.

[b] Cognitive and selected other assessments (GDS, SMC, CDR, and suicidality assessment) should not be administered to the participant immediately after dosing or any potentially stressful procedures (e.g., blood draws, LP, imaging). Also, participants should not perform these assessments while fasting. For each participant, best efforts should be exerted to perform subsequent assessments at the same time of day as the baseline assessments.

[c] In rare instances, these assessments permitted by phone or video technology (see Section 4.5.4).

[d] The API ADAD Composite Cognitive Test Battery should be administered in the following order: MMSE, Multilingual Naming Test, Word List: Memory, CERAD Constructional Praxis, Word List: Recall, Word List: Recognition, Raven's Progressive Matrices–Set A.

[e] The Extended Cognitive Battery should be administered after the API ADAD Composite Cognitive Test Battery.

[f] Study partner portion of the CDR must be administered prior to the participant portion of the CDR.

[g] Completed prior to the administration of all other study partner assessments at each visit.

[h] The SPCQ, CDR (study partner portion), FAST, SMC, and NPI should be administered to the same study partner throughout the study. Best efforts should be exerted in order for the study partner to be present for all other visits requiring study partner assessments. In rare instances, for which the study partner cannot be present in person, these assessments may be administered by telephone. See Appendix B for additional details regarding study partner considerations.

[i] Includes pH, protein, glucose, ketones, bilirubin, blood, specific gravity, leukocyte esterase, urobilinogen, and nitrite, as well as microscopic analysis on the basis of dipstick results.

[j] Includes sodium, potassium, BUN, bicarbonate, estimated glomerular filtration rate, calcium, AST, ALT, γ-glutamyl transpeptidase, total and direct bilirubin, alkaline phosphatase, lactate dehydrogenase, total protein, albumin, glucose, cholesterol, triglycerides, uric acid, creatine phosphokinase, and glycosylated hemoglobin. At screening, this sample will also include vitamin B12, ultrasensitive thyroid-stimulating hormone, and thyroxine. Participants will be fasting (water only) for blood tests only at screening; fasting is not required at all visits.

[k] Includes hematocrit, hemoglobin, RBC count, WBC count with differential and platelets. Participants will be fasting (water only) for blood tests only at screening; fasting is not required at all visits.

[l] Includes aPTT and INR. Participants will be fasting (water only) for blood tests only at screening; fasting is not required at all timepoints.

[m] Routine ECGs will be calculated and read locally.

[n] Collect PK, PD, and exploratory serum, plasma, and RNA samples prior to study drug administration.

[o] Participants who experience serious or severe hypersensitivity reactions (e.g., hypotension, mucosal involvement) should not receive additional study drug. In addition, in any case of anaphylaxis, anaphylactoid reaction, serious or severe hypersensitivity reaction, ADA and concurrent PK samples should be collected as close as possible to the event, and then at 4 and 16 weeks post-dose (see Appendix A-4 for further details).

[p] Includes amphetamines, barbiturates, benzodiazepines, cannabinoids, cocaine, and opioids.

[q] Urine pregnancy test must be administered and have a negative result prior to any study drug administration. If the participant has discontinued study drug but is continuing with study assessments, the urine pregnancy test may be deferred except for visits during which FDG-PET or florbetapir PET are administered. Serum and urine pregnancy testing will apply to all women unless documented (by medical records or physician's note) to be surgically sterile or postmenopausal as defined in Section 3.4.5.

[r] Vital signs will include measurements of temperature, heart rate, respiratory rate, and systolic and diastolic blood pressure while the participant has been supine for ≥3 minutes. On study drug administration days, record vital signs just prior to IV infusion, at the end of IV infusion, and ≥ 60 minutes after the end of the IV infusion.

[s] Participants will be observed for a minimum of 60 minutes following administration of study drug by IV infusion.

[t] Although visit windowing for dosing days allows for ± 5 days around any expected visit day, the interval between study drug administrations from one monthly visit to the following monthly visit must be between 21 and 35 days.

[u] During the treatment period: Participants who develop ≥ 8 amyloid-related imaging abnormalities hemosiderin deposition cumulatively will receive a lower dose of the study drug. Participants who develop ≥ 10 amyloid-related imaging abnormalities-hemosiderin deposition cumulatively will be permanently discontinued from the study drug. Cumulative is the sum of amyloid-related imaging abnormalities hemosiderin deposition at baseline and newly detected amyloid-related imaging abnormalities-hemosiderin deposition during the study.

[v] Following transition from SC to IV (higher dose) administration, participants should receive an MRI scan after 3 and 6 months of initiation of IV treatment. Thereafter, MRI should be scheduled every 24–28 weeks until the end of the study or ET.

[w] Urine pregnancy test must be administered and have a negative result prior to a PET scan. It is recommended, but not required, to use effective contraception during sexual intercourse occurring within 24 hours following a male or female participant receiving a florbetapir PET or FDG-PET scan.

[x] FDG-PET and fibrillar amyloid PET scans should be performed only if the investigator has determined that the total radiation exposure from the ET FDG-PET and fibrillar amyloid PET scans does not exceed local safety guidelines.

[y] If MRI and FDG-PET scans are performed on the same day, the FDG-PET scan should be performed first.

[z] Participant must be fasting (water only) for a minimum of 4 hours prior to the scan. FDG-PET should be performed a minimum of 12 hours and a maximum of 7 days apart from the accompanying fibrillar amyloid PET scan. For each participant, best efforts should be exerted to perform subsequent FDG-PET scans at the same time of day as the baseline scan.

[aa] It is strongly preferred that the LP be performed in the morning. Participants will be required to stay in the clinic or hospital for approximately 1 hour after the LP for safety follow-up. For each participant, best efforts should be exerted to perform subsequent LPs at the same time of day as the baseline LP. See protocol (Section 4.5.1 [l]) for additional details regarding LP.

[bb] For participants who discontinue study drug or the study prior to study completion, see ET-A and ET-B in Appendix A-4 along with corresponding footnotes.

Appendix A-4
(IV Dosing) Study Flowchart (Week 108–End of Study Period A)

Timepoint	From W108, all weeks except those specified to the right	W116, 144, 168, 196, 220, 248, 272, 300, 324, 352, 376, 404, 428	W 128 [a]	W 156 [a]	W 180 [a]	W 208 [a]	W 232 [a]	W 260 [a]	W 284 [a]	W 312 [a]	W 336 [a]	W 364 [a]	W 388 [a]	W 416 [a]	ET-A [a, b]	ET-B [a, c]	Safety Follow-up 16 W last dose [d]
	(Q4W)	(Q4W)	Q4W	Q4W	Q4W	Q4W	Q4W	Q4W	Q4W	Q4W	Q4W	Q4W	Q4W	Q4W			
Visit Window	± 5d	± 5d	± 5d	± 5d	± 5d	± 5d	± 5d	± 5d	± 5d	± 5d	± 5d	± 5d	± 5d	± 5d	± 7d	± 7d	± 7d
Physical examination			x	x	x	x	x	x	x	x	x	x	x	x	x	x	
Weight [e]			x	x	x	x	x	x	x	x	x	x	x	x	x	x	
Neurological examination			x	x	x	x	x	x	x	x	x	x	x	x	x	x	
Evaluate criteria for MCI/AD			x	x	x	x	x	x	x	x	x	x	x	x	x		
GDS [f, g]			x	x	x	x	x	x	x	x	x	x	x	x	x		
Subjective Memory Checklist [f, h]			x	x	x	x	x	x	x	x	x	x	x	x	x		
Composite Cognitive Battery [f, i]			x	x	x	x	x	x	x	x	x	x	x	x	x	x	
Extended Cognitive Battery [f, i]			x	x	x	x	x	x	x	x	x	x	x	x	x		
SPCQ [h, l, g]			x	x	x	x	x	x	x	x	x	x	x	x	x		
CDR [f, h, k,g]			x	x	x	x	x	x	x	x	x	x	x	x	x		
FAST [h,g]			x	x	x	x	x	x	x	x	x	x	x	x	x		
NPI [h,g]			x	x	x	x	x	x	x	x	x	x	x	x	x		
Suicidality assessment [f, g]		x	x	x	x	x	x	x	x	x	x	x	x	x	x	x	
Urinalysis [m]			x	x	x	x	x	x	x	x	x	x	x	x	x	x	
Chemistry [n]			x	x	x	x	x	x	x	x	x	x	x	x	x	x	

Appendix A-4
(IV Dosing) Study Flowchart (Week 108–End of Study Period A) (cont.)

Timepoint	From W108, all weeks except those specified to the right	W116, 144, 168, 196, 220, 248, 272, 300, 324, 352, 376, 404, 428	W 128 [a]	W 156 [a]	W 180 [a]	W 208 [a]	W 232 [a]	W 260 [a]	W 284 [a]	W 312 [a]	W 336 [a]	W 364 [a]	W 388 [a]	W 416 [a]	ET-A [a, b]	ET-B [a, c]	Safety Follow-up 16 W last dose [d]
	(Q4W)	(Q4W)	Q4W	Q4W	Q4W	Q4W	Q4W	Q4W	Q4W	Q4W	Q4W	Q4W	Q4W	Q4W			
Visit Window	± 5d	± 5d	± 5d	± 5d	± 5d	± 5d	± 5d	± 5d	± 5d	± 5d	± 5d	± 5d	± 5d	± 5d	± 7d	± 7d	± 7d
Hematology [o]			x	x	x	x	x	x	x	x	x	x	x	x	x	x	
Coagulation [p]			x	x	x	x	x	x	x	x	x	x	x	x	x	x	
12-lead ECG [q]				x		x		x		x		x		x	x	x	
PK sample (serum) [r, s]			x	x	x	x	x	x	x	x	x	x	x	x	x	x	x
PD sample (plasma) [r]			x	x	x	x	x	x	x	x	x	x	x	x	x	x	x
Exploratory, plasma, serum, RNA samples [r]			x	x	x	x	x	x	x	x	x	x	x	x	x	x	
ADA sample [s]			x	x	x	x	x	x	x	x	x	x	x	x	x	x	x
Urine screen for drugs of abuse [t]				x		x		x		x		x		x	x	x	
Urine pregnancy test [u]	x	x	x	x	x	x	x	x	x	x	x	x	x	x	x	x	x
Vital signs [v]	x	x	x	x	x	x	x	x	x	x	x	x	x	x	x	x	
Dispense study medication [u, w, x, y]	x	x	x	x	x	x	x	x	x	x	x	x	x	x			
Concomitant medication review [g]	x	x	x	x	x	x	x	x	x	x	x	x	x	x	x	x	x
Adverse event review [g]	x	x	x	x	x	x	x	x	x	x	x	x	x	x	x	x	x
Brain MRI [cc]	(x) [z]	(x) [z]	(x) [z]	(x) [z]	(x) [z]	(x) [z]	(x) [z]	(x) [z]	(x) [z]	(x) [z]	(x) [z]	(x) [z]	(x) [z]	(x) [z]	(x) [z]	(x) [z]	
Fibrillar amyloid PET [u, aa]								x							x [ee]	x [ee]	
FDG-PET [u, aa, bb, cc]								x							x [ee]	x [ee]	
Lumbar puncture for CSF sample (optional) [dd]								x							x [ee]	x [ee]	

Appendix A-4
(IV Dosing) Study Flowchart (Week 108–End of Study Period A) (cont.)

AD = Alzheimer's disease; ADA = anti-drug antibody; CDR = Clinical Dementia Rating; CSF = cerebrospinal fluid; d = day; ECG = electrocardiogram; ET = early termination; FAST = Functional Assessment Staging of Alzheimer's Disease; FDG = fluorodeoxyglucose; GDS = Geriatric Depression Scale; IxRS = interactive voice or web-based response system; MCI = mild cognitive impairment; MRI = magnetic resonance imaging; NPI = Neuropsychiatric Inventory; PD = pharmacodynamic; PET = positron emission tomography; PK = pharmacokinetic; Q4W= every 4 weeks; RBANS= Repeatable Battery for the Assessment of Neuropsychological Status; Screen = screening; SMC = Subjective Memory Checklist; SPCQ = Study Partner Characterization Questionnaire; TMT = Trail-Making Test; W = week.

Note: On treatment days, all assessments should be performed prior to dosing unless otherwise noted.

[a] Participants who discontinue from study drug treatment (early discontinuation) will not receive further doses of study drug, but should be asked to stay in the study for subsequent safety, cognitive, and biomarker assessments at complex visits, including at Weeks 24, 36, 52, 76, 104, 128, 156, 180, 208, 232, 260, and every 24–28 weeks thereafter until the study ends. In addition, the suicidality assessment, review of concomitant medications and adverse events should be collected at Weeks 12, 64, 92, 116, 144, 168, 196, 220, 248, and every 24–28 weeks until the study ends; these assessments may be completed by telephone (but it is preferred they be completed in person). If a phone visit it completed during these weeks, vital signs assessments may be omitted.

[b] If a participant discontinues from the study (ET) or discontinues from study drug treatment and elects to no longer stay in the study, ET Visit A should be completed if the last visit occurred more than 12 weeks after functional, behavioral and cognitive assessments were performed;

[c] If a participant discontinues from the study (ET) or discontinues from study drug treatment and elects to no longer stay in the study, ET Visit B should be performed if the last visit occurred within 12 weeks of functional, behavioral, and cognitive assessments.

[d] Safety follow-up 16 weeks after last dose is only applicable to participants that do not consent to participate in the continued access study.

[e] Weight should be obtained at all weeks marked on the study flowchart.

[f] Cognitive and selected other assessments (GDS, SMC, CDR, and suicidality assessment) should not be administered to the participant immediately after dosing or any potentially stressful procedures (e.g., blood draws, LPs, imaging). Also, participants should not perform these assessments while fasting. For each participant, best efforts should be exerted to perform subsequent assessments at the same time of day as the baseline assessments.

[g] In rare instances, these assessments permitted by phone or video technology (see Section 4.5.4).

[h] The SPCQ, CDR (study partner portion), FAST, SMC, and NPI should be administered to the same study partner throughout the study. Best efforts should be exerted in order for the study partner to be present for all other visits requiring study partner assessments. In rare instances, for which the study partner cannot be present in person, these assessments may be administered by telephone. See Appendix B for additional details regarding study partner considerations.

[i] The API ADAD Composite Cognitive Test Battery should be administered in the following order: MMSE, Multilingual Naming Test, Word List: Memory, CERAD Constructional Praxis, Word List: Recall, Word List: Recognition, Raven's Progressive Matrices–Set A.

[j] The Extended Cognitive Battery should be administered after the API ADAD Composite Cognitive Test Battery.

[k] Study partner portion of the CDR must be administered prior to the participant portion of the CDR.

[l] Completed prior to the administration of all other study partner assessments at each visit.

[m] Includes pH, protein, glucose, ketones, bilirubin, blood, specific gravity, leukocyte esterase, urobilinogen, and nitrite, as well as microscopic analysis on the basis of dipstick results.

[n] Includes sodium, potassium, BUN, bicarbonate, estimated glomerular filtration rate, calcium, AST, ALT, γ-glutamyl transpeptidase, total and direct bilirubin, alkaline phosphatase, lactate dehydrogenase, total protein, albumin, glucose, cholesterol, triglycerides, uric acid, creatine phosphokinase, and glycosylated hemoglobin. At screening, this sample will also include vitamin B12, ultrasensitive thyroid-stimulating hormone, and thyroxine. Participants will be fasting (water only) for blood tests only at screening; fasting is not required at all visits.

Appendix A-4
(IV Dosing) Study Flowchart (Week 108–End of Study Period A) (cont.)

[o] Includes hematocrit, hemoglobin, RBC count, WBC count with differential and platelets. Participants will be fasting (water only) for blood tests only at screening; fasting is not required at all visits.

[p] Includes aPTT and INR. Participants will be fasting (water only) for blood tests only at screening; fasting is not required at all timepoints.

[q] Routine ECGs will be calculated and read locally.

[r] Collect PK, PD, and exploratory serum, plasma, and RNA samples prior to study drug administration.

[s] Participants who experience serious or severe hypersensitivity reactions (e.g., hypotension, mucosal involvement) should not receive additional study drug. In addition, in any case of anaphylaxis, anaphylactoid reaction, serious or severe hypersensitivity reaction, ADA, and concurrent PK samples should be collected as close as possible to the event, and then at 4 and 16 weeks post-dose (see study flowchart for further details).

[t] Includes amphetamines, barbiturates, benzodiazepines, cannabinoids, cocaine, and opioids.

[u] Urine pregnancy test must be administered and have a negative result prior to any study drug administration. If the participant has discontinued study drug but is continuing with study assessments, the urine pregnancy test may be deferred except for visits during which FDG-PET or florbetapir PET scans are done. Serum and urine pregnancy testing will apply to all women unless documented (by medical records or physician's note) to be surgically sterile or postmenopausal as defined in Section 3.4.5.

[v] Vital signs will include measurements of temperature, heart rate, respiratory rate, and systolic and diastolic blood pressure while the participant has been supine for ≥ 3 minutes. On study drug administration days, record vital signs just prior to the injection, at the end of IV infusion, and ≥ 60 minutes after the end of the IV infusion

[w] Although visit windowing for dosing days allows for ± 5 days around any expected visit day, the interval between study drug administrations from one monthly visit to the following monthly visit must be between 21 and 35 days.

[x] Participants will be observed for a minimum of 60 minutes following administration of study drug by IV infusion.

[y] During the treatment period: Participants who develop > 8 amyloid-related imaging abnormalities hemosiderin deposition cumulatively will receive a lower dose of the study drug. Participants who develop > 10 amyloid-related imaging abnormalities–hemosiderin deposition cumulatively will be permanently discontinued from the study drug. Cumulative is the sum of amyloid-related imaging abnormalities hemosiderin deposition at baseline and newly detected amyloid-related imaging abnormalities–hemosiderin deposition during the study.

[z] Following transition from SC to IV (higher dose) administration, participants should receive an MRI after 3 and 6 months of initiation of IV treatment. Thereafter, MRI should be scheduled every 24–28 weeks until the end of the study or ET.

[aa] FDG-PET and fibrillar amyloid PET scans should be performed only if the investigator has determined that the total radiation exposure from the ET FDG-PET and fibrillar amyloid PET scans does not exceed local safety guidelines.

[bb] Participant must be fasting (water only) for a minimum of 4 hours prior to the scan. FDG-PET should be performed a minimum of 12 hours and a maximum of 7 days apart from the accompanying fibrillar amyloid PET scan. For each participant, best efforts should be exerted to perform subsequent FDG-PET scans at the same time of day as the baseline scan.

[cc] If MRI and FDG-PET scans are performed on the same day, the FDG-PET scan should be performed first.

[dd] It is strongly preferred that the LP be performed in the morning. Participants will be required to stay in the clinic or hospital for approximately 1 hour after the LP for safety follow-up. For each participant, best efforts should be exerted to perform subsequent LPs at the same time of day as the baseline LP. See protocol (Section 4.5.1 [l]) for additional details regarding LP.

[ee] The LP for CSF, FDG-PET, and amyloid PET scans is not required at ET (A or B) if it has been done within 24 weeks of ET.

Appendix A-5
Study Flow Chart for Study Period B: SC dosing

	Wk 1 and every 2 Wks Thereafter[a]	Wk 13	Wk 25	Wk 53	ET/ SFU Last Dose + 16 Wks[b]
Assessment window	(±7)	(±7)	(±7)	(±7)	(±7)
Informed consent	x[c]				
Vital signs [d]	x	x	x	x	x
Weight			x	x	x
Urinalysis [e]				x	x
Urine pregnancy test [f]	x	x	x	x	x
PK serum sample [g]					x
Serum ADA sample [g]					x
PD plasma sample [h]				x	x
Single 12-Lead ECG [i]				x	x
Physical and neurologic exams [j]				x	x
Serum Chemistry and Hematology [k, l]				x	x
MRI[m]					
Evaluate criteria for MCI/AD			x	x	
GDS [n, o]			x	x	
SMC [n, o]			x	x	

Appendix A-5
Study Flow Chart for Study Period B: SC dosing (cont.)

	Wk 1 and every 2 Wks Thereafter[a]	Wk 13	Wk 25	Wk 53	ET/ SFU Last Dose + 16 Wks[b]
Assessment window	(±7)	(±7)	(±7)	(±7)	(±7)
API ADAD Cognitive Composite Battery [n, p]			x	x	
FCSRT [n]			x	x	
Trail Making Tests A and B [n]			x	x	
RBANS [n]			x	x	
SPCQ [o, q, r]			x	x	
CDR [n, o, q]			x	x	
FAST [o, q]			x	x	
NPI [o, q]			x	x	
Suicidality assessment [n, o]			x	x	
Study drug administration [s, t]	x[u]	x	x	x	
Concomitant medication review [o]	x	x	x	x	x
Adverse event review [o]	x	x	x	x	x

AD = Alzheimer's disease; ADAD = Autosomal Dominant Alzheimer's Disease; ADA = Anti-Drug Antibody; CDR = Clinical Dementia Rating; ET = early termination; FAST = Functional Assessment Staging of Alzheimer's Disease; FCSRT = free and cued selective reminding test; GDS = Geriatric Depression Scale; MCI = mild cognitive impairment; MMSE = Mini-Mental State Examination; MRI = magnetic resonance imaging; NPI = Neuropsychiatric Inventory; PD = pharmacodynamic; PK = pharmacokinetic; RBANS = Repeatable Battery for the Assessment of Neuropsychological Status; SC = subcutaneous; SFU = Safety Follow Up; SMC = Subjective Memory Checklist; SPCQ = Study Partner Characterization Questionnaire; Wk = week.

[a] Wk 1 is the first dose in Study Period B and may occur on the same day as the final ET-A or ET-B visit in Study Period A after all ET assessments are completed and the investigator deems dosing is appropriate.

[b] Early termination visit/Safety Follow Up Visit, occurring when someone terminates prior to the end of the Study Period B, should be done 16 weeks after the last dose of study drug. Participants who continue crenezumab treatment after the end of Study Period B will not have a "last dose + 16 weeks visit".

[c] Informed consent to continue into Study Period B must be obtained at or before the Wk 1 visit in Study Period B.

[d] Vital signs include measurements of pulse rate, systolic and diastolic blood pressure, respiratory rate, and body temperature. The same arm should be used for all blood pressure measurements. Participants should rest ≥ 3 minutes in a quiet room before pulse rate and blood pressure are measured. On study drug administration days, vital sign assessments should be performed prior to study drug administration.

[e] Urinalysis will be performed by dipstick for pH, protein, glucose, ketones, nitrites, bilirubin, blood, specific gravity, leukocyte esterase, urobilinogen. Microscopic examination should be performed at the central laboratory if blood and/or protein results are positive.

[f] Females of childbearing potential (including those who have had a tubal ligation) must have a urine pregnancy test performed at the site prior to each dose administration. If a urine pregnancy test is positive, it must be confirmed by a serum pregnancy test at the central laboratory.

[g] PK samples should be obtained prior to study drug administration on the same day of dosing. Accurate recording of the time of study drug administration and pharmacokinetics is critical. Unscheduled PK samples should be taken in the event of anaphylaxis, anaphylactoid, or serious or severe hypersensitivity reactions. For any participant suspected of developing anaphylaxis, or anaphylactoid or serious or severe hypersensitivity reactions warranting discontinuation of dosing, ADA and concurrent PK samples as well as any clinically relevant samples (such as total tryptase) should be collected as close as possible to the event, and then at 4 and 8 weeks post-dose.

[h] PD plasma sample collection prior to study drug administration.

[i] Perform after the participant has been at rest in a supine position. ECGs for each participant should be obtained from the same machine whenever possible and performed prior to any other assessments (e.g., vital signs).

[j] Complete physical and neurologic examinations should include an evaluation of the head, eyes, ears, nose, and throat, and the cardiovascular, dermatologic, musculoskeletal, respiratory, gastrointestinal, and neurologic systems. Neurologic examinations should include assessment of mental status, level of consciousness, cranial nerve function (including fundoscopic examination), motor function, sensory function, reflexes, coordination, and assessment of gait. Other examination components or systems may be assessed as clinically indicated. Symptom-directed physical and neurologic examinations may be done at any other visit as needed to assess the safety of the participant.

[k] Serum chemistry includes sodium, potassium, BUN, bicarbonate, estimated glomerular filtration rate, calcium, AST, ALT, γ-glutamyl transpeptidase, total and direct bilirubin, alkaline phosphatase, lactate dehydrogenase, total protein, albumin, glucose, cholesterol, triglycerides, uric acid, creatine phosphokinase, and glycosylated hemoglobin.

[l] Hematology includes WBC count with differential, RBC count, hemoglobin, hematocrit, and platelet count

[m] Due to very low risk of ARIA-E (see latest Crenezumab IB), MRIs are not a scheduled activity in Study Period B. However, MRI may be done at any visit if clinically indicated for new or unexplained neurologic symptoms.

[n] Cognitive and selected other assessments (GDS, SMC, CDR, and suicidality assessment) should not be administered to the participant immediately after dosing or any potentially stressful procedures (e.g., blood draws, CSF sampling, imaging). Also, participants should not perform these assessments while fasting. For each participant, best efforts should be exerted to perform subsequent assessments at the same time of day as the baseline assessments.

[o] In exceptional instances, these assessments are permitted by phone or video technology.

[p] The API ADAD Composite Cognitive Test Battery includes the following assessments which should be administered in this order: MMSE, Multilingual Naming Test, Word List: Memory, CERAD Constructional Praxis, Word List: Recall, Word List: Recognition, Raven's Progressive Matrices–Set A. The API ADAD composite should also be administered before administering FCSRT, Trail Making, and RBANS.

[q] The SPCQ, CDR (study partner portion), FAST, SMC, and NPI should be administered to the same study partner throughout the study. Best efforts should be exerted in order for the study partner to be present for all visits requiring study partner assessments. In rare instances, for which the study partner cannot be present in person, these assessments may be administered by telephone.

[r] Completed prior to the administration of all other study partner assessments at each visit.

[s] Study drug administration should be performed only after all assessments/rating scales have been completed (unless indicated otherwise). Study drug will be administered to participants by injection (full details in the pharmacy manual). Participants must be observed for a minimum of 15 minutes after dosing.

[t] Although visit windowing for dosing days allows for ± 7 days around any expected visit day, the interval between study drug administrations from one biweekly visit to the following biweekly visit must be between 8 and 20 days.

[u] There should be a telephone call to the participant the day after the first infusion.

Appendix A-6
Study Flow Chart for Study Period B: IV dosing

	Wk 1 and every 4 Wks Thereafter[a]	Wk 13	Wk 25	Wk 53	ET/ SFU Last Dose + 16 Wks[b]
Assessment window	(±7)	(±7)	(±7)	(±7)	(±7)
Informed consent	x[c]				
Vital signs [d]	x	x	x	x	x
Weight	x	x	x	x	x
Urinalysis [e]				x	x
Urine pregnancy test [f]	x	x	x	x	x
PK serum sample [g]					x
Serum ADA sample [g]					x
PD plasma sample [h]				x	x
Single 12-Lead ECG [i]				x	x
Physical and neurologic exams [j]				x	x
Serum Chemistry and Hematology [k, l]				x	x
MRI[m]					
Evaluate criteria for MCI/AD			x	x	
GDS [n, o]			x	x	
SMC [n, o]			x	x	

Appendix A-6
Study Flow Chart for Study Period B: IV dosing

	Wk 1 and every 4 Wks Thereafter[a]	Wk 13	Wk 25	Wk 53	ET/ SFU Last Dose + 16 Wks[b]
Assessment window	(±7)	(±7)	(±7)	(±7)	(±7)
API ADAD Cognitive Composite Battery [n, p]			x	x	
FCSRT [n]			x	x	
Trail Making Tests A and B [n]			x	x	
RBANS [n]			x	x	
SPCQ [o, q, r]			x	x	
CDR [n, o, q]			x	x	
FAST [o, q]			x	x	
NPI [o, q]			x	x	
Suicidality assessment [n, o]			x	x	
Study drug administration [s, t]	x[u]	x	x	x	
Concomitant medication review [o]	x	x	x	x	x
Adverse event review [o]	x	x	x	x	x

AD = Alzheimer's disease; ADAD = Autosomal Dominant Alzheimer's Disease; ADA = Anti-Drug Antibody; CDR = Clinical Dementia Rating; ET = early termination; FAST = Functional Assessment Staging of Alzheimer's Disease; FCSRT = free and cued selective reminding test; GDS = Geriatric Depression Scale; IV = intravenous; MCI = mild cognitive impairment; MMSE = Mini-Mental State Examination; MRI = magnetic resonance imaging; NPI = Neuropsychiatric Inventory; PD = pharmacodynamic; PK = pharmacokinetic; RBANS= Repeatable Battery for the Assessment of Neuropsychological Status; SFU = Safety Follow Up; SMC = Subjective Memory Checklist; SPCQ = Study Partner Characterization Questionnaire; Wk = week.

[a] Wk 1 is the first dose in Study Period B and may occur on the same day as the final ET-A or ET-B visit in Study Period A after all ET assessments are completed and the investigator deems dosing is appropriate.

[b] Early termination visit/Safety Follow Up Visit, occurring when someone terminates prior to the end of the Study Period B, should be done 16 weeks after the last dose of study drug. Participants who continue crenezumab treatment after the end of Study Period B will not have a "last dose + 16 weeks visit".

Appendix A-6
Study Flow Chart for Study Period B: IV dosing

[c] Informed consent to continue into Study Period B must be obtained at or before the Wk 1 visit in Study Period B.

[d] Vital signs include measurements of pulse rate, systolic and diastolic blood pressure, respiratory rate, and body temperature. The same arm should be used for all blood pressure measurements. Participants should rest for ≥ 3 minutes in a quiet room before pulse rate and blood pressure are measured. On study drug administration days, vital sign assessments should be performed just prior to the infusion, at the end of IV infusion, and ≥ 60 minutes after the end of the IV infusion.

[e] Urinalysis will be performed by dipstick for pH, protein, glucose, ketones, nitrites, bilirubin, blood, specific gravity, leukocyte esterase, urobilinogen. Microscopic examination should be performed at the central laboratory if blood and/or protein results are positive.

[f] Females of childbearing potential (including those who have had a tubal ligation) must have a urine pregnancy test performed at the site prior to each dose administration. If a urine pregnancy test is positive, it must be confirmed by a serum pregnancy test at the central laboratory.

[g] PK samples should be obtained prior to study drug administration on the same day of dosing. Accurate recording of the time of study drug administration and pharmacokinetics is critical. Unscheduled PK samples should be taken in the event of anaphylaxis, anaphylactoid, or serious or severe hypersensitivity reactions. For any participant suspected of developing anaphylaxis, or anaphylactoid or serious or severe hypersensitivity reactions warranting discontinuation of dosing, ADA and concurrent PK samples as well as any clinically relevant samples (such as total tryptase) should be collected as close as possible to the event, and then at 4 and 8 weeks post-dose.

[h] PD plasma sample collection prior to study drug administration.

[i] Perform after the participant has been at rest in a supine position. ECGs for each participant should be obtained from the same machine whenever possible and performed prior to any other assessments (e.g., vital signs).

[j] Complete physical and neurologic examinations should include an evaluation of the head, eyes, ears, nose, and throat, and the cardiovascular, dermatologic, musculoskeletal, respiratory, gastrointestinal, and neurologic systems. Neurologic examinations should include assessment of mental status, level of consciousness, cranial nerve function (including fundoscopic examination), motor function, sensory function, reflexes, coordination, and assessment of gait. Other examination components or systems may be assessed as clinically indicated. Symptom-directed physical and neurologic examinations may be done at any other visit as needed to assess the safety of the participant.

[k] Serum chemistry includes sodium, potassium, BUN, bicarbonate, estimated glomerular filtration rate, calcium, AST, ALT, γ-glutamyl transpeptidase, total and direct bilirubin, alkaline phosphatase, lactate dehydrogenase, total protein, albumin, glucose, cholesterol, triglycerides, uric acid, creatine phosphokinase, and glycosylated hemoglobin.

[l] Hematology includes WBC count with differential, RBC count, hemoglobin, hematocrit, and platelet count.

[m] Due to very low risk of ARIA-E (see latest Crenezumab IB), MRIs are not a scheduled activity in Study Period B. However, MRI may be done at any visit if clinically indicated for new or unexplained neurologic symptoms.

[n] Cognitive and selected other assessments (GDS, SMC, CDR, and suicidality assessment) should not be administered to the participant immediately after dosing or any potentially stressful procedures (e.g., blood draws, CSF sampling, imaging). Also, participants should not perform these assessments while fasting. For each participant, best efforts should be exerted to perform subsequent assessments at the same time of day as the baseline assessments.

[o] In exceptional instances, these assessments are permitted by phone or video technology.

[p] The API ADAD Composite Cognitive Test Battery includes the following assessments which should be administered in this order: MMSE, Multilingual Naming Test, Word List: Memory, CERAD Constructional Praxis, Word List: Recall, Word List: Recognition, Raven's Progressive Matrices–Set A. The API ADAD composite should also be administered before administering FCSRT, Trail Making, and RBANS.

[q] The SPCQ, CDR (study partner portion), FAST, SMC, and NPI should be administered to the same study partner throughout the study. Best efforts should be exerted in order for the study partner to be present for all visits requiring study partner assessments. In rare instances, for which the study partner cannot be present in person, these assessments may be administered by telephone.

[r] Completed prior to the administration of all other study partner assessments at each visit.

[s] Study drug administration should be performed only after all assessments/rating scales have been completed (unless indicated otherwise). Study drug will be administered to participants by injection (full details in the pharmacy manual). Participants must be observed for a minimum of 60 minutes after dosing.

[t] Although visit windowing for dosing days allows for ± 7 days around any expected visit day, the interval between study drug administrations from one monthly visit to the following monthly visit must be between 21 and 35 days.

[u] There should be a telephone call to the participant the day after the first infusion.

Appendix B
Neurocognitive and Functional Assessments

Several cognitive, functional, and behavioral assessments will be administered during the course of this study. These assessments will be given to the participant, the study partner, or both. The cognitive test batteries include a Screening Battery, API ADAD Composite Cognitive Test Battery, and the Extended Cognitive Battery. Additional functional and behavioral assessments and other self-reported questionnaires will also be administered. The various assessments are described briefly below.

Screening Battery

The Screening Battery consists of the MMSE (Folstein et al. 1975) and the Word List (Rosen et al. 1984; Morris et al. 1989; Mohs et al. 1997). The scores obtained on these assessments will be used in determining trial eligibility. Descriptions of these tests are provided below.

Mini-Mental State Examination (MMSE)

The MMSE (Folstein et al. 1975) is a screening instrument frequently used for Alzheimer's disease drug studies (Folstein and McHugh 1975). It evaluates orientation, memory, attention, concentration, naming, repetition, comprehension, the ability to create a sentence, and copy a figure. Scores range from 0 to 30.

Word List

Word List is a measure of delayed verbal memory that is administered per the CERAD test protocol (Morris et al. 1989), comprised of four parallel word lists derived from the ADAS-Cog word pool (Rosen et al. 1984; Mohs et al. 1997). Word List consists of three components: Memory, Recall, and Recognition. Scores range from 0–30 for the Word List: Memory, 0–10 for Word List: Recall, and 0–10 for Word List: Recognition. Although all components of the Word List will be administered, for calculation of the composite cognitive endpoint, only the Word List: Recall score will be used.

API ADAD Composite Cognitive Test Battery

The tests comprising the API ADAD Composite Cognitive Test Battery were selected using over 10 years of neuropsychological longitudinal data collected from the Colombia PSEN1 E280A population and applying specific statistical techniques to extract the test combination that is most sensitive to cognitive decline and progression. The API ADAD Composite Cognitive Test Battery was chosen for its sensitivity to measure clinical change in this and other at-risk populations after controlling for practice effects.
The battery consists of the MMSE (orientation to time), Word List: Recall, CERAD Constructional Praxis, Multilingual Naming Test, and Raven's Progressive Matrices.

Mini-Mental State Exam (MMSE)

The MMSE (Folstein et al. 1975) is a screening instrument frequently used for Alzheimer's disease drug studies (Folstein et al. 1975). It evaluates orientation, memory, attention, concentration, naming, repetition, comprehension, the ability to create a sentence and copy a figure. Total score ranges from 0 to 30. For calculation of the API ADAD Composite Cognitive endpoint, only the Orientation to Time score from the MMSE, which ranges from 0 to 5, will be used.

Word List

Word List is a measure of delayed verbal memory that is administered per the CERAD test protocol (Morris et al. 1989), comprised of four parallel word lists derived from the ADAS-Cog word pool (Rosen et al. 1984; Mohs et al., 1997). Word List consists of three components: Memory, Recall, and Recognition. Scores range from 0–30 for the Word List: Memory, 0–10 for Word List: Recall, and 0–10 for Word List: Recognition. Although all components of the Word List will be administered, for calculation of the API ADAD Composite Cognitive endpoint, only the Word List: Recall score will be used.

Consortium to Establish a Registry for Alzheimer's Disease (CERAD) Constructional Praxis – Immediate Recall

The CERAD (Rosen et al. 1984; Morris et al. 1989) neuropsychological test battery has been used widely for evaluation of the cognitive deficits associated with AD. It has also been used by Dr. Lopera and colleagues in presymptomatic longitudinal research after being adapted to the cultural and linguistic idiosyncrasies of the Colombian culture (CERAD Col) (Aguirre-Acevedo et al. 2007). The CERAD Constructional Praxis test consists of four line drawings of increasing complexity. Participants are shown a figure and are then asked to immediately recall the figure. Scores range from 0 to 11.

Multilingual Naming Test (Gollan et al. 2012)

The Multilingual Naming Test is a measure of visual confrontation naming that requires the participant to name objects depicted in outline drawings. The drawings are graded in difficulty, with the easiest drawings presented first. Difficulty of items is assigned by frequency of occurrence. This study will be using a 15-item subset of the 67 pictures comprising the complete test. This subset consists of five drawings from each of the low, medium, and high frequency categories. Total score ranges from 0–15.

Raven's Progressive Matrices (Raven 1976)

This is a non-verbal, multiple-choice measure of general ability and reasoning in the visual modality. It was designed to be culturally nonbiased, as neither language nor academic skills are required to answer items successfully. The test requires conceptualization of spatial design and numerical relationships with varying levels of difficulty. For each item a participant is asked to identify the missing component to

complete a pattern. For this study, only Set A, consisting of 12 items, will be administered. Total scores range from 0–12.

Extended Cognitive Battery

The Extended Cognitive Battery consists of Free and Cued Selective Reminding Task (FCSRT) (Grober and Buschke 1987), Trail Making Test (Armitage 1946), and Repeatable Battery for the Assessment of Neuropsychological Status (RBANS) (Randolph 1998).

Free and Cued Selective Reminding Task (FCSRT)

The Buschke FCSRT (Grober and Buschke 1987) provides assessment of immediate and delayed verbal episodic memory, using Controlled Learning to optimize encoding specificity for more effective recall (Grober et al. 1997; Buschke 1984). For this test, 16 items, each from a different semantic category, is presented on cards, four to a card. The participant's task is to learn the items, and attempt a free recall of the items, followed by a semantically cued recall of items not spontaneously produced by the participant during free recall. There are a total of three learning trials, each preceded by 20 seconds of an interfering cognitive task (counting backwards). For the first two trials, the participant will be reminded of any item not recalled in the cued recall. The free and cued recall assessment occurs both immediately, after each of the three learning trials, and again after a delay of approximately 30 minutes. A unique characteristic of this test is its use of controlled, semantic encoding by providing category cues during the learning (encoding) phase of the test. This test is thought to be sensitive to conditions, such as AD that compromise the functioning of the hippocampus and connected networks.

Trail Making Test: Parts A and B (Armitage 1946)

Part A consists of 25 circles, numbered 1 through 25, distributed over a white sheet of 8 1/2" × 11" paper. The participant is instructed to connect the circles with a drawn line as quickly as possible in ascending numerical order. Part B also consists of 25 circles, but these circles are either numbered (1 through 13) or contain letters (A through L). Now the participant must connect the circles while alternating between numbers and letters in an ascending order (e.g., 1 to A; A to 2; 2 to B; B to 3, etc.). The participant's performance is judged in terms of the time (in seconds) required to complete each trail, the number of digits correctly connected, and by the number of errors of commission. The time to complete Part A (180-second maximum) and B (300-second maximum) will be the primary measures of interest (testing is stopped if the maximum time is reached). Although both Parts A and B depend on visuomotor and perceptual-scanning skills, Part B also requires considerable cognitive flexibility in shifting from number to letter sets under time pressure. Both parts of the Trail-Making Test are available in multiple forms of equal difficulty for purposes of repeated evaluation.

Repeatable Battery for the Assessment of Neuropsychological Status (RBANS)

The RBANS (Randolph 1998) is a standardized brief, individually administered neurocognitive battery measuring attention, language, visuospatial/constructional abilities, and immediate and delayed memory. The test, available in four parallel versions, consists of 12 subtests.

Other Assessments

Subjective Memory Checklist (SMC)

The SMC (Acosta-Baena et al. 2011) is an assessment developed by researchers at the University of Antioquia to collect subjective ratings of memory status from both a participant and an informant. Participants and informants are asked to rate how often the given participant has difficulties relating to memory and thinking in a variety of areas. The scale employs a Likert-type scale, including rating options of 0 (never), 1 (rarely), 2 (often), and 3 (always). The SMC has a study partner portion, as well as a participant portion.

Suicidality Assessment

The suicidality assessment is a tool used to assess the suicidality of a participant throughout the trial. It will consist of two questions, administered to the participant, which assess current and previous suicidal ideation/behavior, followed by a global assessment of suicidality by a qualified clinician. Questions to be included in this assessment are as follows:

- Since your last assessment (or, at the baseline visit, in the last 30 days):

34. Have you thought you would be better off dead, wished you were dead, or had thoughts of killing yourself in any way?

❒ YES ❒ NO

35. Have you done anything on purpose to harm yourself or tried to kill yourself?

❒ YES ❒ NO

Global Assessment by qualified clinician

36. Does the subject appear suicidal?

❒ YES ❒ NO

If the response is “YES” to Questions 1 or 2, or if the response to Question 3 reveals a concern about a significant level of suicidality, the subject would undergo a more detailed assessment by a qualified clinician who has experience in the evaluation of suicidal ideation and behavior, either at the site or by referral to an outside clinician. In either case, appropriate documentation of the clinical issues and management plan will be required in a narrative that would be part of the participant’s study record.

Because of concerns about burden in such a long trial, the protocol is designed in such a way as to try to achieve the trial's overarching goals while balancing feasibility and burden issues. In the case of assessment of suicidality in this preclinical population, some suicidality assessments will be allowed to be conducted by telephone, using the same staff that would conduct these assessments in person and who would already know the participant.

Clinical Dementia Rating (CDR)

The standard CDR (Morris 1993) describes five degrees of impairment in performance on each of six categories of cognitive functioning, including memory, orientation, judgment and problem solving, community affairs, home and hobbies, and personal care. The ratings of degree of impairment obtained on each of the six categories of function are synthesized into one global rating of dementia (ranging from 0 to 3), with more refined measure of change available by use of the CDR Sum of Boxes (Berg et al. 1992). Reliability and validity has been established, as has high inter-rater reliability. This will be used as a global measure of severity of cognitive and functional impairment.

Geriatric Depression Scale (GDS)

The GDS (Sheikh and Yesavage 1986) (Short Form) is a scale designed to identify symptoms of depression in the elderly. The scale consists of 15 printed questions that the participant is asked to answer on the basis of how they felt over the past week. The items are presented on a single page with more benign items presented first. One point is given for each appropriate answer indicative of a symptom of depression, for a possible total of 15 points. Total scores of 0–4 are considered normal, scores of 5–8 suggest mild depression, scores of 9–11 suggest moderate depression, and scores of 12–15 suggest severe depression.

Study Partner Assessments

The following assessments will be administered to the study partner. As noted above, some of these will be performed in person at screening and baseline; it is preferable that they are performed in person. They may be performed by telephone at some visits. The CDR has a study partner portion in addition to participant portion and should be performed in person, when possible.

Study Partner Characterization Questionnaire (SPCQ)

The SPCQ is a brief set of questions developed internally by Banner and designed to identify the study partner, assess the participant's living arrangements (living alone versus with others; identification of others living with participant), and assess the study partner's relationship with the participant.

Neuropsychiatric Inventory Questionnaire (NPI)

The NPI (Kaufer et al. 2000; Cummings et al. 1994) is a well-validated, reliable, multi-item instrument to assess psychopathology in AD, as well as level of distress experienced by the informant in response to the presence of a given feature. The

assessment is on the basis of an interview with an informant and is relatively brief (< 15 minutes). These properties make it well suited for a multicenter trial. It evaluates both the presence and severity and informant distress of 10 neuropsychiatric features, including delusions, hallucinations, dysphoria, anxiety, agitation/aggression, euphoria, disinhibition, irritability, lability, apathy, and aberrant motor behavior.

Functional Assessment Staging Test (FAST)

The FAST (Reisberg 1988) was developed for use with Alzheimer's disease (AD) patients to stage a patient's level of disability with respect to AD. The FAST is comprised of 7 major levels of functioning (from normal adult to severe AD); levels 6 and 7 are additionally divided into substages (11 total). The FAST is derived from Axis V of the Brief Cognitive Rating Scale (BCRS) (Reisberg et al. 1983), which itself is derived from the Global Deterioration Scale (Reisberg et al. 1982). The stages and substages are, thus, designed to correlate with the Global Deterioration Scale global level of cognition and functional capacity measures (Sclan and Reisberg 1992).

Clinical Dementia Rating (CDR) (Morris 1993)

See Other Assessments for description of study partner portion.

Subjective Memory Checklist (SMC) (Acosta-Baena et al. 2011)

See Other Assessments for description.

REFERENCES (Appendix B only)

Acosta-Baena N, Sepulveda-Falla D, Lopera-Gomez CM, et al. Pre-dementia clinical stages in presenilin 1 E280A familial early-onset Alzheimer's disease: a retrospective cohort study. Lancet Neurol 2011;10:213–20.

Aguirre-Acevedo DC, Gómez RD, Moreno S, et al. Validity and reliability of the CERAD Col neuropsychological battery. Rev Neurol 2007;45:655–60.

Armitage SG. An analysis of certain psychological tests used for the evaluation of brain injury. Psychological Monographs 1946;60: i–48.

Berg L, Miller JP, Baty J, et al. Mild senile dementia of the Alzheimer type. 4. Evaluation of intervention. Ann Neurol 1992;31:242–9.

Buschke H. Cued recall in amnesia. J Clin Neuropsychol 1984;6:433–40.

Cummings JL, Mega M, Gray K, et al. The Neuropsychiatric Inventory: comprehensive assessment of psychopathology in dementia. Neurology 1994;44:2308–14.

Folstein MF, Folstein SE, McHugh PR. "Mini-mental state." A practical method for grading the cognitive state of patients for the clinician. J Psychiatr Res 1975;12:189–98.

Gollan TH, Weissberger G, Runnqvist E, et al. Self-ratings of spoken language dominance: A multi-lingual naming test (MINT) and preliminary norms for young and aging Spanish-English bilinguals. Bilingualism: Language and Cognition 2012;15:594–615.

Grober E, Buschke H. Genuine memory deficits in dementia. Dev Neuropsychol 1987;3:13–36.

Grober E, Merling A, Heimlich T, et al. Free and cued selective reminding and selective reminding in the elderly. J Clin Exp Neuropsychol 1997;19:643–54.

Kaufer DI, Cummings JL, Ketchel P, et al. Validation of the NPI-Q, a brief clinical form of the Neuropsychiatric Inventory. J Neuropsychiatry Clin Neurosci 2000;12:233–9.

Mohs RC, Knopman D, Petersen RC, et al. Development of cognitive instruments for use in clinical trials of antidementia drugs: additions to the Alzheimer's Disease Assessment Scale that broaden its scope. Alzheimer Dis Assoc Disord 1997;11(S2):S13–S21.

Morris JC. The Clinical Dementia Rating (CDR): current version and scoring rules. Neurology 1993;43:2412–14.

Morris JC, Heyman A, Mohs RC, et al. The Consortium to Establish a Registry for Alzheimer's Disease (CERAD). Part I. Clinical and neuropsychological assessment of Alzheimer's disease. Neurology 1989;39:1159–65.

Randolph C. Repeatable Battery for the Assessment of Neuropsychological Status. San Antonio, TX: The Psychological Coporation, 1998.

Raven JC. Revised Manual for Raven's Progressive Matrices and Vocabulary Scale. New York: The Psychological Corporation, Harcourt Brace Jovanovich, Inc., 1976.

Reisberg B. Functional assessment staging (FAST). Psychopharmacol Bull 1988;24:653–9.

Reisberg B, Ferris SH, de Leon MJ, et al. The Global Deterioration Scale for assessment of primary degenerative dementia. Am J Psychiatry 1982;139:1136–9.

Reisberg B, Schneck M, Ferris S, et al. The Brief Cognitive Rating Scale (BCRS): findings in primary degenerative dementia. Psychopharmacol Bull 1983;47:47–50.

Rosen WG, Mohs RC, Davis KL. A new rating scale for Alzheimer's disease. Am J Psychiatry 1984;141:1356–64.

Sclan S G and Reisberg B. Functional assessment staging (FAST) in Alzheimer's disease: reliability, validity, and ordinality. Int Psychogeriatr 1992;4:55–69.

Sheikh RL and Yesavage JA. Geriatric Depression Scale (GDS). Recent evidence and development of a shorter version. Clinical Gerontologist 1986;5:165–73.

STATISTICAL ANALYSIS PLAN

STUDY TITLE: **A DOUBLE-BLIND, PLACEBO-CONTROLLED PARALLEL-GROUP STUDY IN PRECLINICAL PSEN1 E280A MUTATION CARRIERS RANDOMIZED TO CRENEZUMAB OR PLACEBO, AND IN NON-RANDOMIZED, PLACEBO-TREATED NON-CARRIERS FROM THE SAME KINDRED, TO EVALUATE THE EFFICACY AND SAFETY OF CRENEZUMAB IN THE TREATMENT OF AUTOSOMAL-DOMINANT ALZHEIMER'S DISEASE**

STUDY NUMBER: GN28352

VERSION NUMBER: 2

TEST COMPOUND: Crenezumab (RO5490245)

EUDRACT NUMBER: TBD

IND NUMBER: 100839 (Non-IND study)

NCT NUMBER: NCT01998841

SPONSOR'S LEGAL REGISTERED ADDRESS: F. Hoffmann-La Roche Ltd
Grenzacherstrasse 124
4070 Basel, Switzerland

DATE FINAL: See electronic date stamp above

PLAN PREPARED BY: [redacted] Ph.D.

Date and Time(UTC)	Rea[redacted]ning	Name
09-Mar-2022 08:09:39	Company Signatory	[redacted]

STATISTICAL ANALYSIS PLAN AMENDMENT APPROVAL

CONFIDENTIAL

This is an F. Hoffmann-La Roche Ltd document that contains confidential information. Nothing herein is to be disclosed without written consent from F. Hoffmann-La Roche Ltd.

STATISTICAL ANALYSIS PLAN VERSION HISTORY

This Statistical Analysis Plan (SAP) was developed based on Roche SAP model document Version 2.0, 26 October 2020.

SAP Version	Approval Date	Based on Protocol (Version, Approval Date)
2	See electronic date stamp on title page	Version 10, 23 August 2021
1	2 September 2021	Version 9, 19 March 2021

STATISTICAL ANALYSIS PLAN AND AMENDMENT RATIONALE

The SAP has been amended to update the safety analysis to include 16-week follow-up data and to update the type I error proof of the blinded power analysis. In particular, the protocol indicates that adverse events reported during the 16-week follow-up period of Study Period A will be summarized separately but according to this SAP, those events will be included in the analysis of Study Period A. The rationale of this departure from the protocol is explained in Section 5.6.

Key changes to the SAP, along with the rationale(s) for each change, are summarized below.

Section	Description of Change	Rationale for Change
1, 5.6	Adverse events reported during the 16-week follow-up period in Study Period A will not be summarized separately.	Study participants are considered treatment-exposed during the 16-week follow-up period (approximately 5 half-lives).
1.2.4	Details clarifying the process by which control cases are identified for adjudication are added.	
5	Types of analyses from Study Period A are clarified.	
5.3.2, 5.3.4	The main analysis and sensitivity analysis have been modified to include medications that may impact cognition.	
5.3.3, 5.4	The order of removing stratification factors is clarified.	
5.3.4	Sensitivity analysis changed to “first occurrence of discontinuing from crenezumab” instead of “first occurrence of discontinuing from study from the crenezumab arm”.	If a participant discontinues from the study, then no subsequent data is expected to be collected.
5.6.2	The list of adverse events with special interested is augmented.	
5.6.8	Rationale of only analyzing partial anti-drug antibody data is added.	
Appendix 2	Mathematical proof of the blinded power analysis type I error control is generalized to both strong and weak sense.	

Additional minor changes have been made throughout to improve clarity and consistency.

TABLE OF CONTENTS

STATISTICAL ANALYSIS PLAN VERSION HISTORY 2

STATISTICAL ANALYSIS PLAN AND AMENDMENT RATIONALE 3

LIST OF ABBREVIATIONS AND DEFINITIONS OF TERMS 7

1. INTRODUCTION 9
 1.1 Objectives and Endpoints 9
 1.2 Study Design 12
 1.2.1 Study Period A 12
 1.2.2 Study Period B 13
 1.2.3 Treatment Assignment and Blinding 14
 1.2.4 Independent Progression Adjudication Committee 16
 1.2.5 Data Monitoring 17

2. STATISTICAL HYPOTHESES 17

3. SAMPLE SIZE DETERMINATION 17
 3.1 Type I Error Control 18

4. ANALYSIS SETS 20

5. STATISTICAL ANALYSES 21
 5.1 General Considerations 21
 5.2 Participant Disposition 22
 5.3 Primary Endpoints Analysis 22
 5.3.1 Definition of Primary Endpoints 22
 5.3.2 Estimands 22
 5.3.3 Main Analytical Approach for Primary Endpoint Family 23
 5.3.4 Sensitivity Analyses for Primary Endpoints 24
 5.3.5 Supplementary Analyses for Primary Endpoints 26
 5.3.5.1 Subgroup Analyses for Primary Endpoints 27
 5.4 Secondary Endpoints Analyses 27
 5.4.1 Key/Confirmatory Secondary Endpoints 28
 5.4.2 Supportive Secondary Endpoints 29
 5.5 Exploratory Endpoints Analysis 29

5.5.1 Clinical ... 29
5.5.2 Fluid Biomarkers ... 30
5.5.3 Imaging Biomarkers ... 30
5.5.4 Other Endpoints ... 30
5.6 Safety Analyses ... 30
5.6.1 Extent of Exposure ... 31
5.6.2 Adverse Events ... 32
5.6.3 Clinical Laboratory Data ... 33
5.6.4 MRI Evaluations ... 33
5.6.5 Vital Signs ... 33
5.6.6 Electrocardiograms ... 33
5.6.7 Suicidal Ideation and Behavior ... 33
5.6.8 Anti-Drug Antibodies ... 33
5.7 Other Analyses ... 34
5.7.1 Summaries of Conduct of Study ... 34
5.7.2 Summaries of Treatment Group Comparability/Demographics and Baseline Characteristics ... 34
5.7.3 Summaries COVID-19 Impact on the Trial ... 34
5.7.4 Pharmacokinetic and Pharmacodynamic Analyses ... 34
5.8 Analyses in TAU PET LongItudinal Substudy BN40199 ... 35
5.9 Interim Analyses ... 35
5.10 Analyses for Period B and Open-Label Extension Study ... 35
5.10.1 Efficacy Analyses ... 36
5.10.1.1 Delayed-Start Analyses ... 36
5.10.1.2 Rank-Preserving Structural Failure Time Analyses ... 38
5.10.2 Safety Analyses ... 39
5.10.3 Other Analyses ... 39
6. SUPPORTING DOCUMENTATION ... 39
7. REFERENCES ... 50

LIST OF TABLES

Table 1 Objectives and Endpoints ... 9

LIST OF FIGURES

Figure 1 Study Design 14
Figure 2 Study Schematic 14
Figure 3 Graph-Based Testing Procedure for Type I Error Control 19
Figure 4 Illustration of Delayed-Start Analyses Using Data from the Beginning of Period A of Study GN28352 to the End of *Study Period B or* OLE 37
Figure 5 Testing Hierarchy for Delayed-Start Analyses 38

LIST OF APPENDICES

Appendix 1 Changes to Protocol-Planned Analyses 40
Appendix 2 Blinded Power Analysis and its Impact on Type I Error 41
Appendix 3 Details on Selected Statistical Models 48

LIST OF ABBREVIATIONS AND DEFINITIONS OF TERMS

Abbreviation or Term	Description
AD	Alzheimer's disease
ADAD	autosomal-dominant Alzheimer's disease
ADA	anti-drug antibody
API	Alzheimer's Prevention Initiative
APOE	apolipoprotein E
ARIA-E	amyloid-related imaging abnormalities–edema/effusion
ARIA-H	amyloid-related imaging abnormalities–hemosiderin deposition
BP	blinded power
CDR	Clinical Dementia Rating
CERAD	Consortium to Establish a Registry for Alzheimer's Disease
COVID-19	coronavirus disease 2019
CSF	cerebrospinal fluid
CSR	Clinical Study Report
ET-A	end of treatment visit A
ET-B	end of treatment visit B
FCSRT	Free and Cued Selective Reminding Task
FDA	U.S. Food and Drug Administration
FDG	fluorodeoxyglucose
GDS	Geriatric Depression Scale
HR	hazard ratio
iDMC	independent Data Monitoring Committee
iPAC	independent progression adjudication committee
ITT	intent-to-treat
IxRS	interactive voice or Web-based response system
LS	least squares
MCI	mild cognitive impairment
mITT	modified intent-to-treat
MMRM	mixed model with repeated measure
MMSE	Mini-Mental State Examination
MRI	magnetic resonance imaging
NCI CTCAE	National Cancer Institute Common Terminology Criteria for Adverse Events
NfL	neurofilament light
OLE	open-label extension

Abbreviation or Term	Description
PACC	Preclinical Alzheimer Cognitive Composite
PET	positron emission tomography
PD	pharmacodynamic
PK	pharmacokinetic
RBANS	Repeatable Battery for the Assessment of Neuropsychological Status
RCRM	random coefficient regression model
ROI	region of interest
RPSFT	rank-preserving structural failure time
RR	relative reduction
SAP	Statistical Analysis Plan
SE	standard error
SUVr	standardized uptake value ratio
TTE	time-to-event

1. INTRODUCTION

This Statistical Analysis Plan (SAP) provides details of the planned analyses and statistical method for the clinical efficacy and clinical safety for Period A of Study GN28352. *The analyses specified in this document supersede the analysis plan described in the study protocol and the previous version of the SAP.* After *the primary analysis results* from Period A of Study GN28352 are available, the Sponsor may *extend Study Period B, or* initiate an open-label extension (OLE) study for *PSEN1* E280A autosomal-dominant mutation carriers. Key efficacy analyses and general descriptions of safety and other analyses in Period B of Study GN28352 and in *the* potential OLE *study* are also included in *Section 5.10 of* this SAP.

1.1 OBJECTIVES AND ENDPOINTS

Table 1 Objectives and Endpoints

Primary Objectives	Corresponding Endpoints
• To evaluate the efficacy of crenezumab treatment compared with placebo for at least 260 weeks in change in cognitive function as measured by the API ADAD Cognitive Composite Test total score in preclinical *PSEN1* E280A autosomal-dominant mutation carriers<br>• To evaluate the efficacy of crenezumab treatment compared with placebo for at least 260 weeks on change in episodic memory function as measured in the FCSRT Cueing Index in preclinical *PSEN1* E280A autosomal-dominant mutation carriers	• Annualized rate of change in the API ADAD Composite Cognitive Test total score, which is computed from the following 5 cognitive test scores:<br>– CERAD Word List Recall (Rosen et al. 1984; Morris et al. 1989; Mohs et al. 1997)<br>– Multilingual Naming Test (Gollan et al. 2012)<br>– MMSE for orientation to time (Folstein et al. 1975)<br>– CERAD Constructional Praxis (a measure of visuospatial ability; (Morris et al. 1989)<br>– Raven's Progressive Matrices, Set A (a measure of nonverbal fluid reasoning and visuospatial abilities; Raven 1976)<br>• Annualized rate of change on the FCSRT Cueing Index
**Secondary Objectives**	**Corresponding Endpoints**
• Assess the effect of crenezumab on clinical progression<br>• Assess the effect of crenezumab on overall cerebral fibrillar amyloid burden using predefined ROI from florbetapir PET	• Time to progression from preclinical AD to MCI due to AD (Albert et al. 2011) or from preclinical AD to dementia due to AD (McKhann et al. 2011)<br>• Time to progression to non-zero in the CDR Scale global score (Morris 1993)

Table 1 Objectives and Endpoints (cont.)

Secondary Objectives	Corresponding Endpoints
• Assess the effect of crenezumab on regional CMRgl using FDG-PET measurements in predefined ROIs<br>• Assess the effect of crenezumab on brain atrophy as measured by volumetric measurements using MRI<br>• Assess the effect of crenezumab on tau-based CSF biomarkers	• Annualized rate of change in the CDR Scale-Sum of Boxes (Morris 1993)<br>• Annualized rate of change in a measure of overall neurocognitive functioning: RBANS (Randolph 1998)<br>• Annualized rate of change in mean *cerebral* fibrillar amyloid accumulation using florbetapir PET *from a predefined ROI*<br>• Annualized rate of change in *regional* CMRgl using FDG-PET *in a predefined ROI* (Chen et al. 2010; Van Dyck et al. 2019)<br>• Annualized rate of change in *volumetric measurements* using MRI<br>• Annualized rate of change in CSF tTau and pTau
**Safety Objective**	**Corresponding Endpoints**
• To assess the safety and tolerability of crenezumab in preclinical *PSEN1* E280A mutation carriers (comparing crenezumab with placebo)	• Frequency and severity of treatment-emergent adverse events and serious adverse events<br>• Withdrawals of study drug due to adverse events<br>• Incidence of treatment-emergent adverse events of special interest, including:<br>– ARIA-E<br>– ARIA-H<br>– Cerebral macrohemorrhages<br>– Pneumonia<br>• Incidence of injection reactions and IRRs<br>• Incidence of treatment-emergent anti-crenezumab antibodies
**Pharmacokinetic/Pharmacodynamic Objective**	**Corresponding Endpoints**
• Collect sparse PK samples to support confirmation of exposure to crenezumab and to explore the PD response (as measured by plasma total Aβ levels comparing crenezumab with placebo) in preclinical *PSEN1* E280A mutation carriers	• CSF and serum crenezumab concentrations at protocol-specified timepoints<br>• Trough serum crenezumab C_{trough}, ss in serum<br>• Plasma and CSF Aβ1-40 and Aβ1-42 concentrations

Table 1 Objectives and Endpoints (cont.)

Exploratory Objectives	Corresponding Endpoints
• Assess further the effect of crenezumab in preclinical *PSEN1* E280A mutation carriers on additional clinical measures of efficacy and biological markers of disease that have not been prespecified as primary and secondary endpoints in the SAP<br>• Explore pharmacogenetic effects, including but not limited to, a person's APOE ε4 carrier status on the active treatment's cognitive, clinical, and adverse effects<br>• Explore effects of genetic variation, including but not limited to, how genes affect the biology of AD and related disorders and how genes influence biomarker responses<br>• Examine clinical and biomarker changes in *PSEN1* E280A non-carriers and to compare these changes with those seen in carriers treated with placebo<br>• Relate the treatment's biomarker effects to clinical outcomes and to examine predictive and prognostic utility of baseline characteristics<br>• Assess the impact of treatment on brain tau load over time, as measured by tau PET imaging in an optional substudy (GN28352-1/BN40199)	• Changes from baseline over time in the following cognitive measures:<br>– Trail Making Test (Armitage 1946)<br>– MMSE (Folstein et al. 1975)<br>– RBANS Index Scores (Randolph 1998)<br>– Scores of each of the components of the API ADAD Composite Cognitive Test Battery<br>– PACC (Donohue et al. 2014) modified for the API ADAD trial<br>– Clinical endpoints not examined in secondary outcome measures<br>• Changes in the NPI (Cummings et al. 1994; 1997) total score, items, and factors<br>• Changes in the GDS (Sheikh and Yesavage 1986) total score<br>• FAST (Sclan and Reisberg 1992) total score<br>• Changes in Subjective Memory Checklist (Acosta-Baena et al. 2011)<br>• Changes in other blood and CSF measures such as NfL and plasma tau markers<br>• Analysis of *image* ROIs not selected in secondary outcome measures<br>• Other imaging outcome measures and analytic methods, alone, or in conjunction with other imaging modalities not explored in secondary outcome measures<br>• Change in tau burden over time, as measured by GTP1 PET in an optional substudy (GN28352-1/ BN40199)<br>• Changes in primary, secondary, and exploratory outcomes in mutation non-carriers treated with placebo<br>• Comparisons of clinical and biomarker outcomes between carriers treated with placebo and non-carriers<br>• Changes in primary, secondary, and exploratory outcomes in carriers and non-carriers as functions of APOE genotype and other genetic variations

Table 1 Objectives and Endpoints (cont.)

Exploratory Objectives	Corresponding Endpoints
	• Short-term changes in imaging measures as functions of initiation (e.g., baseline to 12 weeks)<br>• Analyses of outcome measures in relation to one another and in relation to baseline characteristics

AD = Alzheimer's disease; API = Alzheimer's Prevention Initiative; APOE = apolipoprotein E; ADAD = autosomal-dominant Alzheimer's disease; ARIA-E = amyloid-related imaging abnormalities–edema/effusion; ARIA-H = amyloid-related imaging abnormalities–hemosiderin deposition; CDR = Clinical Dementia Rating; CERAD = Consortium to Establish a Registry for Alzheimer's disease; CMRgl = cerebral metabolic rate of glucose; CSF = cerebrospinal fluid; C_{trough}, ss = concentration at steady state; FAST = Functional Assessment Staging of Alzheimer's disease; FCSRT = Free and Cued Selective Reminding Task; FDG = fluorodeoxyglucose; GDS = Geriatric Depression Scale; GTP1 = Genentech Tau Probe 1; IRR = infusion-related reaction; MCI = mild cognitive impairment; MMSE = Mini-Mental State Examination; MRI = magnetic resonance imaging; NfL = neurofilament light chain; NPI = Neuropsychiatric Inventory; PACC = Preclinical Alzheimer's Cognitive Composite; PET = positron emission tomography; PD = pharmacodynamic; PK = pharmacokinetic; RBANS = Repeatable Battery for the Assessment of Neuropsychological Status; ROI = region of interest; SAP = Statistical Analysis Plan.

1.2 STUDY DESIGN

1.2.1 Study Period A

This is a prospective, randomized, double-blind, placebo-controlled, parallel-group study of crenezumab versus placebo in individuals who carry the *PSEN1* E280A autosomal-dominant mutation causing early-onset Alzheimer's disease (EOAD), and do not meet criteria for mild cognitive impairment (MCI) due to Alzheimer's disease (AD) (Albert et al. 2011) or dementia due to AD (McKhann et al. 2011) and are, thus, in a preclinical phase of AD (Sperling et al. 2011). The study also incorporates administration of placebo to individuals who are not *PSEN1* E280A autosomal-dominant mutation carriers. This efficacy and safety study is being conducted at a single primary site and 3 satellite sites in Colombia.

In the Study GN28352, *PSEN1* E280A autosomal-dominant mutation carriers who met study eligibility criteria were randomized in a 1:1 ratio to one of two treatment groups: crenezumab or placebo. Crenezumab is being administered either SC (720 mg every 2 weeks) or IV (60 mg/kg every 4 weeks). Matching placebo is administered by the same routes at the same dosing regimen. The switch to the higher IV dose (approximately 4–fold higher exposure to crenezumab) was optional. Participants may decide whether to change from the SC dosing route to the IV dose route; however, once IV dosing in a given participant has occurred, it is not intended for a participant to switch back to the lower SC dose. In order to maintain genotype blind

and to have a genetic kindred control, a cohort of *PSEN1* E280A mutation non-carrier kindred family members were also enrolled in the study and dosed only with placebo.

The study includes three arms *and originally aimed to have* approximately 100 participants per arm; two arms with participants that have a *PSEN1* E280A mutation randomized in a 1:1 ratio to active treatment or placebo and a third arm of non-carriers randomized to placebo (see Figure 1). Participants will receive the randomized treatment until the last participants have reached their last treatment visits at 260 weeks (see Figure 2).

The study duration for individual participants will be at least approximately 284 weeks, including an 8-week screening period, a double-blind treatment period of at least 260 weeks in length, a 4-week final dose visit, and a final safety follow-up visit 16 weeks after the last dose of study drug (crenezumab or placebo) that will allow for clinical follow-up after treatment discontinuation for participants who do not continue with study drug beyond the end of Study Period A or who terminate study drug early. Per protocol, all participants are assessed with clinical cognitive measures, magnetic resonance imaging (MRI), fluorodeoxyglucose (FDG) positron emission tomography (PET), amyloid PET, and blood-based biomarkers per the schedule of activities. Cerebrospinal fluid (CSF) collection is optional and tau PET is collected in an optional substudy.

1.2.2 Study Period B

Following the completion of Study Period A, participants will be offered the opportunity to continue to receive study drug until the results of the study are known. This is termed Study Period B. All *PSEN1* mutation carriers will be provided crenezumab in Study Period B regardless of treatment allocation during Study Period A, and all non-carriers will continue on placebo. Treatment allocation for both Study Periods A and B will remain blinded. Study Period B may last up to approximately 12 months depending on when participants enter Study Period B. See Figure 2.

After the primary efficacy results from Study Period A are available, the Sponsor may extend Study Period B, or initiate an OLE study for PSEN1 E280A autosomal-dominant mutation carriers, or development of crenezumab may be discontinued.

Figure 1 Study Design

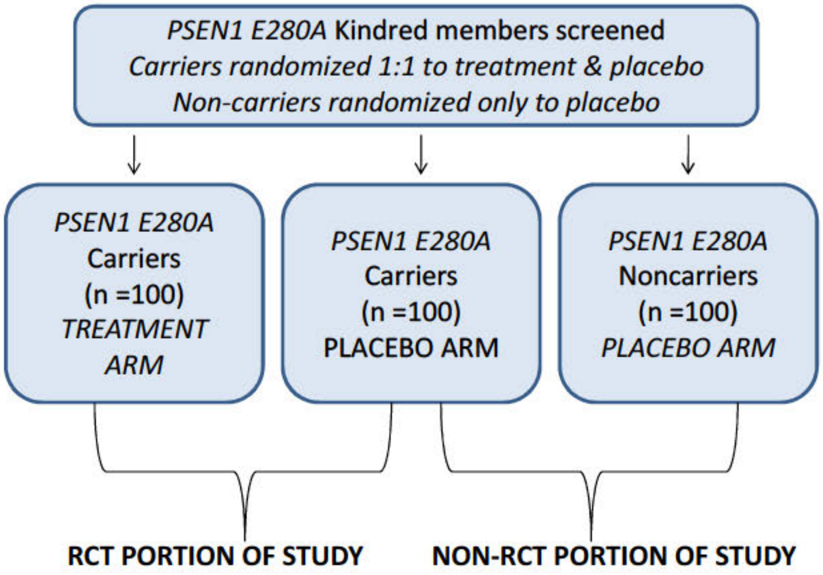

RCT = randomized, controlled trial.

Figure 2 Study Schematic

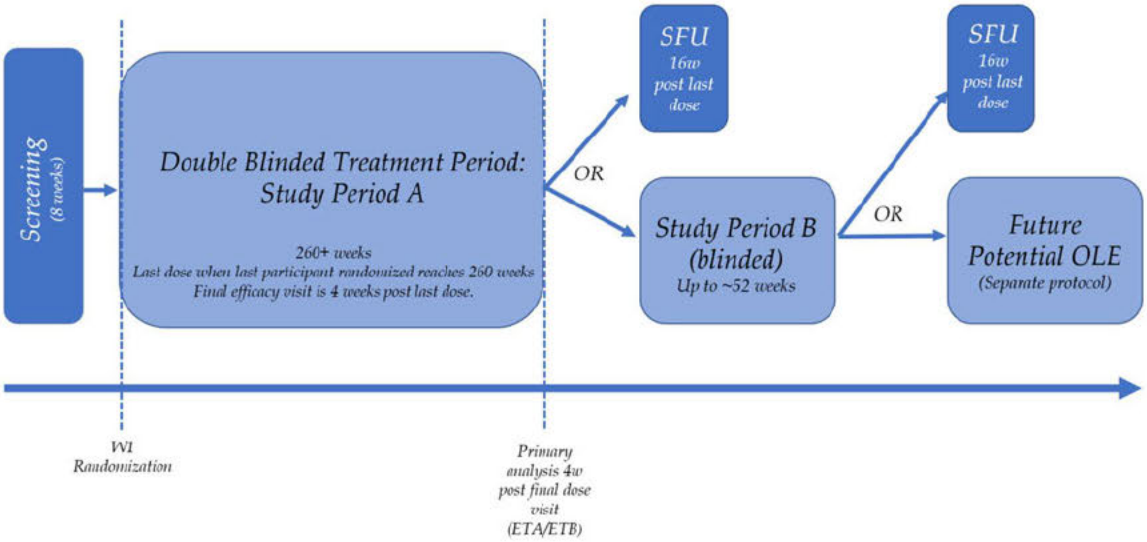

ETA/ETB = end of treatment visit A or end of treatment visit B; OLE = open-label extension; SFU = safety follow-up; W = week.

1.2.3 Treatment Assignment and Blinding

Both *PSEN1* E280A mutation carriers and non-carriers were enrolled. The majority of participants in the *PSEN1* E280A kindred cohort from Colombia do not wish to know their *PSEN1* genotype. In most cases, the participant will not know whether he or she is a mutation carrier or a non-carrier. Participants who had their mutation carrier status

disclosed by a physician in conjunction with genetic counseling outside of the auspices of this study and are mutation carriers were eligible for the study. Genetic disclosure performed independent of this study was not required for a mutation carrier to be eligible to participate. Non-carriers who received information about their genetic status prior to the study were not included.

Participants, blinded study personnel, and the Sponsor will not know if a participant has been assigned to the active treatment or one of the two placebo treatment groups in Study Period A. In Study Period B, participants and blinded study personnel will continue to be blinded to original and current treatment assignment. Following *the data snapshot* for *the* Study Period A *primary analysis*, *s*elected members of the Sponsor team may become unblinded for the purpose of analysis and interpretation of Study Period A data.

In Study Period A, a dynamic randomization design was used with treatment allocation assigned by the interactive voice or Web-based response system (IxRS) vendor. Mutation carriers were randomized to crenezumab and placebo arms in a 1:1 ratio. A smaller group of mutation non-carriers were assigned to placebo to conduct the study, such that autosomal-dominant AD (ADAD) family members were not required to receive information about their genetic risk and to help distinguish those changes related to the predisposition to AD from those associated with normal aging in the two placebo groups (in Study Period B, all carrier participants will be assigned crenezumab by the IxRS vendor and non-carrier participants will be assigned placebo).

While pharmacokinetic (PK) samples should be collected from participants assigned to the placebo arms to maintain the blinding of treatment assignment, PK assay results for these participants are generally not needed for the safe conduct or proper interpretation of this trial. A defined set of personnel responsible for performing PK assays and not otherwise involved in the conduct of the trial will be unblinded to participants' treatment assignments to identify appropriate PK samples to be analyzed. Samples from participants assigned to the placebo arms will not be analyzed except by request (i.e., to evaluate a possible error in dosing). In addition, PK and plasma pharmacodynamic (PD) assay results will not be released to blinded personnel until the study is unblinded.

If unblinding is necessary for participant management (e.g., in the case of a serious adverse event for which participant management might be affected by knowledge of treatment assignment), the investigator will be able to break the treatment code by contacting the IxRS. Treatment codes should not be broken except in emergency situations.

For regulatory reporting purposes, and if required by local health authorities, the Sponsor will break the treatment code for all serious, unexpected, and suspected

adverse reactions that are considered by the investigator or Sponsor to be related to study drug.

1.2.4 Independent Progression Adjudication Committee

Ascertainment of AD dementia or MCI progression will be made by the investigator. An independent progression adjudication committee (iPAC) was formed to review the information provided by the site investigators regarding participants enrolled in the trial. The iPAC will review data on:

- Participants who, in the opinion of the site investigator, appear to have progressed from not meeting criteria for MCI due to AD or dementia due to AD to meeting criteria for MCI due to AD or *to* dementia due to AD;
- Participants who, in the opinion of the investigator, appear to have progressed from meeting criteria for MCI due to other cause to meeting criteria for MCI due to AD or to dementia due to AD;
- Participants who, in the opinion of the investigator, appear to have progressed from meeting criteria for dementia due to other cause to meeting criteria for MCI due to AD or to dementia due to AD, and;
- A matching number of participants who, in the opinion of the investigator, do not appear to have progressed. *For each "case" the site submits, the data-coordinating center randomly selects a participant from the list of participants who have not yet been adjudicated as a progression using a random number generator. Once the participant has been identified, the data-coordinating center instructs the site to provide information needed for adjudication for that participant.*

The primary roles of iPAC members will be to:

- Provide guidance to the site and Sponsor regarding evaluation and as needed, collection of information necessary to adjudicate progression to MCI or dementia
- Review all participants' clinical information supplied in the supporting documents and make a determination as to whether the participant has progressed or not progressed from "normal cognition" (i.e., in this protocol, not meeting criteria for MCI or dementia) to MCI due to AD or dementia due to AD at the time of the most recent major clinical assessment at the site

If there is lack of consensus between the iPAC members as to progression of an individual participant, they will confer and arrive at a consensus. If the members' opinion differs with that of site investigators, one or both members will correspond with site investigators and attempt to arrive at a consensus. Failing that, the opinion of the iPAC will be used for purposes of statistical analysis; *the opinion* of the site investigators will be used for clinical management of the study participant. If after adjudication, both the iPAC members and site agree that the participant has not progressed, the participant will be re-adjudicated if deemed to progress by the site at a future visit.

1.2.5 Data Monitoring

An independent Data Monitoring Committee (iDMC) is being used during the conduct of this trial to periodically review unblinded safety and efficacy data. In addition to periodic review of safety and efficacy data, the iDMC may make recommendations regarding study conduct. Details of the iDMC membership and roles are detailed in the iDMC Charter.

2. STATISTICAL HYPOTHESES

The primary endpoint family consists of 2 endpoints: 1) annualized change on the Alzheimer's Prevention Initiative (API) ADAD Composite Cognitive Test total score and 2) annualized change on Free and Cued Selective Reminding Task (FCSRT) Cueing Index. The type I error is split between testing the treatment effect on the API ADAD Composite Cognitive Test total score ($\alpha = 0.04$) and on the FCSRT Cueing Index ($\alpha = 0.01$). If at least one of the 2 primary analyses is statistically significant, then the trial is considered positive. The primary endpoint family in the crenezumab arm and placebo arm will be compared among mutation carriers and tested using a random coefficient regression model (RCRM) with the null and alternative hypotheses as follows.

- API ADAD Composite Cognitive Test total score:
  - H_0: There is no difference in annualized change on the API ADAD Composite Cognitive Test total score between the crenezumab and placebo arms among mutation carriers
  - H_1: There is a difference in annualized change on the API ADAD Composite Cognitive Test total score between the crenezumab and placebo arms among mutation carriers
- FCSRT Cueing Index:
  - H_0: There is no difference in annualized change on FCSRT Cueing Index between the crenezumab and placebo arms among mutation carriers.
  - H_1: There is a difference in annualized change on FCSRT Cueing Index between the crenezumab and placebo arms among mutation carriers.

Details on the type I error control are described in Section 3.1.

3. SAMPLE SIZE DETERMINATION

This study will enroll up to 300 participants: up to 200 participants will be enrolled in the carrier cohort and up to 100 participants will be enrolled in the non-carrier cohort. Participants in the carrier cohort will be randomized in a 1:1 randomization ratio to active treatment or placebo. The study was initially powered to compare the mean change from baseline over 260 weeks in the API ADAD Composite Cognitive Test total score between the active treatment group and the placebo group. Assuming a 25% dropout rate with use of two-sided testing at the overall 0.05 level, a placebo group coefficient of variation of 65% for the Week 260 change scores ($= 100\% \times$ standard deviation of

placebo participant change scores/mean of placebo participant change scores) and 100 participants per arm, the study will have at least 80% power to detect a true effect of 30% reduction of the mean decline in the API ADAD Composite Cognitive Test total score in the placebo group using a t-test. Participants in the non-carrier cohort will all receive placebo and will be included in the study in order to maintain the genotype blind.

3.1 TYPE I ERROR CONTROL

A graph-based (Bretz et al. 2009; Bretz et al. 2011; U.S. Food and Drug Administration [FDA 2017]) testing procedure will be used to ensure the family-wise type I error will be controlled at a two-sided $\alpha = 0.05$. In Figure 3, we illustrate how hypothesis testing of the primary endpoint family will be performed. The key secondary endpoints will be tested hierarchically at $\alpha = 0.05$ ordered by the following, only if both primary endpoints are significant; after the first occurrence of the p-value > 0.05 in the list below, subsequent hypothesis testing will stop.

1. Annualized rate of change in mean fibrillar amyloid accumulation in a composite region (including frontal, temporal, parietal, and cingulate cortices) with a subcortical white matter reference region using florbetapir PET
2. Time to progression from preclinical AD to MCI due to AD or from preclinical AD to dementia due to AD
3. Annualized rate of change in the Clinical Dementia Rating (CDR) Scale-Sum of Boxes
4. Time to progression to non-zero in CDR global score
5. Annualized rate of change in a measure of overall neurocognitive functioning: Repeatable Battery for the Assessment of Neuropsychological Status (RBANS) total score

Figure 3 Graph-Based Testing Procedure for Type I Error Control

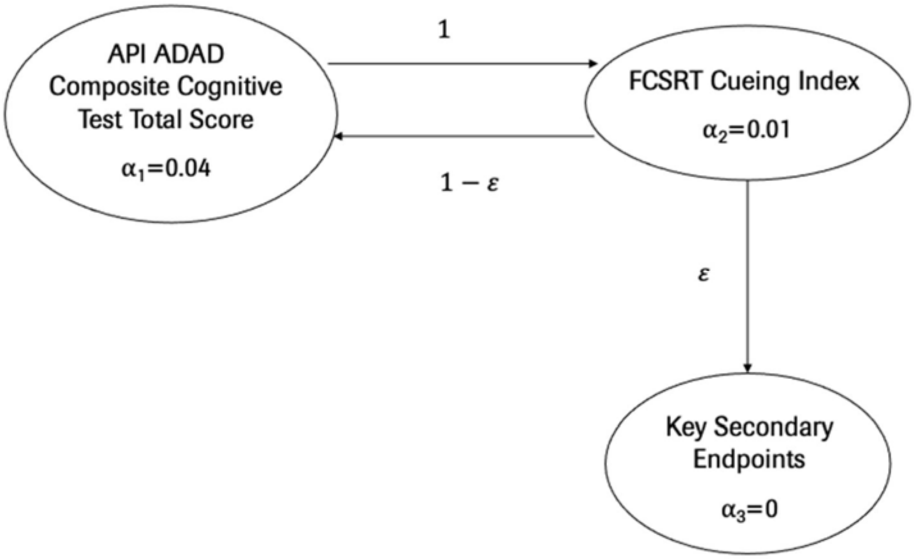

API = Alzheimer's Prevention Initiative; ADAD = autosomal-dominant Alzheimer's disease; FCSRT = Free and Cued Selective Reminding Task.

The arrows in Figure 3 denote the direction of α propagation; the notation ε indicates a positive real number close to zero, which in our case indicates the potential to pass α from the FCSRT Cueing Index to key secondary endpoints but only if it is not necessary to pass α from the FCSRT Cueing Index to the API ADAD Composite Cognitive Test total score. In the following, we illustrate how the procedure works in a few examples.

- **Example 1**: p-value from testing API ADAD Composite Cognitive Test total score hypothesis = 0.045; p-value from testing FCSRT Cueing Index hypothesis = 0.005. Our graph-based approach will declare statistical significance on both primary endpoints and α = 0.05 is passed to key secondary endpoints being tested in our specified order.
- **Example 2**: p-value from testing API ADAD Composite Cognitive Test total score hypothesis = 0.06; p-value from testing FCSRT Cueing Index hypothesis = 0.005. Our graph-based approach will declare statistical significance only on the FCSRT Cueing Index and no α is passed to key secondary endpoints.
- **Example 3:** p-value from testing API ADAD Composite Cognitive Test total score hypothesis = 0.03; p-value from testing FCSRT Cueing Index hypothesis = 0.03. Our graph-based approach will declare statistical significance on both primary endpoints and α = 0.05 is passed to key secondary endpoints being tested in our specified order.
- **Example 4:** p-value from testing API ADAD Composite Cognitive Test total score hypothesis = 0.03; p-value from testing FCSRT Cueing Index hypothesis = 0.06. Our graph-based approach will declare statistical significance only on the API ADAD

Composite Cognitive Test total score and no α is passed to key secondary endpoints.

Before study unblinding, the Sponsor performed the blinded power (BP) analysis on the original primary endpoint (API ADAD Composite Cognitive Test total score) and the secondary clinical endpoints using data from all participants regardless of carrier status. The BP analyses assumed the same crenezumab treatment effect across all endpoints considered. The Sponsor concluded that the FCSRT Cueing Index was expected to have a CV which meets the statistical power target specified in the protocol without speculation on the treatment effect based on the blinded data. Therefore, the Sponsor decided to add the FCSRT Cueing Index to the primary endpoint family due to its expected sensitivity to change over time given its clinical relevance as a sensitive measure of episodic memory decline, a hallmark of emerging symptomatic AD, and the most commonly observed, early, and consistent neuropsychological marker of AD (Tounsi et al. 1999; Bateman et al. 2012; Caselli et al. 2020). Appendix 2 describes details of the BP analysis along with *mathematical proof* of type I error control.

4. ANALYSIS SETS

The following analysis populations are defined for Study Period A:

Population	Definition
ITT	All randomized participants, whether or not the participant received the assigned treatment; participants are grouped according to the treatment assignment at randomization × mutation status.
mITT	All randomized participants who received at least 1 dose of study drug; participants are grouped according to the treatment assignment at randomization; all primary and secondary efficacy analyses are based on the mITT population among the mutation carriers.
Safety-evaluable	All randomized participants who received at least 1 dose of study drug; participants are grouped according to the actual treatment received × mutation status; mutation carriers randomized to placebo and mutation non-carriers randomized to placebo who received more than 2 doses of crenezumab will be grouped in the crenezumab arm; all participants who have received no more than 2 doses of crenezumab will be further grouped together in a placebo arm, regardless of the mutation status.
Pharmacokinetic-evaluable	All safety-evaluable participants with at least 1 plasma sample, provided sufficient dosing information (dose and dosing time) is available. Participants will be grouped according to the actual treatment received; participants randomized to placebo who received more than 2 doses of crenezumab will be grouped in the crenezumab arm.

Population	Definition
Tau-ITT	Tau substudy ITT population. All participants randomized in Study GN28352 and consented to the tau PET substudy, whether or not the participant received the assigned treatment; participants are grouped according to the treatment assignment at randomization × mutation status.
Tau-mITT	Tau substudy modified ITT population. All participants randomized in Study GN28352 and consented to the tau PET substudy who received at least 1 dose of study drug; participants are grouped according to the treatment assignment at randomization; analyses on change in tau burden are based on Tau-mITT population among the mutation carriers.
Tau-safety-evaluable	All participants randomized in Study GN28352 and consented to the tau PET substudy who received at least 1 dose of study drug; participants are grouped according to the actual treatment received × mutation status; mutation carriers randomized to placebo and mutation non-carriers randomized to placebo who received more than 2 doses of crenezumab will be grouped in the crenezumab arm; all participants who have received no more than 2 doses of crenezumab will be further grouped together in a placebo arm, regardless of the mutation status.

ITT = intent-to-treat; mITT = modified intent-to-treat; PET = positron emission tomography.

5. STATISTICAL ANALYSES

5.1 GENERAL CONSIDERATIONS

Analyses for Study Period A includes the following:

- A primary analysis *(initial readout)* will occur after all participants have completed the Week 260 assessments
- *During Study Period B, safety analyses from Study Period A will be refreshed (due to the additional 16-week safety follow-up data accrued in Period A after the primary analysis) and reported in the final Clinical Study Report (CSR); no other analyses (e.g., primary and secondary endpoints and PK/PD endpoints) will be refreshed*

The analyses for Study Period B or the OLE study are described in Section 5.10.

All efficacy analyses will be performed on the modified intent-to-treat (mITT) population, unless otherwise specified. Participants will be analyzed according to the treatment assigned at randomization by IxRS.

All safety analyses will be performed in the safety-evaluable population, unless otherwise specified.

All data, including data collected after Week 260, will be used for the primary analysis.

5.2 PARTICIPANT DISPOSITION

The analysis of participant disposition will be based on the intent-to-treat (ITT) population. Reasons for early discontinuation of treatment or early termination from the study will be listed and summarized by treatment group X mutation status.

5.3 PRIMARY ENDPOINTS ANALYSIS

The primary endpoint analysis from Study Period A will be based on the mITT population. The analysis will be adjusted by the factors used in randomization: age, education, apolipoprotein E (APOE) 4 carrier status (carrier vs. non-carrier), and baseline CDR zero versus CDR non-zero. The stratification factors as recorded in IxRS (see protocol for details) will be used.

5.3.1 Definition of Primary Endpoints

The primary endpoint family is 1) the annualized rate of change in the API ADAD Composite Cognitive Test total score and 2) the annualized rate of change in the FCSRT Cueing Index.

The API ADAD Composite Cognitive Test total score is computed from the following 5 cognitive test scores:

- Consortium to Establish a Registry for AD (CERAD) Word List: Recall (Rosen et al. 1984; Morris et al. 1989; Mohs et al. 1997)
- Multilingual Naming Test (Gollan et al. 2012)
- Mini-Mental State Examination (MMSE) for orientation to time (Folstein et al. 1975)
- CERAD Constructional Praxis (a measure of visuospatial ability; Morris et al. 1989)
- Raven's Progressive Matrices (a measure of nonverbal fluid reasoning and visuospatial abilities) Set A (Raven 1976)

Specifically, the ADAD-API Cognitive Composite Test total score is calculated as [(Multilingual Naming Test Score/15) + (MMSE Score/5) + (Raven's Progressive Matrices Score/12) + (CERAD Word List Recall Score/10) + (CERAD Constructional Praxis Score/11)] × 20.

If at least one of the primary analyses is statistically significant, then the trial will be deemed positive. Type I error will be controlled using a graph-based procedure (FDA 2017; see Section 3.1).

5.3.2 Estimands

Primary endpoint estimands are defined on the basis of the following attributes.

- Population: *PSEN1* E280A mutation carriers as defined by the inclusion/exclusion criteria specified in the protocol (mITT)
- Treatments: crenezumab versus placebo either by SC administration or by IV administration

- Variables: 1) annualized rate of change in the API ADAD Composite Cognitive Test total score and 2) annualized rate of change in the FCSRT Cueing Index
- Intercurrent events:
  - Events that lead to treatment withdrawal due to an adverse event. A treatment strategy will be applied where all observed values will be used regardless of the occurrence of the intercurrent event.
  - Death not due to AD. A hypothetical strategy will be applied where all values will be censored after the occurrence of the intercurrent event.
  - Death due to AD. All values on the primary endpoint after this event will be imputed as 0, the worst score possible
  - Pregnancy that leads to treatment withdrawal. A treatment strategy will be applied where all observed values will be used regardless of the occurrence of the intercurrent event.
  - Resumption of dosing after treatment discontinuation post-protocol amendment, version 8. A treatment policy strategy will be applied where all observed values will be used regardless of the occurrence of the intercurrent event.
  - Dose change from SC to IV administration. A treatment policy strategy will be applied where all observed values will be used regardless of the occurrence of the intercurrent event.
  - Events that lead to treatment interruption or withdrawal due to coronavirus disease 2019 (COVID-19) diagnosis. A hypothetical strategy will be applied where all primary efficacy data will be censored between COVID-19 diagnosis and recovery (if recovery date is missing, then all primary efficacy data after COVID-19 diagnosis date will be censored).
  - *Concurrent use of protocol-defined prohibited medications that can potentially impact cognition (e.g., anti-psychotics, anticonvulsants, benzodiazepines, etc.). A treatment policy strategy will be applied where all observed values will be used regardless of the occurrence of the intercurrent event.*
- Population-level summary: difference in annualized rate of change between the mutation carrier crenezumab arm and mutation carrier placebo arm

5.3.3 Main Analytical Approach for Primary Endpoint Family

The primary analysis on both primary endpoints will be performed using a RCRM (Richeldi et al. 2014; Flaherty et al. 2019; Hu et al. 2021) with a missing at random (MAR) assumption. This model will include all observations, including baseline scores as the response variable. The RCRM will include the randomization stratification factors described in Section 5.3 as fixed effects with an intercept, and will include the categorical treatment groups as interaction terms with time*; stratification factor(s) may be removed from the model if there is risk of non-convergence or sparse strata (in case*

of non-convergence, stratification factors will be removed in the order of the smallest number of observations in any cell caused by that stratification factor).
Participant-level random intercept and random slope are also included in the model, where an unstructured covariance between the random intercept and random slope is assumed. The RCRM is constructed as follows:

$$Y_{ij} = \alpha + (\text{Randomization Factors})_i + u_{0i} + (\beta + \beta_x I_{x,i} + u_{1i}) t_{ij} + \varepsilon_{ij}$$

where Y_{ij} is the score for participant i at visit j; t_{ij} is the duration in the unit of year after first drug intake for participant i at visit j; $I_{x,i}=1$ if participant i in a *PSEN1* E280A mutation carrier is in the crenezumab mITT group, and $I_{x,i}=0$ if participant i is a *PSEN1* E280A mutation carrier is in the placebo mITT group; u_{0i} and u_{1i} are random intercept and random slope, respectively, for the ith participant; ε_{ij} is the random error for the ith participant at visit j. The crenezumab treatment effect on the rate of change is quantified by β_x; the 95% CI and p-value for crenezumab treatment effect will be presented. The crenezumab treatment effect compared with the placebo group on the relative reduction (RR) scale is calculated as $-\beta_x/\beta \times 100\%$; its estimate will also be presented; its 95% CI using bootstrap approach will also be provided.

5.3.4 Sensitivity Analyses for Primary Endpoints

The following sensitivity analyses for the primary endpoints are planned.

- To relax the assumption of the linearity in disease trajectory, a mixed model with repeated measures (MMRM) will be used. The response variable is change from baseline. The effects in the model will include baseline score, randomization factors, treatment group, visit, baseline score-by-visit interaction, and treatment-by-visit interaction. Visits will be treated as the repeated variable within a participant. Participant, treatment, and visit will be treated as class variables. An unstructured variance-covariance structure will be applied to model the within-participant errors; in the case of non-convergence, compound symmetry will be used. If non-convergence still exists, data from Year 8 (longest duration in the trial based on enrollment) will be removed, likely caused by a small number of datapoints at Year 8 due to the common-close study design. If non-convergence still exists, visit data will be removed in reverse chronological order until a convergence (either by unstructured or compound symmetry assumption, with unstructured always being tested first) is reached.

  For instances where the score is assessed outside of the visit window described in the protocol, it will be mapped to the closest scheduled visit as described in the schedule of activities in Appendices A–1 through A–4 of the protocol. Baseline score is defined as the last non-missing score on or before the first study drug exposure. After the mapping, if multiple scores exist at a visit, the score closest to the scheduled visit date is chosen in the MMRM analysis.

  The difference between the crenezumab group and the placebo group in the least squares (LS) mean change from baseline will be estimated at each timepoint. The 95% CIs and p-values for LS mean treatment difference will be presented.

The MMRM LS mean plot with standard errors (SEs) will be made to visually examine the linearity of the disease trajectory.

- To quantitatively examine the impact of the linearity assumption and the assumption of the variance decomposition in the RCRM, the robust variance estimator (Huber 1967; White 1982) will be used in the RCRM to re-calculate the SEs and; therefore, CIs and p-values in the primary analysis model
- Analysis assuming participants who $discontinue$ from $crenezumab\ treatment$ will lose the treatment effect instantaneously.

  Immediately after a participant $discontinues\ in\ the\ first\ occurrence$ from the $crenezumab\ treatment$, the population-level annualized rate of change will be assumed to be the same as the population-level annualized rate of change in the placebo arm. This approach will likely yield a conservative estimate of the treatment effect. In particular, the model is written as:

$$Y_{ij} = \alpha + (\text{Randomization Factors})_i + u_{0i} + (\beta + u_{1i})t_{ij} + \left(\beta_x \left(t_{ij} I_{t_{ij} \leq t_{i,discon}} + t_{DROP} I_{t_{ij} > t_{i,discon}}\right)\right) I_{x,i} + \epsilon_{ij}$$

  where $t_{i,DROP}$ represents the time of $the\ first\ occurrence\ of\ discontinuation\ from\ the\ crenezumab\ treatment$ for participant i, and $I_{t_{ij} \leq t_{i,discon}}$ and $I_{t_{ij} > t_{i,discon}}$ are indicator variables similarly defined previously in this document. If a participant has not $discontinued\ from\ the\ crenezumab\ treatment$ during Study Period A, then $t_{i,discon}$ is defined as an arbitrarily large number (e.g., 100 with “year” as the unit).
  The 95% CIs and p-values for estimates in β_x will be presented.
- Analysis using control-based mean imputation
  The underlying assumption of this sensitivity analysis is that the missing value mean from the crenezumab arm is the same or worse than the estimated overall mean from the placebo arm. Consequently, this approach yields a conservative treatment effect estimate in crenezumab. The details of this approach can be found in Mehrotra et al. (2017). The model setup and more statistical details can be found in Appendix 3.
- Analysis using hypothetical strategy for all intercurrent events, except for the dosing regimen change. The intercurrent events and the handling strategy in this analysis is defined as follows:
  - Events that lead to treatment withdrawal due to an adverse event.
    A hypothetical strategy where all values will be censored after the first occurrence of the intercurrent event.
  - Death: A hypothetical strategy where all values will be censored after the occurrence of the intercurrent event.
  - Pregnancy that leads to treatment withdrawal. A hypothetical strategy will be applied where all values will be censored after the occurrence of the intercurrent event.

– Resumption of dosing after treatment discontinuation post-protocol amendment, v8. A hypothetical strategy where all values will be censored after 1 day before the first occurrence of the intercurrent event.

– Dose change from SC to IV administration. A treatment policy strategy will be applied where all observed values will be used regardless of the occurrence of the intercurrent event.

– Events that lead to treatment interruption or withdrawal due to COVID-19 diagnosis. A hypothetical strategy where all values will be censored after the first occurrence of the intercurrent event.

– *Concurrent use of protocol-defined prohibited medications that can potentially impact cognition (e.g., anti-psychotics, anticonvulsants, benzodiazepines, etc.). A hypothetical strategy will be applied where all values will be censored from the start date of the occurrence of the intercurrent event to 2 days after the occurrence of the intercurrent event.*

5.3.5 Supplementary Analyses for Primary Endpoints

The following supplementary analyses will be performed for the primary endpoints to provide further understanding of the treatment effect.

- Analysis investigating efficacy in SC crenezumab versus efficacy in IV crenezumab:

  In this analysis, the treatment effect will be assumed to be different between the 2 administration periods. This analysis intends to gain insight on the impact of dose on the treatment effect. Since the IV dose is approximately 4–fold higher than the SC dose, we can use the administration method as a proxy for the dose. In this analysis, the change in administration method is assumed to change the population-level outcome trajectory for participants in the placebo group.
  For participants in the crenezumab group, the population-level outcome trajectory will also change once an administration method occurs. Furthermore, the switch can only go from SC to IV. In particular, the statistical model is constructed as:

$$Y_{ij} = \alpha + (\text{Randomization Factors})_i + u_{0i} + (\beta + u_{1i})t_{ij} + \beta_p(t_{ij} - t_{i,s})\left(1 - I_{x,i}\right)t_{ij}I_{t_{ij}>t_{i,s}} + \left(\left(\beta_{SC,x}t_{ij}\right)I_{t_{ij}\le t_{i,s}} + \left(\beta_{IV,x}(t_{ij} - t_{i,s}) + \beta_{SC,x}t_{i,s}\right)I_{t_{ij}>t_{i,s}}\right)I_{x,i} + \epsilon_{ij}.$$

  In this model, $\boldsymbol{t_{i,s}}$ denotes the switching time for participant i after baseline; if a participant never switched in Study Period A of the study, then $\boldsymbol{t_{i,s}}$ is defined as an arbitrarily large number (e.g., 100 with "year" as the unit). The parameter $\boldsymbol{\beta_p}$ denotes the effect of the change in administration method for participants in the placebo group. The variables $\boldsymbol{I_{t_{ij}\le t_{i,s}}}$ and $\boldsymbol{I_{t_{ij}>t_{i,s}}}$ are indicator variables similarly defined as $\boldsymbol{I_{x,i}}$. The annualized treatment benefit of crenezumab by SC is quantified by $\boldsymbol{\beta_{SC,x}}$; the annualized treatment benefit of crenezumab by IV is quantified by $\boldsymbol{\beta_{IV,x}}$. For model simplicity, up to 1 switch is assumed. The 95% CIs and p-values for estimates in $\boldsymbol{\beta_{SC,x}}$ and $\boldsymbol{\beta_{IV,x}}$ will be presented. The point estimates for the SC and IV treatment effect in the RR scale will also be presented.

- Analysis on each component of the API ADAD Composite Cognitive Test total score

  To understand the treatment effect on each component of one of the primary endpoints (API ADAD Composite Cognitive Test total score), the same primary analysis model will be run on each component. The 95% CIs and p-values for the treatment effect estimator will be presented. A forest plot will be made.

- To explore whether decline accelerates or decelerates, a quadratic RCRM where leading t^2 terms (treatment and time^2 interaction terms and random quadratic term) are added to the primary analysis model. The covariance matrix of $\{u_{0i}, u_{1i}, u_{2i}\}$ is assumed to be unstructured and all random effect terms are assumed to be independent of the pure error term. Specifically, this model is written as:

$$Y_{ij} = \alpha + (\text{Randomization Factors})_i + u_{0i} + (\beta + \beta_x I_{x,i} + u_{1i})t_{ij} + (\theta + \theta_x I_{x,i} + u_{2i})t_{ij}^2 + \epsilon_{ij}.$$

All statistical inferences as in the primary analysis model along with the point estimates of θ_x and 95% CI will be presented.

5.3.5.1 Subgroup Analyses for Primary Endpoints

The following subgroups will be analyzed with respect to the primary endpoints using the same primary analysis. Forest plots will be presented to summarize the results.
The subgroup categories may be combined if there is not enough representation of a specific subpopulation or if the statistical model fails to converge.

- Age ($\leq$38 vs. $>$38)
- Education ($<$9 years vs. $\geq$9 years)
- APOE4 carrier status (carrier vs. non-carrier)
- Baseline CDR zero vs. CDR non-zero
- Amyloid status at baseline (amyloid positive vs. amyloid negative) using a standardized uptake value ratio (SUVr) threshold *(details of the threshold will be pre*specified *in the biomarker analysis plan)*

5.4 SECONDARY ENDPOINTS ANALYSES

The continuous secondary efficacy endpoints will be analyzed using the same primary statistical model as for the primary endpoints. All time-to-event endpoints will be analyzed with a stratified log-rank test using the primary analysis stratification factors. Within each time-to-event endpoint, the hazard ratio (HR) for recurrence will be estimated using a stratified Cox proportional hazards model, and the 95% CI for the HR will be provided. Stratification factor(s) may be removed from the stratified analyses if there is risk of non-convergence or sparse strata *(in the case of non-convergence, stratification factors will be removed in the order of the smallest number of observations in any cell caused by that stratification factor).*

5.4.1 Key/Confirmatory Secondary Endpoints

The key secondary endpoints are:

- *Annualized rate of change in mean cerebral fibrillar amyloid accumulation using florbetapir PET from a predefined ROI*
  - *SUVrs will be derived from florbetapir PET images using* a composite region *of interest* (including frontal, temporal, parietal, and cingulate cortices) with a subcortical white matter reference region
- Time to progression from preclinical AD to MCI due to AD or from preclinical AD to dementia due to AD
- Annualized rate of change in the CDR Scale-Sum of Boxes
- Time to progression to non-zero in CDR global score
- Annualized rate of change in a measure of overall neurocognitive functioning: RBANS total score

Time to progression is calculated from the time at baseline, which is the last record on or prior to the first baseline reference date; the baseline reference date is either the first exposure date or the randomization date if the first exposure date is missing.
A gatekeeping strategy will be used for testing hypotheses of the key secondary outcomes using the graph-based type I error approach described in Section 3.1, the key secondary outcomes will be tested only if both the primary endpoints are statistically significant. All the tests will be done based on a two-sided $\alpha = 0.05$. The tests in the key secondary outcomes will be done in sequential order specified in the following:

1. Annualized rate of change in mean fibrillar amyloid accumulation in a composite region (including frontal, temporal, parietal, and cingulate cortices) with a subcortical white matter reference region using florbetapir PET
2. Log-rank analysis of time to progression from preclinical AD to MCI due to AD or from preclinical AD to dementia due to AD
3. RCRM analysis of CDR Scale-Sum of Boxes
4. Log-rank analysis of time to progression to non-zero in CDR global score
5. RCRM analysis of RBANS total score

At initial study readout if the study is positive (at least one of the primary endpoints is statistically significant), the Sponsor will analyze all available data from the key secondary biomarker endpoint: annualized rate of change in mean cerebral fibrillar amyloid accumulation in a composite region (including frontal, temporal, parietal, and cingulate cortices) with a subcortical white matter reference region using florbetapir PET. If the study is negative (neither *of the* primary endpoints are statistically significant), the Sponsor will analyze at least the baseline and last-available (in time) data from the florbetapir PET endpoint at the initial study readout, which is at the *primary analysis.*

5.4.2 Supportive Secondary Endpoints

The supportive secondary biomarker endpoints include:

- *Annualized rate of change in regional cerebral metabolic rate of glucose (CMRgl) using FDG-PET in a predefined ROI*
  - Annualized rate of change in regional (CMRgl) *will be derived from FDG PET images using an empirically predefined statistical ROI (sROI) known to be preferentially affected by CMRgl decline in persons with AD (Chen et al. 2010; Van Dyck et al. 2019)*
- *Annualized rate of change in volumetric measurements using MRI*
  - *Regions will include* the whole brain, bilateral hippocampus, and *bilateral* ventricles
- Annualized rate of change in CSF tTau and pTau biomarkers

Similar to the approach from the key secondary biomarker efficacy endpoint, the Sponsor will analyze all available data on the FDG-PET and volumetric MRI data if the study is positive. Otherwise, the Sponsor will analyze at least the baseline and last-available (in time) data on FDG-PET and volumetric MRI at the initial study readout.

5.5 EXPLORATORY ENDPOINTS ANALYSIS

Exploratory outcome measures are listed below. The continuous endpoints will be analyzed using the RCRM from the primary analysis and/or using an MMRM; the correlation analysis among outcomes will be modeled using a Spearman correlation estimation. The "mutation non-carriers treated with placebo" in this section is defined as all randomized mutation non-carriers that received at least 1 dose of study drug.

5.5.1 Clinical

Changes from baseline over time in the following cognitive measures:

- Trail Making Test (Armitage 1946)
- MMSE (Folstein et al. 1975)
- RBANS Index Scores (Randolph 1998)
- Scores from each of the components of the API ADAD Composite Cognitive Test Battery
- Preclinical Alzheimer Cognitive Composite (PACC; Donohue et al. 2014) modified for the API ADAD trial; PACC is calculated as the standardized sum of MMSE, RBANS story recall, RBANS coding, and FCSRT total score (sum of 3 free and cued recall trials)
- Clinical endpoints not examined in secondary outcome measures
- Changes in the Neuropsychiatric Inventory (NPI) total score, items, and factors (Cummings et al. 1994; 1997)

- Changes in the Geriatric Depression Scale (GDS) total score (Sheikh and Yesavage 1986)
- Changes in Functional Assessment Staging of Alzheimer's Disease (FAST) total score (Sclan and Reisberg 1992)
- Changes in Subjective Memory Checklist (Acosta-Baena et al. 2011)

5.5.2 Fluid Biomarkers

- CSF levels of $A\beta_{1-42}$ and $A\beta_{1-40}$
- Changes in other blood and CSF measures such as neurofilament light (NfL) and plasma tau markers

5.5.3 Imaging Biomarkers

- Analysis of other *regional or global measures* not selected in secondary outcome measures. *See the biomarker analysis plan for more details.*
- Other imaging outcome measures and analytic methods, alone, or in conjunction with other imaging modalities not explored in secondary outcome measures
- Change in tau burden over time, as measured by *Genentech Tau Probe 1 (*GTP1*)* PET in an optional substudy (GN28352-1/BN40199). See Section 5.8 for analysis details.

5.5.4 Other Endpoints

See the biomarker analysis plan for more details on the following endpoints:

- Changes in primary, secondary, and exploratory outcomes in mutation non-carriers treated with placebo
- Comparisons of clinical and biomarker outcomes between carriers treated with placebo and non-carriers
- Changes in primary, secondary, and exploratory outcomes in carriers and non-carriers as functions of the APOE genotype and other genetic variations
- Short-term changes in imaging measures as functions of initiation (e.g., baseline to 12 weeks)
- Analyses of outcome measures in relation to one another and in relation to baseline characteristics

5.6 SAFETY ANALYSES

All safety analyses of Study Period A will be performed in the safety-evaluable population (see Section 4 for definition).

As the study participants are considered treatment-exposed during the 16-week (approximately 5 half-lives) follow-up period, adverse events reported within 16 weeks after the last dose of study drug administered in Study Period A will be included in the safety analyses from Study Period A (contrary to the description in the protocol stating they will be summarized separately). At the end of Study Period B, safety analyses on

Study Period A will be refreshed *(due to the additional 16-week safety follow-up data accrued in Period A after the primary analysis)* and reported in the final CSR.

Adverse events reported within 16 weeks after the last dose of study drug administered in Study Period B will be included in the safety analyses on Study Period B. Safety analyses of Study Period B and/or of the potential OLE data are summarized in Section 5.10.2.

Safety will be assessed through summaries of exposure to study treatment, adverse events, changes in laboratory test results, MRI findings, changes in vital signs and ECGs, and changes in suicidality assessment.

Study treatment exposure (such as treatment duration, total dose received, and dose modifications) will be summarized with descriptive statistics.

All verbatim adverse event terms will be mapped to MedDRA thesaurus terms, and adverse event severity will be graded according to National Cancer Institute Common Terminology Criteria for Adverse Events (NCI CTCAE), v4.0. All adverse events, serious adverse events, adverse events leading to death, adverse events of special interest, and adverse events leading to study treatment discontinuation that occur on or after the first dose of study treatment (i.e., treatment-emergent adverse events) will be summarized. All adverse events will be summarized by severity and by relationship to study drug. For events of varying severity, the highest grade will be used in the summaries.

Relevant laboratory, vital signs (pulse rate, respiratory rate, blood pressure, pulse oximetry, and temperature), and ECG data will be displayed by time, with grades identified where appropriate. Additionally, a shift table of selected laboratory tests will be used to summarize the baseline and maximum post-baseline severity grade. Changes in vital signs and ECGs will be summarized. Proportion of participants with suicidal ideation or behavior, as reported in the suicidality assessment, will be analyzed by treatment groups.

5.6.1 Extent of Exposure

Study treatment exposure (such as treatment duration, total dose received, and dose modifications) will be summarized with descriptive statistics.

Exposure to study drug (number of study drug administrations and duration of treatment), regardless of SC or IV administration, will be summarized by treatment group in the safety-evaluable population.

Exposure will also be summarized by the administration route (SC vs. IV).

If a participant did not resume treatment after a treatment discontinuation, then the duration of treatment is the time from first study drug to the earlier of:

- Date of treatment discontinuation or date of study treatment completion in Study Period A
- The analysis cutoff date

Otherwise, if a participant resumed treatment after a treatment discontinuation, then the duration is the summation of duration of each treatment period, where the duration of the first treatment period is defined as the time from first study drug to the date of first treatment discontinuation and the duration of the last treatment period is defined as the time from the last treatment resumption date to the earlier of:

- Date of treatment discontinuation or date of study treatment completion in Study Period A
- The analysis cutoff date

The duration of the rest of the treatment periods is defined as the time from the treatment resumption date to the date of treatment discontinuation.

See Section 5.7.4 for PK/PD analyses.

5.6.2 Adverse Events

All treatment-emergent adverse events, serious adverse events, adverse events leading to death, protocol-specified adverse events of special interest (including amyloid-related imaging abnormalities–edema/effusion [ARIA-E], amyloid-related imaging abnormalities–hemosiderin deposition [ARIA-H], cerebral macrohemorrhages, pneumonia*, potential drug-induced liver injury, and suspected transmission of infectious agent~~s~~ by the investigational product*) and adverse events leading to study treatment discontinuation will be summarized by MedDRA Preferred Term, appropriate thesaurus level by treatment arm. Adverse events will be summarized by severity and by relationship to study drug. For events of varying severity, the highest grade will be used in the summaries.

In addition, treatment-emergent injection reactions, infusion-related reactions and COVID-19 infections will be summarized by treatment group.

Only treatment-emergent adverse events will be included in the analyses, defined as any adverse event reported during or after the first dose of study drug. Adverse events with a missing onset date will be considered to be treatment-emergent. Adverse events with a partially missing onset date will also be included as treatment-emergent if the month (if it was recorded) and the year occur on or later than the month and year of the study treatment start date.

A separate listing of adverse events reported during the screening period (before the first dose of study drug) will also be produced.

5.6.3 Clinical Laboratory Data

Clinical laboratory data (serum chemistry, hematology evaluations, including CBC with differential and platelet counts, and urinalysis values) will be summarized over time by descriptive statistics by treatment group, with grades identified where appropriate. Additionally, a shift table of selected laboratory tests will be used to summarize the baseline and maximum post-baseline severity grade.

5.6.4 MRI Evaluations

Neuroradiologic evaluation with respect to the occurrence of cerebral vasogenic edema (ARIA-E); superficial siderosis of the CNS or cerebral microhemorrhages (ARIA-H); or of cerebral macrohemorrhages will be performed during the *Study Period A* treatment period and will be summarized by treatment group using descriptive statistics.

5.6.5 Vital Signs

Vital signs (pulse rate, blood pressure, body temperature, and respiratory rate) will be displayed over time and changes from baseline will be summarized by treatment group.

5.6.6 Electrocardiograms

Electrocardiogram data will be displayed over time and changes from baseline for relevant ECG intervals will be summarized by treatment group *up to Week 17*.

5.6.7 Suicidal Ideation and Behavior

The proportion of participants with suicidal ideation or behavior, as reported in the suicidality assessment, will be analyzed by treatment group.

5.6.8 Anti-Drug Antibodies

The number and percentage of participants with confirmed positive anti-drug antibodies (ADAs) will be reported for each treatment group at baseline (prevalence of ADAs in the crenezumab and placebo groups) and after treatment with crenezumab (treatment-emergent ADAs). *At the primary analysis, the Sponsor will have only analyzed partial ADA data. If the study is negative and results from the partial ADA data are consistent with the extensive ADA data collected in previous Phase I–III studies, no additional ADA analyses are planned. The rationale is that ADA data from approximately 1900 samples that have already been tested is sufficient to confirm the low immunogenicity risk of crenezumab in this population without impacting the interpretation of the overall study results. However, if the study is positive, (at least one of the primary endpoints is statistically significant) the Sponsor will then analyze the complete ADA dataset in the final analysis and report it.*

5.7 OTHER ANALYSES

5.7.1 Summaries of Conduct of Study

In Study Period A, the number of participants who enroll, discontinue from treatment, discontinue from study, and complete the study will be tabulated by treatment group. Reasons for early discontinuation of treatment or early termination from the study will be listed and summarized by treatment group. Any eligibility criteria and other major protocol deviations will also be summarized by treatment group. The summaries of conduct of study will be based on the ITT population; randomized non-carriers will also be grouped and presented in this section.

5.7.2 Summaries of Treatment Group Comparability/Demographics and Baseline Characteristics

Demographic and baseline characteristics such as age, sex, race, APOE4 status, and baseline cognitive scores will be summarized for the ITT population by treatment group using descriptive statistics; randomized non-carriers will also be grouped and presented in this section. Baseline is defined as the last-available measurement obtained on or prior to randomization.

5.7.3 Summaries COVID-19 Impact on the Trial

The study was ongoing during the COVID-19 pandemic. Consequently, to monitor the potential impact of the pandemic on the trial, we will provide a specific set of descriptive analyses related to COVID-19, including:

- COVID-19 adverse event
- COVID-19-related protocol deviations
- Missed doses due to COVID-19
- Study discontinuations due to COVID-19
- Remote scale administrations
- Site actions and site closures

5.7.4 Pharmacokinetic and Pharmacodynamic Analyses

Mean serum and CSF crenezumab concentration versus time data will be tabulated and plotted, as appropriate. In serum, trough plasma concentration ($C_{trough,\ ss}$) will be tabulated and summarized (e.g., mean, standard deviation, minimum, and maximum). Additional PK analyses, such as evaluating relationships between crenezumab concentrations, PD biomarkers, efficacy and safety endpoints, may be conducted as appropriate. Mean plasma total $A\beta_{1\text{-}40}$ and $A\beta_{1\text{-}42}$ concentrations versus time will be tabulated and plotted, as appropriate. The relationship between crenezumab and total $A\beta_{1\text{-}40}$ and $A\beta_{1\text{-}42}$ concentrations will be tabulated and plotted to explore the peripheral PK/PD relationship*, as appropriate*.

The schedule for PK and PD analyses will be independent of the safety and efficacy endpoints.

5.8 ANALYSES IN TAU PET LONGITUDINAL SUBSTUDY BN40199

In this section, we outline the main statistical details in the tau PET longitudinal Substudy BN40199. The analysis populations in this substudy are described in detail in Section 4.

The primary endpoint of the tau PET longitudinal substudy is the annualized rate of change in the tau PET SUVr in the entorhinal cortex (Braak Stage 1), and other ROIs, including whole cortical gray matter, may be explored based on evidence in presymptomatic ADAD populations. The analysis population is based on mITT among the mutation carriers. The primary analysis in the tau PET longitudinal substudy will be performed using an RCRM. This model will include the actual tau PET SUVr as the response variable. The model set up is similar to the RCRM in the primary analysis model in the main study.

The 95% CI and p-value for crenezumab treatment effect will be presented. The estimated crenezumab treatment effect compared with the placebo group on the RR scale will also be presented. *Similar to secondary imaging endpoints (Sections 5.4.1 and 5.4.2)*, at the initial study readout, the Sponsor will analyze at least the *first* and last-available (in time) tau PET data. If the result from the main Study GN28352 is positive (at least one of the primary endpoints is statistically significant), the Sponsor will further analyze all available tau PET data and the p-value will be instead based on the full data.

The MMRM analysis may also be used to explore the treatment benefit over time. Additional descriptive summaries that describe the relationship between tau PET SUVr and biomarker and clinical endpoints are specified in Section 1.1.

Summaries of conduct of the substudy and summaries of demographic and baseline characteristics will be presented in a similar fashion to the main study; the analyses will be based on the ITT population; randomized non-carriers will also be grouped and presented in this section.

The main safety analyses planned for Study Period A (see Section 5.6) of the safety data collected during the tau substudy will also be performed in the tau substudy safety-evaluable population (see Section 4 for definition).

5.9 INTERIM ANALYSES

No interim analyses to be conducted.

5.10 ANALYSES FOR PERIOD B AND OPEN-LABEL EXTENSION STUDY

This section will pre-specify the main analyses planned for Study Period B of this study and the potential OLE *study*.

If the Sponsor decides to terminate the study based on the primary analysis results from Study Period A, all the data and analyses from Study Period B will be considered as exploratory in nature; a final CSR will be written, including these extra data. However, if the Sponsor decides to *extend Study Period B or* initiate *an* OLE *study*, a planned *data snapshot* will occur *2 years after finished the last efficacy visit in Study Period A.*

Three analysis populations are defined for this snapshot:

- Study Period A mITT population: the mITT population defined in Section 4: all randomized participants who received at least 1 dose of study drug; participants are grouped according to the treatment assignment at randomization; all efficacy analyses in this snapshot are based on mITT population among the mutation carriers
- Study Period B safety population: all randomized participants that received at least 1 dose of study drug in Study Period B; participants are grouped according to the actual treatment received; participants on placebo who received more than 2 doses of crenezumab will be grouped in the crenezumab arm
- OLE safety population: all OLE enrolled participants that received at least 1 dose of study drug (crenezumab)

5.10.1 Efficacy Analyses

All efficacy analyses will include data from Study Period A, Study Period B, and the OLE *study*; the analysis population will be based on mITT. *The delayed start efficacy analyses will be triggered 2 years after the last efficacy visit in Study Period A.* Two types of efficacy analyses are prespecified depending on the endpoint type: delayed-start analyses for continuous endpoints and rank-preserving structural failure time (RPSFT) analyses for time-to-event endpoints.

5.10.1.1 Delayed-Start Analyses

The delayed-start analysis will be used for all continuous efficacy endpoints that will be collected in *Study Period B and* the *potential* OLE *study*. Since *the potential* OLE *will not be published by the finalization of this SAP*, the description in this section is in general terms and does not focus on any specific endpoint. However, statistical testing will be performed only on the API ADAD Composite Cognitive Test total score and the FCSRT Cueing Index. Other continuous efficacy endpoints, including clinical endpoints, fluid biomarkers, and imaging biomarkers, will be considered exploratory and thus, formal statistical testing will not be done.

Figure 4 illustrates the concept of the delayed-start analysis. As shown in the figure, a treatment effect is assumed in Period A of Study GN28352; the disease progression slope of mutation carriers from the Study Period A crenezumab arm is assumed to be the same in Study Period B and in the OLE; the disease progression slope of mutation carriers from the Study Period A placebo group; however, will have a different slope in Study Period B and in the OLE. The statistical model details can be found in

Appendix 3. To control the type I error within the delayed-start analyses, the fixed testing sequence is specified in the following figure. The α level in each testing is a two-sided 0.05. If at any stage the null hypothesis H_0 is not rejected, then the formal testing procedure will stop.

Figure 4 Illustration of Delayed-Start Analyses Using Data from the Beginning of Period A of Study GN28352 to the End of ***Study Period B or*** **OLE**

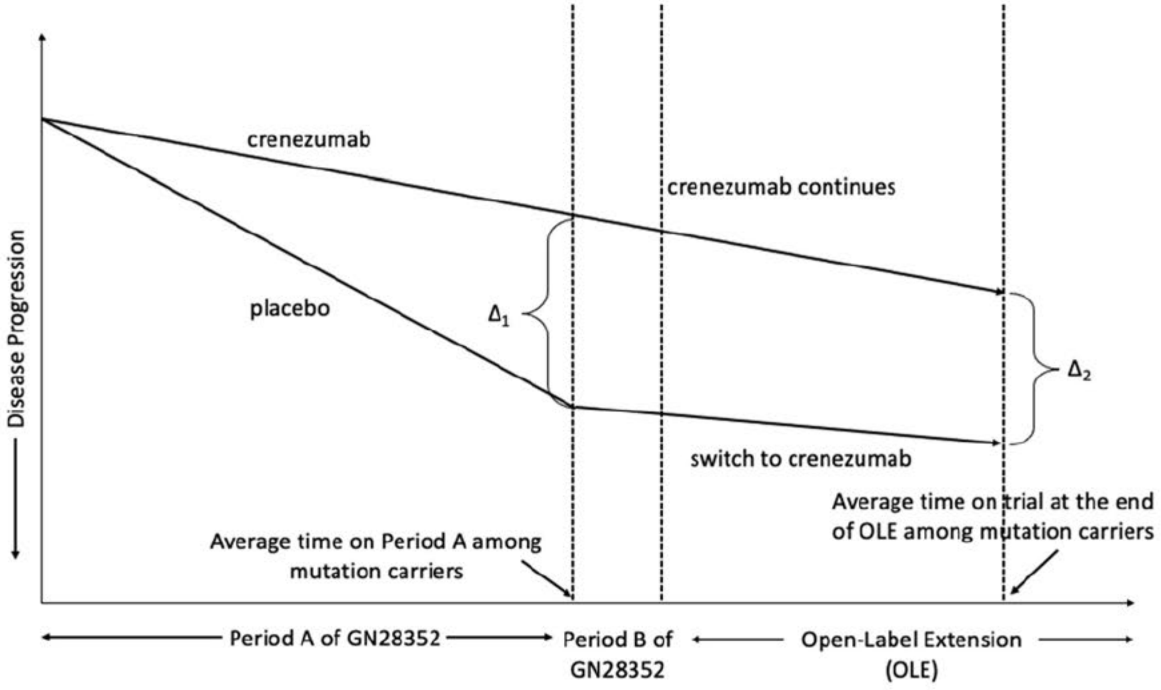

Figure 5 Testing Hierarchy for Delayed-Start Analyses

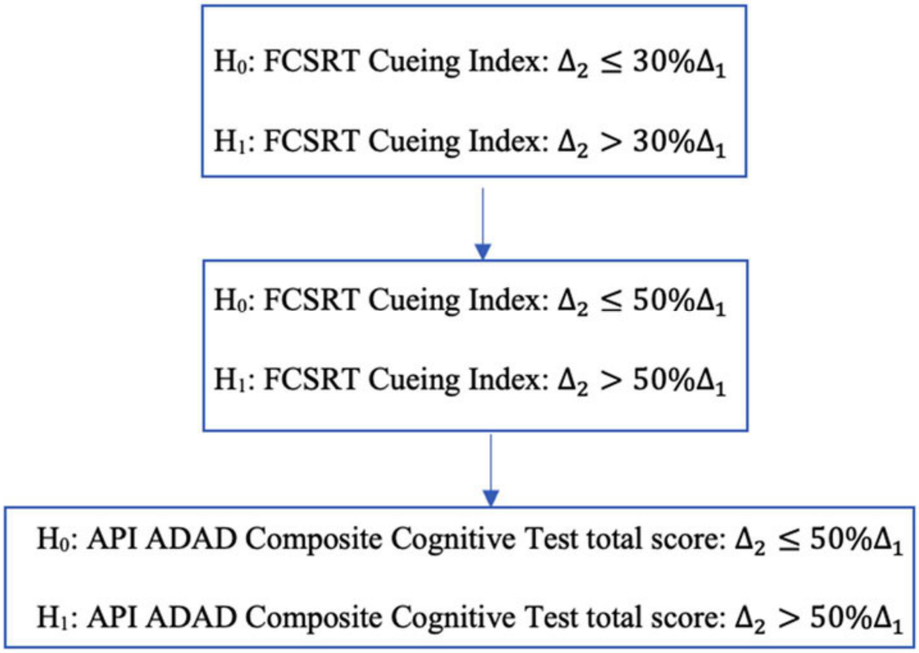

API = Alzheimer's Prevention Initiative; ADAD = autosomal-dominant Alzheimer's disease; FCSRT = Free and Cued Selective Reminding Task.

Note: For a definition of H0 and H1, please see Section 2.

5.10.1.2 Rank-Preserving Structural Failure Time Analyses

The RPSFT analysis will be used for the 2 time-to-event (TTE) endpoints below, also collected in Period B of Study GN28352 and the OLE. The TTE endpoints will anchor the randomization date from Period A of Study GN28352 as the reference date.

- Time to progression to non-zero in CDR global score
- Time to progression from preclinical AD to MCI due to AD or from preclinical AD to dementia due to AD

The Sponsor will ensure that the TTE endpoints will remain as objective/unbiased as possible in Period B of Study GN28352 and the OLE. *During* Period B of Study GN28352, the Sponsor will be unblinded to the mutation status and treatment assignment when analyzing the Study Period A results. *However,* participants, contract research organization staff, and blinded site staff will remain blinded *to treatment allocation in Study Period A*. Furthermore, an independent adjudication committee will still be in place to adjudicate cases deemed by the site that have progressed from preclinical AD to MCI due to AD or from preclinical AD to dementia due to AD. If the Sponsor initiates the OLE after the results of Study GN28352 are available, we anticipate that *only mutation carriers will be eligible for* the OLE.

The RPSFT method as introduced by Robins and Tsiatis (1991) provides an estimate of the overall event time for participants randomized into placebo group in Study Period A had treatment switching not occurred. With this model, we assume that the crenezumab treatment effect on event time is the same regardless of when crenezumab is given. It estimates overall event time measured from the time of treatment switching by applying an estimate of the benefit of crenezumab (derived iteratively and referred to as the inverse of the acceleration factor). The treatment switching does not happen for participants randomized into the crenezumab group in Study Period A; therefore, their event time can be directly used. The stratified log rank test, where the stratification factors are from Study Period A, will then be used to calculate the two-sided p-value; the HR for recurrence will be estimated using a stratified Cox proportional hazards model, and the 95% CI for the HR will be provided. Some stratification factor(s) may be removed from the stratified analyses if there is risk of non-convergence or sparse strata.

5.10.2 Safety Analyses

If the Sponsor decides *to terminate the study based on the primary analysis results from Study Period A*, then safety data from Study Period B will be reported in the final CSR. The Study Period B safety population is defined in the beginning of Section 5.10.

If the Sponsor decides to *extend Study Period B or* initiate *an* OLE, *then safety data from Study Period B will be reported in the final CSR and* safety data from the OLE will be summarized *in the OLE CSR*. The OLE safety population is defined in the beginning of Section 5.10.

All the safety analyses described in Section 5.6 will be reported.

5.10.3 Other Analyses

Summaries of conduct of study and summaries of treatment group comparability/demographics and baseline characteristics will also be presented. The population is based on either the Study Period B ITT population or the OLE ITT population, depending on whether the Sponsor initiates the OLE. The analyses are similar to what are specified for Study Period A.

Pharmacokinetic and PD analyses will be handled in a similar fashion to Study Period A.

6. SUPPORTING DOCUMENTATION

This section is not applicable, since there is no additional supporting document.

Appendix 1 Changes to Protocol-Planned Analyses

The following changes have been made in this Statistical Analysis Plan (SAP) from the latest Protocol Version 10:

- The definition of the safety population for Study Period B has been updated in the SAP
- Adverse events reported during the 16-week follow-up period in Study Period A will not be summarized separately

Appendix 2 Blinded Power Analysis and its Impact on Type I Error

This section outlines the methodology that the Sponsor used for the blinded power (BP) analysis. We then provide the evidence of family-wise type I error control when a BP analysis is performed. *More details of this methodology can be found in (Hu et al. 2022).*

METHODOLOGY

Assuming a BP analysis is performed on p continuous endpoints: $X^{(1)}, X^{(2)}, \dots, and\ X^{(p)}$, right before study unblinding. The goal of the BP analysis is to help ensure that the primary analysis meets the statistical power criteria defined in the protocol.
For simplicity and without loss of generality, we also assume the following:

- All endpoints are expected to decrease overtime in the mutation carrier placebo arm
- n/2 participants randomized to the placebo arm and n/2 participants randomized to the mutation carrier experimental arm, with no dropout
- Change from baseline scores at the last visit are calculated from each participant (statistic denoted as $X_i^{(k)}$ for the kth endpoint for the ith participant), with $X_i^{(k)} \sim N\left(\mu_{pbo}^{(k)}, {\sigma^{(k)}}^2\right)$ if participant i is from the placebo arm and $X_i^{(k)} \sim N\left(\mu_{exp}^{(k)}, {\sigma^{(k)}}^2\right)$ if participant i is from the experimental arm
- **The same treatment effect in relative reduction (RR) scale R_a is assumed in the analysis across endpoints** (note that R_a differs from the true underlying treatment effect $R^{(k)} = \frac{\mu_{pbo}^{(k)} - \mu_{exp}^{(k)}}{\mu_{pbo}^{(k)}} * 100\%$)

From Friede and Kieser (2013), the scaled sample variance $\frac{n-1}{{\sigma^{(k)}}^2} {S^{(k)}}^2 \sim$ Noncentral chi-squared distribution with n-1 degrees of freedom and noncentrality parameter $\frac{n\left(\mu_{pbo}^{(k)} R^{(k)}\right)^2}{4{\sigma^{(k)}}^2}$ and

$$E\left({S^{(k)}}^2\right) = {\sigma^{(k)}}^2 + \frac{n}{4(n-1)}\left(\mu_{pbo}^{(k)} R^{(k)}\right)^2.$$

Since the number of participants from the placebo and experimental arms both consist of ½ of the total population, respectively, then we can construct the following equation.

$$\frac{\hat{\mu}_{pbo}^{(k)} + \hat{\mu}_{pbo}^{(k)}(1 - R_a)}{2} = \overline{X^{(k)}}.$$

From the previous 2 equations, we can estimate the placebo mean decline and standard deviation for endpoint k:

$$\hat{\mu}_{pbo}^{(k)} = \frac{2\overline{X^{(k)}}}{2 - R_a}$$

$$\hat{\sigma}^{(k)^2} = S^{(k)^2} - \frac{n}{4(n-1)}\left(\frac{2R_a\overline{X^{(k)}}}{2-R_a}\right)^2.$$

When we assume the same relative treatment effect R_a across p continuous endpoints, finding the endpoint with the highest statistical power is equivalent to finding the endpoint with the smallest CV in the placebo arm. We have:

$$\widehat{CV}^{(k)} = \frac{\sqrt{\hat{\sigma}^{(k)^2}}}{\hat{\mu}_{pbo}^{(k)}} = \sqrt{\left(\frac{2-R_a}{2}\right)^2 \frac{S^{(k)^2}}{\overline{X^{(k)}}^2} - \frac{nR_a^2}{4(n-1)}}$$
$$= \sqrt{\left(\frac{2-R_a}{2}\right)^2 \widehat{CV}_{pooled}{}^{(k)^2} - \frac{nR_a^2}{4(n-1)}}.$$

We can see that $\widehat{CV}^{(k)}$ is a monotonically increasing function of $\widehat{CV}_{pooled}{}^{(k)^2}$, which implies that endpoints can be evaluated based on their relative $\widehat{CV}_{pooled}{}^{(k)^2} = \frac{S^{(k)^2}}{\left(\overline{X^{(k)}}\right)^2}$ using the blinded data.

The BP analysis just described in the proceeding bears similarities to blinded sample size re-estimation. In blinded sample size re-estimation, the updated sample size depends on the targeted treatment effect and observed pooled sample variance. Likewise, our BP analysis depends on multiple targeted treatment effects and the observed pooled CVs. It is important to note that neither case requires unblinded data.

It has been reported that blinded sample size re-estimation does not lead to substantive type I error inflation (Friede and Kieser 2006; Glimm and Läuter 2013). Next, we prove that analytically, BP analysis controls family-wise error rate (FWER) in *both the strong* the weak sense. We then provide simulations which support FWER control in the strong sense.

TYPE I ERROR CONTROL UNDER BP ANALYSIS

We present the theorem under the assumption that *at least one of the* p endpoints *has* no treatment effect.

<u>Theorem 1</u>

Among the p endpoints $\{X^{(1)}, X^{(2)}, \ldots, X^{(p)}\}$ *in* the *BP analysis, if there is at least one endpoint that has no treatment effect, the family-wise* type I error is controlled under the BP analysis.

Proof: We start by writing out the density function of the endpoint vector for participant i. If we define the endpoint vector $X_i = \left[X_i^{(1)}, X_i^{(2)}, \ldots, X_i^{(p)}\right]^T$ *and if we assume*

the endpoints considered follow a multi-normal distribution, then we have that $X_i \sim N(L_i\mu, \Sigma)$, *where:*

$$L_i = \begin{bmatrix} l_i & 0 & \dots & 0 & 1-l_i & 0 & \dots & 0 \\ 0 & l_i & \dots & 0 & 0 & 1-l_i & \dots & 0 \\ \vdots & \vdots & \ddots & \vdots & \vdots & \vdots & \ddots & \vdots \\ 0 & 0 & \dots & l_i & 0 & 0 & \dots & 1-l_i \end{bmatrix},$$

$$\mu = \left[\mu_{pbo}^{(1)}, \mu_{pbo}^{(2)}, \dots, \mu_{pbo}^{(p)}, \mu_{exp}^{(1)}, \mu_{exp}^{(2)}, \dots, \mu_{exp}^{(p)}\right]^T,$$

$$\Sigma = \begin{bmatrix} {\sigma^{(1)}}^2 & \cdots & \sigma^{(1)(p)} \\ \vdots & \ddots & \vdots \\ \sigma^{(1)(p)} & \cdots & {\sigma^{(p)}}^2 \end{bmatrix}.$$

The constant l_i *takes the value of either 0 or 1 (participant i in the experimental group or placebo group). We denote the parameter vector* $\theta = (\mu^T, vec(\Sigma)^T)^T$. *We also denote* $\mu_{pbo} = \left[\mu_{pbo}^{(1)}, \mu_{pbo}^{(2)}, \dots, \mu_{pbo}^{(p)}\right]^T$ *and* $\mu_{exp} = \left[\mu_{exp}^{(1)}, \mu_{exp}^{(2)}, \dots, \mu_{exp}^{(p)}\right]^T$. *The density function of* X_i *is:*

$$f(X_i|\theta, L_i) = \frac{1}{\sqrt{(2\pi)^p|\Sigma|}} exp\left(-\frac{1}{2}(X_i - L_i\mu)^T \Sigma^{-1}(X_i - L_i\mu)\right).$$

We define functions:

$$\eta(\theta) = \begin{pmatrix} \begin{bmatrix} \Sigma^{-1}\mu_{pbo} \\ \Sigma^{-1}\mu_{exp} \end{bmatrix} \\ -\frac{1}{2}\Sigma^{-1} \end{pmatrix},$$

$$T(X_i) = \begin{pmatrix} L_i^T X_i \\ X_i X_i^T \end{pmatrix},$$

$$h(X_i) = (2\pi)^{-\frac{p}{2}},$$

$$A(\theta) = \frac{1}{2}(L_i\mu)^T \Sigma^{-1} L_i\mu + \frac{1}{2} log\,|\Sigma|.$$

The dot product between $\eta(\theta)$ *and* $T(X_i)$ *is calculated as:*

$$\eta(\theta) \cdot T(X_i) = \begin{bmatrix} \Sigma^{-1}\mu_{pbo} \\ \Sigma^{-1}\mu_{exp} \end{bmatrix}^T L_i^T X_i + < \left(-\frac{1}{2}\Sigma^{-1}\right)^T, X_i X_i^T >_F = \mu^T L_i^T \Sigma^{-1} X_i - \frac{1}{2} tr\left(\Sigma^{-1} X_i X_i^T\right),$$

where $<,>_F$ is the Frobenius inner product and "tr(.)" stands for the trance of a matrix. The density function can then be written as:

$$f(X_i|\theta, L_i) = (2\pi)^{-\frac{p}{2}} exp\left(-\frac{1}{2}(X_i - L_i\mu)^T \Sigma^{-1}(X_i - L_i\mu) - \frac{1}{2}log|\Sigma|\right)$$
$$= h(X_i)\, exp\big(\eta(\theta) \cdot T(X_i) - A(\theta)\big).$$

Therefore, the multivariate normal distribution is in the exponential family with the parameter defined as $\theta = (\mu^T, vec(\Sigma)^T)^T$. The statistic $\sum_{i=1}^n T(X_i) = \begin{pmatrix} \sum_{i=1}^n L_i^T X_i \\ \sum_{i=1}^n X_i X_i^T \end{pmatrix}$ is then both complete and sufficient for θ (Theorems 6.2.10 and 6.2.25 from Casella and Berger 2021). The final t-test statistic t_k is clearly free of the parameter vector θ; therefore, t_k is an ancillary statistic to θ. Based on Basu's theorem, the testing statistic t_k is independent of $\sum_{i=1}^n T(X_i)$. Since the statistics $\frac{S^{(k)^2}}{\left(\overline{X^{(k)}}\right)^2}$ $(k = 1,2, \dots, p)$ in the BP analysis are all functions of $\sum_{i=1}^n T(X_i)$, then the testing statistic t_k is also independent of $\frac{S^{(k)^2}}{\left(\overline{X^{(k)}}\right)^2}$ $(k = 1,2, \dots, p)$.

We assume the final t-test is a two-sided test with the significance level of 0.05. If the experimental drug has no effect on all p endpoints, then the family-wise type I error is:

$$\alpha = \sum_{k=1}^{p} P\left(\frac{S^{(k)^2}}{\left(\overline{X^{(k)}}\right)^2} = min\left\{\frac{S^{(1)^2}}{\left(\overline{X^{(1)}}\right)^2}, \frac{S^{(2)^2}}{\left(\overline{X^{(2)}}\right)^2}, \dots, \frac{S^{(p)^2}}{\left(\overline{X^{(p)}}\right)^2}\right\}, |t_k| \geq t_{0.05,n-2}\right)$$
$$= \sum_{k=1}^{p} P\left(\frac{S^{(k)^2}}{\left(\overline{X^{(k)}}\right)^2} = min\left\{\frac{S^{(1)^2}}{\left(\overline{X^{(1)}}\right)^2}, \frac{S^{(2)^2}}{\left(\overline{X^{(2)}}\right)^2}, \dots, \frac{S^{(p)^2}}{\left(\overline{X^{(p)}}\right)^2}\right\}\right)$$
$$* P\big(|t_k| \geq t_{0.05,n-2}\big) = 1 * 0.05 = 0.05.$$

If the experimental drug has no effect only in the first l endpoint (without loss of generality) but has effect in the remaining endpoints, then the family-wise type I error is:

$$\alpha = \sum_{k=1}^{l} P\left(\frac{S^{(k)^2}}{\left(\overline{X^{(k)}}\right)^2} = min\left\{\frac{S^{(1)^2}}{\left(\overline{X^{(1)}}\right)^2}, \frac{S^{(2)^2}}{\left(\overline{X^{(2)}}\right)^2}, \dots, \frac{S^{(p)^2}}{\left(\overline{X^{(p)}}\right)^2}\right\}, |t_k| \geq t_{0.05,n-2}\right)$$
$$= \sum_{k=1}^{l} P\left(\frac{S^{(k)^2}}{\left(\overline{X^{(k)}}\right)^2} = min\left\{\frac{S^{(1)^2}}{\left(\overline{X^{(1)}}\right)^2}, \frac{S^{(2)^2}}{\left(\overline{X^{(2)}}\right)^2}, \dots, \frac{S^{(p)^2}}{\left(\overline{X^{(p)}}\right)^2}\right\}\right)$$
$$* P\big(|t_k| \geq t_{0.05,n-2}\big) \leq 1 * 0.05 = 0.05.$$

We have proved that the family-wise type I error is not inflated under the BP analysis, both in the strong and weak sense.

Next, we show through simulations *to support the proof.* For simplicity, we assume the BP analysis is performed on 2 endpoints X and Y, where the experimental drug has no treatment effect on X but *could have* a treatment effect on Y.

Mimicking study GN28352, we assume the following:

- 84 participants randomized to the placebo arm and 84 participants randomized to the experimental arm, with no dropout
- Endpoints X and Y both follow a normal distribution within each arm
- Within endpoints X and Y, without loss of generality, the standard deviation in both arms is 1
- Endpoint X has no treatment effect and the placebo arm mean is 1/0.65 (protocol CV assumption on API ADAD Composite Cognitive Test total score = 0.65)

In the simulation study, we vary the following, which yields 135 scenarios:

- Treatment effect in endpoint Y in RR scale: 0%, 50%, and 100%
- Placebo mean in endpoint Y: 0, 1, 1.5, 2, 10
- *Endpoints X and Y follow a bivariate normal distribution with*
  - Correlation between endpoint X and Y within the placebo group: ρ_{pbo} = 0.1, 0.5, 0.9
  - Correlation between endpoint X and Y within the treatment group: ρ_{exp} = 0.1, 0.5, 0.9

The family-wise type I error in this case equals the probability of rejecting the null H_0 in endpoint X. *When there is a treatment effect in endpoint Y, the* only scenario where a type I error can happen is when endpoint X is chosen by the BP analysis. The number of iterations in the simulation study was 100,000. We summarize the simulation results on type I error (along with the probability of choosing endpoint Y *in scenarios where endpoint Y has a treatment effect*) in Tables 1–3.

Table 1 Family-Wise Strong Type I Error, Assuming 0% Treatment Effect in Endpoint Y (*Type I Error in the Weak Sense*)

		Placebo Mean in Endpoint Y				
		**0**	**1**	**1.5**	**2**	**10**
$\rho_{pbo} = 0.1$	$\rho_{exp} = 0.1$	0.05090	0.04968	0.0583	0.04965	0.04862
	$\rho_{exp} = 0.5$	0.04928	0.04963	0.04995	0.05029	0.04983
	$\rho_{exp} = 0.9$	0.05042	0.04981	0.05012	0.04894	0.05068
$\rho_{pbo} = 0.5$	$\rho_{exp} = 0.1$	0.04949	0.04898	0.04953	0.04982	0.05075

		Placebo Mean in Endpoint Y				
	$\rho_{exp} = 0.5$	0.05052	0.05048	0.05014	0.04953	0.04962
	$\rho_{exp} = 0.9$	0.04913	0.05105	0.05016	0.04927	0.04969
$\rho_{pbo} = 0.9$	$\rho_{exp} = 0.1$	0.05074	0.05068	0.05055	0.04959	0.05099
	$\rho_{exp} = 0.5$	0.05010	0.05002	0.05012	0.04951	0.05088
	$\rho_{exp} = 0.9$	0.04969	0.05063	0.04900	0.05004	0.04970

Table 2 Family-Wise Strong Type I Error with Probability of Choosing Endpoint Y under the Blinded Power Analysis, Assuming 50% Treatment Effect in Endpoint Y $(Type\ I\ Error\ in\ the\ Strong\ Sense)$

		Placebo Mean in Endpoint Y				
		0	1	1.5	2	10
$\rho_{pbo} = 0.1$	$\rho_{exp} = 0.1$	0.04927 (0%)	0.05127 (0%)	0.05072 (0.03%)	0.04689 (9.0%)	0.00000 (100%)
	$\rho_{exp} = 0.5$	0.04948 (0%)	0.05005 (0%)	0.04937 (0%)	0.04570 (6.5%)	0.00000 (100%)
	$\rho_{exp} = 0.9$	0.04993 (0%)	0.05038 (0%)	0.05005 (0%)	0.04308 (2.9%)	0.00000 (100%)
$\rho_{pbo} = 0.5$	$\rho_{exp} = 0.1$	0.04936 (0%)	0.04950 (0%)	0.05061 (0.01%)	0.04590 (8.1%)	0.00000 (100%)
	$\rho_{exp} = 0.5$	0.04934 (0%)	0.04995 (0%)	0.04996 (0%)	0.04585 (5.4%)	0.00000 (100%)
	$\rho_{exp} = 0.9$	0.05039 (0%)	0.05019 (0%)	0.04961 (0%)	0.04601 (1.9%)	0.00000 (100%)
$\rho_{pbo} = 0.9$	$\rho_{exp} = 0.1$	0.04943 (0%)	0.05038 (0%)	0.05132 (0%)	0.04672 (5.8%)	0.00000 (100%)
	$\rho_{exp} = 0.5$	0.04975 (0%)	0.05020 (0%)	0.04959 (0%)	0.04746 (3.0%)	0.00000 (100%)
	$\rho_{exp} = 0.9$	0.05086 (0%)	0.04916 (0%)	0.04940 (0%)	0.04895 (0.4%)	0.00000 (100%)

Table 3 Family-Wise Strong Type I Error with Probability of Choosing Endpoint Y under the Blinded Power Analysis, Assuming 100% Treatment Effect in Endpoint Y *(Type I Error in the Strong Sense)*

		Placebo Mean in Endpoint Y				
		**0**	**1**	**1.5**	**2**	**10**
$\rho_{pbo} = 0.1$	$\rho_{exp} = 0.1$	0.05030 (0%)	0.05097 (0%)	0.05008 (0%)	0.05070 (0%)	0.04979 (0%)
	$\rho_{exp} = 0.5$	0.04990 (0%)	0.04985 (0%)	0.04988 (0%)	0.05013 (0%)	0.04838 (0%)
	$\rho_{exp} = 0.9$	0.04908 (0%)	0.04976 (0%)	0.05006 (0%)	0.05013 (0%)	0.05044 (0%)
$\rho_{pbo} = 0.5$	$\rho_{exp} = 0.1$	0.04964 (0%)	0.05094 (0%)	0.05063 (0%)	0.05004 (0%)	0.04949 (0%)
	$\rho_{exp} = 0.5$	0.05021 (0%)	0.05089 (0%)	0.05002 (0%)	0.05042 (0%)	0.05016 (0%)
	$\rho_{exp} = 0.9$	0.04965 (0%)	0.04976 (0%)	0.05033 (0%)	0.04960 (0%)	0.05064 (0%)
$\rho_{pbo} = 0.9$	$\rho_{exp} = 0.1$	0.05039 (0%)	0.05153 (0%)	0.04978 (0%)	0.05014 (0%)	0.04924 (0%)
	$\rho_{exp} = 0.5$	0.05148 (0%)	0.04994 (0%)	0.04925 (0%)	0.04939 (0%)	0.05031 (0%)
	$\rho_{exp} = 0.9$	0.04936 (0%)	0.05032 (0%)	0.05038 (0%)	0.04999 (0%)	0.05082 (0%)

As seen from Tables 1–3, the simulated family-wise type I errors are at or substantially below 0.05. *Also in scenarios where the treatment effect in Y is non-zero*, when the probability of choosing endpoint Y approaches to 100%, the family-wise type I error approaches to 0*%; this can be seen in the proof in Theorem 1.*

We conclude that the BP analysis does not inflate family-wise type I error both weakly or strongly.

Appendix 3 Details on Selected Statistical Models

In this section, we present the details on selected statistical models from this Statistical Analysis Plan (SAP).

CONTROL-BASED MEAN IMPUTATION SENSITIVITY ANALYSIS MODEL FROM SECTION 5.3.3

In the analysis using control-based mean imputation, the treatment effect in annualized rate of change is quantified as

$$\hat{\beta}_x[c] = f_{cren}^{comp}\left(\hat{\beta}_{cren}^{comp} - \hat{\beta}_{pbo,MAR} - c\right) + c,$$

where f_{cren}^{comp} is the percentage of participants from the crenezumab arm who have the score collected from the end of treatment visit A (ET-A)/end of treatment visit B (ET-B) visit; $\hat{\beta}_{cren}^{comp}$ is the RCRM estimated annualized rate of change when using the data only from the participants from the crenezumab arm who have the primary endpoint collected from the ET-A/ET-B visit; $\hat{\beta}_{pbo,MAR}$ is the RCRM estimated annualized rate of change when using the data only from the participants from the placebo arm; c is a non-positive constant (note that the sign of c is different from Mehrotra et al. (2017) because our primary endpoints are expected to decline over time) which will be addressed in the following paragraph. The variance of this treatment effect estimator is estimated as:

$$\hat{V}\left(\hat{\beta}_x[c]\right) = \left(f_{cren}^{comp}\left(1 - f_{cren}^{comp}\right)N_{cren}^{-1} + \left(f_{cren}^{comp}\right)^2\right)\left(\hat{V}_{cren}^{comp} + \hat{V}_{pbo,MAR}\right) + \left(f_{cren}^{comp}\left(1 - f_{cren}^{comp}\right)N_{cren}^{-1}\left(\hat{\beta}_{cren}^{comp} - \hat{\beta}_{pbo,MAR} - c\right)^2\right),$$

where N_{cren} is the number of participants in the crenezumab arm from the mITT population; $\hat{V}_{cren}^{comp}$ is the variance estimate on the RCRM estimated annualized rate of change when using the data only from the participants from the crenezumab arm who have the primary endpoint collected from the ET-A/ET-B visit; $\hat{V}_{pbo,MAR}$ is the variance estimate on the RCRM estimated annualized rate of change when using the data only from the participants from the placebo arm. The degrees of freedom corresponding to $\hat{V}_{cren}^{comp}$ and $\hat{V}_{pbo,MAR}$ using the Kenward and Roger method (Kenward and Roger 1997) are denoted as λ_{cren}^{comp} and $\lambda_{pbo,MAR}$, respectively. The degrees of freedom of the treatment effect estimator can be calculated as:

$$\lambda[c] = \frac{\left(\hat{V}\left(\hat{\beta}_x[c]\right)\right)^2}{\frac{\left(\hat{V}_{cren}^{comp}\right)^2}{\lambda_{cren}^{comp}} + \frac{\left(\hat{V}_{pbo,MAR}\right)^2}{\lambda_{pbo,MAR}}}.$$

We will then perform the imputation based on c=0. This scenario assumes that the mean slope from the participants with missing values from the crenezumab arm is the same as the estimated overall placebo slope. We note that this assumption is already conservative in estimating the treatment effect because it implicitly assumes participants

on treatment will even do worse than an average placebo participant, after dropping out of the study.

DELAYED-START ANALYSIS MODEL FROM SECTION 5.10.1.1

The statistical model for this delayed-start analysis can be written as:

$$Y_{ij} = \alpha + (\text{Randomization Factors})_i + u_{0i} + \left(\beta + \beta_x I_{x,i} + u_{1i}\right)t_{ij} + \left(\beta_{x,B/CAS}t_{ij} - \beta_{x,B/OLE}T_i\right)\left(1 - I_{x,i}\right)I_{t_{ij}>T_i} + \epsilon_{ij},$$

where β_x quantifies the crenezumab annualized treatment effect in slowing down the disease progression in Period A of Study GN28352 and $\beta_{x,B/OLE}$ quantifies the treatment effect by switching from placebo to crenezumab in Period B of Study GN28352 and the OLE; T_i represents the time of the first endpoint assessment after participant i enters into Study Period B or the OLE.

Due to the common-close design of Study GN28352, mutation carriers will have varied duration of follow-up at the end Study Period of A. To calculate the crenezumab treatment effect at the end of Study Period A, we first calculate the arithmetic mean of duration in Study Period A (last endpoint assessment date – randomization date in Study Period A + 1) among mutation carriers and we call this duration $\bar{T}_1$. On average, the treatment benefit in slowing down the disease progression at the end of Study Period A can be written as:

$$\Delta_1 = \beta_x \bar{T}_1.$$

Similarly, we also calculate the arithmetic average of overall duration at the end of the OLE among mutation carriers and we call this duration $\bar{T}_2$. The average treatment benefit remained at the end of OLE can be written as:

$$\Delta_2 = \left(\beta_x - \beta_{x,B/OLE}\right)\bar{T}_2 + \beta_{x,B/OLE}\bar{T}_1.$$

Note that both $\bar{T}_1$ and $\bar{T}_2$ are treated as constants and are estimated from the data. A non-inferiority test with different margins will be applied (Liu-Seifert et al. 2015). A 90% CI will be calculated for $\Delta_2 - 50\%\Delta_1$ or $\Delta_2 - 30\%\Delta_1$, depending on the specified margin in the test; if the lower limit of the CI is greater than 0, then the H_0 will be rejected, meaning that by the end of the OLE at least 50% (or 30%) of the treatment effect at the end of Study Period A is preserved.

7. REFERENCES

Acosta-Baena N, Sepulveda-Falla D, Lopera-Gómez CM, et al. Pre-dementia clinical stages in presenilin 1 E280A familial early-onset Alzheimer's disease: a retrospective cohort study. Lancet Neurol 2011;10:213–20.

Albert MS, DeKosky ST, Dickson D, et al. The diagnosis of mild cognitive impairment due to Alzheimer's disease: recommendations from the National Institute on Aging-Alzheimer's Association workgroups on diagnostic guidelines for Alzheimer’s disease. Alzheimers Dement 2011;7:270–9.

Armitage SG. An analysis of certain psychological tests used for the evaluation of brain injury. Psychological Monographs 1946;60:i–48.

Bateman RJ, Xiong C, Benzinger TL, et al. Clinical and biomarker changes in dominantly inherited Alzheimer's disease. N Engl J Med 2012;367:795–804.

Bretz F, Maurer W, Brannath W, et al. A graphical approach to sequentially rejective multiple test procedures. Stats Med 2009;28:586–604.

Bretz F, Posch M, Glimm E, et al. Graphical approaches for multiple comparison procedures using weighted Bonferroni, Simes, or parametric tests. Biom J 2011;53:894–913.

Casella G, Berger RL. Statistical inference. Cengage Learning 2021, 2nd edition.

Caselli RJ, Langlais BT, Dueck AC, et al. Neuropsychological decline up to 20 years before incident mild cognitive impairment. Alzheimers Dement 2020;16:512–23.

Chen K, Langbaum JB, Fleisher AS, et al. Twelve-month metabolic declines in probable Alzheimer's disease and amnestic mild cognitive impairment assessed using an empirically pre-defined statistical region-of-interest: findings from the Alzheimer's Disease Neuroimaging Initiative. Neuroimage 2010;51:654–64.

Cummings JL, Mega M, Gray K, et al. The Neuropsychiatric Inventory: comprehensive assessment of psychopathology in dementia. Neurology 1994;44:2308–14.

Cummings JL. The Neuropsychiatric Inventory: assessing psychopathology in dementia patients. Neurology 1997;48:S10–6.

Donohue MC, Sperling RA, Salmon DP, et. al. The preclinical Alzheimer cognitive composite: measuring amyloid-related decline. JAMA Neurol 2014;71:961–70.

Flaherty KR, Wells AU, Cottin V, et al. Nintedanib in progressive fibrosing interstitial lung diseases. New Engl J Med 2019;381:1718–27.

Folstein MF, Folstein SE, McHugh PR. "Mini-mental state." A practical method for grading the cognitive state of patients for the clinician. J Psychiatr Res 1975;12:189–98.

Friede T, Kieser M. Blinded sample size re-estimation in superiority and noninferiority trials: bias versus variance in variance estimation. Pharm Stat 2013;12:141–6.

Friede T, Kieser M. Sample size recalculation in internal pilot study designs: a review. Biom J 2006;48:537–55.

Glimm E, Läuter J. Some notes on blinded sample size re-estimation. arXiv 2013 [resource on the Internet]. Available from: https://arxiv.org/pdf/1301.4167v1.pdf.

Gollan TH, Weissberger G, Runnqvist E, et al. Self-ratings of spoken language dominance: a multi-lingual naming test (MINT) and preliminary norms for young and aging Spanish-English bilinguals. Biling (Camb Engl) 2012;15:594–615.

Hu N, Mackey H, Thomas R. Power and sample size for random coefficient regression models in randomized experiments with monotone missing data. Biom J 2021;63:806–24.

[redacted], *et al. Adapative endpoint selection in randomized clinical trials. Manuscript in preparation. 2022.*

Huber PJ. The behavior of maximum likelihood estimates under nonstandard conditions. Berkeley Symposium on Mathematical Statistics and Probability 1967:221–3.

Kenward MG, Roger JH. Small sample inference for fixed effects from restricted maximum likelihood. Biometrics 1997;53:983–97.

Liu-Seifert H, Andersen SW, Lipkovich I, et al. A novel approach to delayed-start analyses for demonstrating disease-modifying effects in Alzheimer’s disease. PloS One 2015;10:e0119632.

McKhann GM, Knopman DS, Chertkow H, et al. The diagnosis of dementia due to Alzheimer's disease: recommendations from the National Institute on Aging-Alzheimer's Association workgroups on diagnostic guidelines for Alzheimer’s disease. Alzheimers Dement 2011;7:263–9.

Mehrotra DV, Liu F, Permutt T. Missing data in clinical trials: control-based mean imputation and sensitivity analysis. Pharm Stat 2017;16:378–92.

Mohs RC, Knopman D, Petersen RC, et al. Development of cognitive instruments for use in clinical trials of antidementia drugs: additions to the Alzheimer’s Disease Assessment Scale that broaden its scope. Alzheimer Dis Assoc Disord 1997;11:S13–21.

Morris JC, Heyman A, Mohs RC, et al. The Consortium to Establish a Registry for Alzheimer's Disease (CERAD). Part I. Clinical and neuropsychological assessment of Alzheimer's disease. Neurology 1989;39:1159–65.

Morris JC. The Clinical Dementia Rating (CDR): current version and scoring rules. Neurology 1993;43:2412–14.

Randolph C, Tierney MC, Mohr E, et al. Repeatable Battery for the Assessment of Neuropsychological Status. San Antonio, TX: The Psychological Coporation, 1998.

Raven JC. Revised Manual for Raven's Progressive Matrices and Vocabulary Scale. New York: The Psychological Corporation, Harcourt Brace Jovanovich, Inc., 1976.

Richeldi L, du Bois RM, Raghu G, et al. Efficacy and safety of nintedanib in idiopathic pulmonary fibrosis. New Engl J Med 2014;370:2071–82.

Robins JM, Tsiatis AA. Correcting for non-compliance in randomized trials using rank preserving structural failure time models. Communications in Statistics-Theory and Methods 1991;20:2609–31.

Rosen WG, Mohs RC, Davis KL. A new rating scale for Alzheimer's disease. Am J Psychiatry 1984;141:1356–64

Sclan SG, Reisberg B. Functional assessment staging (FAST) in Alzheimer's disease: reliability, validity, and ordinality. Int Psychogeriatr 1992;4:55–69.

Sheikh JI and Yesavage JA. Geriatric Depression Scale (GDS): recent evidence and development of a shorter version. Clinical Gerontology 1986;5:165–73.

Sperling RA, Aisen PS, Beckett LA, et al. Toward defining the preclinical stages of Alzheimer's disease: recommendations from the National Institute on Aging-Alzheimer's Association workgroups on diagnostic guidelines of Alzheimer's disease. Alzheimers Dement 2011;7:280–92.

Tounsi H, Deweer B, Ergis AM, et al. Sensitivity to semantic cuing: an index of episodic memory dysfunction in early Alzheimer disease. Alzheimer Dis Assoc Disord 1999;13:38–46.

U.S. Food and Drug Administration (FDA). Multiple endpoints in clinical trials: guidance for industry [resource on the Internet] 2017. Available from: https://www.fda.gov/files/drugs/published/Multiple-Endpoints-in-Clinical-Trials-Guidance-for-Industry.pdf.

van Dyck CH, Nygaard HB, Chen K, et al. Effect of AZD0530 on cerebral metabolic decline in Alzheimer disease: a randomized clinical trial. JAMA Neurol 2019;76:1219–29.

White H. Maximum likelihood estimation of misspecified models. Econometrica 1982;50:1–25.
